# Uncharacterized conserved motifs outside the HD-Zip domain in HD-Zip subfamily I transcription factors; a potential source of functional diversity

**DOI:** 10.1186/1471-2229-11-42

**Published:** 2011-03-03

**Authors:** Agustín L Arce, Jesica Raineri, Matías Capella, Julieta V Cabello, Raquel L Chan

**Affiliations:** 1 Instituto de Agrobiotecnología del Litoral, Universidad Nacional del Litoral, CONICET, CC 242 Ciudad Universitaria, 3000, Santa Fe, Argentina

## Abstract

**Background:**

Plant HD-Zip transcription factors are modular proteins in which a homeodomain is associated to a leucine zipper. Of the four subfamilies in which they are divided, the tested members from subfamily I bind *in vitro *the same pseudopalindromic sequence CAAT(A/T)ATTG and among them, several exhibit similar expression patterns. However, most experiments in which HD-Zip I proteins were over or ectopically expressed under the control of the constitutive promoter 35S CaMV resulted in transgenic plants with clearly different phenotypes. Aiming to elucidate the structural mechanisms underlying such observation and taking advantage of the increasing information in databases of sequences from diverse plant species, an *in silico *analysis was performed. In addition, some of the results were also experimentally supported.

**Results:**

A phylogenetic tree of 178 HD-Zip I proteins together with the sequence conservation presented outside the HD-Zip domains allowed the distinction of six groups of proteins. A motif-discovery approach enabled the recognition of an activation domain in the carboxy-terminal regions (CTRs) and some putative regulatory mechanisms acting in the amino-terminal regions (NTRs) and CTRs involving sumoylation and phosphorylation. A yeast one-hybrid experiment demonstrated that the activation activity of ATHB1, a member of one of the groups, is located in its CTR. Chimerical constructs were performed combining the HD-Zip domain of one member with the CTR of another and transgenic plants were obtained with these constructs. The phenotype of the chimerical transgenic plants was similar to the observed in transgenic plants bearing the CTR of the donor protein, revealing the importance of this module inside the whole protein.

**Conclusions:**

The bioinformatical results and the experiments conducted in yeast and transgenic plants strongly suggest that the previously poorly analyzed NTRs and CTRs of HD-Zip I proteins play an important role in their function, hence potentially constituting a major source of functional diversity among members of this subfamily.

## Background

### Plant transcription factors

Transcription factors (TFs) play key roles in signal transduction pathways in all living organisms. They are proteins able to recognize and bind specific DNA sequences (*cis*-acting elements) present in the regulatory regions of their target genes. In general, these proteins have a modular structure and exhibit at least two types of domains: a DNA binding domain and a protein-protein interaction domain which mediates, directly or indirectly, the activation or repression of transcription [1].

In plants, several TF families have been identified but only a relatively small number of members have been functionally studied [2,3]. Such identification was performed essentially in plants whose genome has been sequenced, e.g. Arabidopsis, for which a comparison with known animal TFs indicated the existence of about 2000 TFs [3,4]. TF families are classified according to their binding domain and divided in subfamilies according to additional structural and functional characteristics [2,5-9].

### The HD-Zip family of transcription factors

Among the identified TF families, the HD-Zip family is composed of proteins bearing a homeodomain associated to a leucine zipper (hereafter, HD and HALZ), association unique to plants. Due to this specific association and knowing that HD proteins in other kingdoms are involved in development, HD-Zip proteins were proposed as key players in plant specific developmental processes, such as those associated to external stimuli and stresses [10]. Four groups, named I to IV, have been identified fundamentally based on four particular characteristics: sequence conservation within the HD-Zip domain, the presence of additional conserved domains, gene structure and the pathways in which these proteins participate (for a review see [9] and [11]). HD-Zip III and IV members are, on average, the largest proteins; they exhibit a START (STeroidogenic Acute Regulatory protein-related lipid Transfer) and SAD (START adjacent) domains towards the C-terminus in relation to the HD-Zip domain [9], plus a MEKHLA (called after the goddess of lightning, water and rain) domain in subfamily III proteins [12]. HD-Zip II TFs also have a distinguishing feature in their C-terminus, the CPSCE motif responsible for redox regulation of protein activity [13], and the ZIBEL motif in their N-terminus [11]. No common feature outside the HD-Zip domain has been assigned to subfamily I TFs.

### What is known about HD-Zip subfamily I members

HD-Zip I group has 17 members in *Arabidopsis thaliana *divided in six classes according to their phylogenetic relationships and intron/exon distribution: α (*ATHB3, -20, -13 *and-*23*), β (*ATHB1, -5, -6 *and -*16*), γ (*ATHB7 *and -*12*), δ (*ATHB21, -40 *and -*53*), ε (*ATHB22 *and -*51*) and φ (*ATHB52 *and -*54*) [14]. The encoded proteins tested for binding specificity *in vitro *recognize the same pseudopalindromic sequence with the highest affinity [15-17]. This affinity, but not the specificity of this protein-DNA interaction is affected by the aminoacids of the homeodomain N-terminal arm [18].

ATHB7 and ATHB12, coded by paralogous genes, share 80% identity in the HD-Zip domain amino acid sequence. Both genes are regulated by drought stress in an abscicic acid (ABA)-dependent way [19,20]. Their developmental expression pattern is similar but *ATHB12 *expression is detectable in lateral root primordia, young leaves and inflorescence stems while *ATHB7 *is not, at least under normal growth conditions. When ABA is exogenously applied, their expression patterns overlap [21,22]. The constitutive expression of *ATHB7 *in the Wassilewskija (WS) genotype generates a developmental delay and a characteristic morphological phenotype (similar to the observed when WT plants are subjected to drought) while the silencing of this gene apparently does not alter the phenotype [21]. *ATHB12 *overexpressors are similar to *ATHB7 *transgenic plants [22]. Both transgenic genotypes presented also increased lateral branching of the stem compared with the WT (WS) genotype. In both cases the phenotype in roots is ABA dependent while the phenotype in stems is ABA independent [22,23]. The characterization of *athb12 *mutants and ATHB12 overexpressing plants indicated that this gene product is somehow inhibiting the expression of the gene encoding the GA-20-oxidase, leading to the short stem phenotype due to a reduction in gibberellins content [23].

Our research group has characterized HAHB4, a sunflower HD-Zip I protein sharing 60% and 53% identity respectively with ATHB7 and -12 in the HD-Zip domain [24]. However, HAHB4 has a short carboxy-terminal region (CTR, 64 amino acids after the HALZ) while ATHB7 and 12 present 127 and 106 amino acids in this region, respectively. *HAHB4 *expression is very low in normal growth conditions and it is up regulated in roots, stems and leaves by ABA, mannitol, NaCl, drought and darkness as well as by jasmonic acid (JA) and ethylene (ET) [24-28]. The phenotype observed when this sunflower gene is ectopically expressed in Arabidopsis plants strongly resembles that of *ATHB7/12 *overexpressing plants [29]. However, *HAHB4 *plants exhibited drought tolerance and a senescence delay while *ATHB7 *and *12 *did not. Moreover, when *HAHB4 *seedlings were treated with exogenous ACC (1-aminocyclopropane-1-carboxylic-acid, a precursor of ET biosynthesis) the plants did not present the typical triple response to ethylene [26]. This observation together with a microarray analysis indicated that HAHB4 inhibits the expression of ethylene receptors and thereafter the ability to sense this hormone [26,28].

Another pair of paralogous genes, *ATHB13 *and *ATHB23*, code for proteins which share 78% identity in the HD-Zip domain and 87 and 77% identity, respectively, with the HD-Zip domain of the sunflower HAHB1 [30]. The morphological characteristics of transgenic plants expressing *ATHB13 *and *HAHB1 *genes under the CaMV 35S promoter are similar; e.g. serrated leaves, differential cotyledons phenotype when grown in sucrose 4% [[31,32]; JV Cabello, AL Arce, and RL Chan, unpublished results].

### Is the HD-Zip domain sufficient for the function of HD-Zip I TFs?

The proteins encoded by the above mentioned genes (i.e., ATHB12, 13, 23; HAHB4 and HAHB1), ATHB5, ATHB1 and CPHB1 bind *in vitro *with maximal affinity the same target sequence [15-17,33]. Notably when transgenic plants in which these or other HD-Zip I encoding genes were expressed in Arabidopsis under the CaMV 35S promoter, the resultant phenotypes were clearly different with the exception of those genes phylogenetically closely related. These facts strongly suggest that the function of these genes may be significantly determined by other characteristics in addition to differences in expression patterns and target gene preferences.

In this sense, previous works have supported the functionality of the CTRs of HD-Zip I proteins. It was shown that this portion of ATHB12 is capable of transcriptional activation in yeast one-hybrid experiments [34] and functional complementation of a NaCl-sensitive calcineurin (CaN)-deficient yeast mutant, only when the protein has a complete CTR [35].

Sakuma *et al. *[36] identified *HvHox2*, a putative paralogue of *VRS1*, by observing the effect caused in the *Hordeum vulgare *spikelets development. These two genes, both encoding HD-Zip I proteins, differ particularly in the CTR. HvHox2 exhibits 14 additional amino acids compared with VRS1. These authors identified a conserved motif in this portion of the protein and suggested that it could interact with certain classes of co-activators in order to exert its biological function, as it has been proposed for HAHB4 [36,37].

TL (Tendril-less) is a garden pea HD-Zip protein which mutation (*tl*) generates plants with a particular phenotype: tendrils are converted to leaflets, they are no longer inhibited from completing laminar development. Notably, a mutant in which this gene codes for a protein lacking 12 amino acids in its CTR exhibited the same phenotype as a mutant unable to express the gene [38].

Based on the literature data and on our own observations we aimed to put in evidence that the CTRs and NTRs (amino terminal regions) may be playing an important role in the signalling networks in which HD-Zip proteins participate, determining to some extent their functionality. We used bioinformatics to detect new sequence motifs in the NTRs and CTRs of the HD-Zip I proteins. Further, we experimentally tested the function of a CTR by making chimeric constructs and uncovered a motif specific function.

## Results

### Phylogenetic analysis of HD-Zip proteins from different species resolved six different clades

An *in silico *analysis was performed on a set of 178 sequences from HD-Zip I transcription factors from different species (Additional file 1). They were selected merging the database of proteins from species with sequenced genomes [11] and a set retrieved from NCBI's Conserved Domain Architecture Retrieval Tool (CDART).

The initial approach involved the construction of three phylogenetic trees: the first with the subsequences comprising the HD and the HALZ domains of each protein (named HZT), the second with this same subset plus three HD-Zip II proteins from Arabidopsis which were used as outgroup (HZT + OG), and the last with the complete sequences of the proteins (named CST). The subset of sequences used for the HZT and HZT + OG was obtained using HMMer [39] and the corresponding HMM models [40].

**Figure 1:**
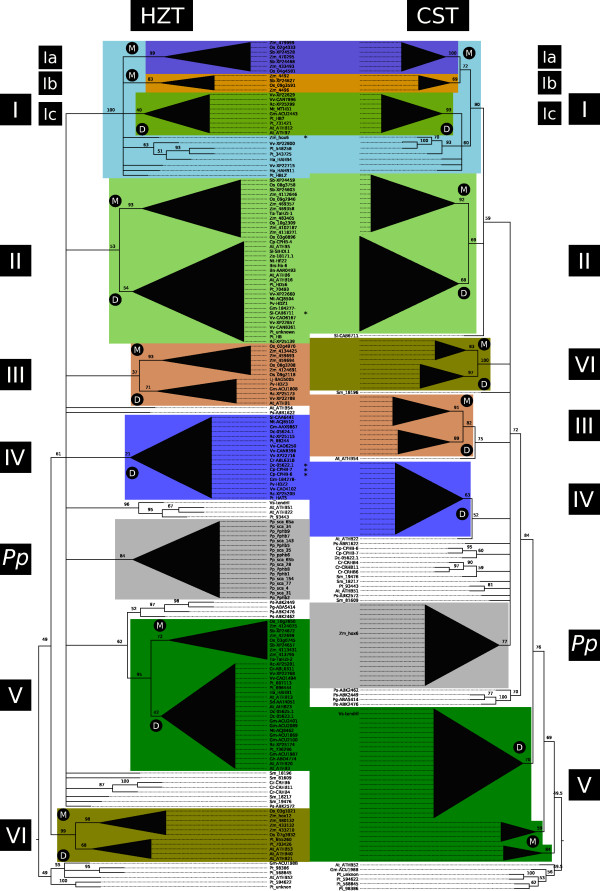
**Phylogenetic trees of HD-Zip I transcription factors**. Maximum Likelihood phylogenetic trees were constructed using the amino acid sequences of 178 HD-Zip subfamily I transcription factors from different plant species including monocots, dicots, mosses, ferns and conifers. The HZT was constructed with the sequences of the HD and HALZ domains and is the reference tree. The CST was calculated with the complete sequences. Clades highlighted with different colours represent groups of transcription factors sharing common motifs in their CTRs. These clades are numbered from I to VI whereas group I is divided in three subgroups named Ia, Ib and Ic. Inside these groups, clades exclusively formed by monocots or dicots transcription factors were labelled with an M or a D, respectively; and their structure was collapsed to ease visualization. Proteins shared between groups in the HZT and CST have been erased from the CST. Unshared members have been marked with an asterisk in the HZT. The group labelled *Pp *includes all the proteins from the moss *Physcomitrella patens*. Bootstrap support values, as percentages, are indicated in the nodes. Branches with low bootstrap values (below 50%) have been collapsed, with the exception of the basal branches of groups Ic, III and IV in the HZT which have further support from bootstrap values in the CST (see Table 1) and conserved motifs in the NTRs and CTRs (Figures 2, 4 and Additional file 8).

The sequences were aligned with MAFFT (Additional files 2 and 3) [41] and maximum-likelihood phylogenetic trees constructed using PhyML [42] with 100 bootstrap replicates for the HZT and HZT + OG, and 144 bootstrap replicates for the CZT (Figure 1 and Additional files 4 and 5). As expected, the three HD-Zip II proteins formed a separate clade in the HZT + OG and its relative location was used to root the three trees.

**Figure 2:**
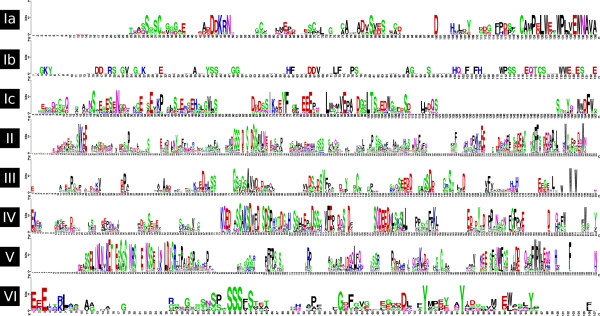
**Sequence logos of CTRs from the six groups identified**. The sequence logos were constructed with the alignment of the CTRs of the proteins belonging to each of the six groups, including subgroups Ia, Ib and Ic. The height of the residues correlates with their frequency in the alignment, which allows the recognition of clearly conserved regions.

The HZT was considered the reference tree because it was constructed with a sequence alignment obtained exclusively from the sites which are homologous to all the HD-Zip I proteins analyzed. The initial strategy involved the comparative analysis of the HZT and CST, and the manual inspection of the alignment of the complete sequences. Overall, major clades with moderate or good statistical support in the HZT and CST had, with some exceptions, a very similar composition. Sequence alignments in the NTRs and CTRs revealed evident sequence conservation for most proteins in each clade. Based on both observations, a total of 137 proteins were divided in six groups (I to VI, Figure 1 and Additional file 4). As can be seen in Figure 2 and Additional file 6, each group has a reasonably distinctive CTR with variable-length stretches of highly conserved amino acids. The informational content in these regions can be appreciated by the increase in bootstrap values for most of these clades in the CST where the NTRs and CTRs are considered (Table 1).

**Table 1:** Bootstrap values in the HZT and the CST

	I	Ia	Ib	Ic	II	III	IV	V	VI
HZT	**100**	99	83	40	53	37	21	95	99

CST	90	**100**	**69**	**93**	**69**	**82**	**63**	pph	**100**

Bootstrap values for the different clades identified in the trees HZT and CZT. pph paraphyletic.

Grouping was mainly aimed at recognizing common potentially functional characteristics in the sequences of groups of HD-Zip I proteins. Consequently, although group I had a high bootstrap support value, it was further divided in three subgroups: Ia, Ib and Ic; according to sequence conservation, particularly in the CTR (Figure 2). Conversely, the conservation in the NTR and CTR (Figure 2) of proteins from groups III and IV together with the significant bootstrap values in the CST supported grouping of clades of proteins with weak bootstrap values in the HZT.

Groups I, II, III, V and VI were formed of proteins from dicots and monocots, excluding the 27 proteins from mosses, lycophytes, ferns and conifers; and 14 proteins from dicots. The 17 TFs from the moss *Physcomitrella patens *formed a separate clade named *Pp *group. The species with sequenced genomes had at least one member in each group, with the exception of Poplar in group III and Arabidopsis in group IV, the only group exclusively formed of proteins from dicots.

### The high conservation of key residues in the HD-Zip I homeodomains suggests little target-sequence variation

Certain residues in the HD, particularly in the helix III and a flexible N-terminal arm are important determinants of the sequence preferentially bound by the HD-Zip I TFs [18,43,44]. The alignment of the HD and HALZ sequences corresponding to the proteins of the dataset analyzed (Additional file 2) shows a very high conservation of the amino acids in these homeodomain positions, i.e.: K2: 74%, K3: 94%, R5: 93%, I/V47: 54/46%, Q50: 100%, N51: 99% and R55: 100% (corresponding to the positions K4, K5, R7, I/V57, Q60, N61 and R65 in the alignment, Additional file 2). This result suggests that target-sequence variation may not be a major source of functional diversity within the subfamily I of HD-Zip TFs.

### HD-Zip proteins from each clade present conserved motifs in their CTRs

**Figure 3:**
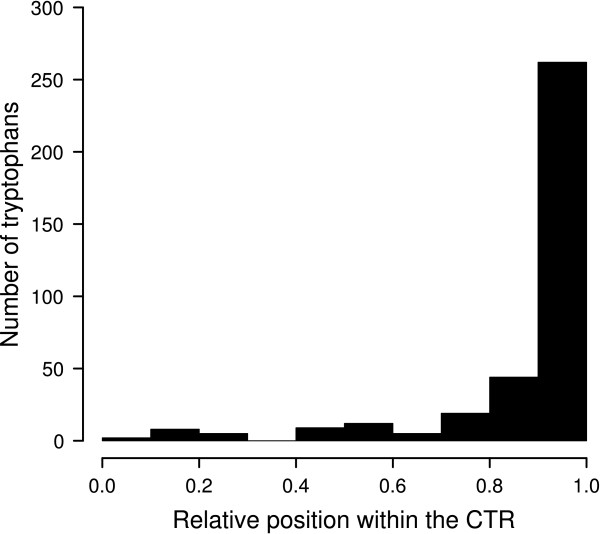
**Frequencies of tryptophans in the CTRs**. The histogram represents the frequencies of Trp within the CTRs of the 178 proteins according to their relative position in this region, which was divided in ten parts. The last tenth shows a visible enrichment.

Previous experimental evidence supporting the functionality of the CTRs of a few HD-Zip I proteins [34-36,38] lead us to further explore this region. From the alignment of the CTRs, the only evident feature was a bias in W composition towards the last residues of the protein. The histogram in Figure 3 shows that W was significantly enriched in the final tenth part of the CTRs of the 178 proteins studied.

**Figure 4:**
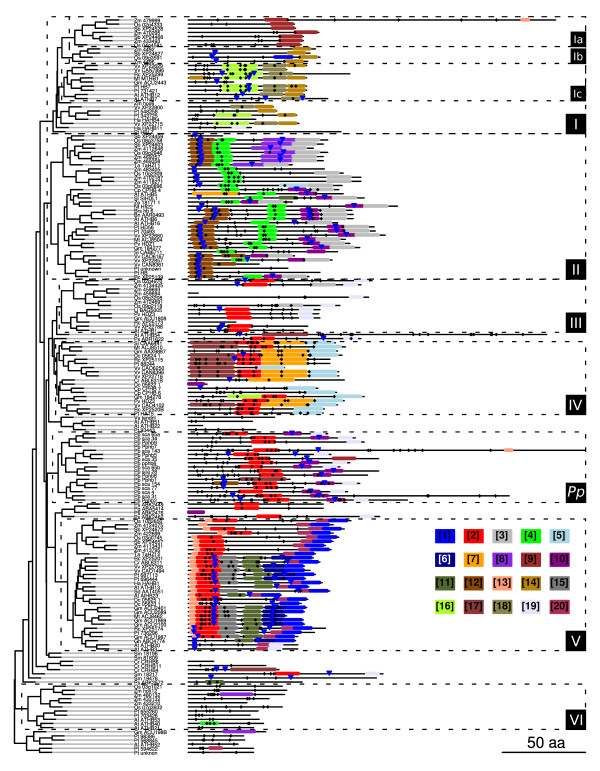
**Motif location in the CTRs**. The 20 motifs found by the program MEME are depicted according to their location in each CTR. The identity of each motif is colour coded according to the legend. Groups are highlighted with a box of dashed boundaries and the phylogenetic relations between the proteins are indicated by the tree on the left side of the plots. Putative phosphorylation sites (Ser, Thr, Tyr) are marked with a black diamond and sumoylation motifs with a blue inverted triangle.

**Figure 5:**
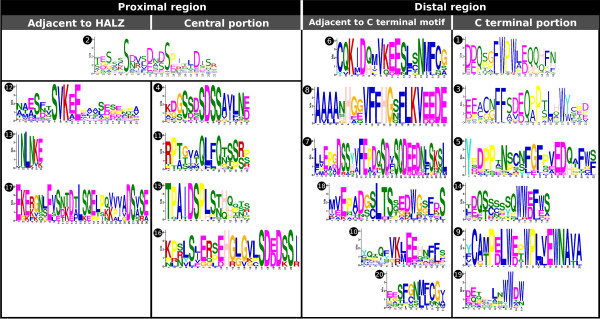
**Sequence logos of the motifs found in the CTRs**. The sequence logos of the 20 motifs found in the CTRs are sorted according to their relative position. To reflect chemical properties in the distal region, the motifs present in the same row are also combined in many CTRs (except for motifs 9, 10, 19 and 20; some alternative combinations to those shown also exist).

In order to deepen the analysis of the CTRs, a motif discovery approach was conducted using the MEME program [45]. A single run with all the sequences (with a limit of 20 motifs and a minimum width of six sites) yielded motifs with e-values ranging from 4.3e-279 to 3.9e-27. Figure 4 illustrates the motif composition and location in each CTR; the sequence logos of each motif are presented in Figure 5.

Most of the motifs found were highly or completely group specific, only group VI lacked distinctive motifs. Nonetheless, there is one clear exception: motif 2 appears in most members of groups III, IV and V and in many *P. patens *proteins. Its distinguishing features are: an enrichment in Ser with two occupying conserved positions separated by six residues, and several acidic amino acids.

On the basis of motif distribution, the CTR could be roughly divided in two regions: a proximal region, adjacent to the HALZ; and a distal region, comprising the final part of the protein. The former involved up to three concatenated motifs adjacent to the HALZ (in groups II, V, IV) and/or a motif located around the central portion of the CTR (Figure 4); while the latter was characterized by a motif covering the last residues, which in groups Ic, II, IV, V and *Pp *was accompanied by an adjacent motif towards the N-terminus (Figure 4).

The analysis of the different motifs according to their position and composition revealed a remarkable feature; the presence of one or more Trp with high frequencies in all the motifs at the end of the proteins (motifs 1, 3, 5, 9, 14, 19, Figures 2 and 5). Another aromatic amino acid with high frequencies was Phe, present in most of the motifs in the distal region (motifs 1, 3, 5, 7, 10, 14 and 20). Additionally, many positions were occupied by acid residues and a few by Pro (motifs 1, 3, 5, 7 and 9). This sequence features highly resemble those of AHA motifs found in HSF (Heat Stress Transcription Factors) TFs [46].

In the motifs found in the proximal region of the CTR, the residues with the highest frequencies were Ser and acidic amino acids (Figure 5). Since Ser are potential phosphorylation sites and transcription factors constitute preferential candidates for this type of modification [47], we explored the predicted possibility of phosphorylation in Ser, Thr and Tyr with the program NetPhos 2.0 [48]. Using a cutoff score of 0.9, the results showed that many of the high-frequency Ser in these motifs are predicted targets of phosphorylation, particularly those present in motifs 2, 4, 6, 7, 10, 12, 16, 17 and 18 (Figure 5 and Additional file 7), most of which were in the proximal region of the CTR (Figure 4).

Interesting results were obtained when another type of putative post-translational modification was analyzed, sumoylation. SUMO is mainly conjugated to the K in the motif ΨKXE/D (Ψ, large hydrophobic residue; X, any amino acid; E/D, Glu or Asp) [49]. This peptide appears with a high frequency in motifs 6, 8, 10 and 12; the last present in the proximal region and the other in the distal region, adjacent to the terminal motif. To further address this observation, we searched for the degenerated motif in all the CTRs, Ψ being F, V, I, M or L. The motif was found 143 times in 95 of the 178 proteins. Moreover, the last position was mostly E: the motif ΨKXE corresponds to 120 of the 143 motifs found, and they are distributed in 92 of the 95 proteins. There was also a bias towards the identity of the hydrophobic residue: V > I > L > M > F (62% > 19% > 11% > 6% > 2%). The sumoylation motifs were mainly present in groups I (b and c), II, V and the *Pp *group (Figure 4). In groups II and V they were found twice per protein.

As a rudimentary test of the significance of these results, the motif ΨKX-[ED] in which the last position could be any of the 20 amino acids but Glu or Asp was searched. A total of 82 motifs in 63 proteins were found, which compared to the appearances of the canonical motif (143 motifs in 95 proteins, (ΨKXE/D)/(ΨKX-[ED]): 1.74) puts in evidence the overrepresentation of putative SUMO conjugation sites.

### The NTRs also present some conserved motifs

The NTRs were analyzed applying a similar motif-discovery strategy. The program MEME elicited 12 motifs with e-values ranging from 2.6e-231 to 3.7e-4. Motifs logos and distribution are illustrated in Additional files 8 and 9. Group definition was somehow supported by this distribution, with some exceptions. Motif 1 is widely distributed appearing in groups II (dicots only), III, IV and *Pp*. Subgroups Ia, Ib and Ic lacked distinctive motifs, and group II was divided in monocots and dicots by unshared motifs. It should be noted that group VI, which had no distinctive motifs in the CTR, was distinguished by motif 10 in the NTR.

In the attempt of finding putative functional significance to the motifs of the NTR, the program NetPhos was employed to predict probable phosphorylation sites with a cutoff of 0.9 (Additional files 8 and 10). The Ser residues in motifs 1 (mostly from group I), 3 and 6 (position 10 with high frequency) are the best candidates for this post-traslational modification because they are also highly conserved.

The program NLStradamus [50] was used to predict nuclear localization signals (NLS) in the complete proteins. This signal was found only in 16 of the 178 proteins; among them, three had it in the CTR (ATHB54, Pp_sca_35 and Pp_sca_143, all three abnormally long HD-Zip I proteins), and the other 13 in the NTR. Of these 13, six NLSs belonged to proteins from group VI (11 members) and fell within motif 10, found in 10 of the members (Additional files 8 and 11).

In order to make a comparison with the sumoylation results obtained with the CTRs, the motif ΨKXE/D was searched in the NTRs. Only eight motifs were found (Additional file 8), seven exhibit a Glu in the last position and four of them a Val in the first position. Despite amino acid frequencies showed some analogy with those in sumoylation motifs found in the CTRs; the number of sites found is negligible to consider sumoylation an important general modification in HD-Zip I NTRs. To reinforce this conclusion, the motif ΨKX-[ED] was searched in the NTRs: it appeared 59 times in 53 proteins ((ΨKXE/D)/(ΨKX-[ED]): 0.14), in contrast with the results obtained with the CTRs ((ΨKXE/D)/(ΨKX-[ED]): 1.74).

### ATHB1 CTR acts as an activation domain in yeast cells

**Figure 6:**
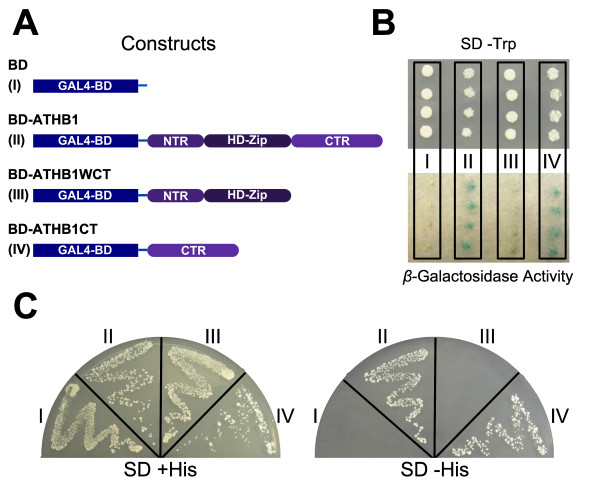
**ATHB1 CTR acts as an activation domain in yeast cells**. (A) The complete sequence of ATHB1, a version without the CTR (ATHB1WCT), and the CTR alone (ATH1CT) were fused to the DNA-binding domain of GAL4 (GAL4-BD). The empty vector expressing only the GAL4-BD was used as negative control. (B) A *β*-galactosidase activity assay. (C) Confirming this results, only the CTR and the complete ATHB1 protein had the transactivation activity required to reverse the auxotrophy to His of the AH109 yeast cells, allowing them to grow in medium lacking this amino acid.

In order to determine the putative activator action of the CTR motif in these TFs, one member of group III, ATHB1, was analyzed. Genetic constructs in which the whole cDNA or a mutated version, where the CTR was deleted, were obtained and yeast cells (AH109) were transformed with these as well as with the appropriate control constructs (Figure 6A). The positive colonies grown in the medium lacking Trp were transferred to a medium lacking His in which only the cells with the ability to transactivate can grow. Cells bearing the complete cDNA or just the CTR grew in this medium while the cells transformed with the truncated construct and those transformed with the empty vector did not (Figure 6C). The empty vector bears the ADH1 promoter directing the expression of the GAL4 transcription factor DNA-binding domain; this construct is not able to transactivate and therefore, the cells transformed with it cannot live in a selective medium. Colonies were also tested for *β*-galactosidase activity and the results supported the growth assay (Figure 6B). These observations indicated that the CTR is the region responsible for the transactivation activity of this TF, at least in yeast.

### The phenotype of the plants transformed with chimerical constructs is similar to that of the plants transformed with the CTR donor protein

In order to determine the importance of the CTR in the structure/function relationship of HD-Zip proteins, we have chosen two well characterized members of this transcription factors family to perform chimerical constructs and evaluate the phenotypes in transgenic plants. *HAHB4 *inhibits the triple response to ethylene when it is ectopically expressed in Arabidopsis while *HAHB1*, like its homologue *ATHB13*, confers a serrated shape to leaves [[26], JV Cabello, AL Arce; and RL Chan, unpublished results]. In relation to the *in silico *analysis, HAHB1 fell in group V and HAHB4 in group I, outside the three subgroups with characteristic CTRs. No motifs were found in HAHB4 NTR (relatively small, 19 amino acids) or CTR (62 amino acids); it has two Trp in the final residues, with the particularity of being adjacent to a Lys, not usual in AHA motifs. HAHB1 possesses motifs 13, 2, 11, 20 and 1 in the CTR (122 amino acids); and motifs 2, 3, 6 and 4 in the NTR (91 amino acids).

**Figure 7:**
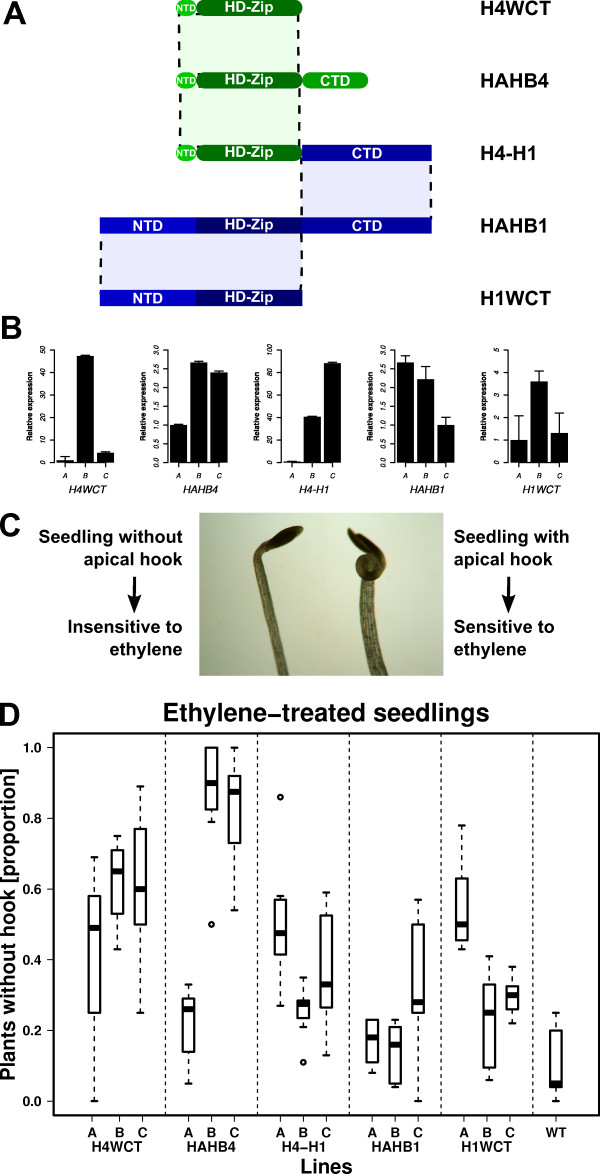
**Triple response to ethylene in chimerical transgenic plants**. (A) Schematic representation of the different constructs used to transform Arabidopsis plants (B) Relative expression levels of the different transgenes in independent lines measured by qPCR. The line with the lowest expression was assigned an unitary level (1). (C) The sensitivity to ethylene was measured analyzing whether the seedlings developed apical hook (sensitive) or not (insensitive). The image exemplifies the phenotypes observed. (D) The results for three different lines from each genotype are presented in the boxplot.

Mutant and chimerical genetic constructs were performed to evaluate the CTR functionality. The CTR of HAHB1 was fused to the HD-Zip of HAHB4 (protein H4-H1) and both cDNAs were deleted in their CTRs forming H1WCT (HAHB1 without CTR) and H4WCT (HAHB4 without CTR), as depicted in Figure 7A. Fused to the 35S CaMV promoter, these constructs were used to transform Arabidopsis plants. Three independent lines of each genotype presenting differential expression levels were chosen for further analysis (Figure 7B).

Seedlings were grown in 5 μM ACC, an ethylene precursor, and photographs taken when they were four-day-old. Figure 7C illustrates the phenotype observed for sensitive and insensitive plants while in Figure 7D the proportions of insensitive plants in eight groups of 20 plants from each line subjected to this treatment is depicted with a box plot. Transgenic plants with high expression levels of HAHB4 (lines B and C) were used as controls and did not show the apical hook, as expected, while a low expression-level line (line A) presented a high percentage of ACC sensitive plants. H4WCT exhibited a moderate insensitivity to ACC. H1WCT and H4-H1 plants showed more sensitivity than H4WCT plants. Finally, the plants which displayed the higher sensitivity to ACC treatment were HAHB1 and WT, showing a very low percentage of seedlings without apical hook (Figure 7D).

Notably, H4-H1 plants were more sensitive to the ACC treatment than HAHB4 plants but not as sensitive as HAHB1 plants, while H1WCT plants decreased their sensitivity to the treatment. Together these observations indicate that the CTR of HAHB1 in H4-H1 seriously impairs the physiological response triggered by HAHB4, more effectively than the removal of its own CTR (i.e., in H4WCT plants). In fact, H1WCT could, to some extent, mimic the physiological response of HAHB4 plants to ACC, questioning the degree of participation of the CTR when HAHB4 is involved in this pathway.

**Figure 8:**
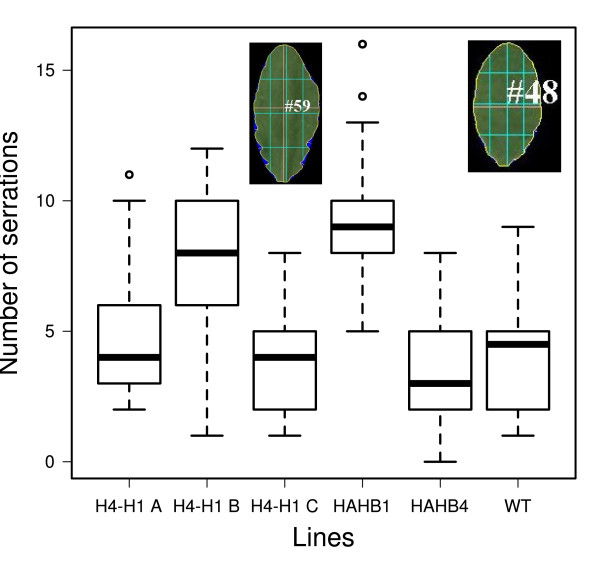
**Leaf serration phenotype**. Boxplot illustrating the serration grade of the leaves belonging to transgenic plants transformed with different constructs. The images are examples of the analysis conducted with the leaves (HAHB1 on the right and HAHB4 on the left).

The phenotype of rosette-leaf serration was also tested. The number of serrations per leaf was calculated for high expression lines of each genotype: WT, HAHB1 B, HAHB4 B and H4-H1 A, B and C plants. The results showed that HAHB1 B and H4-H1 B plants presented a clear increase in serration while the rest of the lines had a serration similar to that of WT plants (Figure 8). The quantifications were subjected to the Kruskal-Wallis one-way analysis of variance by ranks and then the different lines were classified in groups according to pairwise comparisons with a p-value of 0,05 (Table 2). The results indicated that HAHB1 and H4-H1 B had a statistically significant increase in serration. Together with H1-H4 A, these three lines were distinguishable from HAHB4 plants.

**Table 2:** Pairwise comparisons after Kruskal-Wallis test

Transgenic line	Rank	Groups		
HAHB4 B	50.36	A		

H4-H1 C	58.53	A	B	

WT	66.88	A	B	

H4-H1 A	78.53		B	

H4-H1 B	120.03			C

HAHB1 B	143.17			C

The sum of ranks of the Kruskal-Wallis test for each line is presented together with the groups resulting from the pair wise comparison. Lines with statistically different behaviours do not share the same group.

## Discussion

Transcription factors are modular proteins *par excellence *[51]. Among the many types of modules present in different TFs, two are almost indispensable: a DNA-binding domain and a protein-protein interaction domain which mediates activation or repression of transcription [1]. HD-Zip proteins are transcription factors unique to plants and since the isolation of the first member in 1992 [9,52], several works have informed that the protein-DNA interaction mediated by the HD is highly specific and needs as a prerequisite the dimerization of the TF through the HALZ [16,17,53]. Other domains outside the HD and HALZ are present in members from HD-Zip subfamilies III and IV (e.g., START, SAD domains; [9]). HD-Zip II TFs have a redox motif in their CTRs [13] and a Ziebel motif in their NTRs [11]. In the case of HD-Zip I proteins, no additional domains or motifs have been described for the whole group. Some reports suggested the presence of a nuclear localization sequence in their amino terminus [54]; however, no definite experimental evidence in this sense has been presented thus far. A few reports have provided evidence indicating a function for the CTR of these proteins. In this sense, activation activity was demonstrated as dependent on the CTR of ATHB12 in yeast [34]. Additional support to the importance of the CTR was provided by Sakuma et al. [36]; they identified that the recessive allele *vrs1*, which causes the six-rowed phenotype in barley, encodes an HD-Zip I TF 14 residues shorter in the CTR than its paralogous gene *HvHox2 *(both share 88% of identity in the whole protein), which was caused by a 300-bp insertion that introduced a stop codon. These authors identified a conserved motif within these 14 amino acids and suggested that this motif could interact with certain classes of co-activators in order to exert its biological function [36,37].

Recently, a pea deletion mutant in one HD-Zip protein, in which tendrils were converted into leaflets (they were no longer inhibited from completing laminar development), was shown to exhibit the same phenotype as a mutant in which the 12 amino acids of its CTR were not translated [38].

The starting point of our analysis was a 178 HD-Zip I protein dataset retrieved from CDART NCBI's database and that generated by Mukherjee et al. [11]. The first step involved the construction of three phylogenetic trees: the HZT with the HD and HALZ domains, the HZT + OG in which three HD-Zip II proteins were added as outgroup, and the CST with the complete sequences. The HD-Zip II TFs formed a clade which relative position was used to root the 3 threes. The HZT was considered the reference tree as its construction only used the sites homologous to all proteins. With the objective of identifying relevant functional regions outside the HD and HALZ domains, the sequence alignments of the NTRs and CTRs were inspected and the clades formed in the HZT and CST were compared. Based on these observations, six groups were identified (I to VI, Figure 1). The evident similarity in their CTRs, shared by most members of each group (Figure 2 and Additional file 6), strongly suggests the common ancestry of these proteins. This observation was statistically weakly supported for groups Ic, II, III and IV in the HZT; but was significantly supported by bootstrap values in the CST (Table 1). This suggests that the sequence of the HD and HALZ may not be sufficiently informative to clearly resolve some clades, thus the construction of a tree with the complete sequences proved to be a valuable strategy to identify and provide additional support to the hypothesis of relationship among the proteins in the different groups. Nonetheless, as the CST was constructed considering sites which are homologous only to subgroups of proteins, the phylogenetic reconstruction lays over a partially unfulfilled hypothesis of homology and the tree was not taken as the reference reconstruction. None of the six groups identified included proteins from species of non-flowering plants; they included only proteins from monocots and dicots (except for group IV exclusively formed of proteins from dicots). This suggests that a common ancestor of the genes encoding these proteins existed prior to the split of monocots and dicots, but no clear homologues could be found in the analyzed proteins from more basal species.

The 17 proteins from *Physcomitrella patens *formed a separate clade. This is potentially due to the low diversification of subfamily I HD-Zip TFs in this non-vascular plant. Rooting positioned this group as a non-basal group within clades of mostly flowering plant proteins. If the hypothesis of multiple gene losses in this species is considered unlikely, this presents incongruence with the known species phylogeny. This observation together with the relatively poor statistical support in the basal part of the tree (beyond the six groups identified) suggests that the precise relationship among groups could not be fully resolved. More sequences or a more complex approach might be needed to achieve it.

In the phylogeny of Arabidopsis HD-Zip I proteins obtained by Henriksson et al. [14] a classification in six classes was made. Proteins from class α (ATHB3, -20, -13 and-23) were included in group V, three proteins from class β (ATHB5, -6 and -16) were included in group II while ATHB1 fell in group III, proteins from class γ (ATHB7 and -12) were contained by group Ic, and proteins from class δ (ATHB21, -40 and -53) were included in group VI. None of the six groups included proteins from classes ε (ATHB22 and -51) and φ (ATHB52 and -54), moreover, the monophyly of class φ in relation to other Arabidopsis proteins is not preserved in the HZT (i.e., when phylogenetic relationships among Arabidopsis TFs is reconstructed from this tree, this class does not constitute a separate clade). Although the reconstruction of Henriksson et al. [14] is supported by additional information, (e.g., intron/exon distribution within the HD and HALZ domains), it should be noted that classes ε and φ are the only for which no support from duplication history was found by the authors. It should also be mentioned that ATHB54 is a very atypical HD-Zip I protein with an extremely long CTR (325 amino acids) in which a RNA recognition motif can be identified.

As mentioned before, HD-Zip I proteins tested *in vitro *bind specifically and with high affinity the same pseudopalindromic sequence CAAT(A/T)ATTG [16,17]. The alignment of the HD and HALZ of the proteins in the studied dataset showed a high degree of conservation for the residues responsible for sequence binding specificity [9]. Consequently, the putative ability of targeting and regulating different groups of genes may be significantly determined by other protein features outside the HD.

Numerous studies demonstrated that the expression of HD-Zip I proteins under the control of the CaMV 35S promoter [21-23,26-29,55] results in plants with diverse phenotypes. But considering that their DNA-binding specificity could be a minor source of functional variability, conclusion supported by our *in silico *analysis of the 178 protein dataset; the importance of the role of the poorly characterized NTRs and CTRs in the function of HD-Zip I TFs could have been overlooked. The sequence conservation found in these domains and the identification of different groups of TFs are in agreement with this notion.

In order to gain insight into the function of the conserved regions, a motif discovery strategy was carried out. As a result of running the program MEME on the NTRs and CTRs, 12 and 20 motifs were obtained, respectively. Many motifs were group specific/characteristic (Figure 4 and Additional file 8) but a few were more widely distributed. In general, the CTRs displayed more correlation between groups and motifs, although in this analysis no specific motifs were found in the CTR for group VI and the only motif present in subgroup Ib (monocots) was also in subgroup Ic (dicots). The motifs in the NTRs were only specific for groups II (being in monocots different than in dicots), VI (opposed to what happened in the CTR), V and *Pp*. Groups III and IV shared motifs in the NTR and subgroups Ia, Ib and Ic had no characteristic motifs in this region.

Based on motif distribution within each CTR, the domain was divided in two regions: proximal and distal. In the distal region, motifs had up to three Trp occupying positions with high frequency (motifs 1, 3, 5, 9, 14, 19), particularly those corresponding to the last residues of the protein. In some motifs the same occurred with Phe (motifs 1, 3, 5, 7, 10, 14 and 20) and Pro (motifs 1, 3, 5, 7 and 9). Another characteristic of the motifs in the distal region was the high abundance of acidic amino acids. These characteristics strongly resemble those of AHA activator motifs (Aromatic, large Hydrophobic, Acidic context) of the type found in HSFs proteins [46,56] and other transcriptional activators [57]. Mutational analysis in AHA motifs from HSFs has proven that importance of aromatic and large hydrophobic residues in the core positions of this motif, which for these proteins were generally Trp and Phe. The role of AHA motifs in the interaction with proteins from the basal transcription machinery (i.e., SWI/SNF, TFIID and SAGA complexes) has also been demonstrated [46,56]. The presence of these motifs in most HD-Zip I proteins constitutes an important finding for different reasons. Firstly, it provides solid evidence that most TFs from this subfamily act as transcriptional activators, as has been demonstrated experimentally for some proteins previously [14,34,58]. Secondly, it allows the location of the specific region within the CTR, the distal region, which is acting as an activation domain. Good examples of the importance of this regions are VRS1 [36], which lacks a motif in the CTR in relation to HvHox2, and the protein without the last 14 residues of the CTR that mimics the *tl *mutation [38]; in both cases the deletions correspond to AHA-like sequences. Finally, the different versions of AHA motifs found, shared by the members of each group, may be responsible for the interaction with different co-activators or members of the basal transcriptional machinery [37], and thus provide a source of functional divergence among HD-Zip I proteins together with differential expression patterns in some cases.

In the proximal region of the CTR, many motifs were characterized by the presence of Ser and acidic amino acids occupying most of the high-frequency positions. Many of the Ser in these motifs (i.e., motifs 2, 4, 6, 7, 10, 12, 16, 17 and 18) were predicted as putative phosphorylation sites. Particularly interesting was the case of the widespread motif 2 in proteins from groups III, IV, V and *Pp*, always in the proximal region. This may be important as it has been previously demonstrated *in vitro *[58] that the phosphorylation of ATHB6 with the PKA kinase inhibits its DNA-binding activity.

Putative sumoylation sites were also investigated in the CTR. The peptide to which SUMO is conjugated, ΨKXE/D, was mainly present in motifs 6, 8, 10 and 12 which were identified in groups Ic, II, V and *Pp*. A more exhaustive exploration revealed that 95 of the 178 CTRs had this motif, which appeared 143 times. The acidic amino acid was in most cases Glu (84%) and the hidrophobic residue, Val (62%).

The NTR presented 12 motifs and the correlation with putative phosphorylation sites was also analyzed. Some potential residues for this post-translational modification were found in motifs 1 (mostly from group III), 3 and 6. In relation to the motif 10 specific to group VI, six of its 11 members were predicted to have a NLS within this motif characterized by a patch of basic residues. This result may be extended to the other members of the group as they share the same motif. Sumoylation was also analyzed in the NTR but only eight sites were found, too few to consider it a significant general modification in HD-Zip I NTRs.

Proteins in the *Pp *group shared motifs in the NTR and CTR which supports the hypothesis that there has been little diversification of HD-Zip I TFs in this species. This is probably related to the simpler tissue organisation of mosses. There may be also a high tendency to heterodimerize and high functional redundancy among members. Further analysis would be necessary to address these observations.

The functionality of the CTR was also tested experimentally. ATHB1 was selected as a characteristic member of group III showing a typical AHA motif. It was previously demonstrated that this TF presents transactivation activity in plant protoplasts [59]. A yeast one-hybrid experiment was performed with the whole protein, the CTR alone, and a truncated version in which the CTR was removed. As predicted, the removal of the CTR containing the AHA motif generated a mutant protein which lost the ability to transactivate in this system, while the CTR alone was capable of transactivating when fused to GAL4-BD (Figure 6).

In our laboratory, two sunflower HD-Zip I TFs have been extensively studied, HAHB1 and HAHB4 [[26-29], JV Cabello, AL Arce, and RL Chan, unpublished results]. Both bind *in vitro *the pseudopalindromic sequence CAAT(A/T)ATTG, and both have been expressed in Arabidopsis under the control of the 35S CaMV promoter. The resulting plants exhibited clearly different phenotypes. Phylogenetically, HAHB1 is a member of group V whereas HAHB4 is in group I, but not included in any subgroup. HAHB1 NTR and CTR have most of the motifs present in other members of its group, but no particular motifs were detected in HAHB4 NTR or CTR, nevertheless, it has two Trp at the C-terminus, but with an abnormal basic residue close to them. In addition to the constructs of HAHB1 and HAHB4 complete coding sequences, three constructs were prepared and Arabidopsis transgenic plants transformed: a version of HAHB1 without the CTR (H1WCT), an analogous protein where the CTR of HAHB4 was deleted (H4WCT), and a chimerical protein bearing the NTR, HD and HALZ of HAHB4 fused to the CTR of HAHB1 (H4-H1). The promoter was also the 35S CaMV. The phenotypic differences between the plants were assessed in a triple response experiment in which hook formation in plants grown in ACC was measured as an indicator of ethylene sensitivity. The results confirmed previous observations: HAHB4 plants were highly insensitive and HAHB1 plants had a normal response. Among the mutant proteins, H4WCT displayed the higher insensitivity while H1WCT and H4-H1 showed an intermediate response. The deletion of HAHB1 CTR in H1WCT was enough to confer an intermediate insensibility to the transgenic plants, in contrast with the responsiveness displayed by HAHB1 plants. In the case of H4WCT, the insensitivity is not completely reverted by the deletion of the CTR. H4-H1 plants showed some impairment in ethylene response, which puts in evidence that the exchange in CTRs has an impact on the phenotype, at least for HAHB4 CTR which has no motifs in common. Despite these observations, the fact that both mutant proteins lacking the CTR (i.e., H1WCT and H4WCT) exhibit an altered sensitivity to ethylene may suggest that HAHB4 CTR could be non-essential for its activity in this pathway. Moreover, the sole removal of the CTR of HAHB1 produces a protein that does not develop the same response as HAHB1 in transgenic plants, stressing the importance of the CTR in this protein.

When leaf serration was evaluated it was shown that the fusion of HAHB1 CTR with HAHB4 HD-Zip was capable of generating the increase serration phenotype in one of the transgenic lines, as was observed for HAHB1 plants. The results obtained with the construction H4-H1 were not conclusive; a possibility is that both domains, the NTR and the CTR, may be important to generate a protein functionally similar to HAHB1.

Wenkel *et al. *[60] demonstrated that small leucine zipper-containing proteins were responsible for the inhibition of HD-Zip III proteins by heterodimerization. It is tempting to hypothesise that the capability of H1WCT of mimicking HAHB4 and H4WCT insensitivity to ethylene is the product of an inhibitory mechanism important in this pathway, especially considering that HAHB4 has an atypical AHA motif with a basic amino acid. In this scenario, the native HAHB4 protein would be more efficient than the mutant proteins H4WCT and H1WCT in exerting this inhibitory activity.

## Conclusions

**Figure 9:**
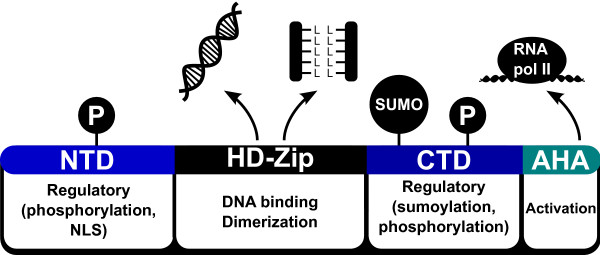
**Proposed model of functional domains in HD-Zip I transcription factors**. The *in silico *analysis conducted on a considerably large dataset of HD-Zip I transcription factors allowed us to postulate a generalized functional model of this family of proteins. This is supported to some extent by previous studies and the experimental results presented in this work. The well characterized HD-Zip domains are in charge of DNA binding and dimerization, an AHA motif in the CTR is responsible for activation, and the NTR and CTR are regions potentially phosphorylated and sumoylated, depending on the group, thus playing a regulatory role.

The analyses of a set of 178 HD-Zip I proteins allowed the identification of six groups, in most cases with high sequence conservation outside the HD and HALZ. An exhaustive exploration of these regions revealed an AHA motif in the CTR of most proteins that could be performing the activation role at a molecular level, like in HSFs TFs; and possibly giving some specificity to the interactions with the basal transcription machinery. Putative phosphorylation sites were found in the NTRs and CTRs, potential sumoylation motifs were discovered in the CTR, and NLSs found in the NTR for the members of one group. Altogether, this data allows us to postulate an enriched model of HD-Zip I functional domains or regions (Figure 9).

The presence of shared motifs in nearly all HD-Zip I proteins of the moss *Physcomitrella patens*, a much simple organism than higher plants, points to an ancestral low functional diversification of these proteins, beyond the numerous genes present in its genome. In more complex plants, like monocots and dicots, a discrete and probably incomplete number of groups has been identified. At a functional and evolutionary level, the potential significance of this division in groups represents an interesting topic for further research.

## Methods

### Database searches and sequence retrieval

The amino acid sequences of subfamily I HD-Zip members from different plant species were retrieved from NCBI's Conserved Domain Architecture Retrieval Tool (CDART, [61]. Members whose genome has been sequenced, i.e. *Arabidopsis thaliana*, *Populus trichocarpa*, *Oryza sativa*, *Zea mays*, *Physcomitrella patens *and *Selaginella moellendorffii *were removed and replaced with the sequences from these species obtained by Mukherjee et al. [11]. An examination of these sequences revealed that the tripeptide "YES" had been accidentally erased from the sequences published in this work. This was confirmed by the authors (Bürglin, TR; personal communication) and the sequences were corrected. Sequence redundance was checked using the "skipredundant" program of the EMBOSS package [62] and the results were manually inspected and curated. Sequences with truncated C-termini not belonging to the species mentioned were removed. Finally, three sequences from sunflower and *Medicago truncatula *(i.e., MTHB1, HAHB1 and HAHB11) were manually added. The final dataset is in Additional file 1.

### Data alignments and phylogenetic analysis

The 178 sequences were aligned using MAFFT v6 with the L-INS-i algorithm [41]. The result was manually inspected and edited using the Seaview4 multiple sequence editor [63]. Prior to phylogenetic analyses, the ProtTest program [64] was used to select the best-fit model of protein evolution, resulting in the JTT + I + G model [65-67]. Phylogenetic reconstruction was performed with this model plus the maximum-likelihood algorithm using the PhyML 3.0 program [42] with the NNI tree topology search operation and a total of 144 bootstrapped datasets. The phylogenetic tree shown in Figure 1 and Additional files 2 and 3 was obtained with the "consense" program from the PHYLIP package [68] and the images generated with FigTree (http://tree.bio.ed.ac.uk/software/figtree/).

The HD-Zip domain, NTRs and CTRs of the proteins were obtained recognizing first the HD and LZ domains with HMMer v2.3.2 [39] using the corresponding PFAM HMM models (PF00046.21 and PF02183.10, [40]). Then, the reports were parsed and the sequences processed with the aid of Perl scripts in which different modules from the Bioperl toolkit [69] were used. In this way three datasets were constructed, one for each portion of the protein: NTRs, HD-Zip domains and CTRs. With the sequences of the HD-Zip domains a phylogenetic reconstruction was performed as previously described for the tree with the complete sequences; the bootstrapped datasets were 100 (Additional file 2). Analogously, a third tree was constructed including in the alignment the HD-Zip domain of 3 HD-Zip II proteins: HAT1, HAT22 and ATHB17; as outgoup (Additional file 3). Their relative position in the tree was used to root the three trees mentioned.

The logos shown in Figure 2 were produced with WebLogo [70].

### Motif discovery and functional predictions

The program MEME [45] was employed on the NTR and CTR datasets. Some parameters were manually adjusted as follows: the motif limit to 20, the minimum motif width to 6, the minimum number of sites for each motif to 6, and the e-value threshold to 0.1. The plots of motif distribution (Figure 4 and Additional file 8) were prepared with the genoPlotR package [71] for the R statistical language [72].

The prediction of potential phosphorylation sites was performed on the NTRs and CTRs sequences of the 178 HD-Zip I proteins. For this purpose the stand-alone version of NetPhos 3.1 [48] was used. The selected cutoff score was 0.9. To better visualize of the results, in Additional files 7 and 10 each protein is represented by three stacked sequences: i) only the residues predicted to be phosphorylated are visible, the remaining are substituted with dots; ii) the visible residues correspond to motifs found by the MEME program; and iii) the complete sequence is visible. For the prediction of NLSs the program NLStradamus [50] was employed on the complete sequences of the 178 proteins with a subsequent threshold of 0.6. The Additional file 11 is the output report.

### Constructs

*35SCaMV:HAHB4 *construct was previously performed [29].

*35SCaMV:HAHB1*: The *HAHB1 *cDNA was isolated from a library constructed in lambda gt10 as previously described [30]. This fragment was cloned in the *EcoR*I site of the pMTL22 vector and restricted with *BamH*I/*Sac*I in order to clone it in the pBI 121 plasmid previously treated with the same enzymes. In this way, *HAHB1 *expression is controlled by the 35S CaMV promoter (JV Cabello, AL Arce, and RL Chan, unpublished results).

*35SCaMV:H4WCT: *the *HAHB4 *cDNA without its CTR was obtained by PCR amplification on the *35SCaMV:HAHB4 *clone with oligonucleotides Transfl and H4-WCT-R (see Table 3) and directly cloned in pBI121 by previously restricted with *BamH*I/*Sac*I.

**Table 3:** Oligonucletotides used for cloning

Oligonucleotide name	Sequence	Restriction sites	construct
Transfl	5'-gCggATCCACCATgTCTTTCAACAAgTA-3'	BamHI	H4-H1 cloning

H1CDS-R	5'-ggggAgCTCTCAATTgAATTgTggTTgTTCC-3'	SacI	H4-H1 cloning

H1*H4F	5'-**gTTggAggTg**TAAAAAATAgggAgCCAgC-3'		H4-H1 cloning

H1*H4R	5'-**TATTTTTTTAg**CACCTCCAACTgATTgAgTAgg-3'		H4-H1 cloning

H4-WCT-R	5'-CCCgAgTCTCTATTCTTCACCgCTgCCAC-3'	SacI	H4WCT cloning

H1-WCT-R	5'-ggCgAgCTCTCATCCTTCTgTTTCTTTTATgTTgAgg-3'	SacI	H1WCT cloning

H1atF	5'-gggggATCCgCTgATgACTTgCACTggAATggC-3'	BamHI	H1WCT cloning

H1qR	5'-CCAACCATggCCAAAACCCTg-3'		H1 qRT-PCR

H1qF	5'-ggCCggCAgATCATCAACTTC-3'		H1 qRT-PCR

H4qR	5'-gCCgAgTCTTAgAACTCCAACCACTTTTg-3'		H4 qRT-PCR

H4qF	5'-CgCgATCAAAgTCgAggCAgATTg-3'		H4 qRT-PCR

H4quimqF	5'-CgCgATCAAAgTCgAggCAgATTg-3'		H4-H1 qRT-PCR

H1AsTH4R	5'-TCCTTCTgTTTCTTTTATgTTgAgg-3'		H4-H1 qRT-PCR

UBCQPCR-R	5'-CAgTggACTCgTACTTgTTCTTgT-3'		Genomic DNA

UBC9GENOM-F	5'-gTTTTggAAATgTTgACAggAC-3'		Genomic DNA

ATHB1F	5' gCggAATTCATggAATCCAATTCgTTTTTC 3'	EcoRI	ATHB1 and ATHB1WCT cloning

ATHB1R	5' gCgggATCCTAAggCCATCCCCAgAAAg 3'	BamH1	ATHB1 cloning

ATHB1WCTR	5' gCggTCgACTACTCTTgTTTgCCCTgAAgC 3'	Sal1	ATHB1WCT cloning

ATHB1CTF	5' gCggAATTCCAAgAgACAgCTAATgAACCA 3'	EcoRI	ATHB1 CTR cloning

Sequence of oligonucleotides used in PCR reactions to perform constructs or cDNA quantifying. In bold, the overlapping sequences for chimerical constructs and underlined the restriction sites used for cloning.

*35SCaMV:H1WCT*: the *HAHB1 *cDNA without its CTR was obtained by PCR amplification on the *35SCaMV:HAHB1 *clone with oligonucleotides H1-WCT-R and H1atF (see Table 3) and directly cloned in pBI121 previously restricted with *BamH*I/*Sac*I.

*35SCaMV:H4-H1: *to obtain the chimerical construct, two separate amplifications were performed generating overlapping DNA segments as described by Higuchi et al. [73]. The 5' region of *HAHB4 *comprising the HD-Zip encoding domain was amplified with oligonucleotides Transfl and H1*H4R from the plasmid containing *35S:HAHB4 *in the pBI vector and the *HAHB1 *3' region encoding the carboxy terminus was amplified using *35SCaMV:HAHB1 *in pBI121 and the oligonucleotides H1*H4F and H1CDS-R (Table 3). Both products were electrophoretically purified and after a cycle of denaturation and hybridization, the hybrid was extended by Klenow and amplified by PCR using oligonucleotides Transfl and H1CDS-R. The final PCR product was purified from agarose and cloned into the pCR R2.1-TOPO vector (Invitrogen). Finally, the mutated cDNA was restricted with *BamH*I/*Sac*I and cloned in the pBI121 vector previously restricted with the same enzymes.

### Isolation and cloning of *ATHB1 *cDNA

Arabidopsis plants (Col 0) were germinated and grown in MS-agar medium during 14 days and after that placed in liquid MS (5 ml; 35 mm diameter vessels) supplemented with 300 mM mannitol during additional 24 h. After that, RNA was isolated by the Trizol method (Invitrogen, Carlsbad, CA, USA), following the manufacturer instructions, as previously described [26] (RT reactions were performed with 1 μg RNA and 200 units of M-MLV (Promega). PCR on cDNA was performed using oligonucleotides ATHB1F and ATHB1R for ATHB1, ATHB1F and ATHB1WCTR for ATHB1WCT, and ATH1CTF and ATHB1R for ATHB1CT (Table 3), and the PCR products cloned into the *Eco*RI/*Bam *HI or the *Eco*RI/*Sal *I sites of the pGBKT7 vector, respectively.

### Yeast culture and transformation

*Saccharomyces cerevisiae *AH109 (Clontech) cells were grown in YPDA or synthetic minimal medium (SD) supplemented with an amino acids dropout solution deficient in Trp or His [74]. Yeast cells were transformed with the previously obtained GAL4-ATHB1, GAL4-ATHB1WCT and GAL4-ATHB1CT chimerical constructs in pGBKT7 following the lithium acetate method [75]. Transformed cells were selected for tryptophan prototrophy on SD medium.

Transcriptional activation ability was assayed by β-galactosidase colony lift filter as suggested by the manufacturer (Clontech).

### Plant material and growth conditions

*Arabidopsis thaliana *Heyhn. Ecotype Columbia (Col-0) was purchased from Lehle Seeds (tucson, AZ). Plants were grown directly on soil in a growth chamber at 22-24 °C under long-day photoperiods (16 h of illumination with a mixture of cool-white and GroLux fluorescent lamps) at intensity of approximately 150 μE m^-2 ^s^-1 ^in 8 cm diameter × 7 cm height pots.

### Plant transformation

Transformed *Agrobacterium tumefaciens *strain LBA4404 was used to obtain transgenic *Arabidopsis *plants by the floral dip procedure [76]. Transformed plants were selected on the basis of kanamycin resistance and positive PCR which was carried out on genomic DNA with specific oligonucleotides for each construct as indicated in Table 3. Fifteen positive independent lines for each construction were used to select homozygous t3 and t4 plants in order to analyze phenotypes. Plants transformed with pBI101.3 were used as negative controls.

### Real time RT-PCR measurements

Expression levels of each transcript in transgenic plants were quantified by qPCR as follows. RNA was prepared with Trizol^® ^reagent (Invitrogen™) according to the manufacturer's instructions. RNA (2 μg) was used for the RT reactions using M-MLV reverse transcriptase (Promega). Quantitative PCRs were carried out using a MJ-Chromo 4 apparatus in a 20 μl final volume containing 1 μl SyBr green (10 ×), 8 pmol of each primer, 2 mM Mgcl_2_, 10 μl of a 1/25 dilution of the Rt reaction and 0,12 μl Platinum Taq (Invitrogen Inc.). Fluorescence was measured at 78-80°C during 40 cycles. Specific oligonucleotides for each gene were designed and their sequences specified in Table 3.

### Ethylene treatments

Seeds were surface-sterilized and plated with MS medium-0,8% agar in Petri dishes. After 2 days of incubation at 4°CC, dishes were placed in a growth chamber at 22-24°C. Dark grown seedlings were grown on 5 μM ACC, and maintained during the same period of time.

### Quantification of leaf serration

Leaves from 21-day-old plants were excised and images were acquired with a regular flatbed image scanner. These images were processed with the program LAMINA [77] which is designed to recognize different shape parameters of leaves, including serration. The number of serrations per leaf was calculated in WT, HAHB1 B, HAHB4 B and H4-H1 A, B and C plants. The results (Figure 8) showed that HAHB1 and H4-H1 B plants presented a clear increase in serration while the rest of the lines had a serration similar to WT plants. These results were subjected to the Kruskal-Wallis one-way analysis of variance by ranks and then the different lines were classified in groups according to pairwise comparisons with a p-value of 0,05 (Table 2).

## Abbreviations

ABA: abscicic acid; ACC: 1-aminocyclopropane-1-carboxylic-acid; AHA: Aromatic, large Hydrophobic, Acidic context; CST: complete sequence tree; CTR: carboxy-terminal region; ET: ethylene; HALZ: homeodomain-associated leucine zipper; HD-Zip: homeodomain-leucine zipper; HSF: Heat Stress Transcription Factors; JA: jasmonic acid; NTR: amino-terminal region; SAGA: Spt-Ada-Gcm5-Acetyltrasnferase; SWI/SNF: SWItch/Sucrose Non Fermentable; TF: transcription factor; TFIID: transcription factor IID; TL: Tendril-less

## Authors' contributions

ALA carried out the phylogenetic analysis, the functional characterization of CTRs and NTRs, analyzed the data and did the illustrations. JR performed the chimerical constructs, obtained the transgenic plants and did the triple response experiments. MC performed the cloning of ATHB1 in its three versions and did the yeast transactivation assays. JC together with ALA and JR analyzed the phenotype of the transgenic plants. RLC conceived this study, participated in the design and coordination and together with ALA drafted the MS. All authors read and approved the final manuscript.

## Supplementary Material

Additional file 1 **Sequences used in the analysis**. This spreadsheet file contains the sequences of the proteins used in this work, plus additional information. Click here for file

Additional file 2 **Sequence alignment of the HD-Zip domains**. The HD-Zip domains of the 178 proteins plus the three outgroups were processed for alignment. This alignment was used for the HZT (only the 178 HD-Zip I proteins) and the HZT + OG. Click here for file

Additional file 3 **Sequence alignment of the complete proteins**. The complete sequences of the 178 proteins were aligned for the construction of the CST. Click here for file

Additional file 4 **HZT and CST**. The phylogenetic trees are shown with their complete topology. Click here for file

Additional file 5 **HZT + OG**. Phylogenetic tree used to root the HZT and CST. Click here for file

Additional file 6 **Alignment of the CTRs**. The CTRs of the proteins of each group were aligned showing the conservation in this region. Click here for file

Additional file 7 **CTR putative phosphorylation sites**. Each of the CTRs is represented by three stacked sequences: i) only the residues predicted to be phosphorylated are visible, the remaining are substituted with dots; ii) the visible residues correspond to motifs found by the MEME program; and iii) the complete sequence is visible. Click here for file

Additional file 8 **Motif distribution in the NTRs**. In an analogous representation to the one in Figure 4, the distribution of motifs in the NTRs is depicted for each protein. The tree on the left represents their phylogenetic relationships. The analysis is divided in three separate plots and the groups identified previously (i.e., I-VI) are highlighted with boxes of dashed boundaries. Putative phosphorylation sites (Ser, Thr, Tyr) are marked with a black diamond, sumoylation motifs with a blue inverted triangle and NLSs with green crosses. Click here for file

Additional file 9 **Motifs found in the NTRs**. The sequence logos of the motifs found in the NTRs by the program MEME are displayed. Click here for file

Additional file 10 **NTRs putative phosphorylation sites**. Each of the NTRs is represented by three stacked sequences: i) only the residues predicted to be phosphorylated are visible, the remaining are substituted with dots; ii) the visible residues correspond to motifs found by the MEME program; and iii) the complete sequence is visible. Click here for file

Additional file 11 **NLStradamus report**. Text file with the report of the analysis performed with the program NLStradamus on the complete sequences. Click here for file
