# Supplementary material for: Uncharacterized conserved motifs outside the HD-Zip domain in HD-Zip subfamily I transcription factors; a potential source of functional diversity
Source: BMC Plant Biol. 2011 Mar 3;11:42. doi: 10.1186/1471-2229-11-42 (PMC3060862; doi:10.1186/1471-2229-11-42)
Supplement: Additional file 2 — Sequence alignment of the HD-Zip domains. The HD-Zip domains of the 178 proteins plus the three outgroups were processed for alignment. This alignment was used for the HZT (only the 178 HD-Zip I proteins) and the HZT + OG. [file 1471-2229-11-42-S2.PDF]

Alignment:
blocks=10

```
           1
At_AtHB23  --KKRRLNME QLKALEKDFE L---G----- ---NKLESDR KLELARALGL QPRQIAIWFQ NRRARSKTKQ
At_ATHB13  --KKRRLNME QVKTLEKNFE L---G----- ---NKLEPER KMQLARALGL QPRQIAIWFQ NRRARWKTKQ
Gm-ACU2089 --KKRRLNME QVKTLEKSFE L---G----- ---NKLEPER KMQLARALGL QPRQIAIWFQ NRRARWKTKQ
Gm-ACU2401 --KKRRLNME QVKTLEKSFE L---G----- ---NKLEPER KMQLARALGL QPRQIAIWFQ NRRARWKTKQ
Gm-ACU1869 --KKRRLNME QVKTLEKSFE L---G----- ---NKLEPER KMQLARALGL QPRQIAIWFQ NRRARWKTKQ
Mt-ACJ8462 --KKRRLNME QVKTLEKSFE L---G----- ---NKLEPER KMQLARALGL QPRQIAIWFQ NRRARWKTKQ
Gm-ACU2100 --KKRRLNME QVKTLEKSFE L---G----- ---NKLEPER KMQLARAFGL QPRQIAIWFQ NRRARWKTKQ
Rc-XP25201 --KKRRLNME QVKTLEKNFE L---G----- ---NKLEPER KMQLARALGL QPRQIAIWFQ NRRARWKTKQ
Vv-XP22768 --KKRRLNME QVKTLEKNFE L---G----- ---NKLEPER KMQLARALGL QPRQIAIWFQ NRRARWKTKQ
Cr-ABL6311 --KKRRLNME QVKTLEKNFE L---G----- ---NKLEPER KMQLARALGL QPRQIAIWFQ NRRARWKTKQ
Pt_687113  --KKRRLNME QVKTLEKNFE L---G----- ---NKLEPER KMQLARALGL QPRQIAIWFQ NRRARWKTKQ
Ha_HAHB1   --KKRRLNME QVKTLERNFE L---G----- ---NKLEPER KMQLARALGL QPRQIAIWFQ NRRARWKTKQ
Pt_696444  --KKRRLSME QVKTLEKNFE L---G----- ---NKLEPER KMQLARALGL QPRQIAIWFQ NRRARWKTKQ
Vv-CAO1494 --KKRRLNLE QVKTLEKNFE L---G----- ---NKLEPER KMQLARTLGL QPRQIAIWFQ NRRARWKTKQ
Sd-AAT4051 --KKRRLNME QVKTLEKNFE L---G----- ---NKLEPER KMQLARALGL QPRQIAIWFQ NRRARWKTKQ
Rc-XP25174 --KKKRLNLE QVKALEKSFE L---G----- ---NKLEPER KMQLAKALGL QPRQIAIWFQ NRRARWKTKQ
Pt_736296  --KKKRLNLE QVKALEKSFE L---G----- ---NKLEPER KMQLAKALGL QPRQIAIWFQ NRRARWKTKQ
Gh-ABO4774 --KKKRLNLE QVKALEKSFE L---G----- ---NKLEPER KVQLAKALGL QPRQIAIWFQ NRRARWKTKQ
Dc-05625.1 --KKRRLNME QVRTLEKSFE M---G----- ---NKLEPDR KLELARALGL QPRQIAIWFQ NRRARWKTKQ
Dc-05623.1 --KKRRLNIM QLKTLEKSFE L---G----- ---NKLEPER KLELSRALGL QPRQIAIWFQ NRRARWKTKQ
At_ATHB3   --KKKRLNLE QVRALEKSFE L---G----- ---NKLEPER KMQLAKALGL QPRQIAIWFQ NRRARWKTKQ
Sb-XP24672 --RKRRLSVE QVRTLERSFE V---A----- ---NKLEPER KAQLARALGL QPRQVAIWFQ NRRARWKTKQ
Zm_4124075 --RKRRLSVD QVRTLERSFE V---A----- ---NKLEPER KAQLARALGL QPRQVAIWFQ NRRARWKTKQ
Os_10g2650 --KKRRLSVE QVRTLERSFE S---G----- ---NKLEPER KAQLARALGL QPRQVAIWFQ NRRARWKTKQ
Sb-XP24657 --KKRRLNVE QVRTLEKNFE L---G----- ---NKLEPER KLQLARALGL QPRQVAIWFQ NRRARWKTKQ
Zm_4113431 --KKRRLNVE QVRTLEKNFE L---G----- ---NKLEPER KLQLARALGL QPRQVAIWFQ NRRARWKTKQ
Zm_413795  --KKRRLNVE QVRTLEKNFE L---G----- ---NKLEAER KLQLARALGL QPRQVAIWFQ NRRARWKTKQ
Os_03g0745 --KKRRLNVE QVRTLEKNFE L---G----- ---NKLEPER KMQLARALGL QPRQVAIWFQ NRRARWKTKQ
Ta-TaHZI-2 --KKRRLNVE QVRTLEKNFE V---A----- ---NKLEPER KMQLARALGL QPRQVAIWFQ NRRARWKTKQ
Zm_422699  --RKRRLSVE QVRTLERSFE V---A----- ---NKLEPER KAQLARALGL QPRQVAIWFQ NRRARWKTKQ
At_ATHB20  --KKKRLQLE QVKALEKSFE L---G----- ---NKLEPER KIQLAKALGM QPRQIAIWFQ NRRARWKTRQ
Gm-ACU1987 ---------- --KGLEKSFE L---G----- ---NKLEPER KMQLAKALGL QPRQISIWFQ DRRARWKTKQ
At_ATHB6   --KKRRLSIN QVKALEKNFE L---E----- ---NKLEPER KVKLAQELGL QPRQVAVWFQ NRRARWKTKQ
Brs-hb-6   --KKRRLSIN QVKALEKNFE L---E----- ---NKLEPER KVKLALELGL QPRQVAVWFQ NRRARWKTKQ
Bn-AAR0493 --KKRRLSIN QVKALEKNFE L---E----- ---NKLEPER KVKLAQELGL QPRQVAVWFQ NRRARWKTKQ
At_ATHB16  --KKRRLKVD QVKALEKNFE L---E----- ---NKLEPER KTKLAQELGL QPRQVAVWFQ NRRARWKTKQ
Vv-XP22660 --KKRRLSVD QVKALEKNFE V---E----- ---NKLEPER KVKLAQELGL QPRQVAVWFQ NRRARWKTKQ
Pt_HD56    --KKRRLSGD QVKALEKNFE V---E----- ---NKLEPER KVKLAQELGL QPRQVAVWFQ NRRARWKTKQ
Pt_70493   --KKRRLSGD QVKALEKNFE V---E----- ---NKLEPER KVKLAQELGL QPRQVAVWFQ NRRARWKTKQ
Gm-184277- --KKRRLSVD QVKALEKNFE V---E----- ---NKLEPDR KLKLAQELGL QPRQVAVWFQ NRRARWKTKQ
Pv-HDZ1    --KKRRLSVD QVKALEKNFE V---E----- ---NKLEPDR KVKLAQELGL QPRQVAVWFQ NRRARWKTKQ
Nt-Hfi22   --KKRRLSVE QVKALEKNFE V---E----- ---NKLEPER KVKLAQELGL QPRQVAVWFQ NRRARWKTKQ
Ze-18171.1 --KKRRLTAV QVKALEKNFE M---E----- ---NKLEPER KVKLAKELAL QPRQVAIWFQ NRRARWKTKQ
Sl-SlHDL1  --KKRRLSVE QVKALEKYFE V---E----- ---NKLEPER KVKLAQELGL QPRQVAVWFQ NRRARWKTKQ
At_ATHB5   --KKRRLGVE QVKALEKNFE I---D----- ---NKLEPER KVKLAQELGL QPRQVAIWFQ NRRARWKTKQ
Cp-CPHB-4  --KKRRLSNE QVKALEKYFE V---E----- ---NKLEPER KVKLAQELGL QPRQVAVWFQ NRRARSKTKN
Mt-ACJ8504 --KKCRLRVD QVKALEKNFE V---E----- ---NKLEPER KEKLAIELGL QPRQVAVWFQ NRRARWKTKQ
Vv-CAO6167 --KKKRLSFD QVKALERSFE I---E----- ---NKLEPER KVKIAEELGL KPRQVAIWFQ NRRARWKTKQ
Vv-XP22857 --KKKRLSFD QVKALERSFE I---E----- ---NKLEPER KVKIAEELGL KPRQVAIWFQ NRRARWKTKQ
Vv-CAN8361 --KKKRLSFD QVKALERSFE I---E----- ---NKLEPER KVKIAEELGL KPRQVAIWFQ NRRARWKTKQ
Rc-XP25139 --KKRRLSMD QVKALEKNFE V---E----- ---NKLEPER KIRLAEELGL QPRQVAIWFQ NRRARWKTKQ
Pt_HB      --KKRRLTLH QVHALEKNFE V---D----- ---NKLVPER KLKLAEELCL QPRQVAIWFQ NRRARWKTKQ
Pt_unknown --KKRRLNLH QVHALEKNFE V---E----- ---NKLEPER KLKLAGELGL QPRQVAIWFQ NRRARWKTKQ
Sb-XP24459 --KKRRLSTE QVRALERSFE T---E----- --NNKLEPER KARLARELGL QPRQVAVWFQ NRRARWKTKQ
Sb-XP24603 --KKRRLSAE QVRALERSFE V---E----- ---NKLEPER KARLARDLGL QPRQVAVWFQ NRRARWKTKQ
Zm_4112646 --KKRRLSSE QVRALERSFE V---E----- ---NKLEPER KARLARDLGL QPRQVAVWFQ NRRARWKTKQ
Os_09g2946 --KKRRLSVE QVRALERSFE V---E----- ---NKLEPER KARLARDLGL QPRQVAVWFQ NRRARWKTKQ
Zm_469358  --KKRRLSAE QVRALERSFE V---E----- ---NKLEPER KARLARDLGL QPRQVAVWFQ NRRARWKTKQ
Zm_469357  --KKRRLSAE QVRALERSFE V---E----- ---NKLEPER KARLARDLGL QPRQVAVWFQ NRRARWKTKQ
Os_08g3758 --KKRRLSVE QVRALERSFE T---E----- ---NKLEPER KARLARDLGL QPRQVAVWFQ NRRARWKTKQ
Ta-TaHZI-1 --KKRRLSAE QVRALERSFE V---E----- ---NKLEPER KARLARDLGL QPRQVAVWFQ NRRARWKTKQ
Os_10g2309 --KKRRLAAE QVRALERSFE A---D----- ---NKLDPER KARIARDLRL HPRQVAVWFQ NRRARWKTKQ
Zm_483405  --KKRRLAAD QVRALERSFE V---D----- ---NKLDPER KARIARDLSL HPRQVAVWFQ NRRARWKTKQ
Os_03g0896 --KKRRLALE QVRALERSFD T---D----- ---NKLDPDR KARIARDLGL QPRQVAVWFQ NRRARWKTKQ
Zm_4102187 --KKRRLALE QVRALERCFE T---D----- ---NKLDPDR KARIARDLAL QPRQVAVWFQ NRRARWKTKT
Zm_4118271 --KKRRLAPD QVRALERCFE A---D----- ---NRLDPDR KARVARDLAL NPRQVAVWFQ NRRARWKTKT
Sl-CAB6711 --KKRRLRVD QVQALEKIFE V---D----- ---NKLDPDR KVKIAQELGL QPRQIAIWFQ NRRARWKTKQ
At_ATHB1   --KKRRLTTE QVHLLEKSFE T---E----- ---NKLEPER KTQLAKKLGL QPRQVAVWFQ NRRARWKTKQ
Gm-ACU1808 --KKHRLSSE QVHLLEKSFE E---E----- ---NKLEPER KTQLAKKLGL QPRQVAVWFQ NRRARWKTKQ
Pv-HDZ3    --KKHRLSSE QVHLLEKSFE E---E----- ---NKLEPER KTQLAKKLGL QPRQVAVWFQ NRRARWKTKQ
Lj-BAG5005 --KKRRLTSE QVNLLEKSFE E---E----- ---NKLEPER KTQLAKKLGL QPRQVAVWFQ NRRARWKTKQ
Rc-XP25173 --KKRRLTSE QVNLLEKSFE E---E----- ---NKLEPER KTQLAKKLGM QPRQVAVWFQ NRRARWKTKQ
Vv-XP22788 --KKRRLTPE QVHLLEKSFE A---E----- ---NKLEPER KTQLAKKLGL QPRQVAVWFQ NRRARWKTKQ
Ps-ABR1622 --KKRRLSAD QVHFLEKSFE V---D----- ---NKLEPER KTQLARDLGL QPRQVAVWFQ NRRARWKTKQ
Os_08g3208 --KKRRLTAE QVQMLERSFE E---E----- ---NKLEPER KTELARRLGM APRQVAVWFQ NRRARWKTKQ
```

```
Zm_459694    --KKRRLTAE QVQLLERSFE E---E----- ---NKLEPER KTELARRLGM APRQVAVWFQ NRRARWKTKQ
Zm_459693    --KKRRLTAE QVQLLERSFE E---E----- ---NKLEPER KTELARRLGM APRQVAVWFQ NRRARWKTKQ
Os_02g4970   --KKRRLTPE QVHLLERSFE E---E----- ---NKLEPER KTELARKLGL QPRQVAVWFQ NRRARWKTKQ
Zm_4134425   --KKRRLTPE QVLLLERSFE E---E----- ---NKLEPER KTELARKLGL QPRQVAVWFQ NRRARWKTKQ
Gm-184278-   --KKRRLTSE QVQFLERNFE V---E----- ---NKLEPER KVQLAKELGL QPRQVAIWFQ NRRARFKTKQ
Pv-HDZ2      --KKRRLTSE QVQFLERNFE V---E----- ---NKLEPER KVQLAKELGL QPRQVAIWFQ NRRARFKTKQ
Rc-XP2520B   --KKRRLTAT QVQFLERNFE V---E----- ---NKLEPER KIQLAKELGL QPRQVAIWFQ NRRARFKNKQ
Pt_HAT5      --KKRRLTAA QVQFLEKSFE A---E----- ---NKLEPER KMQLAKELGL QPRQVAIWFQ NRRARFKNKQ
Cr-ABL631B   --KKRRLTAD QVQFLEKSFE V---E----- ---NKLEPER KVQLAKELGL QPRQVAIWFQ NRRARYKTKQ
Vv-CAO4102   --KKRRLTAG QVQFLERNFE V---E----- ---NKLEPER KNQLAKELGL QPRQVAIWFQ NRRARFKTKQ
Vv-CAO6250   --KKRRLTAD QVQFLERNFE V---E----- ---NKLEPER KVQLAKDLGL QPRQVAIWFQ NRRARWKTKQ
Vv-XP22716   --KKRRLTAD QVQFLERNFE V---E----- ---NKLEPER KVQLAKDLGL QPRQVAIWFQ NRRARWKTKQ
Vv-CAN8396   --KKRRLTAD QVQFLERNFE V---E----- ---NKLEPER KVQLAKDLGL QPRQVAIWFQ NRRARWKTKQ
Rc-XP25115   --KKRRLTVD QVQFLEKSFE V---E----- ---NKLEPER KLQLAKDLGL QPRQVAIWFQ NRRARWKTKQ
Pt_88244     --KKRRLTVD QVQFLERSFE V---E----- ---NKLEPER KIQLAKDLGL QPRQVAIWFQ NRRARWKTKQ
Dc-05624.1   --KKRRLKAD QIQFLEKSFE T---D----- ---NKLEPER KVQLAKELGL QPRQVAIWFQ NRRARWKTKT
Cp-CPHB-7    --KKRRLSSD QVRFLDKSFE V---D----- ---NKLEPER KVQLAKELGL QPRQVAIWFQ NRRARYKTKL
Gm-AAX9867   --KKRRLSVE QVKFLEKSFD E---E----- ---NKLEPER MIWLAKELGL QPRQVAIWFQ NRRARWKTKQ
Mt-ACJ8510   --KKRRLSVD QVQFLEKSFE E---D----- ---NKLEPER KTKLAKDLGL QPRQVAIWFQ NRRARWKTKQ
Cp-CPHB-6    -KKIRRLSAD QVRYLEKTFD Q---D----- ---NKLEPER KAKLAKDLGL QPRQVAIWFQ NRRARYKTKL
Sl-CAA6441   --KKRRLTDN QVQFLEKSFG E---E----- ---NKLEPER KVQLAKELGL QPRQIAIWFQ NRRARWKTKQ
Sm_18196     --KKRRLSVE QVKALEKNFE I---E----- ---NKLEPDR KIQLAKELGL QPRQVAVWFQ NRRARWKTKQ
Dc-05622.1   --KKRRLTVD QVKYLEKSFE V---E----- ---NKLEPDR KVQLAKDLGL QPRQVAIWFQ NRRARYKTKQ
Ps-ABK2476   --KKRRLTLE QVRALEKNFE I---A----- ---NKLEPEK KMQLAKALGL QPRQIAVWFQ NRRARWKTKQ
Pg-ABA5414   --KKRRLTLE QVRALEKNFE M---A----- ---NKLEPEK KMQLAKALGL QPRQIAVWFQ NRRARWKTKQ
Ps-ABK2449   --KKRRLTLE QVRALEKNFE I---A----- ---NKLEPEK KMQLAKALGL QPRQIAVWFQ NRRARWKTKQ
Pp_Pphb2     --KKRRLTFD QVRSLELNFE V---E----- ---NKLEPER KMQLAKELGL QPRQVAVWFQ NRRARWKTKQ
Pp_Pphb1     --KKRRLTFD QVRSHEKNFE I---E----- ---NKLEPER KMQLANELGL QPRQVAVWFQ NRRARWKTKQ
Pp_sca_31    --KKRRLTFD QVRSLERNFE V---E----- ---NKLEPER KMQLAKELGL QPRQVAVWFQ NRRARWKTKQ
Pp_sca_154   --KKRRLTFD QVRSLEKNFE I---E----- ---NKLEPER KLQLAQELGL QPRQVAVWFQ NRRARWKTKQ
Pp_sca_77    --KKRRLTFD QVRSLEYNFE I---E----- ---NKLEPER KMQLAKELGL QPRQVAVWFQ NRRARWKTKQ
Pp_sca_4     --KKRRLTFD QVRFLEKNFD I---E----- ---NKLEPER KLLLAKELGL RPRQVAVWFQ NRRARWRTKQ
Pp_sca_65a   --KKRRLSFD QVRSLERNFE V---E----- ---NKLEPER KMQLAKELGL QPRQVAVWFQ NRRARWKIKQ
Pp_sca_34    --KKRRLSFD QVRSLERNFE V---E----- ---NKLEPER KMQLAKELGL QPRQVAVWFQ NRRARWKIKQ
Pp_pphb6     --KKRRLTFD QVRSLERNFE M---E----- ---NKLEPER KMQLAKELGL RPRQVAVWFQ NRRARWKTKQ
Pp_Pphb8     --KKRRLSFE QVRSLERNFE M---E----- ---IKLEPDR KMKLAKELGL QPRQIAVWFQ NRRARWKTKQ
Pp_sca_28    --KKRRLTFD QVRSLERNFE M---E----- ---NKLEPER KMQLAKELGL QPRQVAVWFQ NRRARWKTKQ
Pp_sca_65b   --KKRRLSFD QVRSLERSFE V---E----- ---NKLEPER KMQLAKELGL QPRQVAVWFQ NRRARWKTKQ
Pp_sca_143   --KKRRLTLD QVRSLETSFE V---V----- ---NKLEPEK KMQLAKELGL RPRQVAVWFQ NRRARWKTKQ
Pp_sca_35    --KKRRLTLD QVRSLERNFE V---E----- ---NKLEPDR KMQLAKELGM RPRQVAVWFQ NRRARWKTKQ
Pp_Pphb7     --KKRRLSLE QVRSLERNFE V---E----- ---NKLEPER KMQLAKELGL QPRQVAVWFQ NRRARWKTKQ
Pp_Pphb9     --KKRRLSFD QVQSLERSFE L---E----- ---NKLEPER KLQLAKELGL QPRQVAVWFQ NRRARWKIKQ
Pp_Pphb5     --KKRRLTIE QVRSLEKNFE A---E----- ---NKLEPER KMRLAKELGL RPRQVAIWFQ NRRARWKTKQ
Sm_81609     --KKRRLSVD QVKSLERHFE Q---D----- ---NKLEPER KLQLAKELSL QPRQVAVWFQ NRRARWKTKQ
Os_09g2118   --RKRRLTAE QVRALERSFE E---E----- --KRKLEPER KSELARRLGI APRQVAVWFQ NRRARWKTKQ
Zm_4124691   RTTKRRLTAE QVRELELSFE E---E----- --KRKLEPER KSELARRLGI APRQVAVWFQ NRRARWRSKQ
Ps-ABK2462   --KKRRLTFQ QVKRLEKSFE V---A----- ---NKLEPER KIQLAKALGL QPRQIAVWFQ NRRARCKTKQ
Ps-ABK2572   --KKRRLSVQ QVRSLETSFE T---E----- ---SKLEPER KMQLAAELGL QPRQVAVWFQ NRRARWKTKQ
Sm_19476     --KKRRLSVD QVRSLELNFE M---E----- ---NKLEPER KKQLAHELGL QPRQVAVWFQ NRRARWKTKQ
Sm_18217     --KKRRLSVE QVRSLELSFE T---E----- ---NRLEPVR KMQLAQELGL QPRQVAVWFQ NRRARWKTKQ
At_ATHB22    --KKKMTSE QLKFLERSFQ E---EIKLNP DRKMKLNPDR KMKLSKELGL QPRQIAVWFQ NRKARWKNKQ
At_ATHB51    --KKKRLTSG QLASLERSFQ E---E----- ---IKLDSDR KVKLSRELGL QPRQIAVWFQ NRRARWKAKQ
Pt_93443     --KKKRLTSD QLESLEKSFQ E---E----- ---IKLDPDR KMKLSRELGL QPRQIAVWFQ NRRARWKAKQ
Vs-tendril   --KKNKLTSN QVDALERSFH E---E----- ---IKLDPER KMKLSAELGL QPRQVAVWFQ NRRTRWKTKQ
Cr-CRHB11    --KKRRLTAE QVNFLETSFS M---D----- ---LKLEPER KAHLAKQLGI QPRQVAIWFQ NRRARWKNQQ
Cr-CRHB6     --KKRRLTAE QVNFLETSFS M---D----- ---LKLEPER KAHLAKQLGI QPRQVAIWFQ NRRARWKNQQ
Cr-CRHB4     -EKKRGLTVD QVKYLEMNFT M---D----- ---FKLEPER KALIAKELGL RPRQVAIWFQ NRRARWKNKQ
At_ATHB7     KNNQRRFSDE QIKSLEMMFE S---E----- ---TRLEPRK KVQLARELGL QPRQVAIWFQ NKRARWKSKQ
At_ATHB12    --NQKRFSEE QIKSLELIFE S---E----- ---TRLEPRK KVQVARELGL QPRQVAIWFQ NKRARWKTKQ
Vv-XP22629   EKNKRRFSDE QIRLLESMFE S---E----- ---TKLEPRK KLQVAKELGL QPRQVAIWFQ NKRARWKSKQ
Vv-CAN7896   EKNKRRFSDE QIRLLESMFE S---E----- ---TKLEPRK KLQVAKELGL QPRQVAIWFQ NKRARWKSKQ
Rc-XP25299   -KNKRRFSDE QIKSLETMFE S---E----- ---TRLEPRK KLQLAKELGL QPRQVAIWFQ NKRARWKSKQ
Pt_HB7       -KNKRRFSDE QIKSLESMFE S---E----- ---TRLEPRK KMQLAKELGL QPRQVAIWFQ NKRARWKSKQ
Pt_731421    -KIKRRFSDE QIKSLETMFE S---E----- ---TRLEPRK KMQLARELGL QPRQVAIWFQ NKRARWKSKQ
Gm-ACU2443   --NTRRFSDE QIKSLETMFE S---E----- ---SRLEPRK KLQLARELGL QPRQVAIWFQ NKRARWKSKQ
Mt_MTHB1     --NTKRFTDE QIKSLETMFE T---E----- ---TRLEPRK KLQLARELGL QPRQVAIWFQ NKRARWKSKQ
Zm_hox6      --NKRRFTDD QIRLLESIFE S---E----- ---TKLEPKK KLQVARELGL QPRQVSIWFQ NRRARLKSKQ
Vv-XP22715   --NKKRFSDE QVQYLESIFE S---D----- ---SKLEARK KEELAVELGM QPRQVAIWFQ NKRARWKSKQ
Sb-XP24627   --NKKRFSEE QIKSLESMFA T---Q----- ---TKLEPRQ KLQLARELGL QPRQVAIWFQ NKRARWKSKQ
Zm_4496      --NKKRFSEE QIKSLESMFA T---Q----- ---TKLEPRQ KLQLARELGL QPRQVAIWFQ NKRARWKSKQ
Os_09g3591   --RKKRFSEE QIKSLESMFA T---Q----- ---TKLEPRQ KLQLARELGL QPRQVAIWFQ NKRARWKSKQ
Zm_4492      --NKKRFSDE QTKSLESMFA T---Q----- ---AKLEPRQ KLQLARELGL QPRQVAIWFQ NKRARWKSKQ
Pt_HBLZ      --NTRRFSDE QVRSLESMFE S---E----- ---TKLEPRK KMQLARELGL QPRQVAIWFQ NRRARWKTKQ
Ha_HAHB11    KHNNRRFSDE QIKSLESVFK R---E----- ---NKLEPRK KVEMARELGL HPRQVAIWFQ NRRARWKSKQ
Sb-XP24468   --RKRRFTEE QIRSLESMFH AH--H----- ---AKLEPRE KAELARELGL QPRQVAIWFQ NKRARWRSKQ
Zm_433493    --RKRRFTEE QIRSLESMFH AH--H----- ---AKLEPRE KAELARELGL QPRQVAIWFQ NKRARWRSKQ
Os_04g4581   --RKRRFTEE QIRSLESMFH AH--H----- ---AKLEPRE KAELARELGL QPRQVAIWFQ NKRARWRSKQ
Sb-XP24528   --RKRRFTEE QVRSLETTFH AR--R----- ---AKLEPRE KAELARELGL QPRQVAIWFQ NKRARWRSKQ
Os_02g4333   --RKRRFTEE QVRSLETTFH AR--R----- ---AKLEPRE KAELARELGL QPRQVAIWFQ NKRARWRSKQ
```

```
Zm_479999     --RKRRFTEE QVRSLETTFH AR--R----- ---AKLEPRE KAELARELGL QPRQVAIWFQ NKRARWRSKQ
Zm_470295     --RKRRFTEE QIRSLESTFR ARHGH----- ---AKLEPRE KAELARELGL QPRQVAIWFQ NKRARWRSKQ
Vv-XP22800    --RKRKFSEE QIRSLEFLFE S---E----- ---ARPEAQL KQKVASELGL HPRQVAIWFQ NKRARSKSKQ
Pt_548258     --YKRRFTDE QIKFLEFMFE S---E----- ---SRPESRV KQQLASELGL EPRQVAIWFQ NRRARLKTKQ
Pt_343725     --NKRRFSNE QIKFLETIFR S---E----- ---SRPELEI KQQLANEIGL EPRQVAIWFQ NRRARLKTKQ
Ha_HAHB4      --GRKRFTDK QISFLEYMFE T---Q----- ---SRPELRM KHQLAHKLGL HPRQVAIWFQ NKRARSKSRQ
At_ATHB21     --RKRKLSDE QVRMLEISFE D---D----- ---HKLESER KDRLASELGL DPRQVAVWFQ NRRARWKNKR
At_ATHB40     --RKRKLTDE QVNMLEMSFG D---E----- ---HKLESER KDRLAAELGL DPRQVAVWFQ NRRARWKNKR
Pt_655260     --RKRKLNEE QVNLLEMNFG N---E----- ---HKLESER KDKLASELGL DPRQVAVWFQ NRRARWKNKK
Pt_703426     --RKRKLSAE QVNFLEMNFG D---E----- ---HKLETER KDKLASDLGL DPRQVAVWFQ NRRARWKNKK
At_ATHB53     --RKRKLTDE QVNMLEYSFG N---E----- ---HKLESGR KEKIAGELGL DPRQVAVWFQ NRRARWKNKK
Os_03g1021    --KKRRLSDE QARFLEMSFK K---E----- ---RKLETPR KVQLAAELGL DAKQVAVWFQ NRRARHKSKL
Zm_480132     --RKRRLSDG QARFLELSFG K---E----- ---RKLETPR KVQLAAELGL DAKQVAVWFQ NRRARHKSKL
Zm_hox12      --RKRRLSDD QARFLELSFR K---E----- ---RKLETPR KVQLAAELGL DAKQVAVWFQ NRRARHKSKL
Os_07g3932    --KKRRLSDE QVEMLELSFR E---E----- ---RKLETGR KVHLASELGL DPKQVAVWFQ NRRARHKSKL
Zm_433132     --KKRRLSDE QAEMLELSFR E---E----- ---RKLETGR KVHLAAELGL DPKQVAVWFQ NRRARHKSKL
Zm_433210     --KKRRLTDE QVEMLELSFR E---E----- ---RKLETGR KVHLAAELGL DPKQVAVWFQ NRRARHKSKL
At_ATHB54     --KKRKLTPI QLRLLEESFE E---E----- ---KRLEPDR KLWLAEKLGL QPSQVAVWFQ NRRARYKTKQ
At_ATHB52     --KKKRLTQD QVRQLEKCFT M---N----- ---KKLEPDL KLQLSNQLGL PQRQVAVWFQ NKRARFKTQS
Gm-ACU198B    --NKKRLTED QVAILEKCFS S---N----- ---MKLEPEQ KFHLANQLGL PPRQVAIWYQ NKRARWKTQR
Pt_568845     --NKKRLTHD QVRLLERTFT T---T----- ---KKLEPEL KVQLANQLGV PPRQIAIWYQ NKRARWKTQS
Pt_98386      --NKKRLTED QVRLLERTFT T---N----- ---KKLEPEL KVQLANQLGV PPRQIAIWYQ NKRARWKTQS
Pt_594622     --RKKRLARD QLNLLETSFN A---N----- ---QKLKAEH KTELARQLGV PPKQVAIWYQ NRRARHKNDA
Pt_unknon     --RKKRLARD QLRLLETSFN A---N----- ---QTLKAEH KIELASQLGL TSRQVEIWYQ NRRARNKNNA
ATHB17        --KKLRLTRE QSRLLEDSFR Q---N----- ---HTLNPKQ KEVLAKHLML RPRQIEVWFQ NRRARSKLKQ
HAT1          --KKLRLSKD QSAVLEDTFK E---H----- ---NTLNPKQ KLALAKKLGL TARQVEVWFQ NRRARTKLKQ
HAT22         --KKLRLTKQ QSALLEDNFK L---H----- ---STLNPKQ KQALARQLNL RPRQVEVWFQ NRRARTKLKQ
```

```
             71
At_AtHB23    LEKDYDMLKR QFESLRDENE VLQTQNQKLQ AQ-VMALKSR EP---IE--- ----
At_ATHB13    LEKDYDTLKR QFDTLKAEND LLQTHNQKLQ AE-IMGLKNR EQ---TE--- ----
Gm-ACU2089   LEKDYDVLKR QYEAVKSDND ALQAQNQKLQ AE-ILALKSR EP---TE--- ----
Gm-ACU2401   LEKDYDVLKR QYEAVKSDND ALQAQNQKLQ AE-ILALKSR EP---TE--- ----
Gm-ACU1869   LEKDYDLLKR QYDAIKADND ALQAQNQKLQ TE-ILALKNR EP---TE--- ----
Mt-ACJ8462   LEKDYDVLKR QYDTIKADND ALQAQNQKLQ TE-ILALKNR EP---TE--- ----
Gm-ACU2100   LEKDYDLLKR QYEAIKADND ALQFQNQKLQ TE-ILALKSR EP---TE--- ----
Rc-XP25201   LEKDYDLLKR QFEAIKADND ALQAQNQKLH AE-IMALKSR EP---TE--- ----
Vv-XP22768   LEKDYDLLKR QFEAVKAEND ALQAQNQKLH AE-MLALKSR EP---TE--- ----
Cr-ABL6311   LEKDYDILKR QFEAIKAEND ALQAQNQKLH AE-IMALKNR EP---TE--- ----
Pt_687113    LEKDYDLLKR QFDAIKAEND ALQAQNQKLH AE-ILTLKSR EP---TE--- ----
Ha_HAHB1     LEKDYDALKR QFEAVKAEND SLQSQNHKLH AE-IMALKNR EP---AE--- ----
Pt_696444    LEIDYDLLKR QFDAVKAENN ALQTQNQRLH AE-ILALKSR EP---TE--- ----
Vv-CAO1494   LEKDYDLLKR QFEAVKADND ALQAQNKKLH AE-LLALKSR EP---KG--- ----
Sd-AAT4051   LEKDYEVLKR QFDAIKAEND ALQTQNQKLH AE-IMSLKNR EQ---PT--- ----
Rc-XP25174   LEKDYDVLKK QFDALKADNE VLQTQNKKLH AE-LMDLKGR DS---ND--- ----
Pt_736296    LEKDYEVLKK QFDALKADND ALQAQNKKLH AE-LLSLKSR ES---NE--- ----
Gh-ABO4774   LEKDYDALKK QFEALKADND ALQAQNKKLN AE-LLALKTK DS---NE--- ----
Dc-05625.1   LEKDYDLLKS QFDAVKAEND SLQSHNQKLH AQ-IMALKNG EP---TE--- ----
Dc-05623.1   LEKDYDLLKS QFDSLKAEND SLQSRNKALH AQ-ILALKNR EP---TD--- ----
At_ATHB3     LERDYDSLKK QFDVLKSDND SLLAHNKKLH AE-LVALKKH DR---KE--- ----
Sb-XP24672   LEKDYDALRR QLDAARAEND TLLSHNKKLQ AE-IMALKGG GG---GG--- ----
Zm_4124075   LEKDYDALRR QLDAARAEND ALLSHNKKLQ AE-IMALKGG GG---GG--- ----
Os_10g2650   LEKDFDALRR QLDAARAEND ALLSLNSKLH AE-IVALKGG AA---AA--- ----
Sb-XP24657   LEKDYDALKR QLDAVKADND ALLSHNKKLQ AE-ILALKGG RE---AG--- ----
Zm_4113431   LEKDYDALKR QLDAVKADND ALLSHNKKLQ AE-ILSLKGR EA---GG--- ----
Zm_413795    LEKDYDALRR QLDAVKADND ALLSHNKKLQ AE-ILXLKGR EA---AG--- ----
Os_03g0745   LEKDYDALKR QLDAVKAEND ALLNHNKKLQ AE-IVALKGR EA---AS--- ----
Ta-TaHZI-2   LEKDYDVLKR QFDAVKAEND ALLSHNKKLQ SE-ILGLKEC RE---AA--- ----
Zm_422699    LEKDYDALRR QLDAARAEND ALLSHNKKLQ TE-DLRLRPR PQ---LK--- ----
At_ATHB20    LERDYDSLKK QFESLKSDNA SLLAYNKKLL AE-VMALKNK EC---NE--- ----
Gm-ACU1987   LEKEYEVLKK LFEAVKADND SLKAQNQKLH AE-LQTLKSR DC---SE--- ----
At_ATHB6     LEKDYGVLKT QYDSLRHNFD SLRRDNESLL QE-ISKLKTK LN---GG--- ----
Brs-hb-6     LEKDYGVLKT QYDSLRHNFD SLRRDNESLL QE-IGKLKAK LN---GE--- ----
Bn-AAR0493   LEKDYGVLKT QYDSLRHNFD SLRRDNESLL QE-IGKLKAK LN---GE--- ----
At_ATHB16    LEKDYGVLKG QYDSLRHNFD SLRRDNDSLL QE-ISKIKAK VN---GE--- ----
Vv-XP22660   LERDYGILKA NYETLKLNYD AIQHDNEALL KE-IRELKSK LN---EE--- ----
Pt_HD56      LERDYGVLKA NYDSLKHNFD ALQHDNEALL KE-IRELKAK LN---EE--- ----
Pt_70493     LERDYGVLKA NYDSLKHNFD AIQQDNEALL KE-IRELKAK LN---EE--- ----
Gm-184277-   LERDYGVLKA NYESLKLNYD TLQQDHEALL KE-IKELKSR LV---QE--- ----
Pv-HDZ1      LERDYGVLKA NYDSLKLNYD TLQQDNEALL KE-IKELKSR LL---LQ--- ----
Nt-Hfi22     LERDYGVLKS NFDALKHNYE SLKHDNEALL KE-ILELKSK VY---TE--- ----
Ze-18171.1   LERDYGVLKS NFDSLKHKYE SLKQDNESMV KQ-IKELKSK LY---EE--- ----
Sl-SlHDL1    LERDYGVLKS NFDTLKLNYD SLLHDKDSLL NQ-IKMLKSK LN---EK--- ----
At_ATHB5     LERDYGVLKS NFDALKRNRD SLQRDNDSLL GQ-IKELKAK LN---VE--- ----
Cp-CPHB-4    LERDYGVLKS NYNALKHDFE TLKRDNESLL KE-IHELKSK LN---ED--- ----
Mt-ACJ8504   LERDYGVLKA NYDALKLKFD AIAQDNKAFH KE-IKELKSK LG---EE--- ----
Vv-CAO6167   LERDYGILKA NYDALKLDYE SLEQEKEALV AE-LRELKAK LQ---GG--- ----
Vv-XP22857   LERDYGILKA NYDALKLDYE SLEQEKEALV AE-LRELKAK LQ---GG--- ----
Vv-CAN8361   LERDYGILKA NYDALKLDYE SLEQEKEALV AE-LRELKAK LQ---GG--- ----
Rc-XP25139   LEREYVTLKT NYEALKLDYN NLERDNESLN LQ-LKELKAK MR---EG--- ----
Pt_HB        LERDYGTLKA NYEALNLDYS NLEQKNEALA QK-VKELKAK LR---EE--- ----
Pt_unknown   LERDYGTLKA NYEALKLDYC NLEQKNEVLA QK-VKELKAK LS---EE--- ----
Sb-XP24459   LERDYAALRH SYDALRADHD ELRRDKDALL DE-IKELKAK LG---DD--- ----
Sb-XP24603   LERDYAALRH SYDALRHDHD ALRRDKDALL AE-IKELKAK LG---DE--- ----
Zm_4112646   LERDYSALRQ SYDALRHDHD ALRRDKDALL AE-IKELKAK LG---DE--- ----
Os_09g2946   LERDYAALRH SYDSLRLDHD ALRRDKDALL AE-IKELKAK LG---DE--- ----
Zm_469358    LERDYAALRR SYDALRLDHD ALRRDKDALL AE-IRELKAK LG---DD--- ----
Zm_469357    LERDYAALRR SYDALRLDHD ALRRDKDALL AE-IRELKAK LG---DD--- ----
Os_08g3758   LERDYAALRQ SYDALRADHD ALRRDKDALL AE-IKELKGK LG---DE--- ----
Ta-TaHZI-1   LERDYNALRH SYDALRVDHD ALRRDKEALL AE-IKDLKGK LG---DE--- ----
Os_10g2309   IERDFAALRS RHDALRLECD ALRRDKDALA AE-IADLRDR VD---GQ--- ----
Zm_483405    IERDFAALRV RHDALRVECD ALRRDKDALA AE-IKELRGM VE---KQ--- ----
Os_03g0896   LERDFAALRA RHDALRADCD ALRRDKDALA AE-IRELREK LP---TK--- ----
Zm_4102187   LERDFAALRA RHDALRADCD ALRRDKDALA AE-IRELRQK LL---PK--- ----
Zm_4118271   LDRDFAALRA RHDALRADCD ALRRDKDALA AE-IRELRQK LL---PK--- ----
Sl-CAB6711   LERDYNILKS NYEALQHNYT KVEQEKEGLI TE-LKGLKEK LG---EE--- ----
At_ATHB1     LERDYDLLKS TYDQLLSNYD SIVMDNDKLR SE-VTSLTEK LQ---GK--- ----
Gm-ACU1808   LERDYDVLKS SYDTLLSSYD SIMKENEKLK SE-VVSLNEK LQ---VQ--- ----
Pv-HDZ3      LERDFDVLKS SYDTLLSSYD SLMKDNEKLK SE-VVSLNEK LQ---DQ--- ----
Lj-BAG5005   LERDYDVLKS SYDSLLATYD TIAKENEKLK SE-VVSLNEK LQ---VQ--- ----
Rc-XP25173   LERDYDVLKA SYDSLLSDFD NTVKENQKLK SE-VVSLTEK LQ---TK--- ----
Vv-XP22788   LERDYDLLKS SYDSLVSEYD SILKEKEKLK SE-VVSLTEK LQ---AK--- ----
Ps-ABR1622   LEREYDILKS SYDTLRVDYD NLLKEKEKLR SE-VICLTDK LH---AK--- ----
Os_08g3208   LEHDFDRLKA AYDALAADHH ALLSDNDRLR AQ-VISLTEK LQ---DK--- ----
Zm_459694    LETDYDRLKA AYDALAADHQ GLLADNDNLR AQ-VADSSPR FV---SS--- ----
Zm_459693    LETDYDRLKA AYNALAADHQ GLLADNDSLR AQ-------- ---------- ----
Os_02g4970   LERDFDRLKA SFDALRADHD ALLQDNHRLH SQ-VMSLTEK LQ---EK--- ----
```

```
Zm_4134425  LERDFDRLKA SFDALRADHD ALLQDNNRLR SQ-VVSLTEK LQ---EK--- ----
Gm-184278-  LEKDYGVLKA SYDRLKSDYE SLVQENDKLK AE-VNSLESK LI---LR--- ----
Pv-HDZ2     LEKDYGTLKA SYDRLKGDYE SLLQENDKLK AE-VNSLESK LI---LR--- ----
Rc-XP2520B  LEKDYDSLKA SYDKLKADYD NLLKENENLK NE-FVSLKDK LL---AR--- ----
Pt_HAT5     LERDYDSLRI SFDKLKADYD KLLLEKQNLK NE-LLSLKEK LL---SR--- ----
Cr-ABL631B  LEKEYDSLKS SFDKLNADYD SLFKENEKLK NE-VKLLTEK LL---MR--- ----
Vv-CAO4102  LEKDYDSLKA SYDSLKADYD CILKEKEKLK TE-LLLLTDK AL---IG--- ----
Vv-CAO6250  LEKDFGALQA SYNSLKAEYE NLLKEKDELK TE-VILLTDK LL---VK--- ----
Vv-XP22716  LEKDFGALQA SYNSLKAEYE NLLKEKDELK TE-VILLTDK LL---VK--- ----
Vv-CAN8396  LEKDFGALQA SYNSLKAEYE NLLKEKDELK TE-VILLTDK LL---VK--- ----
Rc-XP25115  MEKDYDVLQT SYNSLKADYD ALLQEKDRLK AE-VNLLTDK LL---LK--- ----
Pt_88244    LEKDYEVLQS SYNGLKADYD NLFKEKEKLK AE-VNLLTNE LL---LK--- ----
Dc-05624.1  LEKDYDVLQN SYNSLKADYD NLLAEKEKLK AE-VLDLTDK LL---LK--- ----
Cp-CPHB-7   LEKDYDALKS NYDRLKEDFD ALYSENEKLK TE-VNTLAEK LL---GK--- ----
Gm-AAX9867  MEKDYDSLQT SYNDLKANYD NLLREKDKLK AE-VARLTEK VL---GR--- ----
Mt-ACJ8510  LEKDYDSLND GYESLKTEYD NLLKEKDRLQ SE-VASLTEK VL---ER--- ----
Cp-CPHB-6   LQKDCDVLKS SYDRLKRDYD ALFSQNEKLK IE-IDSLMGK LQ---GK--- ----
Sl-CAA6441  LEKDYDELRN RYDTLKSNYN NLLKEKEDLR TE-VFRLTGK LF---IK--- ----
Sm_18196    LEKDYDLLKS EYDDLKASYV DLAKERDKLQ AE-V------ ---------- ----
Dc-05622.1  LEKDYDSLKE CYDKLRDDHD RLSKENEKLR LE-VILDYKS LQ---FC--- ----
Ps-ABK2476  LEKDFNVLKQ DYDALKQDYD NLMEENNNLQ AM-VGNYTLF LF---IL--- ----
Pg-ABA5414  LEKDFNVLKQ DYDALKQDYD NLMEENNNLQ AM-IERMSSK SQ---SC--- ----
Ps-ABK2449  LEKDFNVLKQ DYDALKQDYD NLMEENNNLQ AM-IERMSSK SQ---SC--- ----
Pp_Pphb2    LERDYEVLTL DYNRLKSEFE AVLQEKQELQ GE-HIVNFGQ ME-------- ----
Pp_Pphb1    LERDYEVLTL DYNRLKSEFE AVLQEKQELQ DE-MECLTEK IQ---TA--- ----
Pp_sca_31   LERDYEVLTS DYNRLKSEFE AVLQEKQELQ GE-IECLTGK LQ---IS--- ----
Pp_sca_154  LERDYEVLSL DYNQLKNKFD DVVQEKQQLQ EE-MDCLRGK LP---TP--- ----
Pp_sca_77   LERDYEVLNL DYNRLKKEFD AVIQEKQELQ DA-VKTLKEK SP---MP--- ----
Pp_sca_4    LERDYESLTS GYKQLKSEFE AMLQEKQDLQ GE-VERLTEK LQ---TV--- ----
Pp_sca_65a  LERDYETLTQ DYNRLKSDFE AVLKDKKNLK DE-PASPAQS EK---SD--- ----
Pp_sca_34   LECDYDALTQ DYNRLKNDFD AALRDKKKLK NE-VNRLKGI AP---EV--- ----
Pp_pphb6    LERDYEALAA DYKSLKHDYD LVLAEKNNLK AE-VQRLSGK AP---TS--- ----
Pp_Pphb8    LERDFELLNS GYSKLKRDFE KVLEEKDVLK AE-LVRLSAK II---PK--- ----
Pp_sca_28   LERDYEVLNS GYLKLKVEFE TALREKDFLK AE-VQRLSGK TS---SQ--- ----
Pp_sca_65b  LERDYEMLNS GYIKLKADFE TALREKDVLK AE-VQRLSGK TS---PQ--- ----
Pp_sca_143  LERDYETLAA DYKTLMADYE HVVEERNCLR AE-VVRLTGE TP---PS--- ----
Pp_sca_35   LERDYETLEA GFKRLKADYE LVLDEKNYLK AEQLQRLSGD T--------- ----
Pp_Pphb7    LERDYETLKK AYDRLKADFE AVTLDTSALK AE-VSRLKGI SN---DD--- ----
Pp_Pphb9    LERDYGALAK DYNRLKEEFE AVSRDRNGYK AE-VNLLKGI RN---DD--- ----
Pp_Pphb5    LERDYETLES DYKRLKADYE QVLSEKNHLK AE-FTCVDSN MF---AL--- ----
Sm_81609    LEKDYDALKE NLDALRGDYK SLLKEKQELE AE-VCQIFFL FV---LS--- ----
Os_09g2118  LELDFDRLRA AHDELLAGRT ALAADNESLR SQ-VILLTEK LQ---AN--- ----
Zm_4124691  LEQDFDRLRA AHDDLIAGRD ALLADNDRLR SQ-------- ---------- ----
Ps-ABK2462  VEKDFDALKQ QYDDLKNKYD ILLQENKHFK AE-RLNRESG ND---DQ--- ----
Ps-ABK2572  LERDYDDLKQ QYEEVVAEKK KLEGQVARLT QE-VVAAKGE KK---DQ--- ----
Sm_19476    LERDYESLKA SYDKLLLENK KLQAEVTALV AR-LDSSKSP TA---AA--- ----
Sm_18217    LEKDYDVLKA AYESLA---- ---EENKRLK AQ-LSDKKLV DGDHEKKPDE ----
At_ATHB22   LEHLYESLRQ EFDIVSREKE LLQEELIQLK SM-IRED--- ---------- ----
At_ATHB51   LEQLYDSLRQ EYDVVSREKQ MLHDEVKKLR AL-LRDQGLI KK---QI--- ----
Pt_93443    LERLYDNLKQ EFDSVSKEKQ KLQEEVMKLK AV-VREQATR KQ---VS--- ----
Vs-tendril  LEQDYDVLKQ E-------NQ KLQDEVMTLK EK-LKEK--- ---------- ----
Cr-CRHB11   IEQDYESLKA SYEAVVEEKE RLLKEHDLAL EA-NKRLQAE ---------- ----
Cr-CRHB6    IEQDYESLKA SYEAVVEEKE RLLKEHDLAL EA-NKRLQAE IA---RLTRS LQSY
Cr-CRHB4    LEQDYETLKS SYEALLQENE DMVKRNKALD EE-NKLLQAE IA---RLTGI SGNV
At_ATHB7    LETEYNILRQ NYDNLASQFE SLKKEKQALV SE-LQRLKEA TQ---KK--- ----
At_ATHB12   LEKEYNTLRA NYNNLASQFE IMKKEKQSLV SE-LQRLNEE MQ---RP--- ----
Vv-XP22629  LERDYSILRG NYNSLVSRFE SLKKEKQALV IQ-LQKLNEM VQ---QS--- ----
Vv-CAN7896  LERDYSILRG NYNSLVSRFE SLKKEKQALV IQ-LQKLNEM VQ---QS--- ----
Rc-XP25299  LERDYNILRA NYNSLASRFE SLKKEKQALA LQ-LQKLNNM ME---KS--- ----
Pt_HB7      LERDFSILRA NYNSLASRFE TLKKEKQALV IQ-LQKINDL MK---KP--- ----
Pt_731421   LERDYSMLRA NYNSLASRFE TLKKEKQALA IQ-LQKLNDL MK---KP--- ----
Gm-ACU2443  LERDYGILQS NYNSLASRFE ALKKENQTLL IQ-LQKLNHL MQ---KP--- ----
Mt_MTHB1    LEREYNKLQN SYNNLASKFE SMKKERQTLL IQ-LQKLNDL IQ---KP--- ----
Zm_hox6     MEKDYVTLRD NYDKLVSRFE MLKEEKQCLI SQ-KEKLSEM LA---ES--- ----
Vv-XP22715  IEHDYKALRA SYDALTSRFE SLKEEKQSLL TQ-LQKLGDL ME---KP--- ----
Sb-XP24627  LEREYSALRD DYDALLCSYE SLKKEKHALL KQ-LEKLAEM LH---EP--- ----
Zm_4496     LERDYSALRD DYDALLCSYE SLKKEKHTLL KQ-LEKLAEM LH---EP--- ----
Os_09g3591  LEREYSALRD DYDALLCSYE SLKKEKLALI KQ-LEKLAEM LQ---EP--- ----
Zm_4492     LEREYSALRD DYHALLCSYE SLKDEKRALL KQ-LEKLAEM LH---EP--- ----
Pt_HBLZ     MEQKYKTLKA SYDNLAS--- SLKNERESLL LQ-LQTVSNQ LG---NP--- ----
Ha_HAHB11   VEQDYYNLKA DYDTLAHRFE SLKKEKHALL HQ-VSQLKEL DS---GS--- ----
Sb-XP24468  LEHDYAVLRA KFDDLHARVE SLKQDKLALT TQ-LNELSER LR---ER--- ----
Zm_433493   LEHDYALLRA KFDDLHAHVE SLKQDKLALT TQ-LSELSER LR---ER--- ----
Os_04g4581  LEHDYAALRS KYDALHSRVE SLKQEKLALT VQ-LHELRER LR---ER--- ----
Sb-XP24528  LEHDYAALRA RYDALHARVD SLREEKLALA KQ-VDELRGR LQ---SV--- ----
Os_02g4333  IEHDYAALRA QYDALHARVE SLRQEKLALA DQ-VDELRGK LN---ER--- ----
Zm_479999   LEHDYAALRA QFDAMHARVE SLRQEKIALA AQ-VDELRGR LN---ER--- ----
Zm_470295   LEHDYAVLRA KFDDLHARVE SLRRDKLALS TQ-LFAWCAR VH---DQ--- ----
Vv-XP22800  IEQDYAVLKA SYDNLALQFE SLKEKNQNLA IQ-LQRLRDG LE---KP--- ----
```

```
Pt_548258    IEKEYSILKA SYDVLASSFE SLKREKQSLI IQ-LHKLKNR HV---KQ--- ----
Pt_343725    IEKEYSLLKA NYEALASRFE SLKRENQSLL IH-LQKLKNQ RV---KQ--- ----
Ha_HAHB4     IEQEYNALKH NYETLASKSE SLKKENQALL NQ-LEVLRNV AE---KH--- ----
At_ATHB21    VEDEYTKLKN AYETTVVEKC RLDSEVIHLK EQ-LYEAERE IQ---RL--- ----
At_ATHB40    LEEEYNKLKN SHDNVVVDKC RLESEVIQLK EQ-LYDA--- ---------- ----
Pt_655260    LEEEYTKLKT SHENIVVEKC QLESEVLKLK EQ-LSEA--- ---------- ----
Pt_703426    LEEEYTKLKT AHESIVVQKC QLESEVLKLK EQ-LSRTEKE IQ---RL--- ----
At_ATHB53    LEEEYAKLKN HHDNVVLGQC QLESQILKLT EQ-LSEA--- ---------- ----
Os_03g1021   MEEEFAKLRS AHDAVVLQNC HLETELLKLK ER-LADV--- ---------- ----
Zm_480132    MEEEFSRLRA AHDAAVLHNC HLETELLKVK DR-LAEA--- ---------- ----
Zm_hox12     MEEEFSKLRA AHDAVVLHNC HLETELLKMK DR-LAEV--- ---------- ----
Os_07g3932   LEEEFSKLKH AHDAAILHKC HLENEVLRLK ER-LVVA--- ---------- ----
Zm_433132    LEEEFAKLKQ AHDATILHKC HLENELMRVK DR-LVLA--- ---------- ----
Zm_433210    LEEEFAKLKQ AHDAAILHKC HLENEVMRLK DK-LVLA--- ---------- ----
At_ATHB54    LEHDCDSLKA SYAKLKTDWD ILFVQNQTLK SK-VQFLNRL TSHYFQE--- ----
At_ATHB52    LEVQHCTLQS KHEAALSDKA KLEHQVQFLQ DE-LKRARNQ LA---LF--- ----
Gm-ACU198B   LEVDYGVLQA RLENVVAEKK QLEKDVERLK AE-LKKAQEM LL---IT--- ----
Pt_568845    LELDYNSLHA KLEDALADRR RLEREVVQLQ EE-LRRAQQM VF---HL--- ----
Pt_98386     LELDYNTLQV RLENALADRR RLEREVVRLQ EE-LWQAQQM VF---AV--- ----
Pt_594622    IEHDYMNIQL ELGNVLAENI RLEKQVSMLK FE-LNKVQQM IL---FG--- ----
Pt_unknon    IEHDYKNVQL ELGNVMTENT RLEKQVSTLK YE-LNKVQQM IL---FG--- ----
ATHB17       TEMECEYLKR WFGSLTEENH RLHREVEELR AM-KVGPTTV NS---AS--- ----
HAT1         TEVDCEYLKR CVEKLTEENR RLEKEAAELR AL-KLSPRLY GQ---MS--- ----
HAT22        TEVDCEFLKK CCETLTDENR RLQKELQDLK AL-KLSQPFY MH---MP--- ----
```
