# Supplementary figures and images for: Uncharacterized conserved motifs outside the HD-Zip domain in HD-Zip subfamily I transcription factors; a potential source of functional diversity
Source: BMC Plant Biol. 2011 Mar 3;11:42. doi: 10.1186/1471-2229-11-42 (PMC3060862; doi:10.1186/1471-2229-11-42)

CST

H Z T

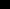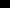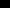

la

**lb**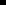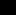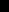

IV

*Pp*

V

VI

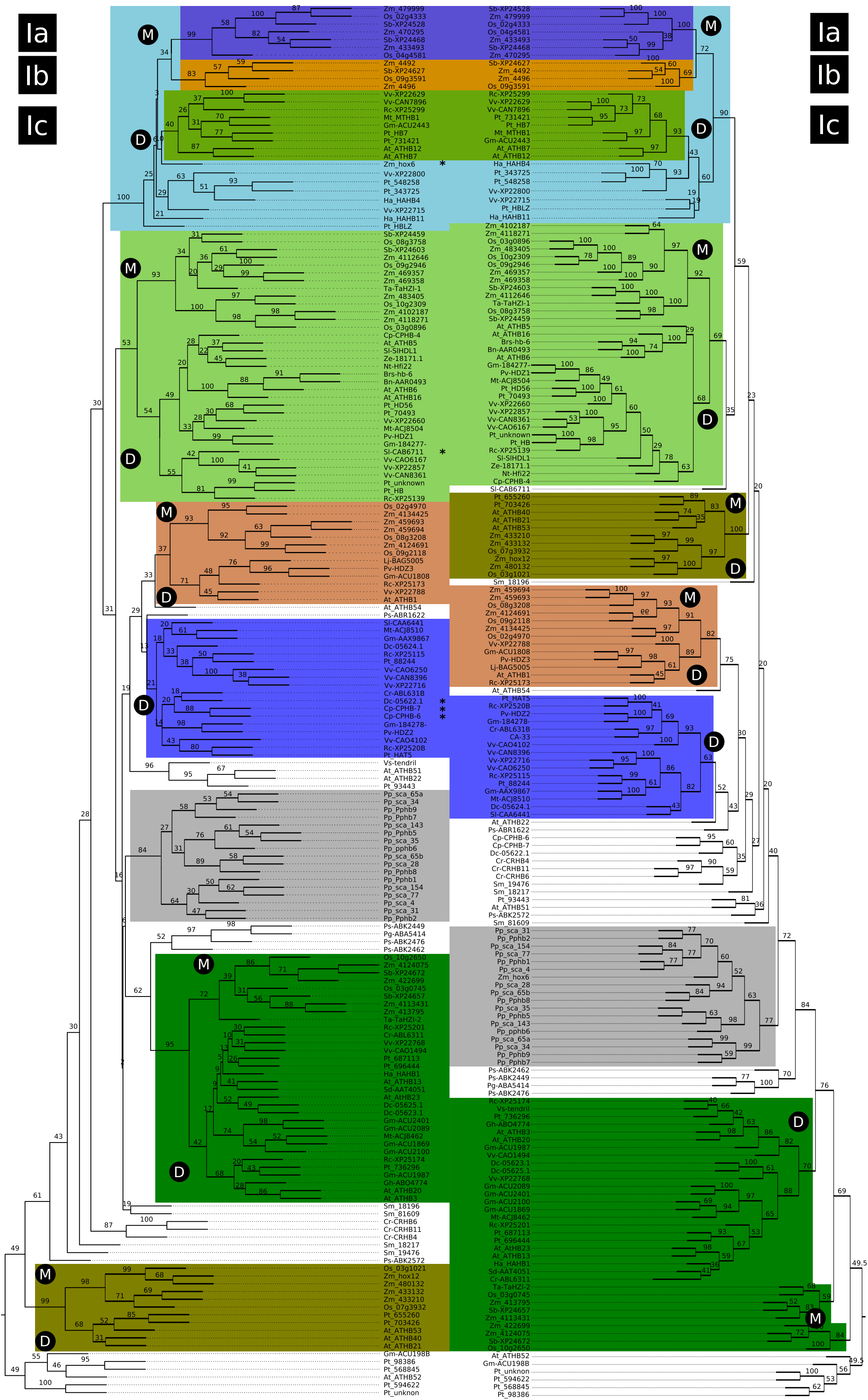

Supplement: Additional file 4 — HZT and CST. The phylogenetic trees are shown with their complete topology. [file 1471-2229-11-42-S4.PDF]

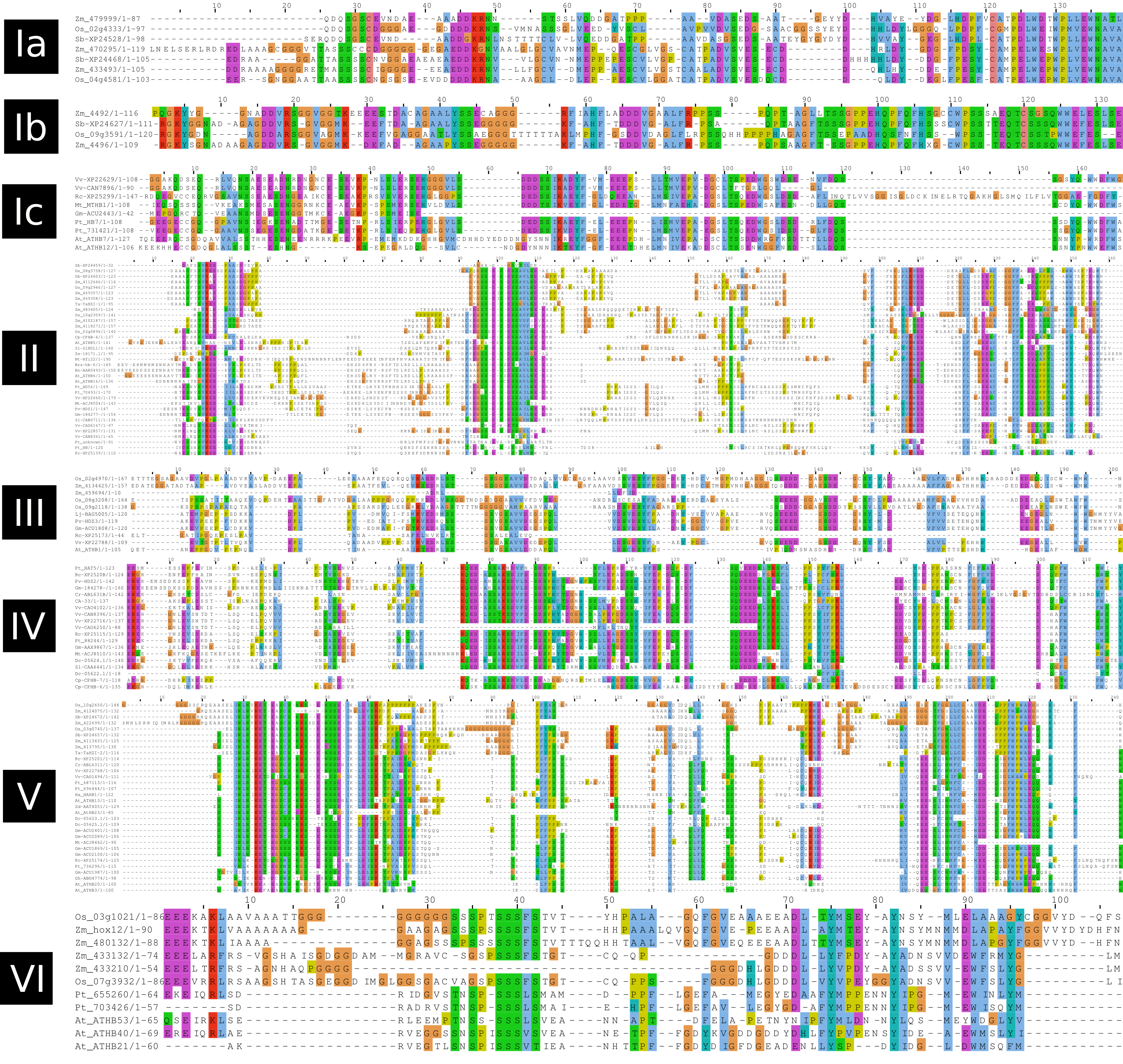

Supplement: Additional file 6 — Alignment of the CTRs. The CTRs of the proteins of each group were aligned showing the conservation in this region. [file 1471-2229-11-42-S6.PNG]

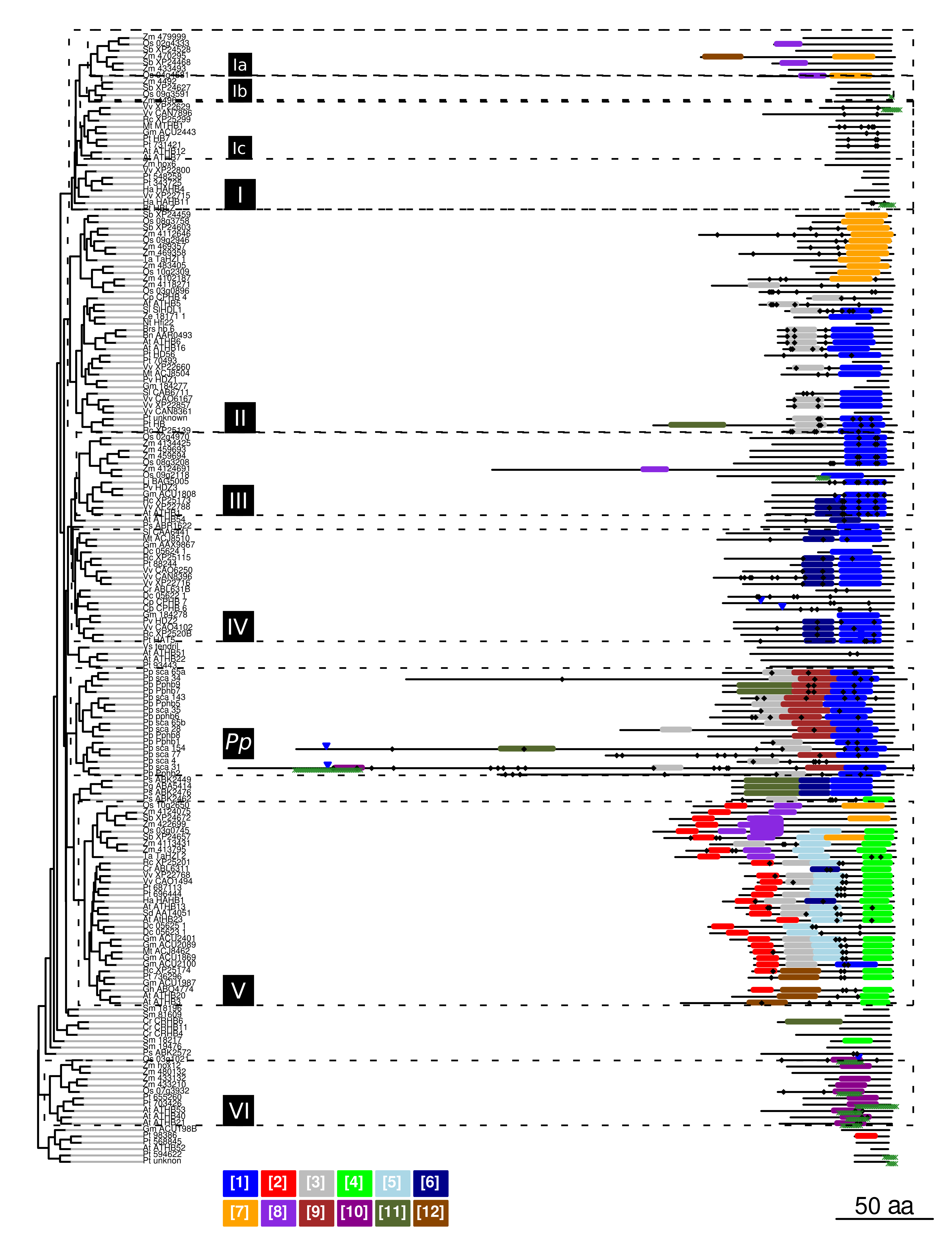

Supplement: Additional file 8 — Motif distribution in the NTRs. In an analogous representation to the one in Figure 4, the distribution of motifs in the NTRs is depicted for each protein. The tree on the left represents their phylogenetic relationships. The analysis is divided in three separate plots and the groups identified previously (i.e., I-VI) are highlighted with boxes of dashed boundaries. Putative phosphorylation sites (Ser, Thr, Tyr) are marked with a black diamond, sumoylation motifs with a blue inverted triangle and NLSs with green crosses. [file 1471-2229-11-42-S8.PNG]

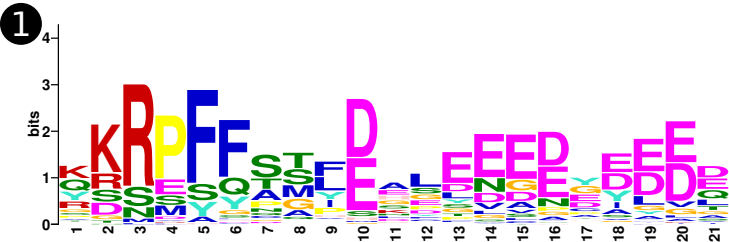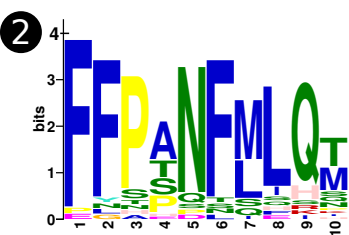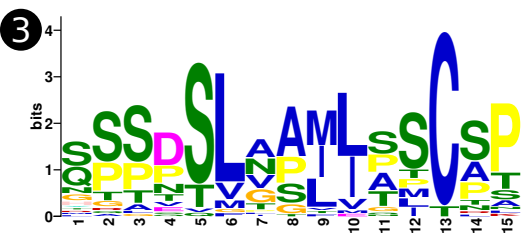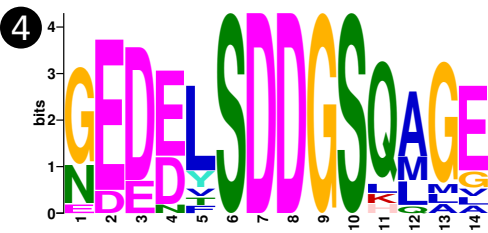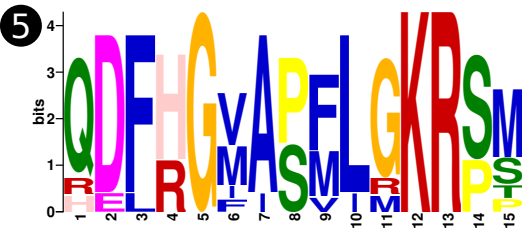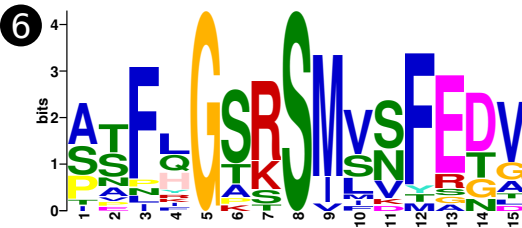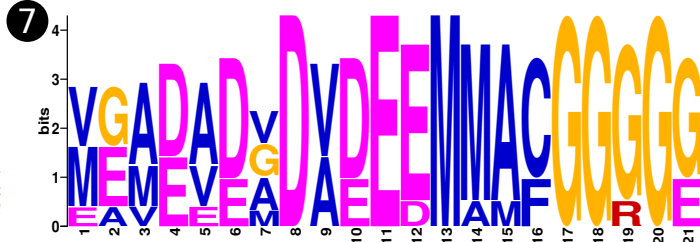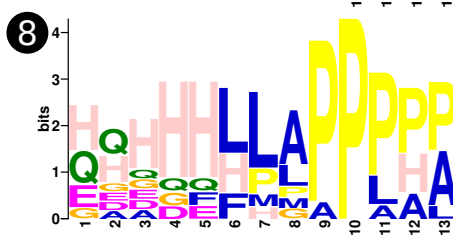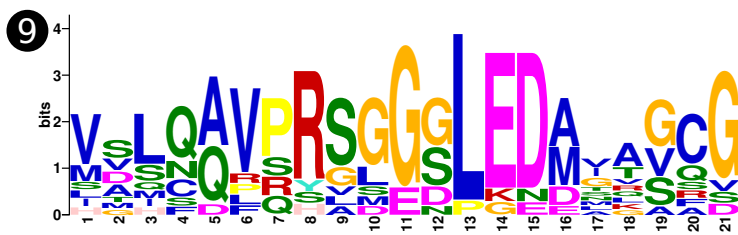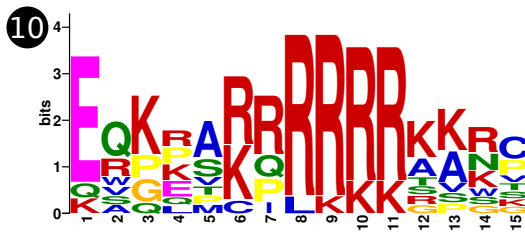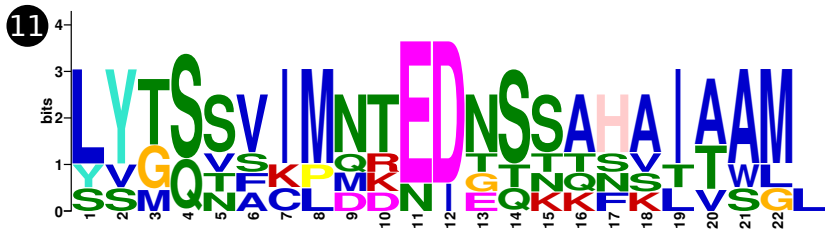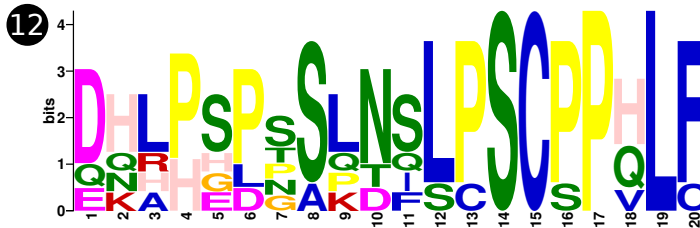

Supplement: Additional file 9 — Motifs found in the NTRs. The sequence logos of the motifs found in the NTRs by the program MEME are displayed. [file 1471-2229-11-42-S9.PDF]
