# Supplementary material for: Uncharacterized conserved motifs outside the HD-Zip domain in HD-Zip subfamily I transcription factors; a potential source of functional diversity
Source: BMC Plant Biol. 2011 Mar 3;11:42. doi: 10.1186/1471-2229-11-42 (PMC3060862; doi:10.1186/1471-2229-11-42)
Supplement: Additional file 7 — CTR putative phosphorylation sites. Each of the CTRs is represented by three stacked sequences: i) only the residues predicted to be phosphorylated are visible, the remaining are substituted with dots; ii) the visible residues correspond to motifs found by the MEME program; and iii) the complete sequence is visible. [file 1471-2229-11-42-S7.PDF]

```
Alignment: /home/
Seaview [blocks=10 fontsize=10 A4] on

              1
Zm_479999     .......... .......... ..S....... .......... .......... .......... ..........
Zm_479999     .......... .......... .......... .......... .......... .......... .......VCA
Zm_479999     QDQSGSCEVN DAEAAADDKR NNSTSSLVQD DGATPPPAAV DASEDSAATG EYYDHVAYEY DGLHDPFVCA
Os_02g4333    .......... .......... .S........ .....Y.... .......... .......... ..........
Os_02g4333    .......... .......... .......... .......... .......... .......... ..........
Os_02g4333    QDQSGSCDGG GAEGDDDDKR NSVMNASSSG LVEEDYVSCL AVPVVDVSED GSAACGGSSY EYDHHLDYLG
Sb-XP24528    .......... .......... .......... .......... ........S. .......... ..........
Sb-XP24528    .......... .......... .......... .......... .......... .......... ..........
Sb-XP24528    SERQDQSGSC EVNDAADDGK RNLNSTTTTC LVLVQEDDGA TPPAAVDASG SEDSAATEYG YGYDYDHVVA
Zm_470295     .......... .......... .......... .......... .......... .......... ..........
Zm_470295     .......... .......... .......... .......... .......... .......... ..........
Zm_470295     LNELSERLRD REDLAAAGCG GGVTTASSSC CCDGGGGGGE GAEDDKGNVA ALGLGCVAVN MEPQESCGLV
Sb-XP24468    .......... ..S....... .......... .......... .......... .......... ..S..S....
Sb-XP24468    .......... .......... .......... .......... .......... .......... ..........
Sb-XP24468    EDRAAGGATT ASSSSCNVGG AEAEAEAEDD KRNVVLGCVN NMEPPEPESC VLVGPCATPA DVSVESECDD
Zm_433493     .......... .......... .......... .......... .......... .......... .....S..S.
Zm_433493     .......... .......... .......... .......... .......... .......... ..........
Zm_433493     DDRAAAAGGG GRETMASSSS CIGGGGEEEA EDDKRNVLLF GCVDMEPPAE SCVLVGSTCA ALADVSVESE
Os_04g4581    .......... .....SS... .S.S...... .......... .......... .......... ..S.......
Os_04g4581    .......... .......... .......... .......... .......... .......... ..........
Os_04g4581    EERSGNGGAA TTAASSSSCN GSGSEEVDDD DDKRNAAAGC LDLEPPESCV LGGATCATPA DVSVESDQCD
Zm_4492       .......... .......... .......... .......... .......... .......... ......S...
Zm_4492       .......... .......... .......... .......... .......... .......... ..........
Zm_4492       PQGKYYGGNA DDVRSGGVGG TKEEEESTDA CAGAALYSSE CAGGGRFIAH FLADDDVGAA LFRPPSSPQP
Sb-XP24627    .......... .......... .......... .......... .......... .......... ..........
Sb-XP24627    .......... .......... .......... .......... .......... .......... ..........
Sb-XP24627    RGKYGGNADA GAGDDVRSGV GGMKEEFTDA AGAALYSSEG GGGGKFAHFT DDDVGALFRP SAQPTAAGFT
Os_09g3591    ...Y...... .......... .......... ......S... .......... .......... .........S
Os_09g3591    .......... .......... .......... .......... .......... .......... ..........
Os_09g3591    RGKYGDNAGD DARSGGVAGM KKEEFVGAGG AATLYSSAEG GGTTTTTTAK LMPHFGSDDV DAGLFLRPSS
Zm_4496       .......... .......... .......... ......S... .......... .......... .S........
Zm_4496       .......... .......... .......... .......... .......... .......... ..........
Zm_4496       RGKYSGNADA AGAGDDVRSG VGGMKDEFAD AGAAPYSSEG GGGGKFAHFT DDDVGALFRP SSPQPSAAGF
Vv-XP22629    ......S... ....S..S.. .......... ......S... ........S. ..SS...... ..........
Vv-XP22629    .......... .......... .......... ..KPNLSLER SEHGGGVLSD DDSSIRADYF VME.......
Vv-XP22629    GGAKQDSEQR LVQNSAESEA DNRDNGNCES EVKPNLSLER SEHGGGVLSD DDSSIRADYF VMEEEPSLLT
Vv-CAN7896    ......S... ....S..S.. .......... ......S... ........S. ..SS...... ..........
Vv-CAN7896    .......... .......... .......... ..KPNLSLER SEHGGGVLSD DDSSIRADYF VME.......
Vv-CAN7896    GGAKQDSEQR LVQNSAESEA DNRDNGNCES EVKPNLSLER SEHGGGVLSD DDSSIRADYF VMEEEPSLLT
Rc-XP25299    .......... ...S...SS. ..S....... ........S. S......... ..S...SS.. ..........
Rc-XP25299    .......... .......... .......... ......PRSV SVERSEHGLG GPSDEDSSIK AEYFGLD...
Rc-XP25299    RDQEGVCCEQ RVGSAVNSSE AESDNGEAIK CESEAKPRSV SVERSEHGLG GPSDEDSSIK AEYFGLDQEE
Mt_MTHB1      .......... ....S..S.S .......... ....S.S... ........S. ...S...... ..........
Mt_MTHB1      IEQSQSSSQV KEAKS..... .......... ..KPSPSMER SEHVLDVLSD DDTSIKVEYF GLE.......
Mt_MTHB1      IEQSQSSSQV KEAKSMESAS ENGGRNKCEA EVKPSPSMER SEHVLDVLSD DDTSIKVEYF GLEDETGLMN
Gm-ACU2443    .......T.. .......S.. .......... ....S.S... ..
Gm-ACU2443    .......... .......... .......... .......... ..
Gm-ACU2443    MEPGQRCTQV EAANSMDSES ENGGTMKCEA EGKPSPSMEI SE
Pt_HB7        .......... .......... .........S ......S... ........S. ...S...... ..........
Pt_HB7        .......... .......... .......... ..NPRLSIER PEHGLGVLSD EDSSIKAEYF ELE.......
Pt_HB7        GEEGECCGQG PAVNSIEGKS ENADTTMGES ETNPRLSIER PEHGLGVLSD EDSSIKAEYF ELEEEPNLIS
Pt_731421     .......... .....S.... .........S ......S... ........S. ...S...... ..........
Pt_731421     .......... .......... .......... ..KPRLSIEQ PEHGLGVLSD EDSSIKVDYF ELE.......
Pt_731421     VEEGECCGQG AAVNSSEGES ENGDATKGES ETKPRLSIEQ PEHGLGVLSD EDSSIKVDYF ELEEEPNLMS
At_ATHB12     .......... .......... ......S... .......... ....Y..... .......... ..........
At_ATHB12     .......... .......... .......... .......... .......... .......... .......VEK
At_ATHB12     KEEKHHECCG DQGLALSSST ESHNGKSEPE GRLDQGSVLC NDGDYNNNIK TEYFGFEEET DHELMNIVEK
At_ATHB7      .......... .......... .......... .......... .......... ........Y. .....Y....
At_ATHB7      .......... .......... .......... .......... .......... .......... ..........
At_ATHB7      TQEEERQCSG DQAVVALSST HHESENEENR RRKPEEVRPE MEMKDDKGHH GVMCDHHDYE DDDNGYSNNI
Zm_hox6       .......... .......... .......... .......... ....S..... .......... .........
Zm_hox6       .......... .......... .......... .......... .......... .......... .........
Zm_hox6       ETTGVNNKPL AALVKLLDLN SCIFTRKLDF AFRDVSTTTE GRRVSMAEKA MIPCWGFFFS VFLFFHKVS
Vv-XP22800    .......... .Y........ .......... .......... .......... S......... ..........
Vv-XP22800    .......... .......... .......... .......... MEEQTNSSLT SSADLGIFKS ...QDQSNCS
Vv-XP22800    RGKEDEESEI GYTNSETKER YNPVLQGRIE YFAEDTHTPK MEEQTNSSLT SSADLGIFKS SCFQDQSNCS
Pt_548258     .......... .SS....... ......S... ..S....... .....SS... .......... ..........
Pt_548258     .......... .......... .......... .......... .......... .......... ......TEQT
Pt_548258     HGSRNCGNQL RSSRDGRFEN KDTGSESKEK PSSPLDGNEN EENRPSSDNN GRNTVNMREE IDILNHTEQT
Pt_343725     ......S.S. .......... ...S...... .......... ..SS...... .......... .........S
Pt_343725     .......... .......... ......KPSF QLEGNDRKED KTSSDNNSRN IECTREK... ..........
Pt_343725     HGNRHQSESS HDGRFEDKGT VSESKRKPSF QLEGNDRKED KTSSDNNSRN IECTREKIDI LNQTEDAVDS
Ha_HAHB4      ....S.S.S. .......... .......... .......... .......... .......... ..
Ha_HAHB4      .......... .......... .......... .......... .......... .......... ..
Ha_HAHB4      QEKTSSSGSG EESDDRFTNS PDVMFGQEMN VPFCDGFAYF EEGNSLLEIE EQLPDPQKWW EF
```

```
Vv-XP22715   .......... .S.....S.. ......S... .......... S....S.... .......... .........S
Vv-XP22715   .......... .......... ....KLSYLE GGLDHRLVKC SDDDKSRSAG YFGHQ..... ..KCENADIS
Vv-XP22715   GDGVGSGFGG NSSTDGGSDT GDDAKLSYLE GGLDHRLVKC SDDDKSRSAG YFGHQEGPEL LDKCENADIS
Ha_HAHB11    .......... .S........ .....S.... .......... .......... ........
Ha_HAHB11    .......... .......... .......... .......... ..FDQSCSNW WDI.....
Ha_HAHB11    ENGGELKNGN SSSGPLEYMQ GDKLVSEEEE EERHENLDMA SLFDQSCSNW WDIWSSNS
Pt_HBLZ      .......... .......... .......... .......... .......... .......... .....T....
Pt_HBLZ      .......... .......... .......... .......... .......... .......... ..........
Pt_HBLZ      CKGLERCDRG IDEVLHDGIT NLEVEAQQGQ DNSTIMANVC DDMSMGNGHS GDQGQGFLYT ASSYTTLKYK
Sb-XP24459   ....S..... ..S....... .......... ..
Sb-XP24459   .......... .......... .......... ..
Sb-XP24459   DAFSSVKEEP AASDVEVPAA GAAQGSVTIL DN
Os_08g3758   .......S.. .......S.. .......... SS........ .......... .......... S.........
Os_08g3758   .AAASFSSVK EEEDPAASDA DP.....PQG SSESDSSAVL ND........ .......... ..........
Os_08g3758   DAAASFSSVK EEEDPAASDA DPPATGAPQG SSESDSSAVL NDAEILPHKP APAAAADAAA SEETEAVVTG
Sb-XP24603   .......S.. .......... ......S... .......... .......... .......... ..........
Sb-XP24603   .AAASFTSVK AEPAASDGPP PVGVGSSESD SSAVLND... .......... .......... ........AA
Sb-XP24603   EAAASFTSVK AEPAASDGPP PVGVGSSESD SSAVLNDAGP PVPEAQPVPE VQGTLLDDAP CAVVAGASAA
Zm_4112646   .......S.. .......... ......S... .......... .......... .......... ..........
Zm_4112646   .AAASFTSVK AEPAASDGPP PVGVGSSESD SSAVLND... .......... .........A VAANHGGVFF
Zm_4112646   EAAASFTSVK AEPAASDGPP PVGVGSSESD SSAVLNDADP PVAEAPVPEV RGTLLDAPGA VAANHGGVFF
Os_09g2946   .......S.. .......... ......S.S. .......... .......... .......... ..........
Os_09g2946   .AAASFTSVK EEPAASDGPP AAGFGSSDSD SSAVLND... .......... .......... ..........
Os_09g2946   EAAASFTSVK EEPAASDGPP AAGFGSSDSD SSAVLNDVDA AGAAPAATDA LAPEACTFLG APPAAGAGAG
Zm_469357    ........S. .......... .......... S......... .......... .......... ..........
Zm_469357    ..AAASFTSV KAEPAASDGP APA.VGSSEI SDSSAVLND. .......... .......... ........AA
Zm_469357    EDAAASFTSV KAEPAASDGP APAGVGSSEI SDSSAVLNDA DPPVAEAPAP APEVQGTLLV APAGPVAGAA
Zm_469358    ........S. .......... .......... S......... .......... .......... ..........
Zm_469358    ..AAASFTSV KAEPAASDGP APA.VGSSEI SDSSAVLND. .......... .......... ........AA
Zm_469358    EDAAASFTSV KAEPAASDGP APAGVGSSEI SDSSAVLNDA DPPVAEAPAP APEVQGTLLV APAGPVAGAA
Ta-TaHZI-1   .......S.. .......... ......S.S. .S........ .......... .......... ..........
Ta-TaHZI-1   .AAASFTSVK EEPAASDGPP PAGMGSSDSD SSGVLND... ..AAAAGHGQ VFLHGNFLKV EEDETGFLDD
Ta-TaHZI-1   EAAASFTSVK EEPAASDGPP PAGMGSSDSD SSGVLNDTDA LGAAAAGHGQ VFLHGNFLKV EEDETGFLDD
Zm_483405    .......... .......... .......... ...S..S... ....S..... .......... ..........
Zm_483405    .......... .......... .......KDG STDSDSSAVF NE........ .......... ..........
Zm_483405    MEVKLESAAE ELLPVATRSA AAAAVYNKDG STDSDSSAVF NEEASPYPYS GAALDHQQQQ ETSHPLGFTG
Os_10g2309   .......... .......... .......... ......S..S .......S.. .......... ..........
Os_10g2309   .......... .......... .......... VDGSTDSDSS AVFNE..... .......... ..........
Os_10g2309   MSVKLEAVAA DEHQPPPPPP PPPLAYNSKV VDGSTDSDSS AVFNEEASPY SGAAIDHHHH QTPASYDTAG
Zm_4102187   ...T...... .......... .......... ......S... .S...S.... S......... ..........
Zm_4102187   .......... .......... .......... ..KDGSSDSD SSVVFSD... .......... ..........
Zm_4102187   PEATVKLEAT TGNDTAEERR QATAGAPPAG ACKDGSSDSD SSVVFSDVEA SPYSGGAAFE PPALAGLGAP
Zm_4118271   ...T...... .......... .......... ......S... .S...S.... S......... ..........
Zm_4118271   .......... .......... .......... ..KDGSSDSD SSVVFSD... .......... ..........
Zm_4118271   PEATVKLEAT TGNDTAEERR QATAGAPPAG ACKDGSSDSD SSVVFSDVEA SPYSGGAAFE PPALAGLGAP
Os_03g0896   ......S... .......... .........S ...S..S... ....S..... .......... ..........
Os_03g0896   .......... .......... .......DGS SDDSDSSVVF ND........ .......... ..........
Os_03g0896   PADTAASVKV EAGNDAAAGA AAATVCKDGS SDDSDSSVVF NDEASPYSGA AFIGFGPSFL VDDASAATVG
Cp-CPHB-4    ....S..... .......... .......... ....S.S... .......... ..S....... .........S
Cp-CPHB-4    .......... .......... .......... LFGSSDSDSS AILNE..... .......... ..........
Cp-CPHB-4    DDSKSVEEEP FVEALELDAN SDDVEPDSNQ LFGSSDSDSS AILNEDNNNS RSSFLDDHRR KLSKIHGGSS
At_ATHB5     .......... .......... .......... ..S.S..... ......S... .......... .........S
At_ATHB5     ...GIEENGA LKAVEANQSV MANNEVLELS HRSPSPPPHI PTDA...... .......... ....DDPADS
At_ATHB5     GVKGIEENGA LKAVEANQSV MANNEVLELS HRSPSPPPHI PTDAPTSELA FEMFSIFPRT ENFRDDPADS
Sl-SlHDL1    .......... .......... .......... .......... .......... ......S... ..........
Sl-SlHDL1    .......... .......... .......... .......... .......... ..KDGPSESD SSAILNE...
Sl-SlHDL1    TENKICVKEE AMMCDSENNG KDIESITPQP SLDEVSDDAK LQNTQKRGVT DLKDGPSESD SSAILNEDNN
Ze-18171.1   .......... .......S.. .......... .....S.S.. S......... .......... ..........
Ze-18171.1   .......... .......... .......... .KDGSSDSDS SAIMGD.... YQPQFVKLEE HNFFG.DESC
Ze-18171.1   DEPDNIPMPE QSDDKPKSPE NMVETAYFPD FKDGSSDSDS SAIMGDAQKA YQPQFVKLEE HNFFGGDESC
Nt-Hfi22     .......... .....S.... .......... .......... .......... .......... ..........
Nt-Hfi22     .......... .......... .......... .......... .......... .......... ..........
Nt-Hfi22     NGESKGVAVK EEAMESESDD NKVIEQSKPN DNDNNNNNFL ENFEEDDEEE EINFENFNVA AAATSTNIFG
Brs-hb-6     .......... .......... S......S.. ........S. .......... ...S...... ..........
Brs-hb-6     .......... ....TMECDV SVKEEEVSLP EELTD..... .......... .......... ..........
Brs-hb-6     EEVEKMMKME NNAVTMECDV SVKEEEVSLP EELTDPPSSP PQALEHSDSF NYRSFTDLRD LLSLKAAASS
Bn-AAR0493   .......... .......... .S......S. .........S .......... ....S..... ..........
Bn-AAR0493   .......... .....TMESD VSVKEEEVSL PEELTD.... .......... .......... ..........
Bn-AAR0493   EEVEEDDEDE ENNAVTMESD VSVKEEEVSL PEELTDPPSS PPQLLEHSDS FNYRSFTDLR DLLPLKAAAS
At_ATHB6     .......... .........S .......... .......S.. .......... ..S....... ..........
At_ATHB6     .......... ...TTESDIS VKEEEVSLPE KITE...... .......... .......... ..........
At_ATHB6     GGEEEEEENN AAVTTESDIS VKEEEVSLPE KITEAPSSPP QFLEHSDGLN YRSFTDLRDL LPLKAAASSF
At_ATHB16    .......... .......... .......... .......... ..S....... .......... ..........
At_ATHB16    .....NKAIT EGVKEEEVHK TDSIPS.... .......... .......... ........AG SSDSCDSSAV
At_ATHB16    EDNNNNKAIT EGVKEEEVHK TDSIPSSPLQ FLEHSSGFNY RRSFTDLRDL LPNSTVVEAG SSDSCDSSAV
Pt_HD56      ......S... .......... .....T.... .....S.... ..Y....... .......... ........S.
Pt_HD56      NAESNVSVKE EIILAESEDK M......... .......... .......... .......... ....KDGSSD
Pt_HD56      NAESNVSVKE EIILAESEDK MPEEDTPALL DSVAASETKE LNYETFNNHS SINIGLGASL FPDFKDGSSD
Pt_70493     ......S... .......... .......... .....S.... ...Y...... .S........ .........S
```

```
Pt_70493    NTESNVSVKE EIILAESEDK V......... .......... .......... .......... .....KDGLS
Pt_70493    NTESNVSVKE EIILAESEDK VTEEDTPPLL DSLTASAEAK ELNYENFNSS SSINNGLGAS LFPDFKDGLS
Vv-XP22660  ......S... ....S..... .......... .......... ...S...S.. .......... .........S
Vv-XP22660  KTESNLSVKE ELVVSESDEK V......... .......... .......... .......... ....KDGSSD
Vv-XP22660  KTESNLSVKE ELVVSESDEK VKVMEQSETA MGAGVGGSDA KDLSNDDSFK DGNGGGRVSV FPDMKDGSSD
Mt-ACJ8504  .......... .......... ........S. ...SS..... .......... .......... .........S
Mt-ACJ8504  .KSTINVLVK EELTMLESCD ED........ .......... .......... .......... .....KDGSS
Mt-ACJ8504  EKSTINVLVK EELTMLESCD EDKHNPSSET SNPSSESKDH LDYDCIINNN DVGIGETSSL FPVDLKDGSS
Pv-HDZ1     .........S .......... .......... ...S...SY. .......... .....S.S.. ..........
Pv-HDZ1     ...NTESDVS VKEEMITLQD SNPL...... .......... .......... .KDGSSDSDS SAILNE....
Pv-HDZ1     EESNTESDVS VKEEMITLQD SNPLCETAIP GSESKELSYE CFNKSDEVLG FKDGSSDSDS SAILNEENAN
Gm-184277-  .......... S........T .......... ....SY.... .......... .......... S.S.......
Gm-184277-  ....NTESDV SVKEEMLTTT LCETA..... .......... .......... ......KDGS SDSDSSAILN
Gm-184277-  ENNNNTESDV SVKEEMLTTT LCETAIPGSD TKELSYECFN KSDEVGGGSS VFHVDFKDGS SDSDSSAILN
Sl-CAB6711  .......... .......... .........S S.S..S.... ...Y...... .........S ..........
Sl-CAB6711  .......... .......... ......KDGS SDSDSSGVMN E......... .......... ..........
Sl-CAB6711  TLLSPVFQRP QEILNKLKNS SDFVDSKDGS SDSDSSGVMN EETYNISTLN YQQLMPKVSS YSNSLDHLSL
Vv-CAO6167  ......S... ....S..... .......... ....S..S.. .........S .......... ..........
Vv-CAO6167  NMELNQSVKE EALSRVRTME I......... .KTASSSSLR LNCASLSSPS PM.......F QHQFGRMEEQ
Vv-CAO6167  NMELNQSVKE EALSRVRTME IYQESMDWET SKTASSSSLR LNCASLSSPS PMNWFPSKAF QHQFGRMEEQ
Vv-XP22857  ......S... .......... .......... .......... ....S.S.S. .......... ........S.
Vv-XP22857  NMELNQSVKE EALVSESENN V......... .......... KDGSSDSDSS GVLKN..... ..........
Vv-XP22857  NMELNQSVKE EALVSESENN VSEQGKNNGN ISGIHGLGDF KDGSSDSDSS GVLKNESNFN AQLMMSPASS
Vv-CAN8361  ......S... .......... ........SS .......... .......... .......... .....
Vv-CAN8361  NMELNQSVKE EALWERDRPK A..TMTLNSS KSELPLSEDG PITP...... .......... .....
Vv-CAN8361  NMELNQSVKE EALWERDRPK AAVTMTLNSS KSELPLSEDG PITPSFCHPR LTVNALWNLA CQPLS
Pt_unknown  ......S... ....S..... .......... .......... .......... .......... ..........
Pt_unknown  NVDSSHSVKE EHRVSESDNN A......... .......... .......... .......... ..........
Pt_unknown  NVDSSHSVKE EHRVSESDNN ASVHRLHFMF SSTGFGVNNH ITATNAIEVA RCSGQLARTL TNLTVPEVNE
Pt_HB       ......S... .......... .......... .......... ......S... .......... ..........
Pt_HB       IVDSSHSVKE EYHVSESDNN A......... .......... .......... .......... ..........
Pt_HB       IVDSSHSVKE EYHVSESDNN ASVHSQNHDF SEHKNSSAVT KDHSNVSSSN ELMNCFNLTD SRAILGNRTS
Rc-XP25139  ......S... ....S..... ......S... ........S. .......... .......... ..........
Rc-XP25139  NAESSQSVKE ECPVSESENN A......... .......... .......... ..MNWIQLSD SRAILGN...
Rc-XP25139  NAESSQSVKE ECPVSESENN ASVQSQSHEF SDNNNSNGSF KDNISDLSSH TLMNWIQLSD SRAILGNGYQ
Os_02g4970  .......... .......... .......... .......... .......... ......S... ..........
Os_02g4970  .......... .......... .......... .......... .......... .......TGS GGSAVVDTDA
Os_02g4970  ETTTEGSAGA AVDVPGLPAA ADVKVAVPDA EEPALEEAAA AFEEQQEQQV KAEDRLSTGS GGSAVVDTDA
Zm_4134425  .......... .......... .......... .......... .........S .......... ..........
Zm_4134425  .......... .......... .......... .......... .......... TGSGGSAVVD ADALLYGRFA
Zm_4134425  EDATEGGATA DTAAPAVDVE ASLADDVEEP AEPAATFEVL QEVKSEDRLS TGSGGSAVVD ADALLYGRFA
Zm_459693
Zm_459693   .
Zm_459693
Zm_459694
Zm_459694   ..........
Zm_459694   ADRLLLDFID
Os_08g3208  .......... .......... .......... .......... .......... .......... ..........
Os_08g3208  .......... .......... .......... .......... .......... .......... ..........
Os_08g3208  ETSPSSATIT TAAQEVDQPD EHTEAASTTG FATVDGALAA PPPGHQQPPH KDDLVSSGGT NDDGDGGAAV
Zm_4124691
Zm_4124691  .
Zm_4124691
Os_09g2118  ....S.S... ...T...... .......... .......... .......... .......... ....S..S..
Os_09g2118  .......... .......... .......... .......... .......... .......... ..........
Os_09g2118  GKSPSPSPAP AEQTAVPAAP ESAKSFQLEE GRRLYDAAGS TTTTNGGGGG VAMPAARVAA ARAASNDSPE
Lj-BAG5005  .........S .......... .......... .......... .......... .......... ..........
Lj-BAG5005  .......... .......... .......... ....SGSVGS AVVDEGSPQL VVDSV..... ..........
Lj-BAG5005  ATEMPGEPPS DKKADPLPVD MVQIFSMKVE DHMSSGSVGS AVVDEGSPQL VVDSVDSYFP ADNYVECVVA
Pv-HDZ3     .........Y .......... .......... .......... .......... .......... ..........
Pv-HDZ3     .......... .......... .......... .....SGSVG SAVVDEGSPQ LVVDSV.... ..........
Pv-HDZ3     AKEVPEEPFY DKKVDPLPVE DIATIFSTRV EDHQSSGSVG SAVVDEGSPQ LVVDSVDSYL PADNPGGCVG
Gm-ACU1808  .......... .......... .......... .......... .......... .......... ..........
Gm-ACU1808  .......... .......... .......... ......SGSV GSAVVDEGSP QVVVDSV... ..........
Gm-ACU1808  AKEVPEEPLC DKKVDPIPVD EDMAPIFGTR VEDHLSSGSV GSAVVDEGSP QVVVDSVDSY ILADNYGGCV
Rc-XP25173  .......... .......... .......... ....S..... ....
Rc-XP25173  .......... .......... .......... .......... ....
Rc-XP25173  ELTGATIPGQ EPESLPAVTA NASAFELNVK LRTGSALEAL EVQW
Vv-XP22788  .......... .......... ........S. ......SS.. .......... .........Y ..........
Vv-XP22788  .......... .......... .......... .......SGS GASAVVDEDG PQLLDSGD.. ..........
Vv-XP22788  EVTGTPTLTV QKVEPLQADA ADVPPVPCSV KVEDRLSSGS GASAVVDEDG PQLLDSGDSY FQNAEYPGCL
At_ATHB1    .......... .......... Y......... ....SS.S.. .......... .......... ..........
At_ATHB1    .......... .......... .......... ....SGSVGS AVLDDDAPQL LDSCD..... ..........
At_ATHB1    QETANEPPGQ VPEPNQLDPV YINAAAIKTE DRLSSGSVGS AVLDDDAPQL LDSCDSYFPS IVPIQDNSNA
At_ATHB54   .......... .......... .......... .......... S........S .......... ..........
At_ATHB54   .......... .......... .......... .......... .......... .......... .....VLGFY
At_ATHB54   SVQNFDDTFK QVDLLKEKLK MQENLETQSI ERKRLGEEGS SVKSDNTQYS EEEGLENQYS FPELAVLGFY
Ps-ABR1622  .......... ...T...... .......... .......... .......... S.....S... .........S
Ps-ABR1622  .......... .......... .......... .......... .......... .......SGT DGSAVVDEES
```

```
Ps-ABR1622   EKGLEIQTND LETTCKKTFI QSNSQFESLE KSGIVSKGMT APFDQQLVSY SIEDPLSSGT DGSAVVDEES
Sl-CAA6441   .......... .......... .......... .......... ....S..... ....S...TS ...SS.....
Sl-CAA6441   EKGNGQLDLR DEHKHSNALA KETVVDPMSN VPALVVKHQQ E.LSSAKSDV FDSESPRYTS RMH..VVDQD
Sl-CAA6441   EKGNGQLDLR DEHKHSNALA KETVVDPMSN VPALVVKHQQ EDLSSAKSDV FDSESPRYTS RMHSSVVDQD
Mt-ACJ8510   .......... .......... .........S .S.......S .......... .......S.. .S......S.
Mt-ACJ8510   ...EGKFKQG ESETKEFLKE PTINKPLVDS VSEGEGSKLS IVEA...... ......ISSA RSDILDCESP
Mt-ACJ8510   EKQEGKFKQG ESETKEFLKE PTINKPLVDS VSEGEGSKLS IVEASNNNNN NNKLEDISSA RSDILDCESP
Gm-AAX9867   ....S..... .......... .....S.S.. .......... .....S...S .......S.. Y.........
Gm-AAX9867   .......... .......... .......... .......... .......... .......... ........LL
Gm-AAX9867   EKNESHLEQA ETNGLQEPLH KSLVDSASEG EGSKVTFEAC KQEDISSAKS DIFDSSESPQ YTDGVHSALL
Dc-05624.1   .......... .......... .......... ......S... .......... .......S.. .....Y.S..
Dc-05624.1   EDKGSKTVVF DKQKVSAAFQ QERVSNDISV GEVLSNSVMD C....HNSVK SDAVDSDSPH YSDEVY..FM
Dc-05624.1   EDKGSKTVVF DKQKVSAAFQ QERVSNDISV GEVLSNSVMD CKQEDHNSVK SDAVDSDSPH YSDEVYSSFM
Rc-XP25115   ......S..S .....S.... ......S... .......... ......S... S......S.. Y.........
Rc-XP25115   EKEKVNSEVS DKDALSQELS KKPIGDSASE VEVSKASTVA F....ISSAK SDIFDSDSPH YTDGVH..LL
Rc-XP25115   EKEKVNSEVS DKDALSQELS KKPIGDSASE VEVSKASTVA FKQEDISSAK SDIFDSDSPH YTDGVHSSLL
Pt_88244     .....SS..S .....S.... ......S.S. ...S...... .......... S......S.. ..........
Pt_88244     EKEKGSSELS DKDALSQEPP KKAIADSASE GEVSKTSTVA C....ISSAK SDMFDSDSPH FADGVH..LL
Pt_88244     EKEKGSSELS DKDALSQEPP KKAIADSASE GEVSKTSTVA CQQEDISSAK SDMFDSDSPH FADGVHSSLL
Vv-CAO6250   .........S .......... .......... .......... .......... .......... ...Y......
Vv-CAO6250   EKERGNLEVS NTDTLSQELP QVVVADSSRA ILLMFLKLTN Q......... .......... ...YPDPPTN
Vv-CAO6250   EKERGNLEVS NTDTLSQELP QVVVADSSRA ILLMFLKLTN QTYNFSKSLL PPSYIFPKLE DVDYPDPPTN
Vv-CAN8396   .........S .......... ......S... .......... ......SS.. .......... Y.........
Vv-CAN8396   EKERGNLEVS NTDTLSQELP QVVVADSVSE GEVSKVSLVV C....LSSTK SDVFDSDSPH YADGGH..LP
Vv-CAN8396   EKERGNLEVS NTDTLSQELP QVVVADSVSE GEVSKVSLVV CKQEDLSSTK SDVFDSDSPH YADGGHSALP
Vv-XP22716   .........S .......... ......S... .......... ......SS.. .......... Y.........
Vv-XP22716   EKERGNLEVS NTDTLSQELP QVVVADSVSE GEVSKVSLVV C....LSSTK SDVFDSDSPH YADGGH..LP
Vv-XP22716   EKERGNLEVS NTDTLSQELP QVVVADSVSE GEVSKVSLVV CKQEDLSSTK SDVFDSDSPH YADGGHSALP
Cr-ABL631B   ......S... .......... .......... .......... ...S..S... ...S...... ..........
Cr-ABL631B   .......... .......... .......... .......... .......... ......CTDG NHSSNVFEAE
Cr-ABL631B   EKEKGKSKTC DSLCGFDIEP DEKQLASNSA VCLPGIKQED AASSAKSDVF DSDSPHCTDG NHSSNVFEAE
Dc-05622.1
Dc-05622.1   SCFIQILLDC QCACD...
Dc-05622.1   SCFIQILLDC QCACDYTV
Cp-CPHB-7    .......... .......... .....SS... .....S.... .......... .......... ...S......
Cp-CPHB-7    .......... .......... .......... .......... .......... .......... ..........
Cp-CPHB-7    ADHGDHRPIK EEPPFDDDVN KQTKASSAKS DVLDYSDADG GNQRSPTMLE LEHGDSSHVV GAISDYEDDD
Cp-CPHB-6    .......... .......... .....S..SS ..S....... .S.....SS. .......... ..........
Cp-CPHB-6    .......... .......... .......... .......... .......... .......... ..........
Cp-CPHB-6    EKGRDQLIMK ENLEPLGGED DVKKQSKTSS AKSDVVDCSD SSPRHGDSSH DVFEAAAADA ISDYYYGEEE
Gm-184278-   .......... .SS....... .S........ .......... ....S..... .......... ..........
Gm-184278-   .......... .......... .......... ......KNAT TTTTSENGTK VLSPLPLPIM VTCCKQE.AN
Gm-184278-   DKEKEENSDD KSSPDDAVNS SSPHNNKEPM DLLIISKNAT TTTTSENGTK VLSPLPLPIM VTCCKQEDAN
Pv-HDZ2      ......S... S......S.. .........S .S........ .......... ..S....... S.S.......
Pv-HDZ2      .......... .......... .......... .......... .......... ANSAKSDVLD SDSPHCTDGN
Pv-HDZ2      DKEKENSDDK SSPDAVNSPH KEPMDLISNS TSENGTKVSL PIMVTCKQED ANSAKSDVLD SDSPHCTDGN
Vv-CAO4102   .......... ...S.S...S ........S. .......... ......SS.. S......... Y.........
Vv-CAO4102   EKEGEKTKAL ENISPSHAES QKAIPNRVSE HVPPNAPILF C....ASSAK SDVFDSDSPL YTDGNH..LL
Vv-CAO4102   EKEGEKTKAL ENISPSHAES QKAIPNRVSE HVPPNAPILF CKQEDASSAK SDVFDSDSPL YTDGNHSSLL
Rc-XP2520B   .......... .......... ........S. ........T. .....SS..S ......S..S ..........
Rc-XP2520B   .......... .......... .......... .......... ....ASSAKS DIFDSDSPHS FLEPA.....
Rc-XP2520B   EKGRENFEPF HAINSVNEEP QNSIPMTVSG KDSNAPIVTP KQEDASSAKS DIFDSDSPHS FLEPADSSNV
Pt_HAT5      .....S.... .......... .......... ........T. .....S.... ........S. ..........
Pt_HAT5      .......... .......... .......... .......... ...ESSAKND VFNSDSPRSF LEPR......
Pt_HAT5      EESMESSEPF DVIHSPDAEL EPIPDTVSEN VSAIVPMVTP KQEESSAKND VFNSDSPRSF LEPRDCYRVF
Vs-tendril   .......... ....S..... .......... .......... .......... Y......... ..........
Vs-tendril   .......... .......... .......... .......... .......... .......... ..........
Vs-tendril   GDCRTQAFGD ETVESPLEGL GWREIEGYNP YPSCYNQQGT TSSTQQAAEG YINSSFIVED FDSVSLHQEC
At_ATHB51    ...T...S.. .......... .......... .......... .......... .........
At_ATHB51    .......... .......... .......... .......... .......... .........
At_ATHB51    SAGTIKVSGE EDTVEISSVV VAHPRTENMN ANQITGGNQV YGQYNNPMLV ASSGWPSYP
At_ATHB22    .S........ ....
At_ATHB22    .......... ....
At_ATHB22    SSCKKKQTWE KACS
Pt_93443     .......S.. ......S... ........S. .......... .......... .S........ ..
Pt_93443     .......... .......... .......... .......... .......... .......... ..
Pt_93443     TAGYTEISGE ETVESTSVAA ASRKLRGHSH HQNNAEHCNY LLNVDEYNPV SSPYWALLPS YP
Pp_sca_65a   .......S.. .......... .....S.... .S..SY.S.. ....S..... .......... ..........
Pp_sca_65a   .......... .......... .......... ..SDSYSSDV MDAESPRTGD SSN....... ..........
Pp_sca_65a   IVTSKVRSTP TIDVLPGAAK EPNERSVKRT MSSDSYSSDV MDAESPRTGD SSNPNLPTDA PWVQYHHPQF
Pp_sca_34    .......... .......... .S......S. .......... .......S.. .......S.. S.........
Pp_sca_34    .......... .......... .......... .......... .......... ........YD SNSSDVMDAE
Pp_sca_34    PKNVDAPKLK HFKGQPVSPA HSEKSDIVSS KTHPTPTIDV LPIADQESTE HRFKRTMSYD SNSSDVMDAE
Pp_Pphb9     .......... .......... S...S..... .......... .......S.. .......... ..........
Pp_Pphb9     .......... .......... .......... .......... .......... ........PD CNSSNTIDAD
Pp_Pphb9     PDSPITTPIR CLSETMHHPA SPAQSEKSDI ISCRNCASPS IDVNPVPSKE AGVTGTMSPD CNSSNTIDAD
Pp_Pphb7     .......... ........S. ..S....... .......... .......... ........
Pp_Pphb7     .......... .......... .......... .......... .......... ........
Pp_Pphb7     VKPAEFVQGK CDTTSHPASP AQSERSDIVS SRNRTTPTIH VDPVAPEEAG AHLTIELG
```

```
Pp_sca_143   .......S.. .....T.... .......... .TS..SS... .......... S......... ........S.
Pp_sca_143   .......... .......... .......... .......... .......... ......NDGN SGDVVNADSP
Pp_sca_143   PQTDRNKSVN DRAPQTPERE VVISGAVARQ RTSRISSTVD ISLVCAKDQD SETNGLNDGN SGDVVNADSP
Pp_Pphb5     ......T... ....S.S... ......S... .......... .......... ....S..... .S..S.....
Pp_Pphb5     .......... .......... .......... .......... ..TDGNSSDI VDADSPRTTN SSR.......
Pp_Pphb5     CLESIQTPER DRHVSDSDAR QLNSRSSPTV DISRVKDEIS GSTDGNSSDI VDADSPRTTN SSRKSVIQSS
Pp_sca_35    .......... ...S...... ....S..... ......S... .......... .S...S.S.. ....S..S..
Pp_sca_35    .......... .......... .......... .......... .......... ..TDGSSSDI VDADSPRSTD
Pp_sca_35    LGYKQGDDNQ GAESSKIPER DLDVSISVAR QHNARASPTV DTSLAKEETR GSTDGSSSDI VDADSPRSTD
Pp_pphb6     .......... .....T.... .......... .T...T.... .......... .......... ........S.
Pp_pphb6     .......... .......... .......... .......... .......... ......TDGN SSDVLDADSP
Pp_pphb6     PCADGNQIVA GESAQTPEVD VETSELVARQ HTSRSTPTVD VSLACVKDKD FETSGCTDGN SSDVLDADSP
Pp_sca_65b   ....S..... .......... ......S... T......... ...S.SS... ........S. .......S..
Pp_sca_65b   .......... .......... .......... .......... ......SGSN SSDLLDADSP HTSDSNP...
Pp_sca_65b   DTEESPKLGK KPYNLAITKD HHHTRSSPTM TVCSTPKLEV EKTSTSSGSN SSDLLDADSP HTSDSNPSDE
Pp_sca_28    ......S... .S........ .......... S........S .......... ......S... S..S......
Pp_sca_28    .......... .......... .......... .......... .......... .......... ...SGSNSSD
Pp_sca_28    DSQGCQSPAD PSQCESETTC SDKTKPDIAV SLKDHYARSS QTVDVVSSAH KLTNGDSKEM STSSGSNSSD
Pp_Pphb8     .......S.S .......... .....S.... .S...T.... .S...S.... .......... .....S....
Pp_Pphb8     .......... .......... .......... .......... .......... .......... .....SGSNS
Pp_Pphb8     DSQSVDFSQS EKDSHCKPTA NDPAKSDKLK ESKPATPKDN QSTRSSPTTV VCSATTLEAE ITSTSSGSNS
Pp_Pphb1     .......... ......S... ..S....... .......... .......T.. ....S....S ......S...
Pp_Pphb1     .......... .......... .......... .......... .........C KEEGSKEEGS KETSSDSISQ
Pp_Pphb1     IPNSADPILE KRKRIISMSQ PSSLKTFEGP VKSERIAAEA NGRNDALTPC KEEGSKEEGS KETSSDSISQ
Pp_sca_154   .......... .......... .S.......S .......... .......... ....SS...S .S........
Pp_sca_154   .......... .......... ...PKMSEAS VKLEQICTEH TASY...... .........S DSMSSEIVNA
Pp_sca_154   QPSSVLGAKE QSKKLKTASQ PSPPKMSEAS VKLEQICTEH TASYDALVPF LEGDSSFESS DSMSSEIVNA
Pp_sca_77    .......... .......... .S....S... .......... .......... .......... ...S......
Pp_sca_77    .......... .......... .......... .......... .......... .......... .SDSLSSEIL
Pp_sca_77    QPSPAGNPQE KLKKPKTATQ PSPPRTSEAP VKLERTSAAH KARNGDLTRP HVGAGLRVTF SSDSLSSEIL
Pp_sca_4     .......S.. .S........ .......... ....S..... ......S... .......... ..........
Pp_sca_4     .......... .......... .......... .......... ......SDSF SSELFNSDSP CTMNSGS...
Pp_sca_4     QPGPADPSLE KSKKHISMSH PSPQWACEAN NDGLSPCKEE GSKETFSDSF SSELFNSDSP CTMNSGSLLP
Pp_sca_31    .......... ...S...S.. .....S...T S......... ..S....SS. .........T S..S.S....
Pp_sca_31    .......... .......... .......... .......... .......... .......... .SDSISSEIL
Pp_sca_31    SQPGPVDGAP EKPSKRLSKA VPSQLSPPKT SSEACVKSER KPSDPNASSN DDKEEGSRAT SSDSISSEIL
Pp_Pphb2     .......... ........S. ..S..S.... .......... S......... ......SS.. ........TS
Pp_Pphb2     .......... .......... .......... .......... .......... .......... ..........
Pp_Pphb2     CLTGRLQTSP QLASGDGVSE KPSKKSSKAV ASQLSLQKMV SEECVKLESK PQDPNDSSND DKEEGSRATS
Ps-ABK2449   .......... .......... .......... .......... .........S .......... ..........
Ps-ABK2449   .......... .......... .......... .......... ...IGSEGSS VLDMDSPGTI DSQQ......
Ps-ABK2449   NDQKFQANSS KLQKDDQDLQ LLMMSATKVD CADKENNNEG PSSIGSEGSS VLDMDSPGTI DSQQNIDSIG
Pg-ABA5414   .......... .......... .......... .......... .........S .......... ..........
Pg-ABA5414   .......... .......... .......... .......... ...IGSEGSS VLDMDSPGTI DSQQ......
Pg-ABA5414   NDQKFQANSS KLQKDDQDLQ LLMMSATKVD CADKENNNEG PSSIGSEGSS VLDMDSPGTI DSQQNIDSIG
Ps-ABK2476
Ps-ABK2476   CMCFGIRYDR IREFG
Ps-ABK2476   CMCFGIRYDR IREFG
Ps-ABK2462   ....S..... .........S S......... ....S..... .......... .TT....... ..........
Ps-ABK2462   .......... .......... .......... .......... .......... .......... ...MSEAASS
Ps-ABK2462   NRNLSDFDFE IEPQQNSANS SHKTTDAPME LSVKSKICQK CAEPLGDLYP TTTKEQEGRC CSIMSEAASS
Os_10g2650   .......... .......... .......... ....S..... .......... .......... ..........
Os_10g2650   .......... .....INLNV K.TEASCSNR SENSSEINLD ISR....... .......... ..........
Os_10g2650   GGGGSSCRQE AASELINLNV KETEASCSNR SENSSEINLD ISRPAPPPPP PPANESPVNR GIPFYASIGR
Zm_4124075   .......... .........S .......... .......... .......... .......... ..........
Zm_4124075   .........I NLNVR..... .......... .......... .......... .......... ..........
Zm_4124075   GRQEAASELI NLNVRETEAS CSENNSSEIN GLDVSRPDPA AGESPAMNSY RGGLPFYASA AARADIHHQL
Sb-XP24672   .......... .......... .......... ..S....... .......... ..S....... ..........
Sb-XP24672   .......... ...INLNVK. TEASCSNRSE NSSEINLDIS R......... .......... ..........
Sb-XP24672   GGGGRHQEAA SELINLNVKE TEASCSNRSE NSSEINLDIS RPPQAPPPAA DDSPTMNSYR GSLPFYASAT
Zm_422699    .......... .......... .......... .........S ..SS....S. .......... ....S.....
Zm_422699    .......... .......... .......INL NVK...ASCS NRSSDENSSE INLDISR... ..........
Zm_422699    SMHLDRRIQI MALKGGGGGR QEAASELINL NVKETEASCS NRSSDENSSE INLDISRPPR AADESPAAMD
Os_03g0745   .......... .......... S......... .......... .......... .......... ..........
Os_03g0745   ..INLNKETE ASCSNRSENS SEINLDISR. .......... .......... .......... ..........
Os_03g0745   ELINLNKETE ASCSNRSENS SEINLDISRT PPPDAAALDT APTAHHHHHG GGGGGGGGGG MIPFYTSIAR
Sb-XP24657   .......... .......... ..S....... ....S..... .......... .......... ..........
Sb-XP24657   ....INLNKE TEASCSNRSE NSSEINLDIS R......... .......... .......... ..........
Sb-XP24657   SSELINLNKE TEASCSNRSE NSSEINLDIS RTPPSEGPMD PPPPHQHPHQ HHHAAGGGLI PFYPSVGGGR
Zm_4113431   .......... .......... ..S....... .......... .......... .......... ..........
Zm_4113431   ....INLNKE TEASCSNRSE NSSEINLDIS R......... .......... .......... ..........
Zm_4113431   SSELINLNKE TEASCSNRSE NSSEINLDIS RAPASEAPLD PTPPPGAGGG GMIPFYPPSV GGRPASAAGV
Zm_413795    .......... ......S..S ...S...... ......S... .......... .......... ..........
Zm_413795    .....INLNK ETEASCSNRS EDSSEINLDI SR........ .......... .......... ..........
Zm_413795    GSSELINLNK ETEASCSNRS EDSSEINLDI SRTPPASEGP MDHPPPPPPH HAAGGGLIPF YPSVGGRHSI
Ta-TaHZI-2   .......... .......S.. .S........ .......... .......... .......... ..........
Ta-TaHZI-2   ...INLNKET EASCSYRSEN SSEINLDISG .......... .......... .......... ..........
Ta-TaHZI-2   SELINLNKET EASCSYRSEN SSEINLDISG TPPSDGPMDA SGPLKHQHGR RGGGMIPFYP SVARPAGVDI
Rc-XP25201   .......... .....S...S .......... .......... .......... .S........ .......S..
```

```
Rc-XP25201   .INLNKETEG SCSNRSENSS DIKLDISRTP AIDSPLSNHP ITS....... ...RPTGVAH LFHNSSSS..
Rc-XP25201   SINLNKETEG SCSNRSENSS DIKLDISRTP AIDSPLSNHP ITSRPLFPSS SSIRPTGVAH LFHNSSSSRP
Cr-ABL6311   .......... .....S.... .......... T........T .......... .......... ...SSS....
Cr-ABL6311   .INLNKETEG SCSNRSENSS DNIKLDIS.. TPAIDSHPQT SRPFF..... RPNTAQQLFQ TTSSS.....
Cr-ABL6311   SINLNKETEG SCSNRSENSS DNIKLDISRT TPAIDSHPQT SRPFFPSSLI RPNTAQQLFQ TTSSSSRPPP
Vv-XP22768   .......... ......S... S......... .......S.. .T........ .......... ....S.....
Vv-XP22768   .INLNIK.TE GSCSNRSENS SDIKLDISRT PAIDSPLSTH PTSR...... .RPAGVVQLF QNSSSR....
Vv-XP22768   SINLNIKETE GSCSNRSENS SDIKLDISRT PAIDSPLSTH PTSRPLFPSS IRPAGVVQLF QNSSSRPDLQ
Vv-CAO1494   .......... ...S...... ..S....... .......... ...S...... ......S... ..........
Vv-CAO1494   ...INLNKE. DQISWSNGSE HSSGINLDIS RTTLINSPVS SQLSSK.... ...RPASITQ LLQGSPRS..
Vv-CAO1494   VGPINLNKET DQISWSNGSE HSSGINLDIS RTTLINSPVS SQLSSKQFFP SSLRPASITQ LLQGSPRSDI
Pt_687113    .......... .SS..S...S .......... .......... .T........ .SS....... ..........
Pt_687113    .INLNKETEG SSSNRSENSS DIKLDISRTP AIDSPLSNHH PTS....... .......... .RPTGVAQLF
Pt_687113    PINLNKETEG SSSNRSENSS DIKLDISRTP AIDSPLSNHH PTSRSFFPSS SSSIRPAGVA IRPTGVAQLF
Pt_696444    .......... .....S...S .......... .......... .T........ .......... ..........
Pt_696444    .INLNKETEG SCSNRSENSS DIKLDISRTP AIDSPLPNHH PTS....... ..RPTGIAQL NQNNSSR...
Pt_696444    SINLNKETEG SCSNRSENSS DIKLDISRTP AIDSPLPNHH PTSRPFFPSS SIRPTGIAQL NQNNSSRPDF
Ha_HAHB1     .......... .......... S......... .......S.. .......... .......... ..........
Ha_HAHB1     .INLNIK.TE GSCSNRSENS SEIKLDISRT PATDSPLSSH HQHQ...... .......... ..NNIVAHQL
Ha_HAHB1     LINLNIKETE GSCSNRSENS SEIKLDISRT PATDSPLSSH HQHQHQPIPN LFPSSNIDRP NSNNIVAHQL
At_ATHB13    .......... .......... .......... .......... .......... .......... ..........
At_ATHB13    .INLNKETEG SCSNRSDNSS DNLRLDIS.. .......... .......... .......... ..TTTTTMQF
At_ATHB13    SINLNKETEG SCSNRSDNSS DNLRLDISTA PPSNDSTLTG GHPPPPQTVG RHFFPPSPAT ATTTTTTMQF
Sd-AAT4051   .......... .......... S......... .......... .......... .......... ..........
Sd-AAT4051   ..INLNKETE GSCSNRSENS SEIKLDISRT PAIDSPLSNH HPNI...... .......... ..........
Sd-AAT4051   ESINLNKETE GSCSNRSENS SEIKLDISRT PAIDSPLSNH HPNISSRPFF PPSMIRSNNN NNSNNGVVVP
At_AtHB23    .......... S......... .......... .......... ........S. .......... ..........
At_AtHB23    .INLNKETEG SCSDRSENIS GDIRPPEI.. .......PTT TTMQFFQNSS SE......EN SISNMFCGID
At_AtHB23    SINLNKETEG SCSDRSENIS GDIRPPEIDS QFALGHPPTT TTMQFFQNSS SEQRMVKEEN SISNMFCGID
Dc-05625.1   .......... ..S..S.... S......S.. S......... .......... .......... ..........
Dc-05625.1   .INLNKE.EG SSSNRSTENS SEIKPDFSR. .......... .......... .......... ..........
Dc-05625.1   SINLNKETEG SSSNRSTENS SEIKPDFSRT SPAIDSHPHK LTSIPLFPPP NNNNKSSFFR LQQPAAFHMS
Dc-05623.1   .......... S.S...S... SY......S. .......... .......... .......... ..........
Dc-05623.1   .INLNKE... .......... .......... .......... .......... .......... ..........
Dc-05623.1   SINLNKETEG SSSNNRSTEN SYEIKPDFSR TPPAIDSYPQ KITSIPFFPP NTSNFIRLQQ PALHLSRPSD
Gm-ACU2401   .......... .....S...S .......... ...S...... .......... .......... ..........
Gm-ACU2401   .INLNKETEG SCSNRSENSS DIKLDISRTP AIDSPHFTHQ QQP....... ...RPAGVVQ LFQTSSRP..
Gm-ACU2401   SINLNKETEG SCSNRSENSS DIKLDISRTP AIDSPHFTHQ QQPSRPFFPP FSVRPAGVVQ LFQTSSRPEL
Gm-ACU2089   .......... .....S...S .......... ...S..S... .......... .......... ..........
Gm-ACU2089   .INLNKETEG SCSNRSENSS DIKLDISRTP AIDSPHSTHQ QSR....... .RPAGVAQLF QTSSRP....
Gm-ACU2089   SINLNKETEG SCSNRSENSS DIKLDISRTP AIDSPHSTHQ QSRPLFPPSS ARPAGVAQLF QTSSRPDLPS
Mt-ACJ8462   .......... .SS..S...S ......S... ....S..S.. .......... .......... ..........
Mt-ACJ8462   .INLNKETEG SSSNRSENSS DIKLDIST.T QAIDSPLSTQ QTSI...... ..PAGVPHQL FQTNSRQ...
Mt-ACJ8462   SINLNKETEG SSSNRSENSS DIKLDISTRT QAIDSPLSTQ QTSINLFPSS SRPAGVPHQL FQTNSRQDIQ
Gm-ACU1869   .......... .SS..S...S .......... .......... .........S .......... ..T.......
Gm-ACU1869   .INLNKETEG SSSNRSENSS EIKLDISRTP AIDSPLSTQQ SNS....... .RPTGVAQLF QTTPRP...C
Gm-ACU1869   SINLNKETEG SSSNRSENSS EIKLDISRTP AIDSPLSTQQ SNSRTLFPSS ARPTGVAQLF QTTPRPEIQC
Gm-ACU2100   .......... .SS..S...S .......... .......... .......... S......... ...T......
Gm-ACU2100   .INLNKETEG SSSNRSENSS EIKLDISRTP AIDSPLSTQQ NNN....... ..RPTGVAQL FQTTPRP...
Gm-ACU2100   SINLNKETEG SSSNRSENSS EIKLDISRTP AIDSPLSTQQ NNNNRTLFPS SARPTGVAQL FQTTPRPEIQ
Rc-XP25174   .......... S......... .......... ....S..... .......... ....S..... ......SS..
Rc-XP25174   VNLKKE.DQG SWSNGSENSC DANNLDIS.T PVATSPVSSQ LTTK...... .RPTSMTQLL QTSSRP....
Rc-XP25174   VNLKKETDQG SWSNGSENSC DANNLDISRT PVATSPVSSQ LTTKHLFSTS IRPTSMTQLL QTSSRPSSDH
Pt_736296    .........S .......... .......... .......... .......... .......... ..........
Pt_736296    VNLKKETEGS WSNGSENSCD VNLDISRNPV ASSPVSSQLT TK.......R PATITQLLQG SSRS......
Pt_736296    VNLKKETEGS WSNGSENSCD VNLDISRNPV ASSPVSSQLT TKHLFSAPIR PATITQLLQG SSRSDLQCLK
Gm-ACU1987   .......... ...S...... ..S....... ......S... .......... .S........ ..........
Gm-ACU1987   ....ISLKKE TEGSWSNGSN NSSEINLDLS RTPVMNSPVS SSLNGK.... ...KPTSITQ LLQCSSRS..
Gm-ACU1987   TGTVISLKKE TEGSWSNGSN NSSEINLDLS RTPVMNSPVS SSLNGKTLLP TSLKPTSITQ LLQCSSRSDL
Gh-ABO4774   .......... .....S.... .......... ....S..... ........S. .......... ..........
Gh-ABO4774   .......... .......... .......... .......... ....RPTSMT QLLQGSSRP. ..CVKLDQVV
Gh-ABO4774   TSCIKKENDC SWSYGSDKNS CDVNLDISRT PLTSSSKHLF PPSVRPTSMT QLLQGSSRPD LQCVKLDQVV
At_ATHB20    .......... S......... .S........ .......... ......S... S......... ..........
At_ATHB20    .......... .......... .......... .......... .......... .......... .....EESLC
At_ATHB20    GNIVKREAEA SWSNNGSTEN SSDINLEMPR ETITTHVNTI KDLFPSSIRS SAHDDDHHQN HEIVQEESLC
At_ATHB3     .......... .......... .......... .......... ..S....... .......... ..........
At_ATHB3     .......... .......... .......... .......... .......... .......... ....DQGFCN
At_ATHB3     SAKIKREFAE ASWSNNGSTE NNHNNNSSDA NHVSMIKDLF PSSIRSATAT TTSTHIDHQI VQDQDQGFCN
Sm_18196
Sm_18196     .
Sm_18196
Sm_81609
Sm_81609     .
Sm_81609     FAF
Cr-CRHB6
Cr-CRHB6     .......... .......... ....
Cr-CRHB6     DRNGMSEPKG GTAEQWQIRG NVNQ
Cr-CRHB11    .......... .S........ .......... .......... S......... .......... ..........
Cr-CRHB11    .......... .......... .......... .......... .......... .......... ..........
```

```
Cr-CRHB11     VCFKHAMSLL WSGYEESHVN IYSMTDSITN AYFHCLTRRH SSLILLHFSS QHTLIGFGGI CDAWSPTFRY
Cr-CRHB4      .......... ......S.S. ..ST...... .......... .......... .......... ..........
Cr-CRHB4      .......... .......... .......... .......... AKLDAQSEIL ESLTLSCLKS QEAIVAFVEQ
Cr-CRHB4      DVPVDIDAKV ASPDLDSDSD LESTVKGEKQ QNLKSEIGIP AKLDAQSEIL ESLTLSCLKS QEAIVAFVEQ
Sm_18217      .......... .......... .......... .......... .......... .......... .........S
Sm_18217      .......... .......... .......... .......... .......... .......... ..........
Sm_18217      DDDDNDEDRN EEDMDEDSGS AAAAATTTTD KPPKPLLLVA TMAKNNKSSG VVEDVDDQVC TTPDSPTVVS
Sm_19476      .......... .......... .......... .......... .......... ...S...... ..........
Sm_19476      .......... .......... .......... .......... .......... .......... ..........
Sm_19476      ASGDKAIVNS VEHKPEAFKG EEVTVMVTTG KNLAVVEDMD VARPPGKGGE KEGSPCSDGY TSEILDVDFP
Ps-ABK2572    .......S.. .........T ......S... .......... .......... .......... ..........
Ps-ABK2572    .......... ......TTTT TTEEMQSTSP S......... .......... .......... ..........
Ps-ABK2572    NTLITAKSEE ENGLQKTTTT TTEEMQSTSP SFCSDADSNR KLLLSSPSCN IQAAACKTDE ENEEENLQAL
Os_03g1021    .......... .......... ......S..S .......... .......... .......... ..Y.......
Os_03g1021    .......... .......... .......... .......... .......... .......... ..........
Os_03g1021    EEEKAKLAAV AAATTGGGGG GGGGSSSPTS SSFSTVTYHP ALAGQFGVEA AAEEADLTYM SEYAYNSYML
Zm_hox12      .......... .......... ....S.SSS. .......... .......... .......... ..Y.......
Zm_hox12      .......... .......... .......... .......... .......... .......... ..........
Zm_hox12      EEEKTKLVAA AAAAAGGAAG AGSSSPSSSS FSTVTHHPAA ALQVGQFGVE PEEAADLAYM TEYAYNSYMN
Zm_480132     .......... ........S. SSSSS..S.. .......... .......... .......... ..Y.......
Zm_480132     .......... .......... .......... TTTQQHHTAA LVGQFGVEQE EEAADLTTY. ..........
Zm_480132     EEEKTKLIAA AAGGAGSSSP SSSSSSFSTV TTTQQHHTAA LVGQFGVEQE EEAADLTTYM SEYAYNSYMN
Zm_433132     .......... .....S.... .......... .S........ .......... .....Y.Y.. .S........
Zm_433132     .......... .......... .......... .......... .......... .......... ..........
Zm_433132     EEELARFRSV GSHAISGDGG DAMMGRAVCS GSPSSSFSTG TCQQPGDDDL LYFPDYAYAD NSVVDEWFRM
Zm_433210     .......... .......... .......... .......... ..S....... ....
Zm_433210     .......... .......... .......... .......... .......... ....
Zm_433210     EEELTRFRSA GNHAQPGGGG GGGDHLGDDD LLYVPDYAYA DSSVVEWFSL YGLM
Os_07g3932    .......... ..S...S... .......... .....S.... .........S .......... ..........
Os_07g3932    .......... .......... .......... .......... .......... .......... ..........
Os_07g3932    EEEVRRLRSA AGSHTASGEG GDIMGLGGSG ACVAGSPSSS FSTGTCQPPS FGGGDHLGDD DLVYVPEYGG
Pt_655260     .......S.. .......S.. S..S...... .........Y .......... .......... ....
Pt_655260     .......... .......... .......... .......... .......... .......... ....
Pt_655260     EKEIQRLSDR IDGVSTNSPS SSLSMAMDPP FLGEFAMEGY EDAAFYMPPE NNYIPGMEWI NLYM
Pt_703426     .......S.. S..S...... .......... .......... .......... .......
Pt_703426     .......... .......... .......... .......... .......... .......
Pt_703426     SDRADRVSTN SPSSSLSMAI EHPFLGEFAV LEGYGDAFYM PPENNYIPGM EWISQYM
At_ATHB53     .......S.. .......... S..S...... .......... .......... .......... .....
At_ATHB53     .......... .......... .......... .......... .......... .......... .....
At_ATHB53     QSEIRKLSER LEEMPTNSSS SSLSVEANNA PTDFELAPET NYNIPFYMLD NNYLQSMEYW DGLYV
At_ATHB40     .......... .......S.. S...S..... .......... .......... ......SY.. .........
At_ATHB40     .......... ..GGSSNSPI SSSVSVE... .......... .......... .......... .........
At_ATHB40     EREIQRLAER VEGGSSNSPI SSSVSVEANE TPFFGDYKVG DDGDDYDHLF YPVPENSYID EAEWMSLYI
At_ATHB21     .......... S..S...... .......... .......... ........Y. ..........
At_ATHB21     .......... .......... .......... .......... .......... ..........
At_ATHB21     AKRVEGTLSN SPISSSVTIE ANHTTPFFGD YDIGFDGEAD ENLLYSPDYI DGLDWMSQFM
Gm-ACU198B    .......... ........S. .......... .......... .......... .......... ..........
Gm-ACU198B    .......... .......... .......... ...TYHHHHE CWQSGGEVMQ VEELYTYFVG AN........
Gm-ACU198B    NSVKGGDHNN NNACEFSTSF EEGGSSGVVL DDATYHHHHE CWQSGGEVMQ VEELYTYFVG ANYGTACMRK
Pt_98386      .......... .....S.... ....S..... .......... Y......... .
Pt_98386      .......... .......... .......... .......... .......... .
Pt_98386      NQETAAQPAV NFSCNSSCDD RGSSSLHEGV NGEVLQLEEL YACLYGAGGG F
Pt_568845     .......... ....SS.... ...S...... .........Y .......... ..
Pt_568845     .......... .......... .......... .......... .......... ..
Pt_568845     NQGPAQPPVN VSSNSSCDEG GSSSLHEEAN GEVLQLEELY ACLYGAGGST WG
At_ATHB52     ....S..... .......... .........Y .......... ...S..
At_ATHB52     .......... .......... .......... .......... ......
At_ATHB52     TNQDSPVDNS NLGSCDEDHD DQVVVFDELY ACFVSNGHGS SSTSWV
Pt_594622     .......... .S.SS..... .......... .......... .......... ..........
Pt_594622     .......... .......... SSSPGNMICN W......... .......... ..........
Pt_594622     PTSSAATLPS VSGSSDEQAN SSSPGNMICN WGDAGNDDMF PVEELYTCLI GSDTQLWPLN
Pt_unknon     .......... ....S..... .......... .......... .....Y.... ..........
Pt_unknon     .......... .......... STSPGNMICN W......... .......... ..........
Pt_unknon     STTSASALAS VSGYSDEQAN STSPGNMICN WRDAGNDEIF PVEELYTCLT GSGTQLWPLS
```

```
             71
Zm_479999    .......... ........S. .......... .......... .......... .......... ..S.......
Zm_479999    TPDLWDTWPL LEWNATL... .......... .......... .......... .......... ..........
Zm_479999    TPDLWDTWPL LEWNATLLSV RRRRQLAHLS RTFVVVVPAP KDNGYAPSED DVAFDGAGTE SLSHTGDDPP
Os_02g4333   .......... .......... .......
Os_02g4333   .......FCG MPDLWEIWPM VEWNAVA
Os_02g4333   GGQLPDPFCG MPDLWEIWPM VEWNAVA
Sb-XP24528   .......... .......... ........
Sb-XP24528   ........LC ATPDLWDTWP LLEWNAVA
Sb-XP24528   YGEGLHDPLC ATPDLWDTWP LLEWNAVA
Zm_470295    .........S ..S....... .........Y .......... .........
Zm_470295    .......... .......... .........Y CAMPELWESW PLVEWNAVA
Zm_470295    GSCATPADVS VESECDDHRL DYGDGFPDSY CAMPELWESW PLVEWNAVA
Sb-XP24468   .......... .....Y.... .......... .....
Sb-XP24468   .......... .....YCAMP ELWEPWPLVE WNAVA
Sb-XP24468   HHHHHLDYDD GFPESYCAMP ELWEPWPLVE WNAVA
Zm_433493    .......... .....Y.... .......... .....
Zm_433493    .......... .....YCAMP ELWEPWPLVE WNAVA
Zm_433493    CDDQHLHYDD EFPESYCAMP ELWEPWPLVE WNAVA
Os_04g4581   .......... .......... .......... ...
Os_04g4581   .......... ...FCATPEL WEPWPLVEWN AVA
Os_04g4581   DQLDYDEGLF PESFCATPEL WEPWPLVEWN AVA
Zm_4492      .......... .......... .......... .......... ......
Zm_4492      .......... .......... ........AE QTCSGSQWWE LES...
Zm_4492      TAGLLTSSGP PEHQPFQFHS GCCWPSSSAE QTCSGSQWWE LESLSE
Sb-XP24627   .......... .......... .S........ .......... .
Sb-XP24627   .......... .......... ...TEQTCSS SQWWEFES.. .
Sb-XP24627   SSSGPPEHQP FQFHSSSCWP SSTTEQTCSS SQWWEFESLS E
Os_09g3591   .......... .......... .......... ...S...... ..........
Os_09g3591   .......... AGFTSSEPAA DHQSFN.... ....TEQTCS STPWWEFES.
Os_09g3591   QHHPPPPHAG AGFTSSEPAA DHQSFNFHSS WPSSTEQTCS STPWWEFESE
Zm_4496      .......... .........S .......... ........
Zm_4496      .......... .......... .TEQTCSSSQ WWEFES...
Zm_4496      TSSGPPEHQP FQFHXGCWPS STEQTCSSSQ WWEFESLSE
Vv-XP22629   .......... S......... .........S ........
Vv-XP22629   MVEPVDGCLT SPEDWGSWDS ...FDQSSGS YQWWDFWG
Vv-XP22629   MVEPVDGCLT SPEDWGSWDS ENVFDQSSGS YQWWDFWG
Vv-CAN7896   .......... ..........
Vv-CAN7896   MVEPVDGCLT FTGRLGQLGL
Vv-CAN7896   MVEPVDGCLT FTGRLGQLGL
Rc-XP25299   .......... .....S.... .......... .......... .......... .......... ..........
Rc-XP25299   .....IVEPA DGSLTSQEDW GSLDS..... .......... .......... .......... ..........
Rc-XP25299   ANLMSIVEPA DGSLTSQEDW GSLDSDAFLI NQTLVVSGGI SGLDCKINEL RTQGAKHGLS MQILFLVTGG
Mt_MTHB1     .......... S......... .......... ........
Mt_MTHB1     FAEHADGSLT SPEDWSAFES ...LGQSSCD YQWWDFWS
Mt_MTHB1     FAEHADGSLT SPEDWSAFES NDLLGQSSCD YQWWDFWS
Gm-ACU2443
Gm-ACU2443
Gm-ACU2443
Pt_HB7       .......... S......... ......S... Y.......
Pt_HB7       MVEPADGSLT SQEDWGSLDS ...FDQSSSD YQWWDFWA
Pt_HB7       MVEPADGSLT SQEDWGSLDS DRLFDQSSSD YQWWDFWA
Pt_731421    .......... S......... .......... ........
Pt_731421    MVEPAEGSLT SQEDWGSIDS ...FDQSSSG YQWWDFWA
Pt_731421    MVEPAEGSLT SQEDWGSIDS DGLFDQSSSG YQWWDFWA
At_ATHB12    .......... .......... .......... ......
At_ATHB12    ADDSCLTSSE NWGGFNS... .DQSSSNYPN WWEFWS
At_ATHB12    ADDSCLTSSE NWGGFNSDSL LDQSSSNYPN WWEFWS
At_ATHB7     .......... .......... .......S.. .......... .......... .......
At_ATHB7     .......... .......IVE PADSCLTSSD DWRGFKS... ..LDQSSNNY PWRDFWS
At_ATHB7     KREYFGGFEE EPDHLMNIVE PADSCLTSSD DWRGFKSDTT TLLDQSSNNY PWRDFWS
Zm_hox6
Zm_hox6
Zm_hox6
Vv-XP22800   ........
Vv-XP22800   SQWWELWS
Vv-XP22800   SQWWELWS
Pt_548258    .......... .
Pt_548258    DNSSQWWEFW S
Pt_548258    DNSSQWWEFW S
Pt_343725    .......... .......... S........
Pt_343725    .......... ....LDESRC SSQWWELLS
Pt_343725    MTSSDWQSFQ SNCFLDESRC SSQWWELLS
Ha_HAHB4
Ha_HAHB4
Ha_HAHB4
Vv-XP22715   .......... .......... .......... .
Vv-XP22715   LESTGKWFGF AS...HDQSC TISQLWDFWS .
Vv-XP22715   LESTGKWFGF ASGGFHDQSC TISQLWDFWS S
```

```
Ha_HAHB11
Ha_HAHB11
Ha_HAHB11
Pt_HBLZ      .......... .......... .......... .......... .......... .......... .........
Pt_HBLZ      .......... .......... .......... .......... .......... .......... .........
Pt_HBLZ      FRDLRGNREL VKPVDRRLYC LALVRKGVGS NPTAVNILYV FPYIFLAPAM FNMGYSWVKL LVNGDKFFW
Sb-XP24459
Sb-XP24459
Sb-XP24459
Os_08g3758   .......... .......... .......... .......... .......... .......
Os_08g3758   AALLHHAEVF FHGQLLKVDD DEAAFLGDD. AACGGFFADE HLPSLPWWAE P......
Os_08g3758   AALLHHAEVF FHGQLLKVDD DEAAFLGDDG AACGGFFADE HLPSLPWWAE PTEQWTT
Sb-XP24603   .......... .......... .......... .......... .......... ...
Sb-XP24603   AANHGGVFFH GSFLKVEEDE TGLLDDDEPC GGFFAVEQPP PMAWWTEP.. ...
Sb-XP24603   AANHGGVFFH GSFLKVEEDE TGLLDDDEPC GGFFAVEQPP PMAWWTEPTE HWN
Zm_4112646   .......... .......... .......... .......... ....
Zm_4112646   HGSFLKVEED ETGLLDDDEP CGGFFSVEQP PPMAWWTEP. ....
Zm_4112646   HGSFLKVEED ETGLLDDDEP CGGFFSVEQP PPMAWWTEPT EHWN
Os_09g2946   .....S.... .......... .......... .......... .......... .......
Os_09g2946   .AAAASHEEV FFHGNFLKVE EDETGFLDDD EPCGGFFADD QPPPLSSWWA E......
Os_09g2946   AAAAASHEEV FFHGNFLKVE EDETGFLDDD EPCGGFFADD QPPPLSSWWA EPTEHWN
Zm_469357    .......... .......... .......... .......... .......... ...
Zm_469357    AANHGGVFFH GSFLKVEEDE TGLLDDDGPC GGFFAVEQPP AMAWWNEP.. ...
Zm_469357    AANHGGVFFH GSFLKVEEDE TGLLDDDGPC GGFFAVEQPP AMAWWNEPAE HWN
Zm_469358    .......... .......... .......... .......... .......... ...
Zm_469358    AANHGGVFFH GSFLKVEEDE TGLLDDDGPC GGFFAVEQPP AMAWWNEP.. ...
Zm_469358    AANHGGVFFH GSFLKVEEDE TGLLDDDGPC GGFFAVEQPP AMAWWNEPAE HWN
Ta-TaHZI-1   .......... .......... .....
Ta-TaHZI-1   EEPCGGFFAD EPPLAWWTEP TD...
Ta-TaHZI-1   EEPCGGFFAD EPPLAWWTEP TDPWK
Zm_483405    .......... S.....S... .......... .......... .......... ....
Zm_483405
Zm_483405    FTSFLTSSFP SVYHGDSHLD QEADGFFSAA AAGDGFFAEE QSAGIGSWYG GEGW
Os_10g2309   .......... .......... ....S..... .......... .......... .......... ..........
Os_10g2309   .......... .......... .......... .......... ........GG AGFFAGDEHA GGLSWYGAE.
Os_10g2309   FTSFFAPSTT LTSSLSFPSM FHASSHFDGH QELLVGGGGA GAVADADLGG AGFFAGDEHA GGLSWYGAEG
Zm_4102187   .......... .......... .......... .......... .......... .......... ..........
Zm_4102187   .......... .......... .......... .......... .......... .........G GAAGFFSEDH
Zm_4102187   FLDTSVAPTG CSSPPVFVET KWQQHGPTQY PFPFDSYKAC AGYGFTEEWL ASSDLIGSDG GAAGFFSEDH
Zm_4118271   .......... .......... .......... .......... .......... .......... ..........
Zm_4118271   .......... .......... .......... .......... .......... .........G GAAGFFSEDH
Zm_4118271   FLDTSVAPTG CSSPPVFVET KWQQHGPTQY PFPFDSYKAC AGYGFTEEWL ASSDLIGSDG GAAGFFSEDH
Os_03g0896   .......... .........S .......... .......... .......... .......... ..........
Os_03g0896   .......... .....YSDDS CKGGVYGFTE EWLAACSGEM ..NDAAGFFS DEHASNLNFG WCA.......
Os_03g0896   CSSSLPALES KWHGPYSDDS CKGGVYGFTE EWLAACSGEM AGNDAAGFFS DEHASNLNFG WCASGNEGWE
Cp-CPHB-4    ...S....S. ...S..S... .......... .......... ...S...... S......... .......
Cp-CPHB-4    .......... .......... ...YKPQFVK IEEQHNLF.. ...SCTTTFF SDEQAPTLNW YTPD...
Cp-CPHB-4    PPSSAKIASE KNGSDESCKR AAAYKPQFVK IEEQHNLFRE EDESCTTTFF SDEQAPTLNW YTPDEWN
At_ATHB5     .......... .......... .......... .......... ........S. .......... ..........
At_ATHB5     SDSSAVLNE. .......... .......... ...CFSQFVK MEEHEDLF.. .......... ..........
At_ATHB5     SDSSAVLNEE YSPNTVEAAG AVAATTVEMS TMGCFSQFVK MEEHEDLFSG EEACKLFADN EQWYCSDQWN
Sl-SlHDL1    .......... .......... ........S. S......S.. ...S...... .......... ..........
Sl-SlHDL1    .......... ....IQNEQQ QLLLMSPRS. .......... .........Y QAQYVKLEEH NFFSEEACNF
Sl-SlHDL1    NSPNASNCSS DGGIIQNEQQ QLLLMSPRSP SLGFNRFSFT ESRSVSDNVY QAQYVKLEEH NFFSEEACNF
Ze-18171.1   .......... .......... .S...
Ze-18171.1   NFFSDDQAPT LQWYCQD... .....
Ze-18171.1   NFFSDDQAPT LQWYCQDQWN LSEEN
Nt-Hfi22     .......S.S .......... .......... .......... .......... .......... S.........
Nt-Hfi22     ...KDGSSDS DSSAILNE.. .......... .......... .......... .......... ..........
Nt-Hfi22     DNFKDGSSDS DSSAILNEDN SPNAAAISSS GAFLISTNGN GNGNGSSTSL NFCFQFTESS SKSNLGDGQK
Brs-hb-6     .........S .......... .......... .......... ....T..... ..S....... ..........
Brs-hb-6     ...AGSSDSS DSRAVLNE.. .......... ....GFLQFV KMEQTEDHD. ....EEACGF FSEEQPPSLH
Brs-hb-6     VAAAGSSDSS DSRAVLNEES SSNVTAGPVT VPQGGFLQFV KMEQTEDHDD FLSGEEACGF FSEEQPPSLH
Bn-AAR0493   .......... S......... .......... .......... .....T.... ...S...... ..........
Bn-AAR0493   ....AGSSDS SDSSAVLNE. .......... .....SFLQF VKMEQTEDHD .....EEACG FFSDEQPPSL
Bn-AAR0493   SVAAAGSSDS SDSSAVLNEE SSSNVTAAPA TVPRGSFLQF VKMEQTEDHD DFLSGEEACG FFSDEQPPSL
At_ATHB6     .........S .......... .......... .......... .....T.... ...S...... ..........
At_ATHB6     ...AGSSDSS DSSALLNE.. .......... .....NFFQF VKMEQTEDHE .....EEACE FFSDEQPPSL
At_ATHB6     AAAAGSSDSS DSSALLNEES SSNVTVAAPV TVPGGNFFQF VKMEQTEDHE DFLSGEEACE FFSDEQPPSL
At_ATHB16    .......... ..T....... .......... .T.......S .......... ......
At_ATHB16    LND....... .......... .......... .......... .EEACGFFSD EQPPSLHWYS AS....
At_ATHB16    LNDETSSDNG RLTPPVTVTG GSFLQFVKTE QTEDHEDFLS GEEACGFFSD EQPPSLHWYS ASDHWT
Pt_HD56      S......... .......... .......... .......... ......S... .......... ..........
Pt_HD56      SDSSAILNE. .......... .......... .......... .......... .........Y QTQFVKLEEH
Pt_HD56      SDSSAILNED NSPNPAISSS GILQSQLMMS PPPSSSLRFN CSASSSSPSS MNCFQFSKSY QTQFVKLEEH
Pt_70493     .......... .......... .......... ......S... .......S.S .......... ..........
Pt_70493     DSDSSAILNE .......... .......... .......... .......... .......... YQTQFVKLEE
Pt_70493     DSDSSAILNE DNSPNPAISS SGILQSQLMM SPPPSSSLKF NCSTSSSSPS TMNSFQFSKT YQTQFVKLEE
Vv-XP22660   S......... .......... .......... .......... ...S...... .......S.. ..........
```

```
Vv-XP22660  SDSSAILNE.  ..........  ..........  ..........  ..........  ..........  .........Y
Vv-XP22660  SDSSAILNED  NSPNAAISSS  GVLQNNHHHH  LLMSPPSSSL  RFNSSSSSSI  NCFHFSDSSR  PTLGNTQKAY
Mt-ACJ8504  .S..S.....  ..........  .S........  ..........  ..........  ..........  ..........
Mt-ACJ8504  DSDSSAISSS  ..........  ..........  ...QKSYHVK  MEEHNFLS.D  EACNFFSDEQ  APTLQWYCPD
Mt-ACJ8504  DSDSSAISSS  GVLQSQQHLL  LSPESSSMNC  FQYQKSYHVK  MEEHNFLSAD  EACNFFSDEQ  APTLQWYCPD
Pv-HDZ1     ..........  ..........  ......S...  ..........  .Y........  ..........  ..........
Pv-HDZ1     ..........  ..........  ..........  .......FPT  QYVKMEEHNF  LS.DEACNFF  SDEQAPTLQW
Pv-HDZ1     NNDNSPNNAT  ISSSGAMQSQ  SFLSSSSPMN  CFQFQKQFPT  QYVKMEEHNF  LSADEACNFF  SDEQAPTLQW
Gm-184277-  ......S...  ..........  ..........  ..........  ..........  ..........  ..........
Gm-184277-  E.........  ..........  ..........  ..........  ......TTTQ  YVKMEEHNFL  S.DEACNFFS
Gm-184277-  EENNNHSPNN  NAAISSCGVL  QSHGLLLSPS  SSSMNNCFQF  QKQYQTTTTQ  YVKMEEHNFL  SADEACNFFS
Sl-CAB6711  ..........  ...S....S.  ...Y......  ..........  ..........  ..........  .......
Sl-CAB6711  ..........  ..........  ..........  ..........  .QQQAVGRIE  EAKWFY....  .......
Sl-CAB6711  SSSTYTQLMD  PRASNSTSSS  MRAYNNNNNN  NDDNNNSNNQ  QQQQAVGRIE  EAKWFYNRGF  LQHLLC
Vv-CAO6167  ...S......  ..........  ........
Vv-CAO6167  SFFS.EEPCN  FFWGDQAPSL  HWYFPD.
Vv-CAO6167  SFFSAEEPCN  FFWGDQAPSL  HWYFPDQ
Vv-XP22857  ..........  ...S......  ..........  .......S..  ..........  ..........  .
Vv-XP22857  ..........  ..........  ...FQHQFGR  MEEQSFFS.E  EPCNFFWGDQ  APSLHWYFPD  .
Vv-XP22857  SSLRLNCASL  SSPSPMNWFP  SKAFQHQFGR  MEEQSFFSAE  EPCNFFWGDQ  APSLHWYFPD  Q
Vv-CAN8361
Vv-CAN8361
Vv-CAN8361
Pt_unknown  .......S..  ..........  .
Pt_unknown  ..........  ..........  .
Pt_unknown  HCQDCAKSSS  RGTNPQGPED  V
Pt_HB       ..........  ..........  ..........  ..........  ..........
Pt_HB       ..........  ..........  ..........  ..........  ..........
Pt_HB       YLRCSIATKH  LQPGREQWFY  HLLQYKKSYY  LYMNLESNFA  ISNTTLDNND
Rc-XP25139  ..........  ..........  ..........  ..........
Rc-XP25139  .YQPHLVKLE  EHHSLF..EE  SCNFLSVDQA  PTLHWHFTE.
Rc-XP25139  VYQPHLVKLE  EHHSLFNIEE  SCNFLSVDQA  PTLHWHFTEQ
Os_02g4970  ..........  ......S...  ..........  ..........  ....S.....  ..S.......  ..........
Os_02g4970  QLVVGCGR..  ..........  ..........  ..........  ..........  ..........  ..........
Os_02g4970  QLVVGCGRQH  LAAVDSSVES  YFPGGDEYHD  CVMGPMDHAA  GGIQSEEDDG  AGSDEGCSYY  ADDAGVLFAD
Zm_4134425  .....S....  .......Y..  ..........  ..........  ...S......  ..........  ..........
Zm_4134425  A.........  ..........  ..........  ..........  ....DEGCSY  YADEAAAAAA  AFFAG.....
Zm_4134425  AAVDSSVESY  FPGGEDHYHD  CGTMGPVNHG  AGGGIQSDDD  GAGSDEGCSY  YADEAAAAAA  AFFAGHATHH
Zm_459693
Zm_459693
Zm_459693
Zm_459694
Zm_459694
Zm_459694
Os_08g3208  ..........  ...S......  ..........  .......SS.  ..........  ..........  ..........
Os_08g3208  ..........  ..........  ..........  ..........  ..........  ..........  ..........
Os_08g3208  VVFDVTEGAN  DRLSCESAYF  ADAAEAYERD  CAGHYALSSE  EEDGGAVSDE  GCSFDLPDAA  AAAAAMFGAA
Zm_4124691
Zm_4124691
Zm_4124691
Os_09g2118  .......S..  SSS.......  .....Y....  ..........  ..........  ..........  .........
Os_09g2118  ..........  ....DDCGGA  GSDDDYPSSS  VLLPV.....  ..........  ....DEEAPL  NSWEW...
Os_09g2118  SYFAGARSPP  SSSEDDCGGA  GSDDDYPSSS  VLLPVDATLV  GDAFEHAVAA  TVAADEEAPL  NSWEWFWN
Lj-BAG5005  .......S..  ........S.  ......S...  ..........  ..........
Lj-BAG5005  ..........  ..........  ..........  ....ENGEGL  NWWGN.....
Lj-BAG5005  PAAERVQSEE  EDGSDDGRSY  LDVFVISETE  QQNHENGEGL  NWWGNMYYVA
Pv-HDZ3     ......S...  .......S..  .....S....  ..........  .........
Pv-HDZ3     ..........  ..........  ..........  ...EEGEALV  WWTN.....
Pv-HDZ3     PVERIQSEEE  DGSDDGRSYM  DVFVVSETEN  QNHEEGEALV  WWTNMYYVE
Gm-ACU1808  .......S..  ........S.  ......S...  ..........  ..........
Gm-ACU1808  ..........  ..........  ..........  ....EEGETL  GWWTN.....
Gm-ACU1808  GPVERVQSEE  EDGSDDGRSY  LDVFVVSETE  HQNHEEGETL  GWWTNMYYVG
Rc-XP25173
Rc-XP25173
Rc-XP25173
Vv-XP22788  ...S......  ..........  ..........  .........
Vv-XP22788  ..........  ..........  ..........  .........
Vv-XP22788  GVQSEEDDGS  DDGQSYFSDA  LVLPEHHHEE  GEALLWVWP
At_ATHB1    S.........  ........S.  ..........  .....
At_ATHB1    ..........  ..........  ..........  .....
At_ATHB1    SDHDNDRSCF  ADVFVPTTSP  SHDHHGESLA  FWGWP
At_ATHB54   ..........  ..........  ..........  ..........  .......S.S  .S........  ........S.
At_ATHB54   YDPTLTASNL  RQEPLKVTCA  DQMTQIQISD  VTEPAS....  ..........  ..........  ..........
At_ATHB54   YDPTLTASNL  RQEPLKVTCA  DQMTQIQISD  VTEPASSAHK  KIEVTQRSSS  MSRKRDKPYT  NRHTPARISK
Ps-ABR1622  ..........  ..........  S.........  S.........  ..........  ..........  ..........
Ps-ABR1622  PHHIDSGH..  ..........  ..........  ..........  ..........  ..........  ..........
Ps-ABR1622  PHHIDSGHSS  VVGYVTSTHI  SPFEADLSDF  SHVDEEEEEG  GISEKLLLPQ  DCHMKMECIL  FPEAPTTSCT
Sl-CAA6441  ..........  S.SS......  ..........  .........Y  .......S..  ..........  ....
Sl-CAA6441  DSARAFETDQ  SDSSQDDDEN  FSKNMLSTAN  LLGKDA...Y  PATSSNLSYF  GFPVEDQGFG  FWTY
```

```
Sl-CAA6441  DSARAFETDQ SDSSQDDDEN FSKNMLSTAN LLGKDADDDY PATSSNLSYF GFPVEDQGFG FWTY
Mt-ACJ8510  .Y........ ...Y.....Y ....S..... .......... ......Y... .......... ..........
Mt-ACJ8510  RYTDGVL... .......... .......... .......... ......YSDP PHNSTSYGFQ EEDHHQALWP
Mt-ACJ8510  RYTDGVLETC DSSYVFEPEY QSDLSQDEED HNLLPPYIFT KLEDVNYSDP PHNSTSYGFQ EEDHHQALWP
Gm-AAX9867  .......... ...S..S... .......... .......... .Y........ .......... ......
Gm-AAX9867  ETGDSSYVFE PDRSDVSQDE EDNLSKTLLP HYIFPKLED. .YSDPPHGSC NFGIPEEDQA IWSWPY
Gm-AAX9867  ETGDSSYVFE PDRSDVSQDE EDNLSKTLLP HYIFPKLEDV DYSDPPHGSC NFGIPEEDQA IWSWPY
Dc-05624.1  .......... ...S..S... .......... ......S... ..SY...... .......... ..........
Dc-05624.1  EPVDRSYVFE PAQSDISQDE EDDMGNNLFL PSYHVFSKT. ...YSDQPSN SSYFGFPVED HTFGFWGT..
Dc-05624.1  EPVDRSYVFE PAQSDISQDE EDDMGNNLFL PSYHVFSKTE DGSYSDQPSN SSYFGFPVED HTFGFWGTEL
Rc-XP25115  .......... ......S... .......... .......... .Y.....S.. .........
Rc-XP25115  EAGDSSYVFE PDQSDVSQDE EDNLSKSLLP PYVFPKLEE. .......... .........
Rc-XP25115  EAGDSSYVFE PDQSDVSQDE EDNLSKSLLP PYVFPKLEEA DYSDPPASFE DHAFWCWSY
Pt_88244    .......... ......S... .......... .......... .Y.....S.. .........
Pt_88244    EAGDSSHVFE PDQSDLSQDE EDNLSKSLLP PYVFPKLED. .......... .........
Pt_88244    EAGDSSHVFE PDQSDLSQDE EDNLSKSLLP PYVFPKLEDG DYSDPPASFE DHAFWCWSY
Vv-CAO6250  .......... ........
Vv-CAO6250  PCSFGFPVED HAFWSWSY
Vv-CAO6250  PCSFGFPVED HAFWSWSY
Vv-CAN8396  .......... ......S... .......... .......... ..Y....... .......... .......
Vv-CAN8396  EPGDSSYVFE ADQSDVSQDE EDNFSKSLLP PSYIFPKLE. ..YPDPPTNP CSFGFPVEDH AFWSWSY
Vv-CAN8396  EPGDSSYVFE ADQSDVSQDE EDNFSKSLLP PSYIFPKLED VDYPDPPTNP CSFGFPVEDH AFWSWSY
Vv-XP22716  .......... ......S... .......... .......... ..Y....... .......... .......
Vv-XP22716  EPGDSSYVFE ADQSDVSQDE EDNFSKSLLP PSYIFPKLE. ..YPDPPTNP CSFGFPVEDH AFWSWSY
Vv-XP22716  EPGDSSYVFE ADQSDVSQDE EDNFSKSLLP PSYIFPKLED VDYPDPPTNP CSFGFPVEDH AFWSWSY
Cr-ABL631B  ....S..... .......... .......... .......... .......... .......... ..........
Cr-ABL631B  LSDFSQDEDD NLSISLLQPS YVFQNLK... .......... .......... .......... ..........
Cr-ABL631B  LSDFSQDEDD NLSISLLQPS YVFQNLKMNA MMNCSRIRVI WGFPLKIKLV GSGVTDDHDS LCCRISRDYF
Dc-05622.1
Dc-05622.1
Dc-05622.1
Cp-CPHB-7   S......... .........S .Y........ .......... ........
Cp-CPHB-7   .......... .......... ..DDLQPNSN CNLGFPVQNN HGTWFWQ.
Cp-CPHB-7   SLGRSLLLEA GNCLTKIEES FYDDLQPNSN CNLGFPVQNN HGTWFWQV
Cp-CPHB-6   .......... .......... .......... ...Y....Y. .......... .......... .....
Cp-CPHB-6   .......... .......... .......... .......... CLQPNSCSNL LGFPVQDHGT WFWQV
Cp-CPHB-6   DNDESLGKSS LLLQASSSCF PKIEVGDDDE SSCYEENDYY CLQPNSCSNL LGFPVQDHGT WFWQV
Gm-184278-  S.......S. S......... .......... ......S... .......... .......... .....Y....
Gm-184278-  SAKSDVLDSD SPHCTSFVE. .......... .......... .......... .......... .....YDGPP
Gm-184278-  SAKSDVLDSD SPHCTSFVEP ADSSHAFEPE DHSEDFSQDE EDNLSENLLM TFPSSCCLPK VEEHCYDGPP
Pv-HDZ2     ...S...... .........S ..S....... .......... .......Y.. .......... ..........
Pv-HDZ2     H...FVEPAD SSHAFEPDHS DFSQDEEDNL SESLLTLPCL PKVEE..YDD PPENPCNFGF HVEDQTFCFW
Pv-HDZ2     HPSSFVEPAD SSHAFEPDHS DFSQDEEDNL SESLLTLPCL PKVEEACYDD PPENPCNFGF HVEDQTFCFW
Vv-CAO4102  .......... ......S... .......... .......... SY........ .......... ......
Vv-CAO4102  EPADSSQIFE PDQSEFSQDE EDNLSKSLIP PLCFPKLED. .YPDPPANAC NLGLPAEEQP FWFWSY
Vv-CAO4102  EPADSSQIFE PDQSEFSQDE EDNLSKSLIP PLCFPKLEDC SYPDPPANAC NLGLPAEEQP FWFWSY
Rc-XP2520B  ........S. .......... .......... .......... .......... ....
Rc-XP2520B  .......... .......... .........Y HDPPANSCNF EFCVDDQPFW SWSY
Rc-XP2520B  FEPDQSDFSQ DEEDDFGRSF LPLPCFSKLY HDPPANSCNF EFCVDDQPFW SWSY
Pt_HAT5     .......S.. .......... .......... ....S..... .S........ ...
Pt_HAT5     .......... .......... ........YR EPPASSRNFE FSAEDQPFWS WIY
Pt_HAT5     ESDQPDFSQV EEDNLTRSFL PPPYFPKLYR EPPASSRNFE FSAEDQPFWS WIY
Vs-tendril  .........
Vs-tendril  .........
Vs-tendril  HWPELPYYP
At_ATHB51
At_ATHB51
At_ATHB51
At_ATHB22
At_ATHB22
At_ATHB22
Pt_93443
Pt_93443
Pt_93443
Pp_sca_65a  .......... .......... .......... .......... .......... .......
Pp_sca_65a  .......... .........T LSLQVKLEEM AGFP...... .....NQTVL PNWWDW.
Pp_sca_65a  PPESFVGPTP NPQLAGVGDT LSLQVKLEEM AGFPTDQAFL LSQLENQTVL PNWWDWA
Pp_sca_34   S......... .......... .......... .......... .......... .......... ..........
Pp_sca_34   SPRTADSSN. .......... .......... .......... ....MVSPQV KLEEMAGFP. ..........
Pp_sca_34   SPRTADSSNP STLVDASWAQ YHNPQFPPEN YVGPISNLHL ADGDMVSPQV KLEEMAGFPT DQAFLLSQLE
Pp_Pphb9    S...S....S .......... T......... .......... .......... .......... ..........
Pp_Pphb9    SPLTSHSTT. .......... .......... .......... .......... .......... ..........
Pp_Pphb9    SPLTSHSTTS WRSLYTRMAQ TDKAPWGAQH TQFPPESFAG PNFPHPCADQ SLAAAVAGAR VKIHNLGADQ
Pp_Pphb7
Pp_Pphb7
Pp_Pphb7
Pp_sca_143  ..S...S..S .........S .......S.. .......... .......... .......... ..........
Pp_sca_143  RISDCSS... .......... .......... .......... .......... .......... ..........
Pp_sca_143  RISDCSSPTS MPDQEQLIHS PEFPPPKSFG DLDHLLHELH HDVPMKLEDE SGHCLHDDQS CNYLLFHPEE
```

```
Pp_Pphb5     .......... .......... .......... .......... .......... ..........
Pp_Pphb5     .......... .......... .......... .......... ........QQ SGVLPWWDW.
Pp_Pphb5     DFPPESLMGH PQLLDTYPEE KIRLDTAVKL EDNFHEDQSC NYLLLHLDQQ SGVLPWWDWP
Pp_sca_35    .S..S..... .......... .......... .....S.... .......... .......... ..........
Pp_sca_35    SSR....... .......... .......... .......... .......... .......... ....PCGTPG
Pp_sca_35    SSRISAIQSH DFPPESFMVH PELLQIYSEE TLHPDSAVKL EDGFHEDQSC NYLLLHLDHQ SGVLPCGTPG
Pp_pphb6     .......S.. .S........ .......... .......... .......... .......... ..........
Pp_pphb6     RTTDSSS... .......... .......... .....LHDVT VKLEEGSGHG .......... .....DQAGV
Pp_pphb6     RTTDSSSLSA MSDHDQLIYS HEIPPPESFM GLSGMLHDVT VKLEEGSGHG FHDDNSSNYF LLHLEDQAGV
Pp_sca_65b   .......... .......... .......... .......... .......... .......... ........
Pp_sca_65b   .......... .......... ........QQ SFPVKLEEGC HAF....... ......VEQS VLPWWDW.
Pp_sca_65b   EKLACCSQFP PPESLVGPLG VDDDVNASQQ SFPVKLEEGC HAFQVDPNCG YFFAPAVEQS VLPWWDWP
Pp_sca_28    .........S .S........ .......... .......... .......... .......... ..........
Pp_sca_28    LLDTDSTRVS DSCY...... .......... .......... .......... .......QVN FPVKLEDASH
Pp_sca_28    LLDTDSTRVS DSCYLSRIQD EQLAGYSSNQ LPPESFVGPN LLHGDDAEPF CGRDVAQQVN FPVKLEDASH
Pp_Pphb8     .......S.. .......... .......... .......T.. .......... S......... ..........
Pp_Pphb8     SDLLDADSPR TTDSNP.... .......... .......... ......QKVY SVKLEDGCHH F.........
Pp_Pphb8     SDLLDADSPR TTDSNPREEQ LANYCGQLLP ENFVGHLTVE EAVNTSQKVY SVKLEDGCHH FQVDATIFFL
Pp_Pphb1     .......... .......S.. ...
Pp_Pphb1     VKYLTRTAFV LWIAARFSHC ...
Pp_Pphb1     VKYLTRTAFV LWIAARFSHC PIV
Pp_sca_154   .S...S.... .......... ......S... ........S. .......... ...S...... S.........
Pp_sca_154   DSPRTSDSIS .......... .......... .......... .........C HRISVKLEDG SFQD......
Pp_sca_154   DSPRTSDSIS LVPLNEFTTY PHIPTDSMVD SSIIHMSDSI CDQMLSPIDC HRISVKLEDG SFQDESCNYM
Pp_sca_77    ...S...T.. ..S....... .........S .....
Pp_sca_77    NADSPHTTSD SV........ .......... .....
Pp_sca_77    NADSPHTTSD SVSLPSLDQI TTDGLISGNS MVDSS
Pp_sca_4     .......... .......... ........Y. .......... S..S...... .......... ..........
Pp_sca_4     .......... .......... .......... ......CQRI SVKSEHDSCQ N......... ...IEEKGLP
Pp_sca_4     VPDDTNMCSL VSTELVGSSI VHMTDGLYEQ MLSPKACQRI SVKSEHDSCQ NNSCNYILAQ LDLIEEKGLP
Pp_sca_31    ...S...T.. .......... .......... .......... .....S.... ..S....... ..........
Pp_sca_31    DADSPRTTEK FN........ .......... .......... .CQRISVKLE DGSFRD.... ..........
Pp_sca_31    DADSPRTTEK FNTDLAAEVP LDSNIIHVSD HLCDHMLYPQ ACQRISVKLE DGSFRDESCN YILSQLDEER
Pp_Pphb2     ..S....... ..S....... .......... .......... .......... .......... .........S
Pp_Pphb2     SDSISSGILD ADSPLTTEMI T......... .......... .......... ........CQ QISVKLEDGS
Pp_Pphb2     SDSISSGILD ADSPLTTEMI TTTTLPHISI HVAAEMPLSS NIINMSDNLC NQMLSPHACQ QISVKLEDGS
Ps-ABK2449   .......... .......... ...S...... .......... .......... ...
Ps-ABK2449   .......... .......... .......... .......... .......... ...
Ps-ABK2449   FSNVKARDPR LECNFRPKVE ENVSQADEPC NYLFYNNLET GPLLWDYNWS SGL
Pg-ABA5414   .......... .......... ...S...... .......... .......... ...
Pg-ABA5414   .......... .......... .......... .......... .......... ...
Pg-ABA5414   FSNVKARDLR LECNFRPKVE ENVSQADEPC NYLFYNNLET GPLLWDYNWS SGL
Ps-ABK2476
Ps-ABK2476
Ps-ABK2476
Ps-ABK2462   .......... ....S..... .......... S........S .......... .......... ..........
Ps-ABK2462   IFNIDSPRTI ESPP...... .......... .......... .......... .......... ..........
Ps-ABK2462   IFNIDSPRTI ESPPSPPFPQ IIAAAAAANS SPELLVEGSS IGEQIACGLE TLPLGHESNC LPDTLPEVEA
Os_10g2650   .......... .......S.. .....T.... .......... .......... .......... ..........
Os_10g2650   .......... .......... .......... .......... .........A GSFGGLLCGA .DEQPPFWPW
Os_10g2650   GGAGGVDIDQ LLLRGGHSPS PAAVTTPPPP KMELGITGNG GGADAAAAGA GSFGGLLCGA VDEQPPFWPW
Zm_4124075   .......... .......... .......... .......... .......... .......... ..
Zm_4124075   .......... .......... .......... .GGTFGSLLC GA..DEPPPF WSWADGHHTC ..
Zm_4124075   LHSGGQPSPA AAAPPKMELA PGSPPATAGG GGGTFGSLLC GAAVDEPPPF WSWADGHHTC FQ
Sb-XP24672   .......... S......... .......... .T........ .......... .......... ..........
Sb-XP24672   .......... .......... .......... .......... ..GGTFGSLL CGA..DEQPP FWPWADGHHT
Sb-XP24672   ARADIDQLLH SGGGHPSPAP KMELGHGTAA DTPPATAAPG GGGGTFGSLL CGAAVDEQPP FWPWADGHHT
Zm_422699    .......... .......... .......... .......... .......... .......... ..
Zm_422699    .......... .......... .......... ..GGTFGSLL CGA..DEQPP FWPWADGHHA F.
Zm_422699    SYRGIPGGHP SPAPKMEPGH GAAATAGGGG GGGGTFGSLL CGAAADEQPP FWPWADGHHA FQ
Os_03g0745   ..S....... ....S..... .......... .......... .......... .......... .......
Os_03g0745   .......... .......... .......... .......... TASFGNLLCG VDEPPPFWPW PDHQHFH
Os_03g0745   PASGGGVDID QLLHSSSGGA GGPKMEHHGG GGNVQAASVD TASFGNLLCG VDEPPPFWPW PDHQHFH
Sb-XP24657   .......... ........T. .......... .......... .......... .......... ..
Sb-XP24657   .......... .......... .......... .....TASFG NLLCGVDEPP PFWPWADHQH FH
Sb-XP24657   PAAAAGVDID QLVLHTSITP KLEPLGNGAA VQAAETASFG NLLCGVDEPP PFWPWADHQH FH
Zm_4113431   .......... .......... .......... .......... .......... .....
Zm_4113431   .......... .......... ........TA SFGNLLCGVD EPPPFWPWAD HQHFH
Zm_4113431   DIDQLLHTSA PKLEQHGSGG AVVVQAAETA SFGNLLCGVD EPPPFWPWAD HQHFH
Zm_413795    .......... ...T...... .......... .......... .......... ..........
Zm_413795    .......... .......... .......... ...AGSFGNL LCGVDETPPF WPWADHHHFH
Zm_413795    AAGVGMDQLL HASTPKLEQH GDGCAVAVQA AETAGSFGNL LCGVDETPPF WPWADHHHFH
Ta-TaHZI-2   .......... .......... .......... .......... ......
Ta-TaHZI-2   .......... .........T PSFGNLLCGV DEPPPFWPWA DHQQFN
Ta-TaHZI-2   DHFLHASVPK MEHHHGGPDT PSFGNLLCGV DEPPPFWPWA DHQQFN
Rc-XP25201   .S........ .........S .......... .......... ....
Rc-XP25201   ........CQ KIDQMVKEES LSNMFCG.DD QSGFWPWLEQ QHFN
Rc-XP25201   DSHHHNIQCQ KIDQMVKEES LSNMFCGIDD QSGFWPWLEQ QHFN
Cr-ABL6311   .......... .........T .......... .......... ..........
```

```
Cr-ABL6311   .......... .......... ...ESSLCNM FCTIDDQTGF WPWLEQHHFN
Cr-ABL6311   ADHHHLIQCQ NNNNNNPTAT VKEESSLCNM FCTIDDQTGF WPWLEQHHFN
Vv-XP22768   .......... .......... .......... ......
Vv-XP22768   CQKMDQPVKE ESFCNMFCG. DDQSGFWPWL EQQHFN
Vv-XP22768   CQKMDQPVKE ESFCNMFCGM DDQSGFWPWL EQQHFN
Vv-CAO1494   .......... .......... .......... .......... .
Vv-CAO1494   .CQKIDQIVQ DESFCNIFSG .SDQSGLWAW PEQDHFH... .
Vv-CAO1494   HCQKIDQIVQ DESFCNIFSG ISDQSGLWAW PEQDHFHQNQ A
Pt_687113    ....S..... .......... ........S. .......... ......
Pt_687113    QTNPSR.... CQKIDQLVKE ENLGNMFCS. EDQSGFWPWL EQQHFN
Pt_687113    QTNPSRPDIQ CQKIDQLVKE ENLGNMFCSI EDQSGFWPWL EQQHFN
Pt_696444    .......... .........S .......... ........
Pt_696444    .YQKMDHIVK EESLTNMLCS .EDQSGFWPW LEQQQFN
Pt_696444    QYQKMDHIVK EESLTNMLCS IEDQSGFWPW LEQQQFN
Ha_HAHB1     .......... .......... .......... .......... .......... ..
Ha_HAHB1     FHNSSSR... .......... .....EECFS TMFVGMDDQS GFWPWLEQPQ FN
Ha_HAHB1     FHNSSSRPAD HQLHCHKLDQ SNAIKEECFS TMFVGMDDQS GFWPWLEQPQ FN
At_ATHB13    ........S. .......... .......... ..........
At_ATHB13    FQNSSSG... ...ENSISNM FCAMDDHSGF WPWLDQQQYN
At_ATHB13    FQNSSSGQSM VKEENSISNM FCAMDDHSGF WPWLDQQQYN
Sd-AAT4051   .......... .......... .........S ....S..... .......... .........
Sd-AAT4051   .......... .......... .......... ..EESLSNMF CGIDDQTSFW PWLEQQHFN
Sd-AAT4051   HQLFHINSSS PRQDLKLMDQ NTTTTNNNSS VKEESLSNMF CGIDDQTSFW PWLEQQHFN
At_AtHB23    .......... .....
At_AtHB23    DQSGFWPWLD QQQYN
At_AtHB23    DQSGFWPWLD QQQYN
Dc-05625.1   ..S......S .......... .......... .........
Dc-05625.1   .......... ..EEHFCNMF TGVEDQTAFW PWLEQQPFN
Dc-05625.1   RPSDHPHQQS VKEEHFCNMF TGVEDQTAFW PWLEQQPFN
Dc-05623.1   .......... .......... .......... ...
Dc-05623.1   .......... ......DDQT AFWPWLEQQQ PF.
Dc-05623.1   HHHHQPVKEE DFCNMLDDQT AFWPWLEQQQ PFN
Gm-ACU2401   .......... ...S...... .......... ........
Gm-ACU2401   ..CQKIDQMV KEESLSNMFC G.DDQSGFWP WLEQQHFN
Gm-ACU2401   ASCQKIDQMV KEESLSNMFC GMDDQSGFWP WLEQQHFN
Gm-ACU2089   .......... .S........ .......... ......
Gm-ACU2089   CQKIDQMVKE ESLSNMFCG. DDQSGFWPRL EQQHFN
Gm-ACU2089   CQKIDQMVKE ESLSNMFCGM DDQSGFWPRL EQQHFN
Mt-ACJ8462   .......... .S........
Mt-ACJ8462   CQKIDHMVKE ESLSNMFCA.
Mt-ACJ8462   CQKIDHMVKE ESLSNMFCAM
Gm-ACU1869   .......... S......... .......... .....
Gm-ACU1869   QKIDHMVKEE SLSNMFCG.D DQSGLWSWLE QQHFN
Gm-ACU1869   QKIDHMVKEE SLSNMFCGID DQSGLWSWLE QQHFN
Gm-ACU2100   .......... .S........ .......... ......
Gm-ACU2100   CQKIDHMVKE ESLSNMFCA. DDQSGLWPWL EQQHFN
Gm-ACU2100   CQKIDHMVKE ESLSNMFCAI DDQSGLWPWL EQQHFN
Rc-XP25174   .......... .......... .......... .......... .......... .....
Rc-XP25174   .......... .......EEN FCNMFNGMEE QQGFWPWPEQ HQPF...... .....
Rc-XP25174   PQCLKIDNHH HHHQLIQEEN FCNMFNGMEE QQGFWPWPEQ HQPFSLNQTH QFSNN
Pt_736296    .......... .......... .......... .......... .....
Pt_736296    .......DEN FCSMFNGIEE QQGFWPWPEQ QHAT...... .....
Pt_736296    VDQLIHQDEN FCSMFNGIEE QQGFWPWPEQ QHATFSLNQA QFSNN
Gm-ACU1987   .......... .......... ..........
Gm-ACU1987   .DESFCNMFH NIDEQQNFWP WPDHQQHQ..
Gm-ACU1987   QDESFCNMFH NIDEQQNFWP WPDHQQHQFH
Gh-ABO4774   .......... .......... ........
Gh-ABO4774   QEESFCNMFN G.DEQQAFWP WSEQQSFH
Gh-ABO4774   QEESFCNMFN GVDEQQAFWP WSEQQSFH
At_ATHB20    .......... .......... ..........
At_ATHB20    NMFNGIDETT PAGYWAW... ..........
At_ATHB20    NMFNGIDETT PAGYWAWSDP NHNHHHHQFN
At_ATHB3     .......... .......... ..........
At_ATHB3     MFNGIDETTS ASYWAW.... ..........
At_ATHB3     MFNGIDETTS ASYWAWPDQQ QQHHNHHQFN
Sm_18196
Sm_18196
Sm_18196
Sm_81609
Sm_81609
Sm_81609
Cr-CRHB6
Cr-CRHB6
Cr-CRHB6
Cr-CRHB11    .
Cr-CRHB11    .
Cr-CRHB11    I
Cr-CRHB4     .......... ...
Cr-CRHB4     PIGANDSVVA C..
```

```
Cr-CRHB4     PIGANDSVVA CAL
Sm_18217     .....S..S. .......... .......... .......... .......... .......... ..........
Sm_18217     .......... SDGYNSDVLD AFSPHPVPPA P......... .......... .......... ..........
Sm_18217     SKFKESPLSS SDGYNSDVLD AFSPHPVPPA PPPPPARAHK LSPLAPQHPA EDEEQAPPVK EEPVAFPYKL
Sm_19476     .......... S......... .......... .......... .......... .......... ..........
Sm_19476     .......... .......... .......... .......... .......... .......... ..........
Sm_19476     LVPVVQESVL SFEHTGVAVA KVLKDEPDGM VFDPHSNQLV LYKFDDGLLD YNNTSISINN NNNNNNINTN
Ps-ABK2572   .......... .......... .......... ....
Ps-ABK2572   .......... .......... .......... ....
Ps-ABK2572   YSNDFHDQLD MYRYLMAAEE QQHHGPDWLN FENL
Os_03g1021   .......... ......
Os_03g1021   .......... ......
Os_03g1021   ELAAAGYCGG VYDQFS
Zm_hox12     .......... ..........
Zm_hox12     .......... ..........
Zm_hox12     MMDLAPAYFG GVVYDYDHFN
Zm_480132    .......... ........
Zm_480132    .......... ........
Zm_480132    MMDLAPGYFG GVVYDHFN
Zm_433132    ....
Zm_433132    ....
Zm_433132    YGLM
Zm_433210
Zm_433210
Zm_433210
Os_07g3932   Y...S..... ......
Os_07g3932   .......... ......
Os_07g3932   YADNSVVEWF SLYGLI
Pt_655260
Pt_655260
Pt_655260
Pt_703426
Pt_703426
Pt_703426
At_ATHB53
At_ATHB53
At_ATHB53
At_ATHB40
At_ATHB40
At_ATHB40
At_ATHB21
At_ATHB21
At_ATHB21
Gm-ACU198B   .
Gm-ACU198B   .
Gm-ACU198B   G
Pt_98386
Pt_98386
Pt_98386
Pt_568845
Pt_568845
Pt_568845
At_ATHB52
At_ATHB52
At_ATHB52
Pt_594622
Pt_594622
Pt_594622
Pt_unknon
Pt_unknon
Pt_unknon
```

```
           141
Zm_479999  .......... .......... .......... .......... .......... ..T....... ..........
Zm_479999  .......... .......... .......... .......... .......... .......... ..........
Zm_479999  FRLLVWKDAG DAIVVSAIHS PRCCCAGRKG GGGKGRCREF GTFWTDGWRK RLTGRESDAQ PNHKYAKCSH
Os_02g4333
Os_02g4333
Os_02g4333
Sb-XP24528
Sb-XP24528
Sb-XP24528
Zm_470295
Zm_470295
Zm_470295
Sb-XP24468
Sb-XP24468
Sb-XP24468
Zm_433493
Zm_433493
Zm_433493
Os_04g4581
Os_04g4581
Os_04g4581
Zm_4492
Zm_4492
Zm_4492
Sb-XP24627
Sb-XP24627
Sb-XP24627
Os_09g3591
Os_09g3591
Os_09g3591
Zm_4496
Zm_4496
Zm_4496
Vv-XP22629
Vv-XP22629
Vv-XP22629
Vv-CAN7896
Vv-CAN7896
Vv-CAN7896
Rc-XP25299 .......
Rc-XP25299 .......
Rc-XP25299 AEFGDFY
Mt_MTHB1
Mt_MTHB1
Mt_MTHB1
Gm-ACU2443
Gm-ACU2443
Gm-ACU2443
Pt_HB7
Pt_HB7
Pt_HB7
Pt_731421
Pt_731421
Pt_731421
At_ATHB12
At_ATHB12
At_ATHB12
At_ATHB7
At_ATHB7
At_ATHB7
Zm_hox6
Zm_hox6
Zm_hox6
Vv-XP22800
Vv-XP22800
Vv-XP22800
Pt_548258
Pt_548258
Pt_548258
Pt_343725
Pt_343725
Pt_343725
Ha_HAHB4
Ha_HAHB4
Ha_HAHB4
Vv-XP22715
Vv-XP22715
Vv-XP22715
```

```
Ha_HAHB11
Ha_HAHB11
Ha_HAHB11
Pt_HBLZ
Pt_HBLZ
Pt_HBLZ
Sb-XP24459
Sb-XP24459
Sb-XP24459
Os_08g3758
Os_08g3758
Os_08g3758
Sb-XP24603
Sb-XP24603
Sb-XP24603
Zm_4112646
Zm_4112646
Zm_4112646
Os_09g2946
Os_09g2946
Os_09g2946
Zm_469357
Zm_469357
Zm_469357
Zm_469358
Zm_469358
Zm_469358
Ta-TaHZI-1
Ta-TaHZI-1
Ta-TaHZI-1
Zm_483405
Zm_483405
Zm_483405
Os_10g2309   .
Os_10g2309   .
Os_10g2309   W
Zm_4102187   .......... .......
Zm_4102187   ASSLNFNWCP .......
Zm_4102187   ASSLNFNWCP SGIQGWE
Zm_4118271   .......... .......
Zm_4118271   ASSLNFNWCP .......
Zm_4118271   ASSLNFNWCP SGIQGWE
Os_03g0896
Os_03g0896
Os_03g0896
Cp-CPHB-4
Cp-CPHB-4
Cp-CPHB-4
At_ATHB5     .
At_ATHB5     .
At_ATHB5     S
Sl-SlHDL1    .......... ..........
Sl-SlHDL1    FSDEAAPTLH WYTPD.....
Sl-SlHDL1    FSDEAAPTLH WYTPDQWTDV
Ze-18171.1
Ze-18171.1
Ze-18171.1
Nt-Hfi22     .......... .......... .......... .......... ..........
Nt-Hfi22     ......QPQQ YVKMEEHNFF N..ESCSTLF TDEQAPTLQW YCPE......
Nt-Hfi22     GNNNYYQPQQ YVKMEEHNFF NGEESCSTLF TDEQAPTLQW YCPEDWNWKE
Brs-hb-6     .........
Brs-hb-6     WYSTV....
Brs-hb-6     WYSTVDQWN
Bn-AAR0493   ..........
Bn-AAR0493   HWYSTV....
Bn-AAR0493   HWYSTVDQWN
At_ATHB6     ..........
At_ATHB6     HWYSTV....
At_ATHB6     HWYSTVDHWN
At_ATHB16
At_ATHB16
At_ATHB16
Pt_HD56      ...S...... .......... .........
Pt_HD56      NFFS.EEACN FFSDEQPPSL PWYSSD...
Pt_HD56      NFFSSEEACN FFSDEQPPSL PWYSSDQWN
Pt_70493     ....S..... .......... ..........
Pt_70493     HNFLS.EEAC NFFSDEQPPT LHWYCSD...
Pt_70493     HNFLSSEEAC NFFSDEQPPT LHWYCSDQWN
Vv-XP22660   .......... .......... .......... .........
```

```
Vv-XP22660 QPQFVKMEEH NFFG.DESCN FFSDDQPPTL PWYCPD...
Vv-XP22660 QPQFVKMEEH NFFGADESCN FFSDDQPPTL PWYCPDQWT
Mt-ACJ8504 ...
Mt-ACJ8504 ...
Mt-ACJ8504 QWS
Pv-HDZ1    .......
Pv-HDZ1    YCSE...
Pv-HDZ1    YCSEEWS
Gm-184277- .......... ......
Gm-184277- DEQAPTLQWY CSE...
Gm-184277- DEQAPTLQWY CSEEWS
Sl-CAB6711
Sl-CAB6711
Sl-CAB6711
Vv-CAO6167
Vv-CAO6167
Vv-CAO6167
Vv-XP22857
Vv-XP22857
Vv-XP22857
Vv-CAN8361
Vv-CAN8361
Vv-CAN8361
Pt_unknown
Pt_unknown
Pt_unknown
Pt_HB
Pt_HB
Pt_HB
Rc-XP25139
Rc-XP25139
Rc-XP25139
Os_02g4970 .......... .......... .......
Os_02g4970 .......... .....DGQQI SCWWMW.
Os_02g4970 HGHHHHHQHA DDDEEDGQQI SCWWMWN
Zm_4134425 .......... .......
Zm_4134425 .....EDAGQ ISWWMW.
Zm_4134425 HADEDEDAGQ ISWWMWN
Zm_459693
Zm_459693
Zm_459693
Zm_459694
Zm_459694
Zm_459694
Os_08g3208 .......... .......... ....
Os_08g3208 .......... EEAQLGSWTA W...
Os_08g3208 GVVHHDAADD EEAQLGSWTA WFWS
Zm_4124691
Zm_4124691
Zm_4124691
Os_09g2118
Os_09g2118
Os_09g2118
Lj-BAG5005
Lj-BAG5005
Lj-BAG5005
Pv-HDZ3
Pv-HDZ3
Pv-HDZ3
Gm-ACU1808
Gm-ACU1808
Gm-ACU1808
Rc-XP25173
Rc-XP25173
Rc-XP25173
Vv-XP22788
Vv-XP22788
Vv-XP22788
At_ATHB1
At_ATHB1
At_ATHB1
At_ATHB54  .......SSS .......... .......... .......... .......... .......... ......S...
At_ATHB54  .......... .......... .......... .......... .......... .......... ..........
At_ATHB54  RRRPWAPSSS EHDEIIDKPI TKPPPPPALV VMGLPANCSV LELKSRFEIY GSISRIRIHK DGIGSVSYRT
Ps-ABR1622 .......... .S.....
Ps-ABR1622 .....DDQEN LSWWDW.
Ps-ABR1622 YVFAMDDQEN LSWWDWP
Sl-CAA6441
Sl-CAA6441
```

```
Sl-CAA6441
Mt-ACJ8510   ...
Mt-ACJ8510   W..
Mt-ACJ8510   WSY
Gm-AAX9867
Gm-AAX9867
Gm-AAX9867
Dc-05624.1
Dc-05624.1
Dc-05624.1
Rc-XP25115
Rc-XP25115
Rc-XP25115
Pt_88244
Pt_88244
Pt_88244
Vv-CAO6250
Vv-CAO6250
Vv-CAO6250
Vv-CAN8396
Vv-CAN8396
Vv-CAN8396
Vv-XP22716
Vv-XP22716
Vv-XP22716
Cr-ABL631B   ..
Cr-ABL631B   ..
Cr-ABL631B   LM
Dc-05622.1
Dc-05622.1
Dc-05622.1
Cp-CPHB-7
Cp-CPHB-7
Cp-CPHB-7
Cp-CPHB-6
Cp-CPHB-6
Cp-CPHB-6
Gm-184278-   .......... ..........
Gm-184278-   ENSCNFGFQV EDQTFCFWPY
Gm-184278-   ENSCNFGFQV EDQTFCFWPY
Pv-HDZ2      ..
Pv-HDZ2      PY
Pv-HDZ2      PY
Vv-CAO4102
Vv-CAO4102
Vv-CAO4102
Rc-XP2520B
Rc-XP2520B
Rc-XP2520B
Pt_HAT5
Pt_HAT5
Pt_HAT5
Vs-tendril
Vs-tendril
Vs-tendril
At_ATHB51
At_ATHB51
At_ATHB51
At_ATHB22
At_ATHB22
At_ATHB22
Pt_93443
Pt_93443
Pt_93443
Pp_sca_65a
Pp_sca_65a
Pp_sca_65a
Pp_sca_34    .......... ..
Pp_sca_34    NQTILPNWWD W.
Pp_sca_34    NQTILPNWWD WA
Pp_Pphb9     .......... ..........
Pp_Pphb9     ........EQ TILTNWWDW.
Pp_Pphb9     AFVASRMEEQ TILTNWWDWD
Pp_Pphb7
Pp_Pphb7
Pp_Pphb7
Pp_sca_143   .......... .......... ...S.S.... .......... .......S.. ...S...... .S.....S..
Pp_sca_143   .......... .......... .......... .......... .......... .......... ..........
Pp_sca_143   QTCVLPCPFV RPPLSRNPNY RSRSWSMDEQ RMRLRRRERM RVGPKKYSTE EQDSRFGLLR KSLQEENSWI
```

```
Pp_Pphb5
Pp_Pphb5
Pp_Pphb5
Pp_sca_35     .......... .......... .......... ...
Pp_sca_35     LVVGLAMVGL SSGA...... .......... ...
Pp_sca_35     LVVGLAMVGL SSGAELQSDE RIGKQNKTKP KPK
Pp_pphb6      .......
Pp_pphb6      LPWWDW.
Pp_pphb6      LPWWDWP
Pp_sca_65b
Pp_sca_65b
Pp_sca_65b
Pp_sca_28     .......... .......... .......... ...
Pp_sca_28     FQ........ .......... .......... ...
Pp_sca_28     FQMPDIPNCI YFLPQIVSEQ SVLPCHIVVE LAL
Pp_Pphb8      .......... .......... ...
Pp_Pphb8      .......... .......... ...
Pp_Pphb8      GGIGRNRGIQ IVPLRHFFPL DLG
Pp_Pphb1
Pp_Pphb1
Pp_Pphb1
Pp_sca_154    .......... ......
Pp_sca_154    ....DEERGL PWWDW.
Pp_sca_154    LSQLDEERGL PWWDWP
Pp_sca_77
Pp_sca_77
Pp_sca_77
Pp_sca_4      .....
Pp_sca_4      WWDW.
Pp_sca_4      WWDWP
Pp_sca_31     .......... .T........ .......... .......... .......... .......... ..........
Pp_sca_31     .......... .......... .......... .......... .......... .......... ..........
Pp_sca_31     ELYCKWSDTM ATPCTAFGWR CHQSGALPLN RIRQPGADTI SSRAPTKGLG WKSVISVQQM KTWTLVAGLG
Pp_Pphb2      S...S..... .......... .......... .......... .......... .........
Pp_Pphb2      SQD....... ....DEERGL PWHYH..... .......... .......... .........
Pp_Pphb2      SQDESSSNYI LTQLDEERGL PWHYHRQHNR FVRSGAWWSR CSAESLDLLS LPDYSAFVL
Ps-ABK2449
Ps-ABK2449
Ps-ABK2449
Pg-ABA5414
Pg-ABA5414
Pg-ABA5414
Ps-ABK2476
Ps-ABK2476
Ps-ABK2476
Ps-ABK2462    .......... .......... .......... ....
Ps-ABK2462    .......... .......... DEQGPLMLWD Y...
Ps-ABK2462    DQIASLNQAE ETCRNLFYSL DEQGPLMLWD YWPQ
Os_10g2650    ........
Os_10g2650    ADGHHHF.
Os_10g2650    ADGHHHFH
Zm_4124075
Zm_4124075
Zm_4124075
Sb-XP24672    ..
Sb-XP24672    F.
Sb-XP24672    FQ
Zm_422699
Zm_422699
Zm_422699
Os_03g0745
Os_03g0745
Os_03g0745
Sb-XP24657
Sb-XP24657
Sb-XP24657
Zm_4113431
Zm_4113431
Zm_4113431
Zm_413795
Zm_413795
Zm_413795
Ta-TaHZI-2
Ta-TaHZI-2
Ta-TaHZI-2
Rc-XP25201
Rc-XP25201
Rc-XP25201
Cr-ABL6311
```

Cr-ABL6311
Cr-ABL6311
Vv-XP22768
Vv-XP22768
Vv-XP22768
Vv-CAO1494
Vv-CAO1494
Vv-CAO1494
Pt_687113
Pt_687113
Pt_687113
Pt_696444
Pt_696444
Pt_696444
Ha_HAHB1
Ha_HAHB1
Ha_HAHB1
At_ATHB13
At_ATHB13
At_ATHB13
Sd-AAT4051
Sd-AAT4051
Sd-AAT4051
At_AtHB23
At_AtHB23
At_AtHB23
Dc-05625.1
Dc-05625.1
Dc-05625.1
Dc-05623.1
Dc-05623.1
Dc-05623.1
Gm-ACU2401
Gm-ACU2401
Gm-ACU2401
Gm-ACU2089
Gm-ACU2089
Gm-ACU2089
Mt-ACJ8462
Mt-ACJ8462
Mt-ACJ8462
Gm-ACU1869
Gm-ACU1869
Gm-ACU1869
Gm-ACU2100
Gm-ACU2100
Gm-ACU2100
Rc-XP25174
Rc-XP25174
Rc-XP25174
Pt_736296
Pt_736296
Pt_736296
Gm-ACU1987
Gm-ACU1987
Gm-ACU1987
Gh-ABO4774
Gh-ABO4774
Gh-ABO4774
At_ATHB20
At_ATHB20
At_ATHB20
At_ATHB3
At_ATHB3
At_ATHB3
Sm_18196
Sm_18196
Sm_18196
Sm_81609
Sm_81609
Sm_81609
Cr-CRHB6
Cr-CRHB6
Cr-CRHB6
Cr-CRHB11
Cr-CRHB11
Cr-CRHB11
Cr-CRHB4
Cr-CRHB4

```
Cr-CRHB4
Sm_18217      ..........  ..........  ..........  .......
Sm_18217      ..........  ..........  .....EQGNW  ASWWDW.
Sm_18217      DDDDALRGLE  DDASYNCLFG  TAAEEEQGNW  ASWWDWA
Sm_19476      ..........  ..........  ..........  .
Sm_19476      ..........  ..........  ..........  .
Sm_19476      NSNSINANVN  EYDMATSSYY  YIPDGIGPWW  T
Ps-ABK2572
Ps-ABK2572
Ps-ABK2572
Os_03g1021
Os_03g1021
Os_03g1021
Zm_hox12
Zm_hox12
Zm_hox12
Zm_480132
Zm_480132
Zm_480132
Zm_433132
Zm_433132
Zm_433132
Zm_433210
Zm_433210
Zm_433210
Os_07g3932
Os_07g3932
Os_07g3932
Pt_655260
Pt_655260
Pt_655260
Pt_703426
Pt_703426
Pt_703426
At_ATHB53
At_ATHB53
At_ATHB53
At_ATHB40
At_ATHB40
At_ATHB40
At_ATHB21
At_ATHB21
At_ATHB21
Gm-ACU198B
Gm-ACU198B
Gm-ACU198B
Pt_98386
Pt_98386
Pt_98386
Pt_568845
Pt_568845
Pt_568845
At_ATHB52
At_ATHB52
At_ATHB52
Pt_594622
Pt_594622
Pt_594622
Pt_unknon
Pt_unknon
Pt_unknon
```

```
             211
Zm_479999   .......... .......... .......... .......... .S........ .......... ..........
Zm_479999   .......... .......... .......... .......... .......... .......... ..........
Zm_479999   ELPESLTLFG RISGLVAATS QSMVGRAPKL TLYLGRNLIK KSSERLPSVP LLSSYCRSFA AAMAPALRTG
Os_02g4333
Os_02g4333
Os_02g4333
Sb-XP24528
Sb-XP24528
Sb-XP24528
Zm_470295
Zm_470295
Zm_470295
Sb-XP24468
Sb-XP24468
Sb-XP24468
Zm_433493
Zm_433493
Zm_433493
Os_04g4581
Os_04g4581
Os_04g4581
Zm_4492
Zm_4492
Zm_4492
Sb-XP24627
Sb-XP24627
Sb-XP24627
Os_09g3591
Os_09g3591
Os_09g3591
Zm_4496
Zm_4496
Zm_4496
Vv-XP22629
Vv-XP22629
Vv-XP22629
Vv-CAN7896
Vv-CAN7896
Vv-CAN7896
Rc-XP25299
Rc-XP25299
Rc-XP25299
Mt_MTHB1
Mt_MTHB1
Mt_MTHB1
Gm-ACU2443
Gm-ACU2443
Gm-ACU2443
Pt_HB7
Pt_HB7
Pt_HB7
Pt_731421
Pt_731421
Pt_731421
At_ATHB12
At_ATHB12
At_ATHB12
At_ATHB7
At_ATHB7
At_ATHB7
Zm_hox6
Zm_hox6
Zm_hox6
Vv-XP22800
Vv-XP22800
Vv-XP22800
Pt_548258
Pt_548258
Pt_548258
Pt_343725
Pt_343725
Pt_343725
Ha_HAHB4
Ha_HAHB4
Ha_HAHB4
Vv-XP22715
Vv-XP22715
Vv-XP22715
```

Ha_HAHB11
Ha_HAHB11
Ha_HAHB11
Pt_HBLZ
Pt_HBLZ
Pt_HBLZ
Sb-XP24459
Sb-XP24459
Sb-XP24459
Os_08g3758
Os_08g3758
Os_08g3758
Sb-XP24603
Sb-XP24603
Sb-XP24603
Zm_4112646
Zm_4112646
Zm_4112646
Os_09g2946
Os_09g2946
Os_09g2946
Zm_469357
Zm_469357
Zm_469357
Zm_469358
Zm_469358
Zm_469358
Ta-TaHZI-1
Ta-TaHZI-1
Ta-TaHZI-1
Zm_483405
Zm_483405
Zm_483405
Os_10g2309
Os_10g2309
Os_10g2309
Zm_4102187
Zm_4102187
Zm_4102187
Zm_4118271
Zm_4118271
Zm_4118271
Os_03g0896
Os_03g0896
Os_03g0896
Cp-CPHB-4
Cp-CPHB-4
Cp-CPHB-4
At_ATHB5
At_ATHB5
At_ATHB5
Sl-SlHDL1
Sl-SlHDL1
Sl-SlHDL1
Ze-18171.1
Ze-18171.1
Ze-18171.1
Nt-Hfi22
Nt-Hfi22
Nt-Hfi22
Brs-hb-6
Brs-hb-6
Brs-hb-6
Bn-AAR0493
Bn-AAR0493
Bn-AAR0493
At_ATHB6
At_ATHB6
At_ATHB6
At_ATHB16
At_ATHB16
At_ATHB16
Pt_HD56
Pt_HD56
Pt_HD56
Pt_70493
Pt_70493
Pt_70493
Vv-XP22660

```
Vv-XP22660
Vv-XP22660
Mt-ACJ8504
Mt-ACJ8504
Mt-ACJ8504
Pv-HDZ1
Pv-HDZ1
Pv-HDZ1
Gm-184277-
Gm-184277-
Gm-184277-
Sl-CAB6711
Sl-CAB6711
Sl-CAB6711
Vv-CAO6167
Vv-CAO6167
Vv-CAO6167
Vv-XP22857
Vv-XP22857
Vv-XP22857
Vv-CAN8361
Vv-CAN8361
Vv-CAN8361
Pt_unknown
Pt_unknown
Pt_unknown
Pt_HB
Pt_HB
Pt_HB
Rc-XP25139
Rc-XP25139
Rc-XP25139
Os_02g4970
Os_02g4970
Os_02g4970
Zm_4134425
Zm_4134425
Zm_4134425
Zm_459693
Zm_459693
Zm_459693
Zm_459694
Zm_459694
Zm_459694
Os_08g3208
Os_08g3208
Os_08g3208
Zm_4124691
Zm_4124691
Zm_4124691
Os_09g2118
Os_09g2118
Os_09g2118
Lj-BAG5005
Lj-BAG5005
Lj-BAG5005
Pv-HDZ3
Pv-HDZ3
Pv-HDZ3
Gm-ACU1808
Gm-ACU1808
Gm-ACU1808
Rc-XP25173
Rc-XP25173
Rc-XP25173
Vv-XP22788
Vv-XP22788
Vv-XP22788
At_ATHB1
At_ATHB1
At_ATHB1
At_ATHB54   ..S....... S......... .......... .......... .......... SS.S...... .........S
At_ATHB54   .......... .......... .......... .......... .......... .......... ..........
At_ATHB54   AESAEAAIAG SHEPSFGISI DSKKLEVVWA TDPLVKWKEG VTAGEGKERT SSFSSKLLRP VMPLRKHGRS
Ps-ABR1622
Ps-ABR1622
Ps-ABR1622
Sl-CAA6441
Sl-CAA6441
```

```
Sl-CAA6441
Mt-ACJ8510
Mt-ACJ8510
Mt-ACJ8510
Gm-AAX9867
Gm-AAX9867
Gm-AAX9867
Dc-05624.1
Dc-05624.1
Dc-05624.1
Rc-XP25115
Rc-XP25115
Rc-XP25115
Pt_88244
Pt_88244
Pt_88244
Vv-CAO6250
Vv-CAO6250
Vv-CAO6250
Vv-CAN8396
Vv-CAN8396
Vv-CAN8396
Vv-XP22716
Vv-XP22716
Vv-XP22716
Cr-ABL631B
Cr-ABL631B
Cr-ABL631B
Dc-05622.1
Dc-05622.1
Dc-05622.1
Cp-CPHB-7
Cp-CPHB-7
Cp-CPHB-7
Cp-CPHB-6
Cp-CPHB-6
Cp-CPHB-6
Gm-184278-
Gm-184278-
Gm-184278-
Pv-HDZ2
Pv-HDZ2
Pv-HDZ2
Vv-CAO4102
Vv-CAO4102
Vv-CAO4102
Rc-XP2520B
Rc-XP2520B
Rc-XP2520B
Pt_HAT5
Pt_HAT5
Pt_HAT5
Vs-tendril
Vs-tendril
Vs-tendril
At_ATHB51
At_ATHB51
At_ATHB51
At_ATHB22
At_ATHB22
At_ATHB22
Pt_93443
Pt_93443
Pt_93443
Pp_sca_65a
Pp_sca_65a
Pp_sca_65a
Pp_sca_34
Pp_sca_34
Pp_sca_34
Pp_Pphb9
Pp_Pphb9
Pp_Pphb9
Pp_Pphb7
Pp_Pphb7
Pp_Pphb7
Pp_sca_143   ....S..... .......... .......... .......... .......... .......... ........Y.
Pp_sca_143   .......... .......... .......... .......... .......... .......... ..........
Pp_sca_143   DDGTSPIANR SSRNICLDME HHHPIFRSLD FHSLHMGFLF LCLDQRPLRH CKAQREVASK ENCADCGEYA
```

```
Pp_Pphb5
Pp_Pphb5
Pp_Pphb5
Pp_sca_35
Pp_sca_35
Pp_sca_35
Pp_pphb6
Pp_pphb6
Pp_pphb6
Pp_sca_65b
Pp_sca_65b
Pp_sca_65b
Pp_sca_28
Pp_sca_28
Pp_sca_28
Pp_Pphb8
Pp_Pphb8
Pp_Pphb8
Pp_Pphb1
Pp_Pphb1
Pp_Pphb1
Pp_sca_154
Pp_sca_154
Pp_sca_154
Pp_sca_77
Pp_sca_77
Pp_sca_77
Pp_sca_4
Pp_sca_4
Pp_sca_4
Pp_sca_31    .......... SS........ .......... .......... .......... .........S ..........
Pp_sca_31    .......... .......... .......... .......... .......... .......... ..........
Pp_sca_31    TSTPYTGHRT SSEGSFHNWG LVSDLFITVT IFLIISQLGG RCVGLQRNNA VRPFLTRPHS NIMAHDSEAL
Pp_Pphb2
Pp_Pphb2
Pp_Pphb2
Ps-ABK2449
Ps-ABK2449
Ps-ABK2449
Pg-ABA5414
Pg-ABA5414
Pg-ABA5414
Ps-ABK2476
Ps-ABK2476
Ps-ABK2476
Ps-ABK2462
Ps-ABK2462
Ps-ABK2462
Os_10g2650
Os_10g2650
Os_10g2650
Zm_4124075
Zm_4124075
Zm_4124075
Sb-XP24672
Sb-XP24672
Sb-XP24672
Zm_422699
Zm_422699
Zm_422699
Os_03g0745
Os_03g0745
Os_03g0745
Sb-XP24657
Sb-XP24657
Sb-XP24657
Zm_4113431
Zm_4113431
Zm_4113431
Zm_413795
Zm_413795
Zm_413795
Ta-TaHZI-2
Ta-TaHZI-2
Ta-TaHZI-2
Rc-XP25201
Rc-XP25201
Rc-XP25201
Cr-ABL6311
```

Cr-ABL6311
Cr-ABL6311
Vv-XP22768
Vv-XP22768
Vv-XP22768
Vv-CAO1494
Vv-CAO1494
Vv-CAO1494
Pt_687113
Pt_687113
Pt_687113
Pt_696444
Pt_696444
Pt_696444
Ha_HAHB1
Ha_HAHB1
Ha_HAHB1
At_ATHB13
At_ATHB13
At_ATHB13
Sd-AAT4051
Sd-AAT4051
Sd-AAT4051
At_AtHB23
At_AtHB23
At_AtHB23
Dc-05625.1
Dc-05625.1
Dc-05625.1
Dc-05623.1
Dc-05623.1
Dc-05623.1
Gm-ACU2401
Gm-ACU2401
Gm-ACU2401
Gm-ACU2089
Gm-ACU2089
Gm-ACU2089
Mt-ACJ8462
Mt-ACJ8462
Mt-ACJ8462
Gm-ACU1869
Gm-ACU1869
Gm-ACU1869
Gm-ACU2100
Gm-ACU2100
Gm-ACU2100
Rc-XP25174
Rc-XP25174
Rc-XP25174
Pt_736296
Pt_736296
Pt_736296
Gm-ACU1987
Gm-ACU1987
Gm-ACU1987
Gh-ABO4774
Gh-ABO4774
Gh-ABO4774
At_ATHB20
At_ATHB20
At_ATHB20
At_ATHB3
At_ATHB3
At_ATHB3
Sm_18196
Sm_18196
Sm_18196
Sm_81609
Sm_81609
Sm_81609
Cr-CRHB6
Cr-CRHB6
Cr-CRHB6
Cr-CRHB11
Cr-CRHB11
Cr-CRHB11
Cr-CRHB4
Cr-CRHB4

Cr-CRHB4
Sm_18217
Sm_18217
Sm_18217
Sm_19476
Sm_19476
Sm_19476
Ps-ABK2572
Ps-ABK2572
Ps-ABK2572
Os_03g1021
Os_03g1021
Os_03g1021
Zm_hox12
Zm_hox12
Zm_hox12
Zm_480132
Zm_480132
Zm_480132
Zm_433132
Zm_433132
Zm_433132
Zm_433210
Zm_433210
Zm_433210
Os_07g3932
Os_07g3932
Os_07g3932
Pt_655260
Pt_655260
Pt_655260
Pt_703426
Pt_703426
Pt_703426
At_ATHB53
At_ATHB53
At_ATHB53
At_ATHB40
At_ATHB40
At_ATHB40
At_ATHB21
At_ATHB21
At_ATHB21
Gm-ACU198B
Gm-ACU198B
Gm-ACU198B
Pt_98386
Pt_98386
Pt_98386
Pt_568845
Pt_568845
Pt_568845
At_ATHB52
At_ATHB52
At_ATHB52
Pt_594622
Pt_594622
Pt_594622
Pt_unknon
Pt_unknon
Pt_unknon

```
             281
Zm_479999    ....T..... .......... .......... .......... .......... ...
Zm_479999    .......... .......... ........YT LNKK...... .......... ...
Zm_479999    HRCSTPVVAS FAMSYVQIAT QGHYEHLPYT LNKKRKGPFA KQFAKKARTS NLI
Os_02g4333
Os_02g4333
Os_02g4333
Sb-XP24528
Sb-XP24528
Sb-XP24528
Zm_470295
Zm_470295
Zm_470295
Sb-XP24468
Sb-XP24468
Sb-XP24468
Zm_433493
Zm_433493
Zm_433493
Os_04g4581
Os_04g4581
Os_04g4581
Zm_4492
Zm_4492
Zm_4492
Sb-XP24627
Sb-XP24627
Sb-XP24627
Os_09g3591
Os_09g3591
Os_09g3591
Zm_4496
Zm_4496
Zm_4496
Vv-XP22629
Vv-XP22629
Vv-XP22629
Vv-CAN7896
Vv-CAN7896
Vv-CAN7896
Rc-XP25299
Rc-XP25299
Rc-XP25299
Mt_MTHB1
Mt_MTHB1
Mt_MTHB1
Gm-ACU2443
Gm-ACU2443
Gm-ACU2443
Pt_HB7
Pt_HB7
Pt_HB7
Pt_731421
Pt_731421
Pt_731421
At_ATHB12
At_ATHB12
At_ATHB12
At_ATHB7
At_ATHB7
At_ATHB7
Zm_hox6
Zm_hox6
Zm_hox6
Vv-XP22800
Vv-XP22800
Vv-XP22800
Pt_548258
Pt_548258
Pt_548258
Pt_343725
Pt_343725
Pt_343725
Ha_HAHB4
Ha_HAHB4
Ha_HAHB4
Vv-XP22715
Vv-XP22715
Vv-XP22715
```

Ha_HAHB11
Ha_HAHB11
Ha_HAHB11
Pt_HBLZ
Pt_HBLZ
Pt_HBLZ
Sb-XP24459
Sb-XP24459
Sb-XP24459
Os_08g3758
Os_08g3758
Os_08g3758
Sb-XP24603
Sb-XP24603
Sb-XP24603
Zm_4112646
Zm_4112646
Zm_4112646
Os_09g2946
Os_09g2946
Os_09g2946
Zm_469357
Zm_469357
Zm_469357
Zm_469358
Zm_469358
Zm_469358
Ta-TaHZI-1
Ta-TaHZI-1
Ta-TaHZI-1
Zm_483405
Zm_483405
Zm_483405
Os_10g2309
Os_10g2309
Os_10g2309
Zm_4102187
Zm_4102187
Zm_4102187
Zm_4118271
Zm_4118271
Zm_4118271
Os_03g0896
Os_03g0896
Os_03g0896
Cp-CPHB-4
Cp-CPHB-4
Cp-CPHB-4
At_ATHB5
At_ATHB5
At_ATHB5
Sl-SlHDL1
Sl-SlHDL1
Sl-SlHDL1
Ze-18171.1
Ze-18171.1
Ze-18171.1
Nt-Hfi22
Nt-Hfi22
Nt-Hfi22
Brs-hb-6
Brs-hb-6
Brs-hb-6
Bn-AAR0493
Bn-AAR0493
Bn-AAR0493
At_ATHB6
At_ATHB6
At_ATHB6
At_ATHB16
At_ATHB16
At_ATHB16
Pt_HD56
Pt_HD56
Pt_HD56
Pt_70493
Pt_70493
Pt_70493
Vv-XP22660

```
Vv-XP22660
Vv-XP22660
Mt-ACJ8504
Mt-ACJ8504
Mt-ACJ8504
Pv-HDZ1
Pv-HDZ1
Pv-HDZ1
Gm-184277-
Gm-184277-
Gm-184277-
Sl-CAB6711
Sl-CAB6711
Sl-CAB6711
Vv-CAO6167
Vv-CAO6167
Vv-CAO6167
Vv-XP22857
Vv-XP22857
Vv-XP22857
Vv-CAN8361
Vv-CAN8361
Vv-CAN8361
Pt_unknown
Pt_unknown
Pt_unknown
Pt_HB
Pt_HB
Pt_HB
Rc-XP25139
Rc-XP25139
Rc-XP25139
Os_02g4970
Os_02g4970
Os_02g4970
Zm_4134425
Zm_4134425
Zm_4134425
Zm_459693
Zm_459693
Zm_459693
Zm_459694
Zm_459694
Zm_459694
Os_08g3208
Os_08g3208
Os_08g3208
Zm_4124691
Zm_4124691
Zm_4124691
Os_09g2118
Os_09g2118
Os_09g2118
Lj-BAG5005
Lj-BAG5005
Lj-BAG5005
Pv-HDZ3
Pv-HDZ3
Pv-HDZ3
Gm-ACU1808
Gm-ACU1808
Gm-ACU1808
Rc-XP25173
Rc-XP25173
Rc-XP25173
Vv-XP22788
Vv-XP22788
Vv-XP22788
At_ATHB1
At_ATHB1
At_ATHB1
At_ATHB54    S...S..... .......... .........T .......... .....
At_ATHB54    .......... .......... .......... .......... .....
At_ATHB54    SRLASAIVNP RSDNTKGISG DGGISSPATT SEVKQRNIVT YDDIV
Ps-ABR1622
Ps-ABR1622
Ps-ABR1622
Sl-CAA6441
Sl-CAA6441
```

```
Sl-CAA6441
Mt-ACJ8510
Mt-ACJ8510
Mt-ACJ8510
Gm-AAX9867
Gm-AAX9867
Gm-AAX9867
Dc-05624.1
Dc-05624.1
Dc-05624.1
Rc-XP25115
Rc-XP25115
Rc-XP25115
Pt_88244
Pt_88244
Pt_88244
Vv-CAO6250
Vv-CAO6250
Vv-CAO6250
Vv-CAN8396
Vv-CAN8396
Vv-CAN8396
Vv-XP22716
Vv-XP22716
Vv-XP22716
Cr-ABL631B
Cr-ABL631B
Cr-ABL631B
Dc-05622.1
Dc-05622.1
Dc-05622.1
Cp-CPHB-7
Cp-CPHB-7
Cp-CPHB-7
Cp-CPHB-6
Cp-CPHB-6
Cp-CPHB-6
Gm-184278-
Gm-184278-
Gm-184278-
Pv-HDZ2
Pv-HDZ2
Pv-HDZ2
Vv-CAO4102
Vv-CAO4102
Vv-CAO4102
Rc-XP2520B
Rc-XP2520B
Rc-XP2520B
Pt_HAT5
Pt_HAT5
Pt_HAT5
Vs-tendril
Vs-tendril
Vs-tendril
At_ATHB51
At_ATHB51
At_ATHB51
At_ATHB22
At_ATHB22
At_ATHB22
Pt_93443
Pt_93443
Pt_93443
Pp_sca_65a
Pp_sca_65a
Pp_sca_65a
Pp_sca_34
Pp_sca_34
Pp_sca_34
Pp_Pphb9
Pp_Pphb9
Pp_Pphb9
Pp_Pphb7
Pp_Pphb7
Pp_Pphb7
Pp_sca_143  .......... .......... .......... .......... .......... .......... ..........
Pp_sca_143  .......... ..LNLNCK.. .......... .......... .......... .......... ..........
Pp_sca_143  QPSQGAIAEN LSLNLNCKPQ TPAPFTRLRF KEPGDYRILL SKINATLNHL CAAFHRAWLA SQNSCIAGIY
```

```
Pp_Pphb5
Pp_Pphb5
Pp_Pphb5
Pp_sca_35
Pp_sca_35
Pp_sca_35
Pp_pphb6
Pp_pphb6
Pp_pphb6
Pp_sca_65b
Pp_sca_65b
Pp_sca_65b
Pp_sca_28
Pp_sca_28
Pp_sca_28
Pp_Pphb8
Pp_Pphb8
Pp_Pphb8
Pp_Pphb1
Pp_Pphb1
Pp_Pphb1
Pp_sca_154
Pp_sca_154
Pp_sca_154
Pp_sca_77
Pp_sca_77
Pp_sca_77
Pp_sca_4
Pp_sca_4
Pp_sca_4
Pp_sca_31    ....S..... .
Pp_sca_31    .......... .
Pp_sca_31    LARSSTSCTL S
Pp_Pphb2
Pp_Pphb2
Pp_Pphb2
Ps-ABK2449
Ps-ABK2449
Ps-ABK2449
Pg-ABA5414
Pg-ABA5414
Pg-ABA5414
Ps-ABK2476
Ps-ABK2476
Ps-ABK2476
Ps-ABK2462
Ps-ABK2462
Ps-ABK2462
Os_10g2650
Os_10g2650
Os_10g2650
Zm_4124075
Zm_4124075
Zm_4124075
Sb-XP24672
Sb-XP24672
Sb-XP24672
Zm_422699
Zm_422699
Zm_422699
Os_03g0745
Os_03g0745
Os_03g0745
Sb-XP24657
Sb-XP24657
Sb-XP24657
Zm_4113431
Zm_4113431
Zm_4113431
Zm_413795
Zm_413795
Zm_413795
Ta-TaHZI-2
Ta-TaHZI-2
Ta-TaHZI-2
Rc-XP25201
Rc-XP25201
Rc-XP25201
Cr-ABL6311
```

Cr-ABL6311
Cr-ABL6311
Vv-XP22768
Vv-XP22768
Vv-XP22768
Vv-CAO1494
Vv-CAO1494
Vv-CAO1494
Pt_687113
Pt_687113
Pt_687113
Pt_696444
Pt_696444
Pt_696444
Ha_HAHB1
Ha_HAHB1
Ha_HAHB1
At_ATHB13
At_ATHB13
At_ATHB13
Sd-AAT4051
Sd-AAT4051
Sd-AAT4051
At_AtHB23
At_AtHB23
At_AtHB23
Dc-05625.1
Dc-05625.1
Dc-05625.1
Dc-05623.1
Dc-05623.1
Dc-05623.1
Gm-ACU2401
Gm-ACU2401
Gm-ACU2401
Gm-ACU2089
Gm-ACU2089
Gm-ACU2089
Mt-ACJ8462
Mt-ACJ8462
Mt-ACJ8462
Gm-ACU1869
Gm-ACU1869
Gm-ACU1869
Gm-ACU2100
Gm-ACU2100
Gm-ACU2100
Rc-XP25174
Rc-XP25174
Rc-XP25174
Pt_736296
Pt_736296
Pt_736296
Gm-ACU1987
Gm-ACU1987
Gm-ACU1987
Gh-ABO4774
Gh-ABO4774
Gh-ABO4774
At_ATHB20
At_ATHB20
At_ATHB20
At_ATHB3
At_ATHB3
At_ATHB3
Sm_18196
Sm_18196
Sm_18196
Sm_81609
Sm_81609
Sm_81609
Cr-CRHB6
Cr-CRHB6
Cr-CRHB6
Cr-CRHB11
Cr-CRHB11
Cr-CRHB11
Cr-CRHB4
Cr-CRHB4

Cr-CRHB4
Sm_18217
Sm_18217
Sm_18217
Sm_19476
Sm_19476
Sm_19476
Ps-ABK2572
Ps-ABK2572
Ps-ABK2572
Os_03g1021
Os_03g1021
Os_03g1021
Zm_hox12
Zm_hox12
Zm_hox12
Zm_480132
Zm_480132
Zm_480132
Zm_433132
Zm_433132
Zm_433132
Zm_433210
Zm_433210
Zm_433210
Os_07g3932
Os_07g3932
Os_07g3932
Pt_655260
Pt_655260
Pt_655260
Pt_703426
Pt_703426
Pt_703426
At_ATHB53
At_ATHB53
At_ATHB53
At_ATHB40
At_ATHB40
At_ATHB40
At_ATHB21
At_ATHB21
At_ATHB21
Gm-ACU198B
Gm-ACU198B
Gm-ACU198B
Pt_98386
Pt_98386
Pt_98386
Pt_568845
Pt_568845
Pt_568845
At_ATHB52
At_ATHB52
At_ATHB52
Pt_594622
Pt_594622
Pt_594622
Pt_unknon
Pt_unknon
Pt_unknon

351
Zm_479999
Zm_479999
Zm_479999
Os_02g4333
Os_02g4333
Os_02g4333
Sb-XP24528
Sb-XP24528
Sb-XP24528
Zm_470295
Zm_470295
Zm_470295
Sb-XP24468
Sb-XP24468
Sb-XP24468
Zm_433493
Zm_433493
Zm_433493
Os_04g4581
Os_04g4581
Os_04g4581
Zm_4492
Zm_4492
Zm_4492
Sb-XP24627
Sb-XP24627
Sb-XP24627
Os_09g3591
Os_09g3591
Os_09g3591
Zm_4496
Zm_4496
Zm_4496
Vv-XP22629
Vv-XP22629
Vv-XP22629
Vv-CAN7896
Vv-CAN7896
Vv-CAN7896
Rc-XP25299
Rc-XP25299
Rc-XP25299
Mt_MTHB1
Mt_MTHB1
Mt_MTHB1
Gm-ACU2443
Gm-ACU2443
Gm-ACU2443
Pt_HB7
Pt_HB7
Pt_HB7
Pt_731421
Pt_731421
Pt_731421
At_ATHB12
At_ATHB12
At_ATHB12
At_ATHB7
At_ATHB7
At_ATHB7
Zm_hox6
Zm_hox6
Zm_hox6
Vv-XP22800
Vv-XP22800
Vv-XP22800
Pt_548258
Pt_548258
Pt_548258
Pt_343725
Pt_343725
Pt_343725
Ha_HAHB4
Ha_HAHB4
Ha_HAHB4
Vv-XP22715
Vv-XP22715
Vv-XP22715

Ha_HAHB11
Ha_HAHB11
Ha_HAHB11
Pt_HBLZ
Pt_HBLZ
Pt_HBLZ
Sb-XP24459
Sb-XP24459
Sb-XP24459
Os_08g3758
Os_08g3758
Os_08g3758
Sb-XP24603
Sb-XP24603
Sb-XP24603
Zm_4112646
Zm_4112646
Zm_4112646
Os_09g2946
Os_09g2946
Os_09g2946
Zm_469357
Zm_469357
Zm_469357
Zm_469358
Zm_469358
Zm_469358
Ta-TaHZI-1
Ta-TaHZI-1
Ta-TaHZI-1
Zm_483405
Zm_483405
Zm_483405
Os_10g2309
Os_10g2309
Os_10g2309
Zm_4102187
Zm_4102187
Zm_4102187
Zm_4118271
Zm_4118271
Zm_4118271
Os_03g0896
Os_03g0896
Os_03g0896
Cp-CPHB-4
Cp-CPHB-4
Cp-CPHB-4
At_ATHB5
At_ATHB5
At_ATHB5
Sl-SlHDL1
Sl-SlHDL1
Sl-SlHDL1
Ze-18171.1
Ze-18171.1
Ze-18171.1
Nt-Hfi22
Nt-Hfi22
Nt-Hfi22
Brs-hb-6
Brs-hb-6
Brs-hb-6
Bn-AAR0493
Bn-AAR0493
Bn-AAR0493
At_ATHB6
At_ATHB6
At_ATHB6
At_ATHB16
At_ATHB16
At_ATHB16
Pt_HD56
Pt_HD56
Pt_HD56
Pt_70493
Pt_70493
Pt_70493
Vv-XP22660

Vv-XP22660
Vv-XP22660
Mt-ACJ8504
Mt-ACJ8504
Mt-ACJ8504
Pv-HDZ1
Pv-HDZ1
Pv-HDZ1
Gm-184277-
Gm-184277-
Gm-184277-
Sl-CAB6711
Sl-CAB6711
Sl-CAB6711
Vv-CAO6167
Vv-CAO6167
Vv-CAO6167
Vv-XP22857
Vv-XP22857
Vv-XP22857
Vv-CAN8361
Vv-CAN8361
Vv-CAN8361
Pt_unknown
Pt_unknown
Pt_unknown
Pt_HB
Pt_HB
Pt_HB
Rc-XP25139
Rc-XP25139
Rc-XP25139
Os_02g4970
Os_02g4970
Os_02g4970
Zm_4134425
Zm_4134425
Zm_4134425
Zm_459693
Zm_459693
Zm_459693
Zm_459694
Zm_459694
Zm_459694
Os_08g3208
Os_08g3208
Os_08g3208
Zm_4124691
Zm_4124691
Zm_4124691
Os_09g2118
Os_09g2118
Os_09g2118
Lj-BAG5005
Lj-BAG5005
Lj-BAG5005
Pv-HDZ3
Pv-HDZ3
Pv-HDZ3
Gm-ACU1808
Gm-ACU1808
Gm-ACU1808
Rc-XP25173
Rc-XP25173
Rc-XP25173
Vv-XP22788
Vv-XP22788
Vv-XP22788
At_ATHB1
At_ATHB1
At_ATHB1
At_ATHB54
At_ATHB54
At_ATHB54
Ps-ABR1622
Ps-ABR1622
Ps-ABR1622
Sl-CAA6441
Sl-CAA6441

Sl-CAA6441	
Mt-ACJ8510	
Mt-ACJ8510	
Mt-ACJ8510	
Gm-AAX9867	
Gm-AAX9867	
Gm-AAX9867	
Dc-05624.1	
Dc-05624.1	
Dc-05624.1	
Rc-XP25115	
Rc-XP25115	
Rc-XP25115	
Pt_88244	
Pt_88244	
Pt_88244	
Vv-CAO6250	
Vv-CAO6250	
Vv-CAO6250	
Vv-CAN8396	
Vv-CAN8396	
Vv-CAN8396	
Vv-XP22716	
Vv-XP22716	
Vv-XP22716	
Cr-ABL631B	
Cr-ABL631B	
Cr-ABL631B	
Dc-05622.1	
Dc-05622.1	
Dc-05622.1	
Cp-CPHB-7	
Cp-CPHB-7	
Cp-CPHB-7	
Cp-CPHB-6	
Cp-CPHB-6	
Cp-CPHB-6	
Gm-184278-	
Gm-184278-	
Gm-184278-	
Pv-HDZ2	
Pv-HDZ2	
Pv-HDZ2	
Vv-CAO4102	
Vv-CAO4102	
Vv-CAO4102	
Rc-XP2520B	
Rc-XP2520B	
Rc-XP2520B	
Pt_HAT5	
Pt_HAT5	
Pt_HAT5	
Vs-tendril	
Vs-tendril	
Vs-tendril	
At_ATHB51	
At_ATHB51	
At_ATHB51	
At_ATHB22	
At_ATHB22	
At_ATHB22	
Pt_93443	
Pt_93443	
Pt_93443	
Pp_sca_65a	
Pp_sca_65a	
Pp_sca_65a	
Pp_sca_34	
Pp_sca_34	
Pp_sca_34	
Pp_Pphb9	
Pp_Pphb9	
Pp_Pphb9	
Pp_Pphb7	
Pp_Pphb7	
Pp_Pphb7	
Pp_sca_143	....
Pp_sca_143	....
Pp_sca_143	KLNGSNQWHH

Pp_Pphb5
Pp_Pphb5
Pp_Pphb5
Pp_sca_35
Pp_sca_35
Pp_sca_35
Pp_pphb6
Pp_pphb6
Pp_pphb6
Pp_sca_65b
Pp_sca_65b
Pp_sca_65b
Pp_sca_28
Pp_sca_28
Pp_sca_28
Pp_Pphb8
Pp_Pphb8
Pp_Pphb8
Pp_Pphb1
Pp_Pphb1
Pp_Pphb1
Pp_sca_154
Pp_sca_154
Pp_sca_154
Pp_sca_77
Pp_sca_77
Pp_sca_77
Pp_sca_4
Pp_sca_4
Pp_sca_4
Pp_sca_31
Pp_sca_31
Pp_sca_31
Pp_Pphb2
Pp_Pphb2
Pp_Pphb2
Ps-ABK2449
Ps-ABK2449
Ps-ABK2449
Pg-ABA5414
Pg-ABA5414
Pg-ABA5414
Ps-ABK2476
Ps-ABK2476
Ps-ABK2476
Ps-ABK2462
Ps-ABK2462
Ps-ABK2462
Os_10g2650
Os_10g2650
Os_10g2650
Zm_4124075
Zm_4124075
Zm_4124075
Sb-XP24672
Sb-XP24672
Sb-XP24672
Zm_422699
Zm_422699
Zm_422699
Os_03g0745
Os_03g0745
Os_03g0745
Sb-XP24657
Sb-XP24657
Sb-XP24657
Zm_4113431
Zm_4113431
Zm_4113431
Zm_413795
Zm_413795
Zm_413795
Ta-TaHZI-2
Ta-TaHZI-2
Ta-TaHZI-2
Rc-XP25201
Rc-XP25201
Rc-XP25201
Cr-ABL6311

Cr-ABL6311
Cr-ABL6311
Vv-XP22768
Vv-XP22768
Vv-XP22768
Vv-CAO1494
Vv-CAO1494
Vv-CAO1494
Pt_687113
Pt_687113
Pt_687113
Pt_696444
Pt_696444
Pt_696444
Ha_HAHB1
Ha_HAHB1
Ha_HAHB1
At_ATHB13
At_ATHB13
At_ATHB13
Sd-AAT4051
Sd-AAT4051
Sd-AAT4051
At_AtHB23
At_AtHB23
At_AtHB23
Dc-05625.1
Dc-05625.1
Dc-05625.1
Dc-05623.1
Dc-05623.1
Dc-05623.1
Gm-ACU2401
Gm-ACU2401
Gm-ACU2401
Gm-ACU2089
Gm-ACU2089
Gm-ACU2089
Mt-ACJ8462
Mt-ACJ8462
Mt-ACJ8462
Gm-ACU1869
Gm-ACU1869
Gm-ACU1869
Gm-ACU2100
Gm-ACU2100
Gm-ACU2100
Rc-XP25174
Rc-XP25174
Rc-XP25174
Pt_736296
Pt_736296
Pt_736296
Gm-ACU1987
Gm-ACU1987
Gm-ACU1987
Gh-ABO4774
Gh-ABO4774
Gh-ABO4774
At_ATHB20
At_ATHB20
At_ATHB20
At_ATHB3
At_ATHB3
At_ATHB3
Sm_18196
Sm_18196
Sm_18196
Sm_81609
Sm_81609
Sm_81609
Cr-CRHB6
Cr-CRHB6
Cr-CRHB6
Cr-CRHB11
Cr-CRHB11
Cr-CRHB11
Cr-CRHB4
Cr-CRHB4

Cr-CRHB4
Sm_18217
Sm_18217
Sm_18217
Sm_19476
Sm_19476
Sm_19476
Ps-ABK2572
Ps-ABK2572
Ps-ABK2572
Os_03g1021
Os_03g1021
Os_03g1021
Zm_hox12
Zm_hox12
Zm_hox12
Zm_480132
Zm_480132
Zm_480132
Zm_433132
Zm_433132
Zm_433132
Zm_433210
Zm_433210
Zm_433210
Os_07g3932
Os_07g3932
Os_07g3932
Pt_655260
Pt_655260
Pt_655260
Pt_703426
Pt_703426
Pt_703426
At_ATHB53
At_ATHB53
At_ATHB53
At_ATHB40
At_ATHB40
At_ATHB40
At_ATHB21
At_ATHB21
At_ATHB21
Gm-ACU198B
Gm-ACU198B
Gm-ACU198B
Pt_98386
Pt_98386
Pt_98386
Pt_568845
Pt_568845
Pt_568845
At_ATHB52
At_ATHB52
At_ATHB52
Pt_594622
Pt_594622
Pt_594622
Pt_unknon
Pt_unknon
Pt_unknon
