# Supplementary material for: Uncharacterized conserved motifs outside the HD-Zip domain in HD-Zip subfamily I transcription factors; a potential source of functional diversity
Source: BMC Plant Biol. 2011 Mar 3;11:42. doi: 10.1186/1471-2229-11-42 (PMC3060862; doi:10.1186/1471-2229-11-42)
Supplement: Additional file 10 — NTRs putative phosphorylation sites. Each of the NTRs is represented by three stacked sequences: i) only the residues predicted to be phosphorylated are visible, the remaining are substituted with dots; ii) the visible residues correspond to motifs found by the MEME program; and iii) the complete sequence is visible. [file 1471-2229-11-42-S10.PDF]

```
Alignment: /home/
Seaview [blocks=10 fontsize=10 A4] on

             1
Zm_479999
Zm_479999    .......... .......... .......... .......... .......
Zm_479999    MERGDCQFTV VPPRQYDEAQ FMHQLMVAGD QQDPAGAGRG AAAAGGE
Os_02g4333
Os_02g4333   .ESDCQFLVA PPQP...... .......... .......... .......... .......... ...
Os_02g4333   MESDCQFLVA PPQPHMYYDT AAAAVDEAQF LRQMVAAADH HAAAAGRGGG DGDGGGGGGG GGE
Sb-XP24528
Sb-XP24528   .......... .......... .......... .......... .......... .
Sb-XP24528   MERGDDCQFM VVHQYDEAAQ LMHQLMVAAA GDQQDPNAGA AGRGAGGGGG E
Zm_470295    .......... .......... .......... .......... ........T. .......... ..........
Zm_470295    .KRPGPGAKN ISCSCSPVLC S......... .......... .......... .......... ..........
Zm_470295    EKRPGPGAKN ISCSCSPVLC SSHAPRVCRI TIGRRATVGF RTLGRDRGTP EREQKRLCLP GDWQQQQQLC
Sb-XP24468   .......... .......... .......... .......... .......... .........S ....
Sb-XP24468   .....HHQHQ FFMPAPAP.. .......... .......... .......... .......... ....
Sb-XP24468   MDRPDHHQHQ FFMPAPAPAT VQVPQPQQQQ QQQICVPMMD EPSSFLAGMG GGGGGPSSSS ARGE
Zm_433493
Zm_433493    .......... .......... .......... .......... .......... .....
Zm_433493    MDRPDHQQQQ FFMPTTVQVP QPQQQQQQLC VPMLDEPPSS FLAGRGGGGA SGRGE
Os_04g4581   .......... .......... .......... .......... .......... ..Y....... ..........
Os_04g4581   .......... .......... ...QQQHQFL MPPPAP.... QLCMPAMMAD EQYMDLGGGG A.........
Os_04g4581   MNGRTQLASW ARIAMDRGDH HHLQQQHQFL MPPPAPVVPP QLCMPAMMAD EQYMDLGGGG AAAAPGRGGA
Zm_4492
Zm_4492      .......... .......... ........
Zm_4492      MDGAEDDGTE WMMHGAGGKG KGGGALDK
Sb-XP24627
Sb-XP24627   .......... .......... .......... ..
Sb-XP24627   MDGEDDVPEW MMEVGGAGGK GGKGGGGGAL DK
Os_09g3591
Os_09g3591   .......... .......... .........
Os_09g3591   MDGEEDSEWM MMDVGGKGGK GGGGGGAAD
Zm_4496
Zm_4496      .......... .......... ..........
Zm_4496      MEGDDDGPEW MMEVGGAGAT GKGKGGALDK
Vv-XP22629   .......... ...Y...... .......... ..T.....
Vv-XP22629   .......... .......... .......... ........
Vv-XP22629   MNLNEMLGGE EYKYSPMAAV DDGSLACLNS IATARKKK
Vv-CAN7896   .......... .......... .......... .......... ....Y..... .......... ..T.....
Vv-CAN7896   .......... .......... .......... .......... .......... .......... ........
Vv-CAN7896   MAVWKDTISF RLDIAPDSNG GITALIHSPV GMNLNEMLGG EEYKYSPMAA VDDGSLALNS IATARKKK
Rc-XP25299
Rc-XP25299   .......... .......... .........
Rc-XP25299   MLDGGDFSSS SAAADPFPTT ISSRRNKRN
Mt_MTHB1     ......S..S ........Y. ..S.SS.... ...
Mt_MTHB1     .......... .......... .......... ...
Mt_MTHB1     MEYSQYSSYS AEAGEEETYT TSSISSMRKK KNK
Gm-ACU2443   .......... .......S.. ...S.....
Gm-ACU2443   .......... .......... .........
Gm-ACU2443   MEYTYSAGVE AETHTSSSTT PSRSKKRNN
Pt_HB7       .......S.. .......... ..TS.....
Pt_HB7       .......... .......... .........
Pt_HB7       MFDGGEYSPS ATEPFSCMNG VTTSRKKKN
Pt_731421    .......S.. .......... ..TS.....
Pt_731421    .......... .......... .........
Pt_731421    MFDGGEYSPS ATEPFSCLNS VTTSRKKKN
At_ATHB12
At_ATHB12    .......... .......... .........
At_ATHB12    MEEGDFFNCC FSEISSGMTM NKKKMKKSN
At_ATHB7
At_ATHB7     .......... .......... .........
At_ATHB7     MTEGGEYSPA MMSAEPFLTM KKMKKSNHN
Zm_hox6
Zm_hox6      .......... .......... .......... .....
Zm_hox6      MDTRDHHHPS SNELILQQYS QAPPPGKNNS NNSSS
Vv-XP22800
Vv-XP22800   .......... ....
Vv-XP22800   METSENIATS IRKI
Pt_548258
Pt_548258    .......... .....
Pt_548258    MDPGTHPLSS IQKNA
Pt_343725
Pt_343725    .........
Pt_343725    SLSSVQKNV
Ha_HAHB4     .......... .T.......
Ha_HAHB4     .......... .........
Ha_HAHB4     MSLQQVPTTE TTTRKNRNE
```

```
Vv-XP22715   ......S... .......... ....
Vv-XP22715   .......... .......... ....
Vv-XP22715   MESRGCSAEE AGEEGEQLTR KKSR
Ha_HAHB11    ......SS.. ...S.
Ha_HAHB11    .......... .....
Ha_HAHB11    MAENSSSSIE RKKSK
Pt_HBLZ
Pt_HBLZ      .
Pt_HBLZ
Sb-XP24459   ....S..... .......... .......... .......... .......... .
Sb-XP24459   .......... .......... ........FG GVGMEDADED MVPCNGIGF. .
Sb-XP24459   MKRPSGSISG GGGGGSPDLL PVVHSDDGFG GVGMEDADED MVPCNGIGFG E
Os_08g3758
Os_08g3758   .......... .......VGV EEEMDVDEDM AMCGGRGG.. ..
Os_08g3758   MRSPAALLPV VADGGGGVGV EEEMDVDEDM AMCGGRGGGG GE
Sb-XP24603   .......S.. .......... ..Y....... .......... ..........
Sb-XP24603   .......... .......... ........EA ADADADADEE MMACGGGGG.
Sb-XP24603   MKRPRGGSPS SLAAMTNPDG DGYGVVGMEA ADADADADEE MMACGGGGGE
Zm_4112646   .........S .......... .......... .......... ..S....... .S........ ...S......
Zm_4112646   .......... .......... .......... .......... .......... .......... ..........
Zm_4112646   PRAFLDREES TGNFAIVVEN CIVRRRCMVL AMGHGREQRR AGSPAVASEF HSIEHDMKRP RGASPPLAAM
Os_09g2946   .......... .......... .S....Y... .......... ..........
Os_09g2946   .......... .......... .........V GMEAEGDVEE EMMACGGGGE
Os_09g2946   MKRPGGAGGG GGSPSLVTMA NSSDDGYGGV GMEAEGDVEE EMMACGGGGE
Zm_469357    .......T.. .......... ..Y....... .......... ..........
Zm_469357    .......... .......... .......MEA DVDVDVDEEM MAFGGGGG..
Zm_469357    MKRPRGATPS LSAMPNHQED DGYGVLGMEA DVDVDVDEEM MAFGGGGGGE
Zm_469358    .......T.. .......... .......... .......... ...S...... ....Y..... ..........
Zm_469358    .......... .......... .......... .......... .......... .........M EADVDVDVDE
Zm_469358    MKRPRGATPS LSAMPNHQEG IHCSGLLWGW VMSWRECVRN EDLSPCACVF PDDGYGVLGM EADVDVDVDE
Ta-TaHZI-1
Ta-TaHZI-1   .......... .......... .....VGMEA DGDAEEEMMA CGGGGG.... ..
Ta-TaHZI-1   MKRPGGGNPS SHLQMASPND MDYGVVGMEA DGDAEEEMMA CGGGGGGCGG GE
Zm_483405
Zm_483405    .......... .......... .GAYDEEVDE EEELAGSRGG LG.
Zm_483405    MEPHGVDGDA TAAAERDDVD AGAYDEEVDE EEELAGSRGG LGE
Os_10g2309   ...Y...... .......... .......... ..........
Os_10g2309   .......... ...EEEEEEV EDDDGGGGGG GGGG......
Os_10g2309   MRSYMDGGGA AAYEEEEEEV EDDDGGGGGG GGGGGGGLGE
Zm_4102187   .......... .S.....S.. S......... .......... .......... .......... .........S
Zm_4102187   .......... .......... .......... .......... .....DDEAE MDEDEDELGP GLGGGR....
Zm_4102187   LPKRGSQMKR QSKRPTASRE SPETGEKQKL AFAEEEAPPA RKVEPDDEAE MDEDEDELGP GLGGGRAARS
Zm_4118271   .......... .......... .......... .......... .......... .........S ........S.
Zm_4118271   .......... .......... LSTPSLTPVL QSCSA..... .......... .......... ..........
Zm_4118271   MAAWVSCLHL WPLSLSLSLS LSTPSLTPVL QSCSARKEIG ITANSFSSAA KCRSQMKRQS KRPTASRDSP
Os_03g0896   .....SSS.. S......... S......... .......... .......... .......... .S.S......
Os_03g0896   .......... .......... .......... .......... .......... .......... ..........
Os_03g0896   MKRPTSSSRK SKKQGEDLAF SEEGSLPAVT MEQKDEAEME EVDEEEEEEV DEDMAGGHAA QSPSPSCGLG
Cp-CPHB-4    .......... .........T ......S... .......... .....
Cp-CPHB-4    ....NTSETL GALISICPN. .......... .......... .....
Cp-CPHB-4    MKTQNTSETL GALISICPNT TDEERVSNVE TSAMFGLDDE CFASE
At_ATHB5     ......SS.S .........S ......S... .......... .......... ....S..... ..........
At_ATHB5     .....GSSDS LSGFLPIRHS .......... .......... .......... .......... ..........
At_ATHB5     MKRSRGSSDS LSGFLPIRHS TTDKQISPRP TTTGFLYSGA GDYSQMFDAL EDDGSLEDLG GVGHASSTAA
Sl-SlHDL1    .......... .........S ...S..S... .S...Y.S.. .......S.. .......
Sl-SlHDL1    ....GGSDSL GALISMCPA. .......... .SGRDYESMY DGMDEDGSID EG.....
Sl-SlHDL1    MKRLGGSDSL GALISMCPAS DERSPRSKHM YSGRDYESMY DGMDEDGSID EGSHIPE
Ze-18171.1
Ze-18171.1   .......... .......... ETREFHSMLM EGLDDDCGGV D......... ..
Ze-18171.1   MKRHGSSDSV GLMPMCHTLD ETREFHSMLM EGLDDDCGGV DGGECDRGLI SE
Nt-Hfi22
Nt-Hfi22     .......... ........
Nt-Hfi22     GTRLDEEGCI EESGHISE
Brs-hb-6     .....S...S .......... ......S... .......... .......... .......... .
Brs-hb-6     .....SSSDS VGGLISLCPT .......... YGREFQSMLE GYEEEEEEAV T......... .
Brs-hb-6     MMKRLSSSDS VGGLISLCPT TSTDQPSPRR YGREFQSMLE GYEEEEEEAV TEERGQTGLA E
Bn-AAR0493   .....S...S .......... ......S... .......... .......... .......... .
Bn-AAR0493   .....SSSDS VGGLISLCPT .......... YGREFQSMLE GYEEEEEEAI T......... .
Bn-AAR0493   MMKRLSSSDS VGGLISLCPT TSTDQPSPRR YGREFQSMLE GYEEEEEEAI TEERGQTGLA E
At_ATHB6     .....S...S .......... ......S... .......... .......... .......... .
At_ATHB6     .....SSSDS VGGLISLCPT .......... .GGREFQSML EGYEEEEEAI VE........ .
At_ATHB6     MMKRLSSSDS VGGLISLCPT TSTDEQSPRR YGGREFQSML EGYEEEEEAI VEERGHVGLS E
At_ATHB16    ....SS.... ......S... .S........ .......... .......... ........
At_ATHB16    ....SSSDSM CGLISTSTD. .....YGSNY QSMLEGYDED ATLIEE.... ........
At_ATHB16    MKRLSSSDSM CGLISTSTDE QSPRGYGSNY QSMLEGYDED ATLIEEYSGN HHHMGLSE
Pt_HD56      .......... T......... .......... ...S...... .......S.. .......... ........
Pt_HD56      .......... .......... .......... .......... .YSREFQSML DGLDEEGCVE EA......
Pt_HD56      MGETVEGRRG TVVYSKTVQV GLAIYMKILT EEHSPRNHTH VYSREFQSML DGLDEEGCVE EAGGHVTE
Pt_70493
```

```
Pt_70493     .......... ..........
Pt_70493     MLNGLDEEGC VEESGGHVTE
Vv-XP22660   .....S.... .......... ...S...... .......... .......... ......
Vv-XP22660   ....GSSESL GALMAICPT. .......... YSREFQTMFD GLDEEGCVEE S.....
Vv-XP22660   MKRLGSSESL GALMAICPTT DEHSPRNNHV YSREFQTMFD GLDEEGCVEE SGQIVE
Mt-ACJ8504   .......... .......... S...S..... .......... .......... .........
Mt-ACJ8504   .......... .......... .......... ..YGMEFQSM MLDGFEEEGC VEE......
Mt-ACJ8504   MKRLNNTSDS SSTPLITTSP STEEHSPRNK HVYGMEFQSM MLDGFEEEGC VEETGHHSE
Pv-HDZ1
Pv-HDZ1      .......... ..
Pv-HDZ1      EGCVEEPGHH AE
Gm-184277-
Gm-184277-   .......... .........
Gm-184277-   MLEGLDEEGC VEEPGHHSE
Sl-CAB6711   ....S.S..S .......... .......... .......... .......... .
Sl-CAB6711   .......... .......... .....YSNEF QAMLDGLEDE DGIEES.... .
Sl-CAB6711   MKRFSFSDSS GKLLYPTEEK KENLGYSNEF QAMLDGLEDE DGIEESGCGT G
Vv-CAO6167   ....S..... .......S.. .......... .......... .......... ......
Vv-CAO6167   ....SSSVSL APLVSMCSP. .......... YDSEFQAMLD SFDEEDCAEE T.....
Vv-CAO6167   MKRFSSSVSL APLVSMCSPK EGKNSKNYPV YDSEFQAMLD SFDEEDCAEE TGLITE
Vv-XP22857   ....S..... .......S.. .......... .......... .......... ......
Vv-XP22857   ....SSSVSL APLVSMCSP. .......... YDSEFQAMLD SFDEEDCAEE T.....
Vv-XP22857   MKRFSSSVSL APLVSMCSPK EGKNSKNYPV YDSEFQAMLD SFDEEDCAEE TGLITE
Vv-CAN8361
Vv-CAN8361   .......... .........
Vv-CAN8361   MLDSFDEEDC AEETGLITE
Pt_unknown   .......... .......SS. .......... .....S.... S.....Y... ......
Pt_unknown   ....NSSDPL AAFISISSS. .......... YSRDFQSMLD SLEEEDYSEA A.....
Pt_unknown   MKRFNSSDPL AAFISISSSK EERTQKTNQG YSRDFQSMLD SLEEEDYSEA ASHVGE
Pt_HB        .......... .......... .......... .......... .......... .......... ..........
Pt_HB        ........YM QVACMDKEDT QTKFKTTWLQ CSPFRLH... .......... .......... ..........
Pt_HB        ISHGKVILYM QVACMDKEDT QTKFKTTWLQ CSPFRLHPFE KAWRFGKPSE TGGGAPLLLK LIIRSVSFLL
Rc-XP25139   ....SS.... .......SS. .......... ..S....... .S........ ........
Rc-XP25139   ....SSPESF AALLSISSS. .......... .YSREFQAML DSLEEEDCTE ET......
Rc-XP25139   MKRFSSPESF AALLSISSSK EEKTTQKSNQ VYSREFQAML DSLEEEDCTE ETTLTTTE
Os_02g4970   .......... .......... .......... .......... .......... .........T ........YY
Os_02g4970   .......... .......... .......... .......... .......... ..VKRPFFTT PDELLEEEYY
Os_02g4970   MESGRLIFST AGSGAGQMLF LDCGAGGGGV GGGAMFHRGA RPVLGMEEGG RGVKRPFFTT PDELLEEEYY
Zm_4134425   .......... .......... .......... .......... .......S.. ......YY.. ....
Zm_4134425   .......... .......... .......... .......... VKRPFFTSPD ELLEEEYYDE Q...
Zm_4134425   MESGRLIFNA PGSGAGQMLF LDCGAGGGPG GGLFHREGRG VKRPFFTSPD ELLEEEYYDE QLPE
Zm_459693
Zm_459693    .......... .......... .......... .......... .......... ..GKRPFFTT HEELLEEEYY
Zm_459693    MHPSAVSFDS GDGRRRGGAQ MLLFGGGGSA NSNGFFRGVP MAVLAMDDAT RVGKRPFFTT HEELLEEEYY
Zm_459694    .......... .......... .......... .......... .......... ........TT ........YY
Zm_459694    .......... .......... .......... .......... .......... ..GKRPFFTT HEELLEEEYY
Zm_459694    MDPSAVSFDS GGTRRGGGAQ MLLFGGGGSA NSNGFFRGVP MAVLGMDDAT RVGKRPFFTT HEELLEEEYY
Os_08g3208   .......... .......... .......... .......... .......... ..SSS..... .......TT.
Os_08g3208   .......... .......... .......... .......... .......... .......... .AKRPFFTTH
Os_08g3208   MDPGRVVFDS GVARRACPGG AQMLLFGGGG SANSGGFFRG VPAAVLGMDE SRSSSSAAGA GAKRPFFTTH
Zm_4124691
Zm_4124691   .......... .......... .......... .......... .......... .......... ..........
Zm_4124691   FTGYKPTRIA ATTPHDILQP QPHSTGPAPQ TTGGRGHLPA SFSGHHRRPC KTCTDEAYLL IRRRGHGVLA
Os_09g2118   ......Y... .......... .......... .......... .......... .......... ..........
Os_09g2118   .......... .......... .......... .......... .......... .......RKR PFLTTTHDEL
Os_09g2118   MCSMDDYSGR LVFSSAGAAP PCSAAGAGGG QMLLFGGHGG FVGGSPVMEE AELRRRRRKR PFLTTTHDEL
Lj-BAG5005   .......... .......... .......... .S..S....S ......SS.. ..Y...YY.. .S..
Lj-BAG5005   .......... .......... .......... .......... KRRPFFSSPD ELYDEEYYEE Q...
Lj-BAG5005   MESGRLYFDN NRGNNMNTFF LGNADLGFRG RSMMSMEEGS KRRPFFSSPD ELYDEEYYEE QSPE
Pv-HDZ3
Pv-HDZ3      .
Pv-HDZ3
Gm-ACU1808   .......... .......... .......... ....S....S .......S.. ..Y...YY.. .S..
Gm-ACU1808   .......... .......... .......... .......... KRRPFFTSPD ELYDEEYYEK Q...
Gm-ACU1808   MESGRIFFGA SASSGNNMLF LGNTELAFRG RSIMSMEEAS KRRPFFTSPD ELYDEEYYEK QSPE
Rc-XP25173   .......... .......... .......... .......... ..TS...... .S........ Y.......
Rc-XP25173   .......... .......... ........SV FRGPRSMFKM EDT.KKRPFF SSPEDFFDDE YYDEQ...
Rc-XP25173   MDQSGRLLFN PSASSCHGGN MLFLGNCDSV FRGPRSMFKM EDTSKKRPFF SSPEDFFDDE YYDEQLPE
Vv-XP22788   .......... .......... .......... .S......TS .......S.. ......YY.. ....
Vv-XP22788   .......... .......... ....SLFRGP RSIMNIEET. KKRPFFTSPE DMFDEEYYDE Q...
Vv-XP22788   MDSCRLFFDP SSGHGNMLFL GNGDSLFRGP RSIMNIEETS KKRPFFTSPE DMFDEEYYDE QMPE
At_ATHB1     .......... .......... .......... .......... .TS....... S....Y.... Y......
At_ATHB1     .......... .......... .......VQG GGARSMMNME ET.KRRPFFS SPEDLYDDDF YDDQ...
At_ATHB1     MESNSFFFDP SASHGNSMFF LGNLNPVVQG GGARSMMNME ETSKRRPFFS SPEDLYDDDF YDDQLPE
At_ATHB54    .......... .......... .S........ .......... ..S....... .......... .......
At_ATHB54    .......... .......... .......... .....SAFYG SSSMINTETA .......... .......
At_ATHB54    MENSDTDSEV FFWFQNQNQN HSHKFPSSCF PPSSHSAFYG SSSMINTETA TMDEEDVCES YMMREIT
Ps-ABR1622
Ps-ABR1622   .......... .......... .......... .......... ..SKRPFYNT LDAEEAGDED LLD.......
```

```
Ps-ABR1622   MASNGVRFNA SNRNLIVMVN ETPSFEADST PLTSLDGVMK SVSKRPFYNT LDAEEAGDED LLDECVHQPG
Sl-CAA6441   .......... .......... .......... .......... .......... .......... .....S....
Sl-CAA6441   .......... .......... .......... .......... .....SNFLG SSSMVSFRGV ..GKRSFFDS
Sl-CAA6441   MAPGILYGGS SNFDGVFTQK QRDVFSSSTA PKGHLGSLFA PASSSSNFLG SSSMVSFRGV NGGKRSFFDS
Mt-ACJ8510   .......... .......... ........T. .......... .......... .......S.. ..........
Mt-ACJ8510   .......... .......... .......... .......... .......ASF FGSRSMVSFE DV.....RNR
Mt-ACJ8510   MAGGRVFSNG PANISNINMN ILLQNQQQTP RGNSSQQPLD SLFLSSSASF FGSRSMVSFE DVQGRKRRNR
Gm-AAX9867
Gm-AAX9867   .........
Gm-AAX9867   MDEYFHQPE
Dc-05624.1   .......... .........S .......... .........
Dc-05624.1   ........RK NPYDGFFMRS YDEEEIGDE. .........
Dc-05624.1   MVSFQDAKRK NPYDGFFMRS YDEEEIGDEE YDEYFQQPE
Rc-XP25115   .......... .......... .......... .......... ..S....... ...S...... .........S
Rc-XP25115   .......... .......... .......... .......... ...PSFLGSR SMVSFEDV.. ...STRPFFS
Rc-XP25115   MANASAGSGA GGSGSNFSVL LQSQRGACAA SQPLDAFFLS GSSPSFLGSR SMVSFEDVHQ VNGSTRPFFS
Pt_88244
Pt_88244     .......INN IGSRSMMSFA DV.....STR PFFRPYDHED NGDDDLDE.. ....
Pt_88244     MAFDLWCINN IGSRSMMSFA DVHQANGSTR PFFRPYDHED NGDDDLDEYF HQPE
Vv-CAO6250   .......... .......... .......... ..S....... ...S...... .......... ..........
Vv-CAO6250   .......... .......... .......... ...PSFLGSR SMLSFEDV.. ...PDNPFFC QFDHDENGDE
Vv-CAO6250   MASGRVNLAA MLQNQRVPCS SQPLDALFLS ASSPSFLGSR SMLSFEDVRA GKRPDNPFFC QFDHDENGDE
Vv-CAN8396   .......... ....S.S..S S......... .S..S..... ....S....S .........S ..........
Vv-CAN8396   .......... .......... .......... .......... .........S EPKGSRSMLS FEDV.....P
Vv-CAN8396   MDKVAKNMGT RSARSRSLRS SEFGSYGSLR ISLESCKYGE WEGQSRSDAS EPKGSRSMLS FEDVRAGKRP
Vv-XP22716   .......... .......... .......... ..S....... ...S...... .......... ..........
Vv-XP22716   .......... .......... .......... ...PSFLGSR SMLSFEDV.. ...PDNPFFC QFDHDENGDE
Vv-XP22716   MASGRVNLAA MLQNQRVPCS SQPLDALFLS ASSPSFLGSR SMLSFEDVRA GKRPDNPFFC QFDHDENGDE
Cr-ABL631B   .......... .S.....S.. .......... ..........
Cr-ABL631B   .......... .......... .......... ..........
Cr-ABL631B   MVNFSEAEGE NSRKREKSFF PQEEENSGNE NFDGYFHHPE
Dc-05622.1   .......... .....SS... ..S....... .....S.S.. .......... .....S.... ..........
Dc-05622.1   .......... .......... .......... .......... .......... .......... ..........
Dc-05622.1   MEIDKVYEDA DDVLLSSERP PPSSEFLDSF WISKSSPSFQ ENSASGTTTM VHLQNSPPEN GRLGGFLPSL
Cp-CPHB-7    .......... .......... .......... .......... .......... ........S. S.........
Cp-CPHB-7    .......... .......... .......... .......... .......... .......... ..........
Cp-CPHB-7    MDSCSVYGDD HPHLKNALLK NEASEDLDSL WPHDSSNPSF HGSASPAMVN FEDVRGEDSP SPQKPIFPKI
Cp-CPHB-6    .......... .......... .........S .....S..S. .......... SS........ .......S..
Cp-CPHB-6    .......... .......... .......... .......... .......... .......... ..........
Cp-CPHB-6    MNMEPICGAY DEDDDFLIKN EDDDDPLWLS PQDSSSPTSF HCLQGGSSSS SSMANLPLLP KAGKVRISSG
Gm-184278-   .......... .........Y ........
Gm-184278-   .TDRPFFQAL EKEENCDEDY EG......
Gm-184278-   MTDRPFFQAL EKEENCDEDY EGCFHQPG
Pv-HDZ2      .......... .......... .......... .......... .....S.... ........T. ..........
Pv-HDZ2      .......... .......... .......... ........AS FQGSKSMVDF ENV.....TD RPFFQALEKE
Pv-HDZ2      MAGGKLHPGS NMSLLLQNDR LPCSSEVLES LWAHTSNAAS FQGSKSMVDF ENVSGGRVTD RPFFQALEKE
Vv-CAO4102   .......... .......... ...S...... .......... .....S.... .......... .....T....
Vv-CAO4102   .......... .......... .......... ........TT FHGSKSVVNF EGV.....KD RPFFQTLHEE
Vv-CAO4102   MDGRKLYGGG SNMAVLLRKE TLPSPSELLE SFWIPDSSTT FHGSKSVVNF EGVGGDDTKD RPFFQTLHEE
Rc-XP2520B   .......... .......... .......... .......... .S........ .......... ..........
Rc-XP2520B   .......... .......... .......... ....ATIHGA KSMVNFEDV. ....MDAPFF QPLVKEENGD
Rc-XP2520B   MAGDKVSDVS NVMTTVLLQN DTLPCEPVWV PASSATIHGA KSMVNFEDVG GEDTMDAPFF QPLVKEENGD
Pt_HAT5      ........S. .......... .......... .......... .......... .......... .....SS...
Pt_HAT5      .......... .......... .......... ...STLHGAK SMVNFRND.. ..VDMSFFQP HVKEESSDED
Pt_HAT5      MAGDKDCGSS KMTIFLRNGR LPPCESLCIL TSFSTLHGAK SMVNFRNDGG DTVDMSFFQP HVKEESSDED
Vs-tendril
Vs-tendril   .......... .......... .......... .......... .......... .......... ..........
Vs-tendril   MDWNTDSTVP FVPPPGSSLS FFYNYNNYDY SGIEASEGAL AETQQRLLPV IDDDPNKINN GKDHRDKKKA
At_ATHB51    .......... .......... .......... .......... .......... ....S...S. ..........
At_ATHB51    .......... .......... .......... .......... .......... .......... ..........
At_ATHB51    MEWSTTSNVE NVRVAFMPPP WPESSSFNSL HSFNFDPYAG NSYTPGDTQT GPVISVPESE KIMNAYRFPN
At_ATHB22    ......S... .......... .......... .......... .......... .......... ..........
At_ATHB22    .......... .......... .......... .......... .......... .......... ..........
At_ATHB22    MEYWSSSFID GASSSSFISP FYNFDHFSGN QDNRCLGTMM GAQQDILHVP LAMVESGYGE ESNSFNGQEK
Pt_93443     .......... ....S..... .......... .......... .......... .......... .....
Pt_93443     .......... .......... .......... .......... .......... .......... .....
Pt_93443     MDNMDWNGNF RSFVSRPDQT SFNFLYNYAY DPQYPGMDMK HPAILAENAP NRFVPTALDK ITSQE
Pp_sca_65a   .......... .......... ......T... .......... ......S... .......... ..........
Pp_sca_65a   .......... .......... ..HRADTLVA MLGSCSP.MA LQQVPRSLGD LEDMSAGCGQ KRPYYAAYEN
Pp_sca_65a   MAVVSLGPYG GQSLNMLMQR NDHRADTLVA MLGSCSPHMA LQQVPRSLGD LEDMSAGCGQ KRPYYAAYEN
Pp_sca_34    .......... .......... .......... .......... .......... .......... ..........
Pp_sca_34    .......... .......... .......... .......... .......... .......... ..........
Pp_sca_34    MEQNLATETH KALETNPPNW KCVRLEIFPA PAHLGDDRQG KFRNCIAPVE AGEHLVDAVS ETPVVMRQHR
Pp_Pphb9     .......... .......... .......... .......... ......S..S .......... ..........
Pp_Pphb9     ........YS GQNFKLMRNI GSNTNSLVAM LQGSCSP.IA SNQVPRSMGS PKDMTKVCGQ KRPFYPTIDR
Pp_Pphb9     MAVASLSSYS GQNFKLMRNI GSNTNSLVAM LQGSCSPRIA SNQVPRSMGS PKDMTKVCGQ KRPFYPTIDR
Pp_Pphb7     .......... .......... .......... .......... ......S..S .......... .....S....
Pp_Pphb7     ........FG GQNAMLRRNI DNNTDTLISL LQGSCSP.VS MQQVPRSSES LENMMGACGQ KLPYFSSFDG
Pp_Pphb7     MVVPSLPAFG GQNAMLRRNI DNNTDTLISL LQGSCSPRVS MQQVPRSSES LENMMGACGQ KLPYFSSFDG
```

```
Pp_sca_143   ........S. ..S....... .......... .....S.... .......... ......T... ...S......
Pp_sca_143   ........SS SDSLTAELAP CFL.VGLQAP LHVGVSLEDT IVQGS.HKRP FHDACDTPTG EDGSAED...
Pp_sca_143   MLQLLCNDSS SDSLTAELAP CFLQVGLQAP LHVGVSLEDT IVQGSGHKRP FHDACDTPTG EDGSAEDDEG
Pp_Pphb5
Pp_Pphb5     .......... .......... NTSDSFAAMI GPCSP.VDLQ ALRRSGGGLG DDGQSQG..Q KRRYFASYDA
Pp_Pphb5     MAITSMGHYS GQNEVFTGNG NTSDSFAAMI GPCSPHVDLQ ALRRSGGGLG DDGQSQGGGQ KRRYFASYDA
Pp_sca_35    .......... .......... S......... .......... .......... .......... ...S......
Pp_sca_35    .......... .......... STSNSLAAML SPCLP.VDLQ ARRYGGGNLE DDGRVQG..Q KRPSFATNDS
Pp_sca_35    MAISSTGYYS GQNEMFMRDE STSNSLAAML SPCLPQVDLQ ARRYGGGNLE DDGRVQGVGQ KRPSFATNDS
Pp_pphb6     .......... .......... ..S.S..... ..T....... .........S .......... ..........
Pp_pphb6     .......... .......... ..SSSDSLAA ILTPCSP.VG LQAPPHVGGS LEDAVLVAS. ...QKRPFFA
Pp_pphb6     MAITSMGGYP CQSAMQQLIR NESSSDSLAA ILTPCSPHVG LQAPPHVGGS LEDAVLVASE GSRQKRPFFA
Pp_sca_65b
Pp_sca_65b   .......... .......... ...GSSNSLV AMLNSCNPHV SFQVSRLGGG LEDAIAGCGQ KRPFFPTFGN
Pp_sca_65b   MAMAATGLSS FGGQNVMLMR NDMGSSNSLV AMLNSCNPHV SFQVSRLGGG LEDAIAGCGQ KRPFFPTFGN
Pp_sca_28    .......... .......... .......... .......... .......... .......... ..........
Pp_sca_28    .......... .......... .RGSDSMLAM LTSCNP.... .......... .......... ..........
Pp_sca_28    MAATSLGSFG GQNVILVRND MRGSDSMLAM LTSCNPVLGF QGEFAMAIVR DPCGPIRVVS LGVLLCCGDR
Pp_Pphb8     .......... .......... .......... .......... ......S... .......... ..........
Pp_Pphb8     .......... .......... .......... .......... ......SSLN DFSSSGGGLE EEATGRGQKR
Pp_Pphb8     MAALGRAGET MSMTATGGVQ FALVYDCESG RNCCLPQFDH LKARQMSSLN DFSSSGGGLE EEATGRGQKR
Pp_Pphb1     ......S... .......... .......... .......... .......S.. ..S....... ..........
Pp_Pphb1     ......SSGS SLVAMLASCS P......... .......... .QKRPYYSTI EASGEDPGEE DV........
Pp_Pphb1     MHVRNDSSGS SLVAMLASCS PAAAVQAQPG GGGMEDTVSC GQKRPYYSTI EASGEDPGEE DVGDDCIQQS
Pp_sca_154   .......... .......... .......... .......... .......S.. .......... ..........
Pp_sca_154   .......... .......... .......... .......... .......... .......... ..........
Pp_sca_154   MIPRSHDFFI KDDILAKKIT LVSTIVTVES TVARRNEKQH RGPVRRGSGS HLFLRCVNPV PTDVSPVCHV
Pp_sca_77    ....S..S.. .......... .......... .......... ........S. .........S ...T.....S
Pp_sca_77    .......... .......... .......... .......... .......... .......... ..........
Pp_sca_77    STKVSPLSTV RLRHRDPTTF RHEGCTKDAL TRRLVIAVGI TISEGLRRSL MNAVGSSAKS DAATVRRAGS
Pp_sca_4     ........S. .......... ........S. .......... .YS.S...S. .......... ..........
Pp_sca_4     ......TASD SGIAMVASCS P......... .......... .......... .......... ..........
Pp_sca_4     MFVRNDTASD SGIAMVASCS PAAFQVSFSK KLAKMGFEKS KYSTSLSRSV KQILTFIIQH GFASSLRALI
Pp_sca_31    .......... .......... .......... .......... .......... .S........ ....S.....
Pp_sca_31    .......... .......... .......... .......... .......... ..GRKGEKEK RKRGSRG...
Pp_sca_31    MAEPTPNSRA CNEDPDQKER KAGWVEEEER KEKEGEEARG EKKKKEVKRE GSGRKGEKEK RKRGSRGVDL
Pp_Pphb2     .T...S...S .......... .......... .......... .......... .......... ..........
Pp_Pphb2     .......... .......... .......... .......... .......... .......... ..........
Pp_Pphb2     MTVRDSGSAS CREAGSRGRA ARGATTAVIK VVVVAAATAA APGGWLAGWL AGRFVNCRRL ELAEEQFRGG
Ps-ABK2449   .......... .......... .......... .......... ......S... .......... ..........
Ps-ABK2449   .......LYT SSVIMNTEDN SSAHAIAAMI ASSCTP.ATF QGTRSISVFE TG..RKRPAG NSYSALELSD
Ps-ABK2449   MACDRSALYT SSVIMNTEDN SSAHAIAAMI ASSCTPPATF QGTRSISVFE TGNERKRPAG NSYSALELSD
Pg-ABA5414   .......... .......... .......... .......... ......S... .......... ..........
Pg-ABA5414   .......LYT SSVIMNTEDN SSAHAIAAMI ASSCTP.ATF QGTRSISVFE TG..RKRPAG NSYSALELSD
Pg-ABA5414   MACDRSALYT SSVIMNTEDN SSAHAIAAMI ASSCTPPATF QGTRSISVFE TGNERKRPAG NSYSALELSD
Ps-ABK2476   .......... .......... .......... .......... ......S... .......... ..........
Ps-ABK2476   .......LYT SSVIMNTEDN SSAHAIAAMI ASSCTP.ATF QGTRSISVFE TG..RKRPAG NSYSALELSD
Ps-ABK2476   MACDRSALYT SSVIMNTEDN SSAHAIAAMI ASSCTPPATF QGTRSISVFE TGNERKRPAG NSYSALELSD
Ps-ABK2462   .....S.... .......... .......... .......... .......... ...SS.S... .......S..
Ps-ABK2462   .......... .........Y SANVMASMIA SCTS...... .......... .......... ..........
Ps-ABK2462   MSCDGSPYRY ANTAMTTEDY SANVMASMIA SCTSVGVQGA ATLTRCECEN KRKSSMSLSA YSGAMDLSDY
Os_10g2650   .......... .......... .......... .......... .......... .......... ..........
Os_10g2650   .......... .......FFP PNFLLQM... .......... ....GHGGHH LLPPPPP... ..........
Os_10g2650   MRPAMASNGA AAGAMAPFFP PNFLLQMQQP LPLHHQHLQD HAHGGHGGHH LLPPPPPSLS PFLPDLAMDA
Zm_4124075   .......... .......... .......... .......... .......... .......... ..........
Zm_4124075   .........F YPANFLLQM. .......... .......... ..EAAHHLLA PPPAA..... ..........
Zm_4124075   MATNGMAPSF YPANFLLQMQ QALPHHYQQQ EQHHEGHDDE DHEAAHHLLA PPPAALVSPF LHDFGGAMAA
Sb-XP24672   .......... .......... .......... .......... .......... .......... ..........
Sb-XP24672   .......... ..FFPANFLL QM........ .......... ..HDDHHLLA PPPPA..... ..........
Sb-XP24672   MKPMATNGMA PSFFPANFLL QMQQPLHHQQ QQEHHHHHHQ HHHDDHHLLA PPPPALVSPF LHDFGGAMAA
Zm_422699
Zm_422699    .........F FAANFLLQM. .......... ..EGHDHLLA PPPPA..... .......... ..........
Zm_422699    MATNGMAPSF FAANFLLQMQ QEQQAHHQHQ HHEGHDHLLA PPPPALVSPF LHDFGGAMTA PPPPMLATGI
Os_03g0745   .......... .......... .......... .......... .......... .......... ..........
Os_03g0745   .......... ..FFPPNFLL HM........ .....HHHHH HHGHHGHH.. ....QQHHHH LGPPPPP...
Os_03g0745   MASNGMASSP SSFFPPNFLL HMAQQQAAPP HDPQEHHHHH HHGHHGHHHE QQQQQQHHHH LGPPPPPPPH
Sb-XP24657   .......... ...S...... .......T.S .......... .......... .......... ..........
Sb-XP24657   .......... .....FFPPN FLLQM..... .......... .....HHEHH LPAPPLHP.. ..........
Sb-XP24657   MRPMASNGMA SSPSPFFPPN FLLQMQQTPS DHDPQEQQHH HHHHHHHEHH LPAPPLHPHH NPFLPSSQCP
Zm_4113431   .......... .......... .......... .........T S.....S... .......... ..........
Zm_4113431   .......... ...STTSLLL PFILTTTP.. .......... .........S SLGMAPIMLG KRPM......
Zm_4113431   MSIKNSITTT TTTSTTSLLL PFILTTTPSS PLLNAPRSRT SEVTRRSMES SLGMAPIMLG KRPMYGADVQ
Zm_413795    .......... S......... ....T.S... .......... .......... .......... ..........
Zm_413795    .......... ..FFPPNFLL QM........ .QEHHHLPAP PLHL...... ........QD FRGMAPMLRK
Zm_413795    MASNGMASSL SPFFPPNFLL QMQQTPSDHD PQEHHHLPAP PLHLHHNPFL PSSQCPSLQD FRGMAPMLRK
Ta-TaHZI-2   .......... .......... .......... .......... .......... .......... ..........
Ta-TaHZI-2   .......... ..FFPPNFLL HM........ .........H HHHEHHLPPP HP........ .......DFR
Ta-TaHZI-2   MANNGMASSP SAFFPPNFLL HMQQAPPQHD PQEHHQQHHH HHHEHHLPPP HPQHNPFLPS PQCPSLQDFR
Rc-XP25201   .......... ......T... .......... .......... .......... ....S.S... ..........
```

```
Rc-XP25201  .......FFP ANFMLQT... ....QPPTSL NPILPSCTTH DFHGVASFLG KRSM...... ........GE
Rc-XP25201  MTCNGMAFFP ANFMLQTPHE EDHHQPPTSL NPILPSCTTH DFHGVASFLG KRSMSCSGID ASCHEEANGE
Cr-ABL6311  .........S .S........ .......... .......... ...
Cr-ABL6311  ASFLGKRSMS FSGVD..... .........G EDDLSDDGSQ LGE
Cr-ABL6311  ASFLGKRSMS FSGVDICDQD GVGGGGGHGG EDDLSDDGSQ LGE
Vv-XP22768  .......... ......T... .......... .......... .......... ...S.S.... ..........
Vv-XP22768  .......FFP ANFMLQT... ...QPPNSLN PILPSCAPQD FHGVASLLGK RSM....... .....GEDDL
Vv-XP22768  MTCNGMAFFP ANFMLQTPRE DDHQPPNSLN PILPSCAPQD FHGVASLLGK RSMSFSGIDV CEETNGEDDL
Vv-CAO1494  .......... .........Y .......... .......... ......S.S. .......... ..........
Vv-CAO1494  ..FLPTDFLF QT....HPPY TSHTGLPSCT PQDFHGVIPV LMKRSM.... ........GD DDFSDDGSQA
Vv-CAO1494  MAFLPTDFLF QTHLEDHPPY TSHTGLPSCT PQDFHGVIPV LMKRSMSFSG VDGCEEVHGD DDFSDDGSQA
Pt_687113   .......... .......... .......... .......... .......... .S.S.S.... ..........
Pt_687113   .......FFP TNFMLQI... .......... .......QDF HGVASFIGKR SS........ ......GEDE
Pt_687113   MTCNGMAFFP TNFMLQISHD QDDHQPPTSL NPILPSPQDF HGVASFIGKR SSMSFSGIDA CHEEGNGEDE
Pt_696444   .......... .......... .......... .......... .......... ...S.S.S.. ..........
Pt_696444   .......FFP TNFMLQS... ....QPPTSL NPILPSCAPR DFHGVAPFLG KRSS...... ........GE
Pt_696444   MTCNGMAFFP TNFMLQSSHD QDDHQPPTSL NPILPSCAPR DFHGVAPFLG KRSSMSFSGI DVCHEEGNGE
Ha_HAHB1    .......... .......S.. .......... .......... .......... ....S..... ..........
Ha_HAHB1    .......FFS SNFMLQS... ...HAPTSLS PILPPCST.. .....AAFLG KRSMSSYSGL ..........
Ha_HAHB1    MTCTGMAFFS SNFMLQSSQE DDHHAPTSLS PILPPCSTTT QDFSGAAFLG KRSMSSYSGL NNNNMDGCDQ
At_ATHB13   .......... .......TS. .......... .......... .......... ....S..... ..........
At_ATHB13   ........FF PSNFMIQT.. .....HQSPS LAPLLPSCSL .QDLHGFASF LGKRSP.... ..........
At_ATHB13   MSCNNGMSFF PSNFMIQTSY EDDHPHQSPS LAPLLPSCSL PQDLHGFASF LGKRSPMEGC CDLETGNNMN
Sd-AAT4051  .......... .......... .......... ..S....... .........S .S........ ..........
Sd-AAT4051  ..FFPTNFML QT.......Q PSTSLNPILP SCSPQDFHGI ASFLGKRSM. .......... ....GEDDLS
Sd-AAT4051  MAFFPTNFML QTPHHEDEHQ PSTSLNPILP SCSPQDFHGI ASFLGKRSMS FSGMDGNNAC EENHGEDDLS
At_AtHB23   .......... .......... .......... .......... .S........ .......... Y.........
At_AtHB23   .........F FPENFSLQN. ........QL LQDFHGFLGK RSP....... ......GDEE YSDDGSKMGE
At_AtHB23   MSCNNNGLAF FPENFSLQNH HQEEEDHPQL LQDFHGFLGK RSPMNNVQGF CNLDMNGDEE YSDDGSKMGE
Dc-05625.1  .......... .......... .......... .......... .......... ......S.S. ..........
Dc-05625.1  ..FFPANFML HH........ .......... .......... .DFHGTIGSF LGKRSM.... ..........
Dc-05625.1  MDFFPANFML HHHQEQDHRR NHIQNPASQF IDSSQNLLPP QDFHGTIGSF LGKRSMSFSG GDHHQQQLNE
Dc-05623.1  .......... .......... .......... .......... .......... .......... S.........
Dc-05623.1  .........F FPANFMIQN. .......... .......... ..QDFHGTIG SFLGKSS... ..........
Dc-05623.1  MSTCTREMDF FPANFMIQNH QHQEQEEQVH QFPHIQNHML PPQDFHGTIG SFLGKSSMTY SRVDQHLHEE
Gm-ACU2401  .......... .......... .......... .......... .......... .......S.S ..S.......
Gm-ACU2401  .......... FFPANFMLQT .........Q PPPSLTSILP TCAPQEYHGG VTILGKRSM. ..........
Gm-ACU2401  MWHKTCNEMA FFPANFMLQT PHHDDHHHHQ PPPSLTSILP TCAPQEYHGG VTILGKRSMS FSSGIEHGEE
Gm-ACU2089  .......... .......... .......... .......... .......... S..S...... ..........
Gm-ACU2089  ..FFPANFML QT........ QPPPSLTSIL PTCAPQEYHG GATFLGKRSM .......... ...AEEDLSD
Gm-ACU2089  MAFFPANFML QTPHHDDHHH QPPPSLTSIL PTCAPQEYHG GATFLGKRSM SFSSGIEHGE EVNAEEDLSD
Mt-ACJ8462  .......... ..S....... .......... .......... .......... S.S....... ..........
Mt-ACJ8462  ..FFPANFML QT.......Q PPPSLNSIIT SCAP.DYHGG GVSFLGKRSM .......... ..VEEELSDD
Mt-ACJ8462  MAFFPANFML QTSHQDEHHQ PPPSLNSIIT SCAPQDYHGG GVSFLGKRSM SFSGIELGEE ANVEEELSDD
Gm-ACU1869  .......... .......... .......... .......... ........S. S......... ..........
Gm-ACU1869  ..FFPANFML QT......QP PPSLNSIITS CAPQEYHGGA SFLGKRSM.. .......... AEEDLSDDGS
Gm-ACU1869  MAFFPANFML QTPHQDDHQP PPSLNSIITS CAPQEYHGGA SFLGKRSMSF SGIELGEEAN AEEDLSDDGS
Gm-ACU2100  .......... .......... .......... .......... .........S .S........ ..........
Gm-ACU2100  ..FFPTNFML QT......QP PPSLNSIITS CAP....... .....KRSMS FSGIELGEEA NAEEDS....
Gm-ACU2100  MAFFPTNFML QTPHQDDHQP PPSLNSIITS CAPQEYHGGG ASFLGKRSMS FSGIELGEEA NAEEDSDDGS
Rc-XP25174  .......... .S........ .......... .......... ........S. S......... ..........
Rc-XP25174  ..FPPHGFLF HS....DHLP SPSSLNSLPS CPPHLF.... .......... .......... .GDDDLSDDG
Rc-XP25174  MAFPPHGFLF HSQEDNDHLP SPSSLNSLPS CPPHLFPGGG HFLMKRSMSF SGVEKCEEGV HGDDDLSDDG
Pt_736296   .......... ..S....... .......... .......... .......... S.S....... ..........
Pt_736296   .......... .......DHH PSPSSLNSLP SCPPQLF... .......... .......... ...GEDDLSD
Pt_736296   MAFPPHHGFM FHSHEDNDHH PSPSSLNSLP SCPPQLFPGS GGQFLMKRSM SFSGMEKCEE VGHGEDDLSD
Gm-ACU1987
Gm-ACU1987  .
Gm-ACU1987
Gh-AB04774  .......... .......... .......... .......... .......... .S.S....S. ..........
Gh-AB04774  ..FPPHAFMF QP......DH LPSPTSLNFL PSCPPQLF.. .......... .......... ...GDDELSD
Gh-AB04774  MAFPPHAFMF QPHEDHHNDH LPSPTSLNFL PSCPPQLFHG GGAPFMMKRS VSFSGVDKSE EVHGDDELSD
At_ATHB20   .......... .......... .......... .......... .......... .......S.. ........T.
At_ATHB20   .......... .......... .....QQLHE DNSQDQLPSC PPHLF..... .......... ..........
At_ATHB20   MYVFDPTTEA GLRLEMAFPQ HGFMFQQLHE DNSQDQLPSC PPHLFNGGGN YMMNRSMSLM NVQEDHNQTL
At_ATHB3    .......... .......... .......... .......... ...S...... .......... .........S
At_ATHB3    .......... .......... .......... ......DNAH HLPSPTSLPS CPPHLF.... ..........
At_ATHB3    MYMYEEERNN INNNQEGLRL EMAFPQHGFM FQQLHEDNAH HLPSPTSLPS CPPHLFYGGG GNYMMNRSMS
Sm_18196
Sm_18196    .......... ....
Sm_18196    DDDLCDESIA QHVE
Sm_81609
Sm_81609    .......... .......... ....
Sm_81609    MDHASDEEDA IDDDPGAGQQ HHIE
Cr-CRHB6
Cr-CRHB6    .....SVGST SIPQDEDETK QSVITSGISK TKGH...... .......... .......... .
Cr-CRHB6    MAWSVSVGST SIPQDEDETK QSVITSGISK TKGHNQGHRK HPSKDVTKDI GDGDELMCGV E
Cr-CRHB11
Cr-CRHB11   .......... .......... .......
```

```
Cr-CRHB11    NQGHRKHPSK DVTKDIGDGD ELMCGVE
Cr-CRHB4     .......... S......... .......... .......... ........
Cr-CRHB4     .......... .......... .......... .......... .......... .
Cr-CRHB4     MLNYSFDVKI SSSDLIPAGN YYQHVTFNEQ GDLADDDGIC YDIINSTG
Sm_18217
Sm_18217     ........GD GEVADDGSTG GG........ ..
Sm_18217     MLDSLDETGD GEVADDGSTG GGGSSGGGGF PE
Sm_19476     .......... .....S.... .......... ....
Sm_19476     .......... .......... .......... ....
Sm_19476     LRGSDALLYA TFEGSSDQES GDVEASDHEV HPVE
Ps-ABK2572   .......... .......... .......... .......... .......... S.S....... ..........
Ps-ABK2572   .......... .......... .......... .......... .......... .......... ..........
Ps-ABK2572   MSFNFGSGND HAVNGFLHLQ SGLFSRRFHA NGYGHRSGSG FYGSVLYDNP SSSDEVGLCD DEEISGGLHS
Os_03g1021   .S........ .......... ......S... .......... .......... ...S......
Os_03g1021   .......... .......... .........E QKKARQRRRR KVKP...... ..........
Os_03g1021   MSREEDEKLL FPSFAFPAEC FPEAATSGGE QKKARQRRRR KVKPEAAAAL AGESGGDEQA
Zm_hox12     .......... .......... .......S.. .......... .......... ......
Zm_hox12     .......... .......... .......... EQKKARQRRR RKPRP..... ......
Zm_hox12     MGCEEEERLL FPSFVFPESF AEAATPGSGG EQKKARQRRR RKPRPAEGGE GADEQA
Zm_480132    .......... .......... .......... .......... .........S. ...
Zm_480132    .......... .......... .......... .......... .......... ...
Zm_480132    MGCEEEDRLL FPSFAFTESF TEAGATATTP GSGEYFTYTS SLTKARQLSE GQA
Zm_433132
Zm_433132    .........E RPRARRRRRR AAWC...... .....
Zm_433132    MPNGSKAQGE RPRARRRRRR AAWCGGGELD GGGDP
Zm_433210
Zm_433210    .....EWQLM CILRRRAARC .......... ...
Zm_433210    MHLLHEWQLM CILRRRAARC GGGGGELDGG GDH
Os_07g3932   ...Y...... .......... .....S.... .......... .......... .......... .
Os_07g3932   .......... .......... .......... .ERPRARRRR RRGARC.... .......... .
Os_07g3932   MDRYGEKQQQ QQMFASYVDA SLLAASGEVQ GERPRARRRR RRGARCVGGG GGGGEVDGGD P
Pt_655260
Pt_655260    .......... .......... .....QQGES KPRRRRKKNK .......
Pt_655260    MSYKLEDHMA LTSQLYPGVF TQMVPQQGES KPRRRRKKNK DADLSGA
Pt_703426
Pt_703426    .......... .......... .....KQGES KPRRRRKKNT .......
Pt_703426    MNHKVEDHIA LISQLYPGLY TQMVPKQGES KPRRRRKKNT GEGVSGA
At_ATHB53    .......... .......... .......... .......... .......... .S...S.... ..........
At_ATHB53    .......... .......... .......... .........E SKPVKRRRKR RSKG...... ..........
At_ATHB53    MDHGRLMDDQ MMLGSQVYPY TTQPQNSHCI IVNQIDGGEE SKPVKRRRKR RSKGSSATNE EDVAEIGGML
At_ATHB40    .......... .......... .......... .........T ..S....... ....
At_ATHB40    .......... .......... ........EV KQPKRRRKKT KGS....... ....
At_ATHB40    MNYTVDDQNM AFISQLYPDV YTQIVQPGEV KQPKRRRKKT KGSVASADGG NGLF
At_ATHB21    .......... .......... .......... .......... ..S.S..... ..........
At_ATHB21    .......... .......... .......... .EAKPTRRRK RKSKSV.... ..........
At_ATHB21    MNNQNVDDHN LLLISQLYPN VYTPLVPQQG GEAKPTRRRK RKSKSVVVAE EGENEGNGWF
Gm-ACU198B
Gm-ACU198B   .......... .......... ......
Gm-ACU198B   MGDFIFSFKT QQQEHHAHHQ RAKHNN
Pt_98386
Pt_98386     ..FFNSQTQQ KQ........
Pt_98386     MDFFNSQTQQ KQQYLPKHN
Pt_568845    .......... S........
Pt_568845    .......... .........
Pt_568845    MDFFHSQNQH SPKHQNSS
At_ATHB52
At_ATHB52    ..........
At_ATHB52    MENSQSQGKN
Pt_594622
Pt_594622    .......... .........
Pt_594622    MNSHLHHQSQ SQGNRPLKP
Pt_unknon
Pt_unknon    .......... .........
Pt_unknon    MNSHLHHQSQ SHGNHALKP
```

```
               71
Zm_479999
Zm_479999
Zm_479999
Os_02g4333
Os_02g4333
Os_02g4333
Sb-XP24528
Sb-XP24528
Sb-XP24528
Zm_470295     .......... .......... .......... ..
Zm_470295     ..MMDDPSSF LAELMGVGGG GGG....... ...
Zm_470295     VPMMDDPSSF LAELMGVGGG GGGGPSSSAR GE
Sb-XP24468
Sb-XP24468
Sb-XP24468
Zm_433493
Zm_433493
Zm_433493
Os_04g4581    ..
Os_04g4581    ..
Os_04g4581    GE
Zm_4492
Zm_4492
Zm_4492
Sb-XP24627
Sb-XP24627
Sb-XP24627
Os_09g3591
Os_09g3591
Os_09g3591
Zm_4496
Zm_4496
Zm_4496
Vv-XP22629
Vv-XP22629
Vv-XP22629
Vv-CAN7896
Vv-CAN7896
Vv-CAN7896
Rc-XP25299
Rc-XP25299
Rc-XP25299
Mt_MTHB1
Mt_MTHB1
Mt_MTHB1
Gm-ACU2443
Gm-ACU2443
Gm-ACU2443
Pt_HB7
Pt_HB7
Pt_HB7
Pt_731421
Pt_731421
Pt_731421
At_ATHB12
At_ATHB12
At_ATHB12
At_ATHB7
At_ATHB7
At_ATHB7
Zm_hox6
Zm_hox6
Zm_hox6
Vv-XP22800
Vv-XP22800
Vv-XP22800
Pt_548258
Pt_548258
Pt_548258
Pt_343725
Pt_343725
Pt_343725
Ha_HAHB4
Ha_HAHB4
Ha_HAHB4
Vv-XP22715
Vv-XP22715
Vv-XP22715
```

```
Ha_HAHB11
Ha_HAHB11
Ha_HAHB11
Pt_HBLZ
Pt_HBLZ
Pt_HBLZ
Sb-XP24459
Sb-XP24459
Sb-XP24459
Os_08g3758
Os_08g3758
Os_08g3758
Sb-XP24603
Sb-XP24603
Sb-XP24603
Zm_4112646   .......Y.. .......... .......... ...
Zm_4112646   .......... ...EADADAD ADEEMMACGG GGE
Zm_4112646   TNPNDDGYGV VGMEADADAD ADEEMMACGG GGE
Os_09g2946
Os_09g2946
Os_09g2946
Zm_469357
Zm_469357
Zm_469357
Zm_469358    .......... ..
Zm_469358    EMMAFGGGGG ..
Zm_469358    EMMAFGGGGG GE
Ta-TaHZI-1
Ta-TaHZI-1
Ta-TaHZI-1
Zm_483405
Zm_483405
Zm_483405
Os_10g2309
Os_10g2309
Os_10g2309
Zm_4102187   ......
Zm_4102187   .......
Zm_4102187   PCGLGE
Zm_4118271   .......... .......... .......
Zm_4118271   .......... .......... .......
Zm_4118271   ETGTVDKQLA LAESLTARKT EPDELGE
Os_03g0896   .
Os_03g0896   .
Os_03g0896   E
Cp-CPHB-4
Cp-CPHB-4
Cp-CPHB-4
At_ATHB5     .
At_ATHB5     .
At_ATHB5     E
Sl-SlHDL1
Sl-SlHDL1
Sl-SlHDL1
Ze-18171.1
Ze-18171.1
Ze-18171.1
Nt-Hfi22
Nt-Hfi22
Nt-Hfi22
Brs-hb-6
Brs-hb-6
Brs-hb-6
Bn-AAR0493
Bn-AAR0493
Bn-AAR0493
At_ATHB6
At_ATHB6
At_ATHB6
At_ATHB16
At_ATHB16
At_ATHB16
Pt_HD56
Pt_HD56
Pt_HD56
Pt_70493
Pt_70493
Pt_70493
Vv-XP22660
```

```
Vv-XP22660
Vv-XP22660
Mt-ACJ8504
Mt-ACJ8504
Mt-ACJ8504
Pv-HDZ1
Pv-HDZ1
Pv-HDZ1
Gm-184277-
Gm-184277-
Gm-184277-
Sl-CAB6711
Sl-CAB6711
Sl-CAB6711
Vv-CAO6167
Vv-CAO6167
Vv-CAO6167
Vv-XP22857
Vv-XP22857
Vv-XP22857
Vv-CAN8361
Vv-CAN8361
Vv-CAN8361
Pt_unknown
Pt_unknown
Pt_unknown
Pt_HB        .......... .........S S......... .......... ..S.....Y. .........
Pt_HB        .......SSD SLAAFISITS SK........ ...SRDFQAM LDSLEEEDYI EEAN.....
Pt_HB        SAMKRFNSSD SLAAFISITS SKEDRTQNTN QGYSRDFQAM LDSLEEEDYI EEANLGSE
Rc-XP25139
Rc-XP25139
Rc-XP25139
Os_02g4970   ......
Os_02g4970   DEQ...
Os_02g4970   DEQLPE
Zm_4134425
Zm_4134425
Zm_4134425
Zm_459693
Zm_459693    DEQ...
Zm_459693    DEQAPE
Zm_459694    ......
Zm_459694    DEQ...
Zm_459694    DEQAPE
Os_08g3208   .......YY. .....
Os_08g3208   EELLEEEYYD EQ...
Os_08g3208   EELLEEEYYD EQAPE
Zm_4124691
Zm_4124691   .........A VEHQLLPPQV PG........ .......... .......... .......... ..........
Zm_4124691   VPHQVVDAHA VEHQLLPPQV PGRRGVLLIR LAPAARRSTS LRASRRGGGA YIQTRTAWRM DPASSLISSS
Os_09g2118   .......... .......... ....
Os_09g2118   ELQMEDLV.. .......... ....
Os_09g2118   ELQMEDLVDE LYGVDEQGSS SAAA
Lj-BAG5005
Lj-BAG5005
Lj-BAG5005
Pv-HDZ3
Pv-HDZ3
Pv-HDZ3
Gm-ACU1808
Gm-ACU1808
Gm-ACU1808
Rc-XP25173
Rc-XP25173
Rc-XP25173
Vv-XP22788
Vv-XP22788
Vv-XP22788
At_ATHB1
At_ATHB1
At_ATHB1
At_ATHB54
At_ATHB54
At_ATHB54
Ps-ABR1622
Ps-ABR1622
Ps-ABR1622
Sl-CAA6441   .......... .........
Sl-CAA6441   FDQDDNEADE LGE......
```

```
Sl-CAA6441   FDQDDNEADE LGEYLHQAE
Mt-ACJ8510   S......... .......... ....
Mt-ACJ8510   SFFGGFDLDE NGEDEMDE.. ....
Mt-ACJ8510   SFFGGFDLDE NGEDEMDEYF HQSE
Gm-AAX9867
Gm-AAX9867
Gm-AAX9867
Dc-05624.1
Dc-05624.1
Dc-05624.1
Rc-XP25115   .......... ..........
Rc-XP25115   SFDHEENADD DLDE......
Rc-XP25115   SFDHEENADD DLDEYFHQPE
Pt_88244
Pt_88244
Pt_88244
Vv-CAO6250   ..........
Vv-CAO6250   DLDE......
Vv-CAO6250   DLDEYFHQPE
Vv-CAN8396   .......... .......... ......
Vv-CAN8396   DNPFFCQFDH DENGDEDLDE ......
Vv-CAN8396   DNPFFCQFDH DENGDEDLDE YFHQPE
Vv-XP22716   ..........
Vv-XP22716   DLDE......
Vv-XP22716   DLDEYFHQPE
Cr-ABL631B
Cr-ABL631B
Cr-ABL631B
Dc-05622.1   .......... ........
Dc-05622.1   .......... ........
Dc-05622.1   EKEENGIEDY DPCLHPPE
Cp-CPHB-7    .........S .......... .
Cp-CPHB-7    .......... .......... .
Cp-CPHB-7    GNNAGKTRNS EELILDAFSS Q
Cp-CPHB-6    ........
Cp-CPHB-6    ........
Cp-CPHB-6    DISSAAAA
Gm-184278-
Gm-184278-
Gm-184278-
Pv-HDZ2      ......Y... .....
Pv-HDZ2      DNCDDDYEG. .....
Pv-HDZ2      DNCDDDYEGC FHQPG
Vv-CAO4102   .......... .....
Vv-CAO4102   ENGDEDFEG. .....
Vv-CAO4102   ENGDEDFEGC FHRPE
Rc-XP2520B   ..Y....... .
Rc-XP2520B   EDYDV..... .
Rc-XP2520B   EDYDVFLNPP A
Pt_HAT5      Y........
Pt_HAT5      YDA......
Pt_HAT5      YDAHLKPSE
Vs-tendril
Vs-tendril   ..
Vs-tendril   KN
At_ATHB51    ......
At_ATHB51    ......
At_ATHB51    NNNEMI
At_ATHB22
At_ATHB22
At_ATHB22
Pt_93443
Pt_93443
Pt_93443
Pp_sca_65a   .SS....... .......... .
Pp_sca_65a   HSSEEPEDVD .......... .
Pp_sca_65a   HSSEEPEDVD EGVDEFSHHV E
Pp_sca_34    .......... .S........ .......... .......... .......... .......... ..........
Pp_sca_34    .......... .......... .......... .......... .......... .......... ..........
Pp_sca_34    AVYAGMFLRH WSGERGIVSA SVVLSKFGFR ESVLLKNRAS VLAGLERYTH ARPWCRVQAR RLQETTGSQS
Pp_Pphb9     .S........ .......... .
Pp_Pphb9     RSVEEPDDDD .......... .
Pp_Pphb9     RSVEEPDDDD EGADEFSLRS E
Pp_Pphb7     .S........ .......... .
Pp_Pphb7     PSVEEQEDVD .......... .
Pp_Pphb7     PSVEEQEDVD EGIDEFAHHV E
Pp_sca_143   ...S...... .
Pp_sca_143   .......... .
Pp_sca_143   GDDSPTGSHN E
```

```
Pp_Pphb5
Pp_Pphb5     PKMEDGPEDA .......... .....
Pp_Pphb5     PKMEDGPEDA DEGTEDGTGN GSQLE
Pp_sca_35    .......... .......... .
Pp_sca_35    TTVEDGAEDG .......... .
Pp_sca_35    TTVEDGAEDG DEGTASASHL E
Pp_pphb6     .......... S......... .........
Pp_pphb6     TYDAPTVEDG SVED...... .........
Pp_pphb6     TYDAPTVEDG SVEDDEGADD SQGASQLE
Pp_sca_65b
Pp_sca_65b   GPGEEPEDGD .......... .
Pp_sca_65b   GPGEEPEDGD DGGDDGSQPV E
Pp_sca_28    .......... .......... .......... ....S..... .......... .........S. ..........
Pp_sca_28    .......... .......... ......VVIS AVPRSGGGLE DAIAGCGQKR LFYPTFENSP VEETEDGD..
Pp_sca_28    VWGNEDCRGI GVYSWLAKCL IEDVFSVVIS AVPRSGGGLE DAIAGCGQKR LFYPTFENSP VEETEDGDDG
Pp_Pphb8     .......... .......... .........
Pp_Pphb8     PHYPISDKGP GEEPEEGD.. .........
Pp_Pphb8     PHYPISDKGP GEEPEEGDDC CYDGAHPME
Pp_Pphb1
Pp_Pphb1
Pp_Pphb1
Pp_sca_154   .......... .......... .......... .......... .........S .......... ..........
Pp_sca_154   .......... .......... .......... ......SSTI QDASSQNSKS TKSALFRMYT TTEKP.....
Pp_sca_154   AYHPSVCNCS EELDSFPSSL NQESAIHYVV KKITSFSSTI QDASSQNSKS TKSALFRMYT TTEKPQFSET
Pp_sca_77    .......... .........S .......... .......... .....S.... .......... ......S...
Pp_sca_77    .......... .......... .......... ....LTHCAV QRADGSLEDA VTSFDKKRPF CAAFEASGED
Pp_sca_77    RKRCESVYPR NRQRGVLRKS DIGDAYLPYL TKCPLTHCAV QRADGSLEDA VTSFDKKRPF CAAFEASGED
Pp_sca_4     .......... ...
Pp_sca_4     .......... ...
Pp_sca_4     SWKCHWRQSN QPS
Pp_sca_31    ..S....... ....Y..... .......... .......... .......... .......... .......S..
Pp_sca_31    .......... .......... .......... .......... .......... .......... ..........
Pp_sca_31    RESPTKYIKY RELIYARILS SIQDFDKACL LQRGFCAASV QSQTCSTVYA LLSCALFVSE RAYICGMSKR
Pp_Pphb2     .......... ........S. .......... .......... .......... .......... ..........
Pp_Pphb2     .......... .......... .......... .......... .......... .......... ..........
Pp_Pphb2     DVEICHCCNR SRLAGITGSM RELPSAAMLG QPRLFRQQPD LSEVQGVFNP STARLDMASN VGRGGYSRGG
Ps-ABK2449   .......... ......
Ps-ABK2449   DIGDE..... ......
Ps-ABK2449   DIGDEDGSDD CIHLGE
Pg-ABA5414   .......... ......
Pg-ABA5414   DIGDE..... ......
Pg-ABA5414   DIGDEDGSDD CIHLGE
Ps-ABK2476   .......... ......
Ps-ABK2476   DIGDE..... ......
Ps-ABK2476   DIGDEDGSDD CIHLGE
Ps-ABK2462   .......... ......
Ps-ABK2462   ..GEEDGSDD CLHFGG
Ps-ABK2462   DIGEEDGSDD CLHFGG
Os_10g2650   .......... .........S .......... .......
Os_10g2650   .......... .DGGGGGAAS EDEEDGCGGG GG.....
Os_10g2650   PPPPMYEASG GDGGGGGAAS EDEEDGCGGG GGGGGGE
Zm_4124075   .......... .......... ...S...... ........
Zm_4124075   .......... .......... .......... ........
Zm_4124075   APPPPMLAGG FGGKRMYPDE QQASDDEEEG SAAVGGGE
Sb-XP24672   .......... .......... .......... ...S...... ..........
Sb-XP24672   .......... .......... .......DGG GVASDDEEVS AAAGGGCG..
Sb-XP24672   PPPMLAGGLG MGGDDSHLHA AEPQQQQDGG GVASDDEEVS AAAGGGCGGE
Zm_422699
Zm_422699    .......... .......... .......... .......... .....
Zm_422699    KRMYPDGMCD DGSGHLHAEP KQHQQDCGGG ASDDEEGSAA AACGE
Os_03g0745   .......... .......... .......... S......... .......... .........
Os_03g0745   .......... .....QEFRG MAPMLGKRPM .......... .....GEDEL SDDGSQAGE
Os_03g0745   PHNPFLPSSA QCPSLQEFRG MAPMLGKRPM SYGDGGGGGD EVNGGGEDEL SDDGSQAGE
Sb-XP24657   .......... .......... .......... .......... .......... ...
Sb-XP24657   ..QDFRGMAP MLGKRPMYGA DVVAGGGDEV NGCGGGGG.N EDELSDDGSQ AGE
Sb-XP24657   SLQDFRGMAP MLGKRPMYGA DVVAGGGDEV NGCGGGGGAN EDELSDDGSQ AGE
Zm_4113431   .......... .......... ........
Zm_4113431   .......... ...NEDELSD DGSQAGG.
Zm_4113431   VVGGEEVNGC GGVNEDELSD DGSQAGGE
Zm_413795    .......... .......... .......... .......... .
Zm_413795    RPT....... .......... ...NEDEVSD DGSLQAV... .
Zm_413795    RPTMYCGADD VGVGGEEASC GATNEDEVSD DGSLQAVGPG E
Ta-TaHZI-2   .......... .......... .......... ......T... .S.....
Ta-TaHZI-2   GGLSPMLGKR PA........ .......... ..NEEETSDD GSQLGG.
Ta-TaHZI-2   GGLSPMLGKR PAMYGGGGDG GCGGDEVTGG GANEEETSDD GSQLGGE
Rc-XP25201   .......... ..
Rc-XP25201   DDLSDDGSQA GE
Rc-XP25201   DDLSDDGSQA GE
Cr-ABL6311
```

```
Cr-ABL6311
Cr-ABL6311
Vv-XP22768   .........
Vv-XP22768   SDDGSQAGE
Vv-XP22768   SDDGSQAGE
Vv-CAO1494   ..
Vv-CAO1494   GE
Vv-CAO1494   GE
Pt_687113    ..........
Pt_687113    LSDDGSQAGE
Pt_687113    LSDDGSQAGE
Pt_696444    .......... ..
Pt_696444    DELSDDGSQA GE
Pt_696444    DELSDDGSQA GE
Ha_HAHB1     .......... .......... .
Ha_HAHB1     .....GEDEL SDDGSQLLA. .
Ha_HAHB1     EGNMNGEDEL SDDGSQLLAG E
At_ATHB13    ....Y..... ....
At_ATHB13    GEEDYSDDGS QMGE
At_ATHB13    GEEDYSDDGS QMGE
Sd-AAT4051   ........
Sd-AAT4051   DDGSQAGE
Sd-AAT4051   DDGSQAGE
At_AtHB23
At_AtHB23
At_AtHB23
Dc-05625.1   .......... ..S....... .........
Dc-05625.1   .......... .......... .........
Dc-05625.1   ESNIHGDHVD DLSDPEDDDG SGTVGSGGS
Dc-05623.1   .......... .S........ ........
Dc-05623.1   .......... .......... ........
Dc-05623.1   SNVNGDHVDD LSDADDEDGS GALGSGGS
Gm-ACU2401   .......... .......
Gm-ACU2401   ...AEEDLSD DGSQAGE
Gm-ACU2401   ANNAEEDLSD DGSQAGE
Gm-ACU2089   .......
Gm-ACU2089   DGSQAGE
Gm-ACU2089   DGSQAGE
Mt-ACJ8462   ......
Mt-ACJ8462   GSQLGE
Mt-ACJ8462   GSQLGE
Gm-ACU1869   ....
Gm-ACU1869   QAGE
Gm-ACU1869   QAGE
Gm-ACU2100   ....
Gm-ACU2100   ....
Gm-ACU2100   QAGE
Rc-XP25174   .....
Rc-XP25174   SQLGE
Rc-XP25174   SQLGE
Pt_736296    .......
Pt_736296    DGSQIGE
Pt_736296    DGSQIGE
Gm-ACU1987
Gm-ACU1987
Gm-ACU1987
Gh-ABO4774   .......
Gh-ABO4774   DGSHLGE
Gh-ABO4774   DGSHLGE
At_ATHB20    .......... ......
At_ATHB20    DEENLSDDGA HTML..
At_ATHB20    DEENLSDDGA HTMLGE
At_ATHB3     .......... T......... .......... .......... ....
At_ATHB3     .......... .......... ........EE DNLSDDGSHM ML..
At_ATHB3     FTGVSDHHHL TQKSPTTTNN MNDQDQVGEE DNLSDDGSHM MLGE
Sm_18196
Sm_18196
Sm_18196
Sm_81609
Sm_81609
Sm_81609
Cr-CRHB6
Cr-CRHB6
Cr-CRHB6
Cr-CRHB11
Cr-CRHB11
Cr-CRHB11
Cr-CRHB4
Cr-CRHB4
```

Cr-CRHB4
Sm_18217
Sm_18217
Sm_18217
Sm_19476
Sm_19476
Sm_19476
Ps-ABK2572
Ps-ABK2572
Ps-ABK2572
Os_03g1021
Os_03g1021
Os_03g1021
Zm_hox12
Zm_hox12
Zm_hox12
Zm_480132
Zm_480132
Zm_480132
Zm_433132
Zm_433132
Zm_433132
Zm_433210
Zm_433210
Zm_433210
Os_07g3932
Os_07g3932
Os_07g3932
Pt_655260
Pt_655260
Pt_655260
Pt_703426
Pt_703426
Pt_703426
At_ATHB53
At_ATHB53
At_ATHB53
At_ATHB40
At_ATHB40
At_ATHB40
At_ATHB21
At_ATHB21
At_ATHB21
Gm-ACU198B
Gm-ACU198B
Gm-ACU198B
Pt_98386
Pt_98386
Pt_98386
Pt_568845
Pt_568845
Pt_568845
At_ATHB52
At_ATHB52
At_ATHB52
Pt_594622
Pt_594622
Pt_594622
Pt_unknon
Pt_unknon
Pt_unknon

141

Zm_479999
Zm_479999
Zm_479999
Os_02g4333
Os_02g4333
Os_02g4333
Sb-XP24528
Sb-XP24528
Sb-XP24528
Zm_470295
Zm_470295
Zm_470295
Sb-XP24468
Sb-XP24468
Sb-XP24468
Zm_433493
Zm_433493
Zm_433493
Os_04g4581
Os_04g4581
Os_04g4581
Zm_4492
Zm_4492
Zm_4492
Sb-XP24627
Sb-XP24627
Sb-XP24627
Os_09g3591
Os_09g3591
Os_09g3591
Zm_4496
Zm_4496
Zm_4496
Vv-XP22629
Vv-XP22629
Vv-XP22629
Vv-CAN7896
Vv-CAN7896
Vv-CAN7896
Rc-XP25299
Rc-XP25299
Rc-XP25299
Mt_MTHB1
Mt_MTHB1
Mt_MTHB1
Gm-ACU2443
Gm-ACU2443
Gm-ACU2443
Pt_HB7
Pt_HB7
Pt_HB7
Pt_731421
Pt_731421
Pt_731421
At_ATHB12
At_ATHB12
At_ATHB12
At_ATHB7
At_ATHB7
At_ATHB7
Zm_hox6
Zm_hox6
Zm_hox6
Vv-XP22800
Vv-XP22800
Vv-XP22800
Pt_548258
Pt_548258
Pt_548258
Pt_343725
Pt_343725
Pt_343725
Ha_HAHB4
Ha_HAHB4
Ha_HAHB4
Vv-XP22715
Vv-XP22715
Vv-XP22715

Ha_HAHB11
Ha_HAHB11
Ha_HAHB11
Pt_HBLZ
Pt_HBLZ
Pt_HBLZ
Sb-XP24459
Sb-XP24459
Sb-XP24459
Os_08g3758
Os_08g3758
Os_08g3758
Sb-XP24603
Sb-XP24603
Sb-XP24603
Zm_4112646
Zm_4112646
Zm_4112646
Os_09g2946
Os_09g2946
Os_09g2946
Zm_469357
Zm_469357
Zm_469357
Zm_469358
Zm_469358
Zm_469358
Ta-TaHZI-1
Ta-TaHZI-1
Ta-TaHZI-1
Zm_483405
Zm_483405
Zm_483405
Os_10g2309
Os_10g2309
Os_10g2309
Zm_4102187
Zm_4102187
Zm_4102187
Zm_4118271
Zm_4118271
Zm_4118271
Os_03g0896
Os_03g0896
Os_03g0896
Cp-CPHB-4
Cp-CPHB-4
Cp-CPHB-4
At_ATHB5
At_ATHB5
At_ATHB5
Sl-SlHDL1
Sl-SlHDL1
Sl-SlHDL1
Ze-18171.1
Ze-18171.1
Ze-18171.1
Nt-Hfi22
Nt-Hfi22
Nt-Hfi22
Brs-hb-6
Brs-hb-6
Brs-hb-6
Bn-AAR0493
Bn-AAR0493
Bn-AAR0493
At_ATHB6
At_ATHB6
At_ATHB6
At_ATHB16
At_ATHB16
At_ATHB16
Pt_HD56
Pt_HD56
Pt_HD56
Pt_70493
Pt_70493
Pt_70493
Vv-XP22660

```
Vv-XP22660
Vv-XP22660
Mt-ACJ8504
Mt-ACJ8504
Mt-ACJ8504
Pv-HDZ1
Pv-HDZ1
Pv-HDZ1
Gm-184277-
Gm-184277-
Gm-184277-
Sl-CAB6711
Sl-CAB6711
Sl-CAB6711
Vv-CAO6167
Vv-CAO6167
Vv-CAO6167
Vv-XP22857
Vv-XP22857
Vv-XP22857
Vv-CAN8361
Vv-CAN8361
Vv-CAN8361
Pt_unknown
Pt_unknown
Pt_unknown
Pt_HB
Pt_HB
Pt_HB
Rc-XP25139
Rc-XP25139
Rc-XP25139
Os_02g4970
Os_02g4970
Os_02g4970
Zm_4134425
Zm_4134425
Zm_4134425
Zm_459693
Zm_459693
Zm_459693
Zm_459694
Zm_459694
Zm_459694
Os_08g3208
Os_08g3208
Os_08g3208
Zm_4124691
Zm_4124691    ..........  ..........  ..........  ..........  ..........  ..........  ..........
Zm_4124691    AVPFPAAGDG  GGQRQVFLFG  GGFVGGAPPL  VGGAADGRRK  RPFQLTAHDE  LQLQLQLQLD  DELCGLDYEL
Os_09g2118
Os_09g2118
Os_09g2118
Lj-BAG5005
Lj-BAG5005
Lj-BAG5005
Pv-HDZ3
Pv-HDZ3
Pv-HDZ3
Gm-ACU1808
Gm-ACU1808
Gm-ACU1808
Rc-XP25173
Rc-XP25173
Rc-XP25173
Vv-XP22788
Vv-XP22788
Vv-XP22788
At_ATHB1
At_ATHB1
At_ATHB1
At_ATHB54
At_ATHB54
At_ATHB54
Ps-ABR1622
Ps-ABR1622
Ps-ABR1622
Sl-CAA6441
Sl-CAA6441
```

```
Sl-CAA6441
Mt-ACJ8510
Mt-ACJ8510
Mt-ACJ8510
Gm-AAX9867
Gm-AAX9867
Gm-AAX9867
Dc-05624.1
Dc-05624.1
Dc-05624.1
Rc-XP25115
Rc-XP25115
Rc-XP25115
Pt_88244
Pt_88244
Pt_88244
Vv-CAO6250
Vv-CAO6250
Vv-CAO6250
Vv-CAN8396
Vv-CAN8396
Vv-CAN8396
Vv-XP22716
Vv-XP22716
Vv-XP22716
Cr-ABL631B
Cr-ABL631B
Cr-ABL631B
Dc-05622.1
Dc-05622.1
Dc-05622.1
Cp-CPHB-7
Cp-CPHB-7
Cp-CPHB-7
Cp-CPHB-6
Cp-CPHB-6
Cp-CPHB-6
Gm-184278-
Gm-184278-
Gm-184278-
Pv-HDZ2
Pv-HDZ2
Pv-HDZ2
Vv-CAO4102
Vv-CAO4102
Vv-CAO4102
Rc-XP2520B
Rc-XP2520B
Rc-XP2520B
Pt_HAT5
Pt_HAT5
Pt_HAT5
Vs-tendril
Vs-tendril
Vs-tendril
At_ATHB51
At_ATHB51
At_ATHB51
At_ATHB22
At_ATHB22
At_ATHB22
Pt_93443
Pt_93443
Pt_93443
Pp_sca_65a
Pp_sca_65a
Pp_sca_65a
Pp_sca_34     .......... .......... .......... .......... .......... .......... ..........
Pp_sca_34     .......... .......... .......... .......... .......... .....QHADT LIAMLGSRSP
Pp_sca_34     ARLNGLGMIA GPNALARSAL VKILSDAADR SENMAVASLG PYVGQNLNML LQHKDQHADT LIAMLGSRSP
Pp_Pphb9
Pp_Pphb9
Pp_Pphb9
Pp_Pphb7
Pp_Pphb7
Pp_Pphb7
Pp_sca_143
Pp_sca_143
Pp_sca_143
```

```
Pp_Pphb5
Pp_Pphb5
Pp_Pphb5
Pp_sca_35
Pp_sca_35
Pp_sca_35
Pp_pphb6
Pp_pphb6
Pp_pphb6
Pp_sca_65b
Pp_sca_65b
Pp_sca_65b
Pp_sca_28     .......
Pp_sca_28     .......
Pp_sca_28     GDEGRVE
Pp_Pphb8
Pp_Pphb8
Pp_Pphb8
Pp_Pphb1
Pp_Pphb1
Pp_Pphb1
Pp_sca_154    .......... .......... .......... .......... .......... .......... ..........
Pp_sca_154    .......... .......... .......... .......... .......... .......... ..........
Pp_sca_154    GGMRLHKASS HLYSKTCRLR DDTLASIFHN FNDAELVSLK CVSAMKCCHK VGNEERFGRK EVSGVVTIVP
Pp_sca_77     .......... .....
Pp_sca_77     PAEEEI.... .....
Pp_sca_77     PAEEEIGDDF TQQVE
Pp_sca_4
Pp_sca_4
Pp_sca_4
Pp_sca_31     .S..S..... ..S...S... .......... .......... .......... .......... ..........
Pp_sca_31     .......... .......... .......... .......... .......... .......... ..........
Pp_sca_31     DSRCSWYKKN RRSRDWSDRK VQCEDVLPRQ CWDSPSYSGT IPKQQPALLA VQGVYNPSTT WLEMANNISR
Pp_Pphb2      .........S .......... .......... ....S..... .......... .....S.... ..........
Pp_Pphb2      .........S STDTLVAMLA SCSP...... .......... ....QKRSFF PLFEASREDA GDEDL.....
Pp_Pphb2      SNVVPVRNDS STDTLVAMLA SCSPVALQVQ RTGGSLEDAV VSCGQKRSFF PLFEASREDA GDEDLGDDCT
Ps-ABK2449
Ps-ABK2449
Ps-ABK2449
Pg-ABA5414
Pg-ABA5414
Pg-ABA5414
Ps-ABK2476
Ps-ABK2476
Ps-ABK2476
Ps-ABK2462
Ps-ABK2462
Ps-ABK2462
Os_10g2650
Os_10g2650
Os_10g2650
Zm_4124075
Zm_4124075
Zm_4124075
Sb-XP24672
Sb-XP24672
Sb-XP24672
Zm_422699
Zm_422699
Zm_422699
Os_03g0745
Os_03g0745
Os_03g0745
Sb-XP24657
Sb-XP24657
Sb-XP24657
Zm_4113431
Zm_4113431
Zm_4113431
Zm_413795
Zm_413795
Zm_413795
Ta-TaHZI-2
Ta-TaHZI-2
Ta-TaHZI-2
Rc-XP25201
Rc-XP25201
Rc-XP25201
Cr-ABL6311
```

Cr-ABL6311
Cr-ABL6311
Vv-XP22768
Vv-XP22768
Vv-XP22768
Vv-CAO1494
Vv-CAO1494
Vv-CAO1494
Pt_687113
Pt_687113
Pt_687113
Pt_696444
Pt_696444
Pt_696444
Ha_HAHB1
Ha_HAHB1
Ha_HAHB1
At_ATHB13
At_ATHB13
At_ATHB13
Sd-AAT4051
Sd-AAT4051
Sd-AAT4051
At_AtHB23
At_AtHB23
At_AtHB23
Dc-05625.1
Dc-05625.1
Dc-05625.1
Dc-05623.1
Dc-05623.1
Dc-05623.1
Gm-ACU2401
Gm-ACU2401
Gm-ACU2401
Gm-ACU2089
Gm-ACU2089
Gm-ACU2089
Mt-ACJ8462
Mt-ACJ8462
Mt-ACJ8462
Gm-ACU1869
Gm-ACU1869
Gm-ACU1869
Gm-ACU2100
Gm-ACU2100
Gm-ACU2100
Rc-XP25174
Rc-XP25174
Rc-XP25174
Pt_736296
Pt_736296
Pt_736296
Gm-ACU1987
Gm-ACU1987
Gm-ACU1987
Gh-ABO4774
Gh-ABO4774
Gh-ABO4774
At_ATHB20
At_ATHB20
At_ATHB20
At_ATHB3
At_ATHB3
At_ATHB3
Sm_18196
Sm_18196
Sm_18196
Sm_81609
Sm_81609
Sm_81609
Cr-CRHB6
Cr-CRHB6
Cr-CRHB6
Cr-CRHB11
Cr-CRHB11
Cr-CRHB11
Cr-CRHB4
Cr-CRHB4

Cr-CRHB4
Sm_18217
Sm_18217
Sm_18217
Sm_19476
Sm_19476
Sm_19476
Ps-ABK2572
Ps-ABK2572
Ps-ABK2572
Os_03g1021
Os_03g1021
Os_03g1021
Zm_hox12
Zm_hox12
Zm_hox12
Zm_480132
Zm_480132
Zm_480132
Zm_433132
Zm_433132
Zm_433132
Zm_433210
Zm_433210
Zm_433210
Os_07g3932
Os_07g3932
Os_07g3932
Pt_655260
Pt_655260
Pt_655260
Pt_703426
Pt_703426
Pt_703426
At_ATHB53
At_ATHB53
At_ATHB53
At_ATHB40
At_ATHB40
At_ATHB40
At_ATHB21
At_ATHB21
At_ATHB21
Gm-ACU198B
Gm-ACU198B
Gm-ACU198B
Pt_98386
Pt_98386
Pt_98386
Pt_568845
Pt_568845
Pt_568845
At_ATHB52
At_ATHB52
At_ATHB52
Pt_594622
Pt_594622
Pt_594622
Pt_unknon
Pt_unknon
Pt_unknon

211
Zm_479999
Zm_479999
Zm_479999
Os_02g4333
Os_02g4333
Os_02g4333
Sb-XP24528
Sb-XP24528
Sb-XP24528
Zm_470295
Zm_470295
Zm_470295
Sb-XP24468
Sb-XP24468
Sb-XP24468
Zm_433493
Zm_433493
Zm_433493
Os_04g4581
Os_04g4581
Os_04g4581
Zm_4492
Zm_4492
Zm_4492
Sb-XP24627
Sb-XP24627
Sb-XP24627
Os_09g3591
Os_09g3591
Os_09g3591
Zm_4496
Zm_4496
Zm_4496
Vv-XP22629
Vv-XP22629
Vv-XP22629
Vv-CAN7896
Vv-CAN7896
Vv-CAN7896
Rc-XP25299
Rc-XP25299
Rc-XP25299
Mt_MTHB1
Mt_MTHB1
Mt_MTHB1
Gm-ACU2443
Gm-ACU2443
Gm-ACU2443
Pt_HB7
Pt_HB7
Pt_HB7
Pt_731421
Pt_731421
Pt_731421
At_ATHB12
At_ATHB12
At_ATHB12
At_ATHB7
At_ATHB7
At_ATHB7
Zm_hox6
Zm_hox6
Zm_hox6
Vv-XP22800
Vv-XP22800
Vv-XP22800
Pt_548258
Pt_548258
Pt_548258
Pt_343725
Pt_343725
Pt_343725
Ha_HAHB4
Ha_HAHB4
Ha_HAHB4
Vv-XP22715
Vv-XP22715
Vv-XP22715

Ha_HAHB11
Ha_HAHB11
Ha_HAHB11
Pt_HBLZ
Pt_HBLZ
Pt_HBLZ
Sb-XP24459
Sb-XP24459
Sb-XP24459
Os_08g3758
Os_08g3758
Os_08g3758
Sb-XP24603
Sb-XP24603
Sb-XP24603
Zm_4112646
Zm_4112646
Zm_4112646
Os_09g2946
Os_09g2946
Os_09g2946
Zm_469357
Zm_469357
Zm_469357
Zm_469358
Zm_469358
Zm_469358
Ta-TaHZI-1
Ta-TaHZI-1
Ta-TaHZI-1
Zm_483405
Zm_483405
Zm_483405
Os_10g2309
Os_10g2309
Os_10g2309
Zm_4102187
Zm_4102187
Zm_4102187
Zm_4118271
Zm_4118271
Zm_4118271
Os_03g0896
Os_03g0896
Os_03g0896
Cp-CPHB-4
Cp-CPHB-4
Cp-CPHB-4
At_ATHB5
At_ATHB5
At_ATHB5
Sl-SlHDL1
Sl-SlHDL1
Sl-SlHDL1
Ze-18171.1
Ze-18171.1
Ze-18171.1
Nt-Hfi22
Nt-Hfi22
Nt-Hfi22
Brs-hb-6
Brs-hb-6
Brs-hb-6
Bn-AAR0493
Bn-AAR0493
Bn-AAR0493
At_ATHB6
At_ATHB6
At_ATHB6
At_ATHB16
At_ATHB16
At_ATHB16
Pt_HD56
Pt_HD56
Pt_HD56
Pt_70493
Pt_70493
Pt_70493
Vv-XP22660

```
Vv-XP22660
Vv-XP22660
Mt-ACJ8504
Mt-ACJ8504
Mt-ACJ8504
Pv-HDZ1
Pv-HDZ1
Pv-HDZ1
Gm-184277-
Gm-184277-
Gm-184277-
Sl-CAB6711
Sl-CAB6711
Sl-CAB6711
Vv-CAO6167
Vv-CAO6167
Vv-CAO6167
Vv-XP22857
Vv-XP22857
Vv-XP22857
Vv-CAN8361
Vv-CAN8361
Vv-CAN8361
Pt_unknown
Pt_unknown
Pt_unknown
Pt_HB
Pt_HB
Pt_HB
Rc-XP25139
Rc-XP25139
Rc-XP25139
Os_02g4970
Os_02g4970
Os_02g4970
Zm_4134425
Zm_4134425
Zm_4134425
Zm_459693
Zm_459693
Zm_459693
Zm_459694
Zm_459694
Zm_459694
Os_08g3208
Os_08g3208
Os_08g3208
Zm_4124691
Zm_4124691    .......
Zm_4124691    HGPQQE
Os_09g2118
Os_09g2118
Os_09g2118
Lj-BAG5005
Lj-BAG5005
Lj-BAG5005
Pv-HDZ3
Pv-HDZ3
Pv-HDZ3
Gm-ACU1808
Gm-ACU1808
Gm-ACU1808
Rc-XP25173
Rc-XP25173
Rc-XP25173
Vv-XP22788
Vv-XP22788
Vv-XP22788
At_ATHB1
At_ATHB1
At_ATHB1
At_ATHB54
At_ATHB54
At_ATHB54
Ps-ABR1622
Ps-ABR1622
Ps-ABR1622
Sl-CAA6441
Sl-CAA6441
```

```
Sl-CAA6441
Mt-ACJ8510
Mt-ACJ8510
Mt-ACJ8510
Gm-AAX9867
Gm-AAX9867
Gm-AAX9867
Dc-05624.1
Dc-05624.1
Dc-05624.1
Rc-XP25115
Rc-XP25115
Rc-XP25115
Pt_88244
Pt_88244
Pt_88244
Vv-CAO6250
Vv-CAO6250
Vv-CAO6250
Vv-CAN8396
Vv-CAN8396
Vv-CAN8396
Vv-XP22716
Vv-XP22716
Vv-XP22716
Cr-ABL631B
Cr-ABL631B
Cr-ABL631B
Dc-05622.1
Dc-05622.1
Dc-05622.1
Cp-CPHB-7
Cp-CPHB-7
Cp-CPHB-7
Cp-CPHB-6
Cp-CPHB-6
Cp-CPHB-6
Gm-184278-
Gm-184278-
Gm-184278-
Pv-HDZ2
Pv-HDZ2
Pv-HDZ2
Vv-CAO4102
Vv-CAO4102
Vv-CAO4102
Rc-XP2520B
Rc-XP2520B
Rc-XP2520B
Pt_HAT5
Pt_HAT5
Pt_HAT5
Vs-tendril
Vs-tendril
Vs-tendril
At_ATHB51
At_ATHB51
At_ATHB51
At_ATHB22
At_ATHB22
At_ATHB22
Pt_93443
Pt_93443
Pt_93443
Pp_sca_65a
Pp_sca_65a
Pp_sca_65a
Pp_sca_34    .........S ..........  ..........  ....S..... Y......... ....
Pp_sca_34    .MSLQQVPRS LEDLEDMNAG CVLKRPYYTA YENPSSLETD YAD....... ....
Pp_sca_34    QMSLQQVPRS LEDLEDMNAG CVLKRPYYTA YENPSSLETD YADDGCDEFS HRVE
Pp_Pphb9
Pp_Pphb9
Pp_Pphb9
Pp_Pphb7
Pp_Pphb7
Pp_Pphb7
Pp_sca_143
Pp_sca_143
Pp_sca_143
```

```
Pp_Pphb5
Pp_Pphb5
Pp_Pphb5
Pp_sca_35
Pp_sca_35
Pp_sca_35
Pp_pphb6
Pp_pphb6
Pp_pphb6
Pp_sca_65b
Pp_sca_65b
Pp_sca_65b
Pp_sca_28
Pp_sca_28
Pp_sca_28
Pp_Pphb8
Pp_Pphb8
Pp_Pphb8
Pp_Pphb1
Pp_Pphb1
Pp_Pphb1
Pp_sca_154   ..........  ..........  ..........  ......S...  ..........  S.........  ..........
Pp_sca_154   ..........  ..........  ..........  ..........  ..........  SSTDTLVAML  ASCSS.....
Pp_sca_154   LGAQGKCEDL  RRQCWDAKTF  SSVFSGTTWI  GMASTVSGYG  GPNVMLVRNE  SSTDTLVAML  ASCSSVQLQV
Pp_sca_77
Pp_sca_77
Pp_sca_77
Pp_sca_4
Pp_sca_4
Pp_sca_4
Pp_sca_31    ..........  ......S...  ..........  ..........  .........S  ..S...ST..  ..........
Pp_sca_31    ..........  ......SSTD  TLVAMLASCS  P.........  ..........  ..........  ..........
Pp_sca_31    GGFGHGSSNV  MLVRNESSTD  TLVAMLASCS  PAALQENHYG  LIRVTLLQHS  EKSERRSTMR  QLAVVMTRYH
Pp_Pphb2     ....
Pp_Pphb2     ....
Pp_Pphb2     HNVE
Ps-ABK2449
Ps-ABK2449
Ps-ABK2449
Pg-ABA5414
Pg-ABA5414
Pg-ABA5414
Ps-ABK2476
Ps-ABK2476
Ps-ABK2476
Ps-ABK2462
Ps-ABK2462
Ps-ABK2462
Os_10g2650
Os_10g2650
Os_10g2650
Zm_4124075
Zm_4124075
Zm_4124075
Sb-XP24672
Sb-XP24672
Sb-XP24672
Zm_422699
Zm_422699
Zm_422699
Os_03g0745
Os_03g0745
Os_03g0745
Sb-XP24657
Sb-XP24657
Sb-XP24657
Zm_4113431
Zm_4113431
Zm_4113431
Zm_413795
Zm_413795
Zm_413795
Ta-TaHZI-2
Ta-TaHZI-2
Ta-TaHZI-2
Rc-XP25201
Rc-XP25201
Rc-XP25201
Cr-ABL6311
```

Cr-ABL6311
Cr-ABL6311
Vv-XP22768
Vv-XP22768
Vv-XP22768
Vv-CAO1494
Vv-CAO1494
Vv-CAO1494
Pt_687113
Pt_687113
Pt_687113
Pt_696444
Pt_696444
Pt_696444
Ha_HAHB1
Ha_HAHB1
Ha_HAHB1
At_ATHB13
At_ATHB13
At_ATHB13
Sd-AAT4051
Sd-AAT4051
Sd-AAT4051
At_AtHB23
At_AtHB23
At_AtHB23
Dc-05625.1
Dc-05625.1
Dc-05625.1
Dc-05623.1
Dc-05623.1
Dc-05623.1
Gm-ACU2401
Gm-ACU2401
Gm-ACU2401
Gm-ACU2089
Gm-ACU2089
Gm-ACU2089
Mt-ACJ8462
Mt-ACJ8462
Mt-ACJ8462
Gm-ACU1869
Gm-ACU1869
Gm-ACU1869
Gm-ACU2100
Gm-ACU2100
Gm-ACU2100
Rc-XP25174
Rc-XP25174
Rc-XP25174
Pt_736296
Pt_736296
Pt_736296
Gm-ACU1987
Gm-ACU1987
Gm-ACU1987
Gh-ABO4774
Gh-ABO4774
Gh-ABO4774
At_ATHB20
At_ATHB20
At_ATHB20
At_ATHB3
At_ATHB3
At_ATHB3
Sm_18196
Sm_18196
Sm_18196
Sm_81609
Sm_81609
Sm_81609
Cr-CRHB6
Cr-CRHB6
Cr-CRHB6
Cr-CRHB11
Cr-CRHB11
Cr-CRHB11
Cr-CRHB4
Cr-CRHB4

Cr-CRHB4
Sm_18217
Sm_18217
Sm_18217
Sm_19476
Sm_19476
Sm_19476
Ps-ABK2572
Ps-ABK2572
Ps-ABK2572
Os_03g1021
Os_03g1021
Os_03g1021
Zm_hox12
Zm_hox12
Zm_hox12
Zm_480132
Zm_480132
Zm_480132
Zm_433132
Zm_433132
Zm_433132
Zm_433210
Zm_433210
Zm_433210
Os_07g3932
Os_07g3932
Os_07g3932
Pt_655260
Pt_655260
Pt_655260
Pt_703426
Pt_703426
Pt_703426
At_ATHB53
At_ATHB53
At_ATHB53
At_ATHB40
At_ATHB40
At_ATHB40
At_ATHB21
At_ATHB21
At_ATHB21
Gm-ACU198B
Gm-ACU198B
Gm-ACU198B
Pt_98386
Pt_98386
Pt_98386
Pt_568845
Pt_568845
Pt_568845
At_ATHB52
At_ATHB52
At_ATHB52
Pt_594622
Pt_594622
Pt_594622
Pt_unknon
Pt_unknon
Pt_unknon

281
Zm_479999
Zm_479999
Zm_479999
Os_02g4333
Os_02g4333
Os_02g4333
Sb-XP24528
Sb-XP24528
Sb-XP24528
Zm_470295
Zm_470295
Zm_470295
Sb-XP24468
Sb-XP24468
Sb-XP24468
Zm_433493
Zm_433493
Zm_433493
Os_04g4581
Os_04g4581
Os_04g4581
Zm_4492
Zm_4492
Zm_4492
Sb-XP24627
Sb-XP24627
Sb-XP24627
Os_09g3591
Os_09g3591
Os_09g3591
Zm_4496
Zm_4496
Zm_4496
Vv-XP22629
Vv-XP22629
Vv-XP22629
Vv-CAN7896
Vv-CAN7896
Vv-CAN7896
Rc-XP25299
Rc-XP25299
Rc-XP25299
Mt_MTHB1
Mt_MTHB1
Mt_MTHB1
Gm-ACU2443
Gm-ACU2443
Gm-ACU2443
Pt_HB7
Pt_HB7
Pt_HB7
Pt_731421
Pt_731421
Pt_731421
At_ATHB12
At_ATHB12
At_ATHB12
At_ATHB7
At_ATHB7
At_ATHB7
Zm_hox6
Zm_hox6
Zm_hox6
Vv-XP22800
Vv-XP22800
Vv-XP22800
Pt_548258
Pt_548258
Pt_548258
Pt_343725
Pt_343725
Pt_343725
Ha_HAHB4
Ha_HAHB4
Ha_HAHB4
Vv-XP22715
Vv-XP22715
Vv-XP22715

Ha_HAHB11
Ha_HAHB11
Ha_HAHB11
Pt_HBLZ
Pt_HBLZ
Pt_HBLZ
Sb-XP24459
Sb-XP24459
Sb-XP24459
Os_08g3758
Os_08g3758
Os_08g3758
Sb-XP24603
Sb-XP24603
Sb-XP24603
Zm_4112646
Zm_4112646
Zm_4112646
Os_09g2946
Os_09g2946
Os_09g2946
Zm_469357
Zm_469357
Zm_469357
Zm_469358
Zm_469358
Zm_469358
Ta-TaHZI-1
Ta-TaHZI-1
Ta-TaHZI-1
Zm_483405
Zm_483405
Zm_483405
Os_10g2309
Os_10g2309
Os_10g2309
Zm_4102187
Zm_4102187
Zm_4102187
Zm_4118271
Zm_4118271
Zm_4118271
Os_03g0896
Os_03g0896
Os_03g0896
Cp-CPHB-4
Cp-CPHB-4
Cp-CPHB-4
At_ATHB5
At_ATHB5
At_ATHB5
Sl-SlHDL1
Sl-SlHDL1
Sl-SlHDL1
Ze-18171.1
Ze-18171.1
Ze-18171.1
Nt-Hfi22
Nt-Hfi22
Nt-Hfi22
Brs-hb-6
Brs-hb-6
Brs-hb-6
Bn-AAR0493
Bn-AAR0493
Bn-AAR0493
At_ATHB6
At_ATHB6
At_ATHB6
At_ATHB16
At_ATHB16
At_ATHB16
Pt_HD56
Pt_HD56
Pt_HD56
Pt_70493
Pt_70493
Pt_70493
Vv-XP22660

Vv-XP22660
Vv-XP22660
Mt-ACJ8504
Mt-ACJ8504
Mt-ACJ8504
Pv-HDZ1
Pv-HDZ1
Pv-HDZ1
Gm-184277-
Gm-184277-
Gm-184277-
Sl-CAB6711
Sl-CAB6711
Sl-CAB6711
Vv-CAO6167
Vv-CAO6167
Vv-CAO6167
Vv-XP22857
Vv-XP22857
Vv-XP22857
Vv-CAN8361
Vv-CAN8361
Vv-CAN8361
Pt_unknown
Pt_unknown
Pt_unknown
Pt_HB
Pt_HB
Pt_HB
Rc-XP25139
Rc-XP25139
Rc-XP25139
Os_02g4970
Os_02g4970
Os_02g4970
Zm_4134425
Zm_4134425
Zm_4134425
Zm_459693
Zm_459693
Zm_459693
Zm_459694
Zm_459694
Zm_459694
Os_08g3208
Os_08g3208
Os_08g3208
Zm_4124691
Zm_4124691
Zm_4124691
Os_09g2118
Os_09g2118
Os_09g2118
Lj-BAG5005
Lj-BAG5005
Lj-BAG5005
Pv-HDZ3
Pv-HDZ3
Pv-HDZ3
Gm-ACU1808
Gm-ACU1808
Gm-ACU1808
Rc-XP25173
Rc-XP25173
Rc-XP25173
Vv-XP22788
Vv-XP22788
Vv-XP22788
At_ATHB1
At_ATHB1
At_ATHB1
At_ATHB54
At_ATHB54
At_ATHB54
Ps-ABR1622
Ps-ABR1622
Ps-ABR1622
Sl-CAA6441
Sl-CAA6441

Sl-CAA6441
Mt-ACJ8510
Mt-ACJ8510
Mt-ACJ8510
Gm-AAX9867
Gm-AAX9867
Gm-AAX9867
Dc-05624.1
Dc-05624.1
Dc-05624.1
Rc-XP25115
Rc-XP25115
Rc-XP25115
Pt_88244
Pt_88244
Pt_88244
Vv-CAO6250
Vv-CAO6250
Vv-CAO6250
Vv-CAN8396
Vv-CAN8396
Vv-CAN8396
Vv-XP22716
Vv-XP22716
Vv-XP22716
Cr-ABL631B
Cr-ABL631B
Cr-ABL631B
Dc-05622.1
Dc-05622.1
Dc-05622.1
Cp-CPHB-7
Cp-CPHB-7
Cp-CPHB-7
Cp-CPHB-6
Cp-CPHB-6
Cp-CPHB-6
Gm-184278-
Gm-184278-
Gm-184278-
Pv-HDZ2
Pv-HDZ2
Pv-HDZ2
Vv-CAO4102
Vv-CAO4102
Vv-CAO4102
Rc-XP2520B
Rc-XP2520B
Rc-XP2520B
Pt_HAT5
Pt_HAT5
Pt_HAT5
Vs-tendril
Vs-tendril
Vs-tendril
At_ATHB51
At_ATHB51
At_ATHB51
At_ATHB22
At_ATHB22
At_ATHB22
Pt_93443
Pt_93443
Pt_93443
Pp_sca_65a
Pp_sca_65a
Pp_sca_65a
Pp_sca_34
Pp_sca_34
Pp_sca_34
Pp_Pphb9
Pp_Pphb9
Pp_Pphb9
Pp_Pphb7
Pp_Pphb7
Pp_Pphb7
Pp_sca_143
Pp_sca_143
Pp_sca_143

```
Pp_Pphb5
Pp_Pphb5
Pp_Pphb5
Pp_sca_35
Pp_sca_35
Pp_sca_35
Pp_pphb6
Pp_pphb6
Pp_pphb6
Pp_sca_65b
Pp_sca_65b
Pp_sca_65b
Pp_sca_28
Pp_sca_28
Pp_sca_28
Pp_Pphb8
Pp_Pphb8
Pp_Pphb8
Pp_Pphb1
Pp_Pphb1
Pp_Pphb1
Pp_sca_154   .......... ....S..... .S....S... .......... ....
Pp_sca_154   .......... .....QKRPY YSSFEASGED PGDEEI.... ....
Pp_sca_154   QLAGGGLEDA VASCSQKRPY YSSFEASGED PGDEEIEDCT QQVE
Pp_sca_77
Pp_sca_77
Pp_sca_77
Pp_sca_4
Pp_sca_4
Pp_sca_4
Pp_sca_31    .......... .......... ....S..... .......... .......... .......... ..S.......
Pp_sca_31    .......... .......... .......... VMQCAVQRGG GGLEDALVSS GQKRSFFPTF EASGEDAGDE
Pp_sca_31    VCNWPGSYFS VRYSLLVIWS VCAISKYVTE VMQCAVQRGG GGLEDALVSS GQKRSFFPTF EASGEDAGDE
Pp_Pphb2
Pp_Pphb2
Pp_Pphb2
Ps-ABK2449
Ps-ABK2449
Ps-ABK2449
Pg-ABA5414
Pg-ABA5414
Pg-ABA5414
Ps-ABK2476
Ps-ABK2476
Ps-ABK2476
Ps-ABK2462
Ps-ABK2462
Ps-ABK2462
Os_10g2650
Os_10g2650
Os_10g2650
Zm_4124075
Zm_4124075
Zm_4124075
Sb-XP24672
Sb-XP24672
Sb-XP24672
Zm_422699
Zm_422699
Zm_422699
Os_03g0745
Os_03g0745
Os_03g0745
Sb-XP24657
Sb-XP24657
Sb-XP24657
Zm_4113431
Zm_4113431
Zm_4113431
Zm_413795
Zm_413795
Zm_413795
Ta-TaHZI-2
Ta-TaHZI-2
Ta-TaHZI-2
Rc-XP25201
Rc-XP25201
Rc-XP25201
Cr-ABL6311
```

Cr-ABL6311
Cr-ABL6311
Vv-XP22768
Vv-XP22768
Vv-XP22768
Vv-CAO1494
Vv-CAO1494
Vv-CAO1494
Pt_687113
Pt_687113
Pt_687113
Pt_696444
Pt_696444
Pt_696444
Ha_HAHB1
Ha_HAHB1
Ha_HAHB1
At_ATHB13
At_ATHB13
At_ATHB13
Sd-AAT4051
Sd-AAT4051
Sd-AAT4051
At_AtHB23
At_AtHB23
At_AtHB23
Dc-05625.1
Dc-05625.1
Dc-05625.1
Dc-05623.1
Dc-05623.1
Dc-05623.1
Gm-ACU2401
Gm-ACU2401
Gm-ACU2401
Gm-ACU2089
Gm-ACU2089
Gm-ACU2089
Mt-ACJ8462
Mt-ACJ8462
Mt-ACJ8462
Gm-ACU1869
Gm-ACU1869
Gm-ACU1869
Gm-ACU2100
Gm-ACU2100
Gm-ACU2100
Rc-XP25174
Rc-XP25174
Rc-XP25174
Pt_736296
Pt_736296
Pt_736296
Gm-ACU1987
Gm-ACU1987
Gm-ACU1987
Gh-ABO4774
Gh-ABO4774
Gh-ABO4774
At_ATHB20
At_ATHB20
At_ATHB20
At_ATHB3
At_ATHB3
At_ATHB3
Sm_18196
Sm_18196
Sm_18196
Sm_81609
Sm_81609
Sm_81609
Cr-CRHB6
Cr-CRHB6
Cr-CRHB6
Cr-CRHB11
Cr-CRHB11
Cr-CRHB11
Cr-CRHB4
Cr-CRHB4

Cr-CRHB4
Sm_18217
Sm_18217
Sm_18217
Sm_19476
Sm_19476
Sm_19476
Ps-ABK2572
Ps-ABK2572
Ps-ABK2572
Os_03g1021
Os_03g1021
Os_03g1021
Zm_hox12
Zm_hox12
Zm_hox12
Zm_480132
Zm_480132
Zm_480132
Zm_433132
Zm_433132
Zm_433132
Zm_433210
Zm_433210
Zm_433210
Os_07g3932
Os_07g3932
Os_07g3932
Pt_655260
Pt_655260
Pt_655260
Pt_703426
Pt_703426
Pt_703426
At_ATHB53
At_ATHB53
At_ATHB53
At_ATHB40
At_ATHB40
At_ATHB40
At_ATHB21
At_ATHB21
At_ATHB21
Gm-ACU198B
Gm-ACU198B
Gm-ACU198B
Pt_98386
Pt_98386
Pt_98386
Pt_568845
Pt_568845
Pt_568845
At_ATHB52
At_ATHB52
At_ATHB52
Pt_594622
Pt_594622
Pt_594622
Pt_unknon
Pt_unknon
Pt_unknon

351
Zm_479999
Zm_479999
Zm_479999
Os_02g4333
Os_02g4333
Os_02g4333
Sb-XP24528
Sb-XP24528
Sb-XP24528
Zm_470295
Zm_470295
Zm_470295
Sb-XP24468
Sb-XP24468
Sb-XP24468
Zm_433493
Zm_433493
Zm_433493
Os_04g4581
Os_04g4581
Os_04g4581
Zm_4492
Zm_4492
Zm_4492
Sb-XP24627
Sb-XP24627
Sb-XP24627
Os_09g3591
Os_09g3591
Os_09g3591
Zm_4496
Zm_4496
Zm_4496
Vv-XP22629
Vv-XP22629
Vv-XP22629
Vv-CAN7896
Vv-CAN7896
Vv-CAN7896
Rc-XP25299
Rc-XP25299
Rc-XP25299
Mt_MTHB1
Mt_MTHB1
Mt_MTHB1
Gm-ACU2443
Gm-ACU2443
Gm-ACU2443
Pt_HB7
Pt_HB7
Pt_HB7
Pt_731421
Pt_731421
Pt_731421
At_ATHB12
At_ATHB12
At_ATHB12
At_ATHB7
At_ATHB7
At_ATHB7
Zm_hox6
Zm_hox6
Zm_hox6
Vv-XP22800
Vv-XP22800
Vv-XP22800
Pt_548258
Pt_548258
Pt_548258
Pt_343725
Pt_343725
Pt_343725
Ha_HAHB4
Ha_HAHB4
Ha_HAHB4
Vv-XP22715
Vv-XP22715
Vv-XP22715

Ha_HAHB11
Ha_HAHB11
Ha_HAHB11
Pt_HBLZ
Pt_HBLZ
Pt_HBLZ
Sb-XP24459
Sb-XP24459
Sb-XP24459
Os_08g3758
Os_08g3758
Os_08g3758
Sb-XP24603
Sb-XP24603
Sb-XP24603
Zm_4112646
Zm_4112646
Zm_4112646
Os_09g2946
Os_09g2946
Os_09g2946
Zm_469357
Zm_469357
Zm_469357
Zm_469358
Zm_469358
Zm_469358
Ta-TaHZI-1
Ta-TaHZI-1
Ta-TaHZI-1
Zm_483405
Zm_483405
Zm_483405
Os_10g2309
Os_10g2309
Os_10g2309
Zm_4102187
Zm_4102187
Zm_4102187
Zm_4118271
Zm_4118271
Zm_4118271
Os_03g0896
Os_03g0896
Os_03g0896
Cp-CPHB-4
Cp-CPHB-4
Cp-CPHB-4
At_ATHB5
At_ATHB5
At_ATHB5
Sl-SlHDL1
Sl-SlHDL1
Sl-SlHDL1
Ze-18171.1
Ze-18171.1
Ze-18171.1
Nt-Hfi22
Nt-Hfi22
Nt-Hfi22
Brs-hb-6
Brs-hb-6
Brs-hb-6
Bn-AAR0493
Bn-AAR0493
Bn-AAR0493
At_ATHB6
At_ATHB6
At_ATHB6
At_ATHB16
At_ATHB16
At_ATHB16
Pt_HD56
Pt_HD56
Pt_HD56
Pt_70493
Pt_70493
Pt_70493
Vv-XP22660

Vv-XP22660
Vv-XP22660
Mt-ACJ8504
Mt-ACJ8504
Mt-ACJ8504
Pv-HDZ1
Pv-HDZ1
Pv-HDZ1
Gm-184277-
Gm-184277-
Gm-184277-
Sl-CAB6711
Sl-CAB6711
Sl-CAB6711
Vv-CAO6167
Vv-CAO6167
Vv-CAO6167
Vv-XP22857
Vv-XP22857
Vv-XP22857
Vv-CAN8361
Vv-CAN8361
Vv-CAN8361
Pt_unknown
Pt_unknown
Pt_unknown
Pt_HB
Pt_HB
Pt_HB
Rc-XP25139
Rc-XP25139
Rc-XP25139
Os_02g4970
Os_02g4970
Os_02g4970
Zm_4134425
Zm_4134425
Zm_4134425
Zm_459693
Zm_459693
Zm_459693
Zm_459694
Zm_459694
Zm_459694
Os_08g3208
Os_08g3208
Os_08g3208
Zm_4124691
Zm_4124691
Zm_4124691
Os_09g2118
Os_09g2118
Os_09g2118
Lj-BAG5005
Lj-BAG5005
Lj-BAG5005
Pv-HDZ3
Pv-HDZ3
Pv-HDZ3
Gm-ACU1808
Gm-ACU1808
Gm-ACU1808
Rc-XP25173
Rc-XP25173
Rc-XP25173
Vv-XP22788
Vv-XP22788
Vv-XP22788
At_ATHB1
At_ATHB1
At_ATHB1
At_ATHB54
At_ATHB54
At_ATHB54
Ps-ABR1622
Ps-ABR1622
Ps-ABR1622
Sl-CAA6441
Sl-CAA6441

Sl-CAA6441
Mt-ACJ8510
Mt-ACJ8510
Mt-ACJ8510
Gm-AAX9867
Gm-AAX9867
Gm-AAX9867
Dc-05624.1
Dc-05624.1
Dc-05624.1
Rc-XP25115
Rc-XP25115
Rc-XP25115
Pt_88244
Pt_88244
Pt_88244
Vv-CAO6250
Vv-CAO6250
Vv-CAO6250
Vv-CAN8396
Vv-CAN8396
Vv-CAN8396
Vv-XP22716
Vv-XP22716
Vv-XP22716
Cr-ABL631B
Cr-ABL631B
Cr-ABL631B
Dc-05622.1
Dc-05622.1
Dc-05622.1
Cp-CPHB-7
Cp-CPHB-7
Cp-CPHB-7
Cp-CPHB-6
Cp-CPHB-6
Cp-CPHB-6
Gm-184278-
Gm-184278-
Gm-184278-
Pv-HDZ2
Pv-HDZ2
Pv-HDZ2
Vv-CAO4102
Vv-CAO4102
Vv-CAO4102
Rc-XP2520B
Rc-XP2520B
Rc-XP2520B
Pt_HAT5
Pt_HAT5
Pt_HAT5
Vs-tendril
Vs-tendril
Vs-tendril
At_ATHB51
At_ATHB51
At_ATHB51
At_ATHB22
At_ATHB22
At_ATHB22
Pt_93443
Pt_93443
Pt_93443
Pp_sca_65a
Pp_sca_65a
Pp_sca_65a
Pp_sca_34
Pp_sca_34
Pp_sca_34
Pp_Pphb9
Pp_Pphb9
Pp_Pphb9
Pp_Pphb7
Pp_Pphb7
Pp_Pphb7
Pp_sca_143
Pp_sca_143
Pp_sca_143

```
Pp_Pphb5
Pp_Pphb5
Pp_Pphb5
Pp_sca_35
Pp_sca_35
Pp_sca_35
Pp_pphb6
Pp_pphb6
Pp_pphb6
Pp_sca_65b
Pp_sca_65b
Pp_sca_65b
Pp_sca_28
Pp_sca_28
Pp_sca_28
Pp_Pphb8
Pp_Pphb8
Pp_Pphb8
Pp_Pphb1
Pp_Pphb1
Pp_Pphb1
Pp_sca_154
Pp_sca_154
Pp_sca_154
Pp_sca_77
Pp_sca_77
Pp_sca_77
Pp_sca_4
Pp_sca_4
Pp_sca_4
Pp_sca_31    .......... .
Pp_sca_31    DL........ .
Pp_sca_31    DLGDDCTHNV E
Pp_Pphb2
Pp_Pphb2
Pp_Pphb2
Ps-ABK2449
Ps-ABK2449
Ps-ABK2449
Pg-ABA5414
Pg-ABA5414
Pg-ABA5414
Ps-ABK2476
Ps-ABK2476
Ps-ABK2476
Ps-ABK2462
Ps-ABK2462
Ps-ABK2462
Os_10g2650
Os_10g2650
Os_10g2650
Zm_4124075
Zm_4124075
Zm_4124075
Sb-XP24672
Sb-XP24672
Sb-XP24672
Zm_422699
Zm_422699
Zm_422699
Os_03g0745
Os_03g0745
Os_03g0745
Sb-XP24657
Sb-XP24657
Sb-XP24657
Zm_4113431
Zm_4113431
Zm_4113431
Zm_413795
Zm_413795
Zm_413795
Ta-TaHZI-2
Ta-TaHZI-2
Ta-TaHZI-2
Rc-XP25201
Rc-XP25201
Rc-XP25201
Cr-ABL6311
```

Cr-ABL6311
Cr-ABL6311
Vv-XP22768
Vv-XP22768
Vv-XP22768
Vv-CAO1494
Vv-CAO1494
Vv-CAO1494
Pt_687113
Pt_687113
Pt_687113
Pt_696444
Pt_696444
Pt_696444
Ha_HAHB1
Ha_HAHB1
Ha_HAHB1
At_ATHB13
At_ATHB13
At_ATHB13
Sd-AAT4051
Sd-AAT4051
Sd-AAT4051
At_AtHB23
At_AtHB23
At_AtHB23
Dc-05625.1
Dc-05625.1
Dc-05625.1
Dc-05623.1
Dc-05623.1
Dc-05623.1
Gm-ACU2401
Gm-ACU2401
Gm-ACU2401
Gm-ACU2089
Gm-ACU2089
Gm-ACU2089
Mt-ACJ8462
Mt-ACJ8462
Mt-ACJ8462
Gm-ACU1869
Gm-ACU1869
Gm-ACU1869
Gm-ACU2100
Gm-ACU2100
Gm-ACU2100
Rc-XP25174
Rc-XP25174
Rc-XP25174
Pt_736296
Pt_736296
Pt_736296
Gm-ACU1987
Gm-ACU1987
Gm-ACU1987
Gh-ABO4774
Gh-ABO4774
Gh-ABO4774
At_ATHB20
At_ATHB20
At_ATHB20
At_ATHB3
At_ATHB3
At_ATHB3
Sm_18196
Sm_18196
Sm_18196
Sm_81609
Sm_81609
Sm_81609
Cr-CRHB6
Cr-CRHB6
Cr-CRHB6
Cr-CRHB11
Cr-CRHB11
Cr-CRHB11
Cr-CRHB4
Cr-CRHB4

Cr-CRHB4
Sm_18217
Sm_18217
Sm_18217
Sm_19476
Sm_19476
Sm_19476
Ps-ABK2572
Ps-ABK2572
Ps-ABK2572
Os_03g1021
Os_03g1021
Os_03g1021
Zm_hox12
Zm_hox12
Zm_hox12
Zm_480132
Zm_480132
Zm_480132
Zm_433132
Zm_433132
Zm_433132
Zm_433210
Zm_433210
Zm_433210
Os_07g3932
Os_07g3932
Os_07g3932
Pt_655260
Pt_655260
Pt_655260
Pt_703426
Pt_703426
Pt_703426
At_ATHB53
At_ATHB53
At_ATHB53
At_ATHB40
At_ATHB40
At_ATHB40
At_ATHB21
At_ATHB21
At_ATHB21
Gm-ACU198B
Gm-ACU198B
Gm-ACU198B
Pt_98386
Pt_98386
Pt_98386
Pt_568845
Pt_568845
Pt_568845
At_ATHB52
At_ATHB52
At_ATHB52
Pt_594622
Pt_594622
Pt_594622
Pt_unknon
Pt_unknon
Pt_unknon
